# A Substrate-Dependent Redox Catalysis of Tellurenyl Species Involving Oxidation States +I, +II, and +IV

**DOI:** 10.1021/acs.inorgchem.5c05915

**Published:** 2026-03-26

**Authors:** Martin Hejda, Lukáš Doležal, Aleš Růžička, Emanuel Hupf, Jens Beckmann, Libor Dostál

**Affiliations:** † Department of General and Inorganic Chemistry, 48252 University of Pardubice, Studentská 573, Pardubice CZ 532 10, Czech Republic; ‡ Institut für Anorganische Chemie und Kristallographie, 9168 Universität Bremen, Leobener Straße 7, Bremen 28359, Germany

## Abstract

Neutral Te­(II) species 2-(*t*BuNCH)­C_6_H_4_TeCl (**[I]­Cl**) as well as tellurenyl Te­(II)^+^ cations [2-(*t*BuNCH)­C_6_H_4_Te]­[X] (X = OTf or SbF_6_), i.e., **[I]­[OTf]** and **[I]­[SbF**_**6**_ **]**, exhibit a wide range of reactivity toward various *ortho*-quinones. While the reaction of *ortho*-chloranil leads to the oxidation of Te­(II) into Te­(IV), 3,5-di-*tert*-butyl-*ortho*-benzoquinone leads only to partial oxidation due to an ongoing dynamic reversible reaction, being the first example of a chemically reversible two electron Te­(II)/Te­(IV) redox couple. By contrast, 9,10-phenanthrenequinone as a very weak oxidant shows no reaction; however, the most Lewis acidic **[I]­[SbF**_**6**_ **]** produces a corresponding Lewis adduct interacting by both electrostatic and weak chalcogen bond interactions. The diverse reactivity scope is further complemented by DFT computed thermochemistry data. Furthermore, **[I]­[OTf]** is successfully utilized for the catalytic transfer of silanes (Et_3_SiH, Ph_3_SiH, Ph_2_SiH_2_, and (EtO)_3_SiH) to *ortho*-quinones via redox single or double Si–H bond activation, yielding silylated catechols, monomeric cyclic catecholatosilanes, or bis­(catecholato)­silanes. Surprisingly, in the absence of the catalyst **[I]­[OTf]**, the reactivity spans from no reaction to the formation of hexachloro-dibenzo­[1,4]­dioxine-2,3-dione. The latter product is an entirely different substance class compared to the one formed during the catalyzed reaction.

## Graphical abstract

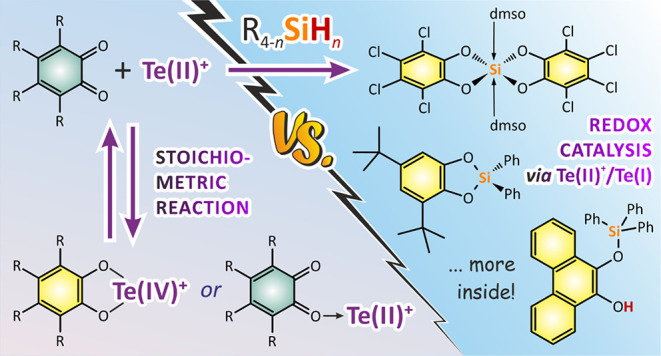

## Introduction

The role of organotellurium compounds in catalysis gained a significant boost in attention in recent years. Tellurium’s ability to change its oxidation state rather easily makes organotellurium species ideal candidates as main group redox catalysts, which have been utilized in various catalytic oxidations.^1^ Besides redox reactions, the catalytic activity of Te species was shown to be stemming from other properties, such as chalcogen bond interactions^2^ of Lewis acidic neutral diorganotellurium (R_2_Te­(II)) compounds,^3^ triorganotelluronium (R_3_Te­(IV)^+^),^3b,4^ or diorganohydroxytelluronium (R_2_(HO)­Te­(IV)^+^)^5^ salts, as well as mixed-valence telluride/telluronium salts^6^ with the substrate as catalysts. We recently demonstrated the unprecedented tellurenyl cation **[I]­[OTf]**-mediated redox Si–H bond activation, converting the silanes R_3_SiH (R = Et, TMS, Ph) into the corresponding silyl triflates. This process is governed by a Te­(II)/Te­(I) redox couple, as was proven by the formation of the monoiminium ditelluride **II** as a byproduct of the conversion (Scheme 1A).^7^ This stoichiometric reaction was then extended to utilize **[I]­[OTf]** as the tellurium-redox catalyst to produce siloxylated phenols by the reaction of silanes with *para*-quinones, including the unprecedented activation of all four Si–H bonds in SiH_4_ (Scheme 1B).^8^

**Scheme 1:**
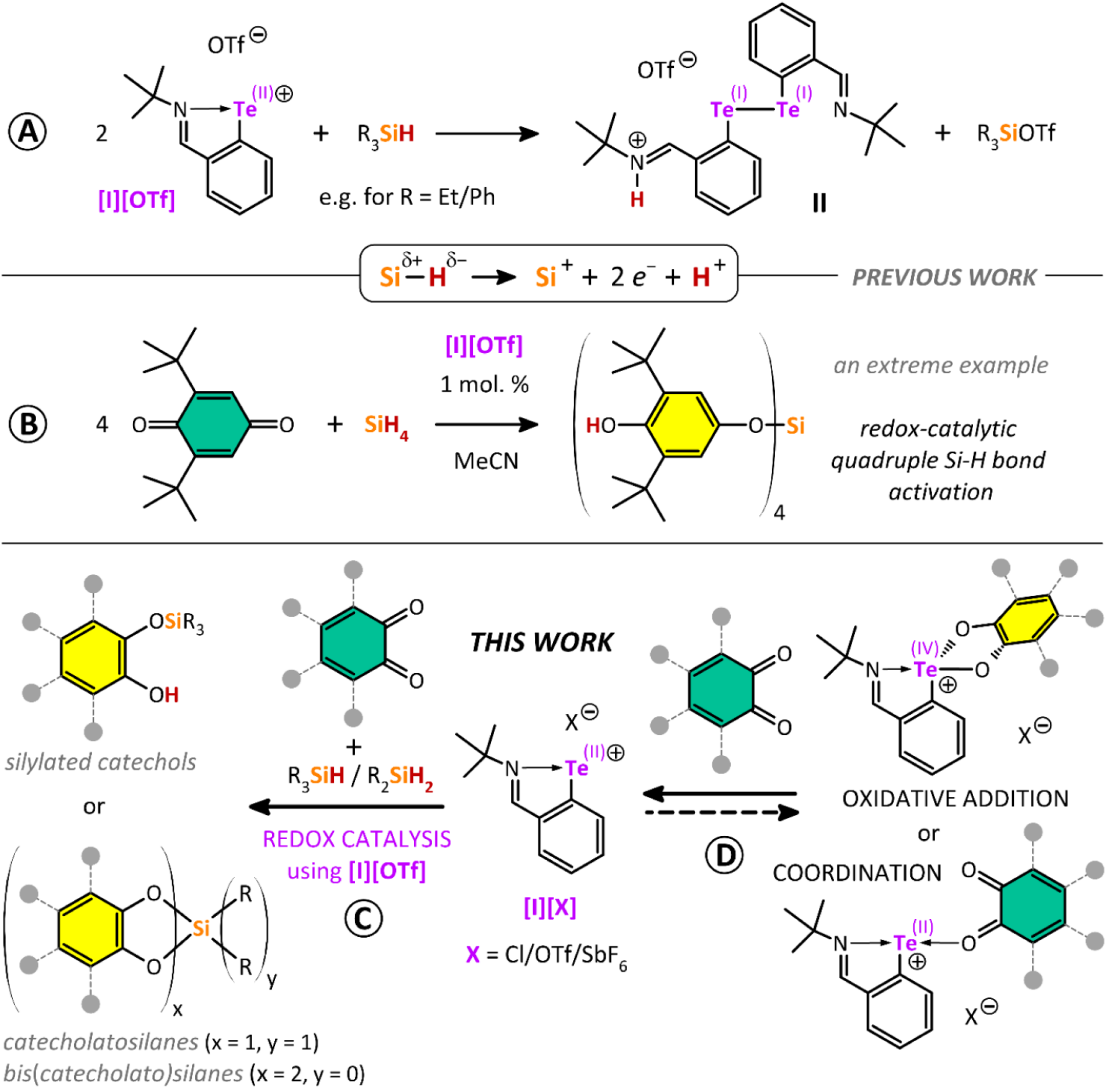
Previously Reported Stoichiometric Redox Si–H Bond Activation Mediated by Tellurenyl Triflate **[I]­[OTf]** ^7,13^ (A; Adapted from Ref 8. Copyright (2025) The Authors. Published by Wiley under a Creative Commons Attribution (CC BY) license.) and Its Application in Redox Catalysis Using *Para*-Quinones as Both Oxidants and Substrates^7,8^ (B), Key Results of the Current Study Showing **[I]­[OTf]**-Catalyzed Reaction of *ortho*-Quinones with Various Silanes (C) and Stoichiometric Reactivity of Tellurenyl Chloride/Triflate/Hexafluoroantimonate with *ortho*-Quinones (D)

Herein, we further extend this investigation to *ortho*-quinones, namely *ortho*-chloranil (***o*** **-q** ^**Cl**^), 3,5-di-*tert*-butyl-*ortho*-benzoquinone (***o*** **-q** ^***t*** **Bu**^), and 9,10-phenanthrenequinone (***o*** **-q** ^**phen**^), aiming at the production of various novel silylated catechols and catecholatosilanes (Scheme 1C). Catecholatosilanes and bis­(catecholato)­silanes have been extensively studied in recent years due to their extremely high Lewis acidity, stemming from silicon’s tendency for hypercoordination in the bis­(catecholato) coordination environment.^9^

Unexpectedly, we also found that the neutral telluride **[I]­Cl** as well as the tellurenyl cations **[I]­[OTf]** and **[I]­[SbF**_**6**_ **]** can be directly oxidized by *ortho*-quinones giving the corresponding Te­(IV) catecholato species (Scheme 1D), meaning, that under catalytic conditions, this process must compete with the Si–H bond activation mediated by the tellurenyl cation. This contrasts with the inertness of the tellurenyl cation **[I]­[OTf]** toward *para*-quinones reported previously.^8^ It is known that various tellurium species, including ditellurides,^10^ Te­(II) species,^11^ or even elemental tellurium,^11,12^ can be irreversibly oxidized by *ortho*-quinones. This study demonstrates that the Te­(II)/Te­(IV) redox process can also be reversible (Scheme 1D).

## Results and Discussion

### Stoichiometric Te­(II)/Te­(IV) Reactions

The oxidation of **[I]­Cl**, **[I]­[OTf]**, and **[I]­[SbF**_**6**_ **]** by ***o*** **-q** ^**Cl**^ smoothly provided the expected tellurium­(IV) complexes with the catecholate framework attached to the tellurium center, i.e., **[I­(cat** ^**Cl**^ **)]­Cl**, **[I­(cat** ^**Cl**^ **)]­[OTf],** and **[I­(cat** ^**Cl**^ **)]­[SbF**_**6**_ **]**, in essentially quantitative yields (Scheme 2). All products revealed high sensitivity to moisture, but are indefinitely stable when stored under rigorously anhydrous conditions. The molecular structure of **[I­(cat** ^**Cl**^ **)]­Cl** is shown in Figure 1. The spatial arrangement around the Te atom is square-pyramidal with the ligand *ipso*-carbon atom in the axial position. The N1–Te1 bond (2.418(2) Å) is longer than in the precursor **[I]­Cl** (2.203(2) Å)^13^ and in the related tellurium­(IV) trichloride 2-(*t*BuNCH)­C_6_H_4_TeCl_3_ (2.286(1) Å).^14^ All C–C bonds within the catechol unit span over a narrow range of 1.380(4)–1.405(4) Å, proving its aromatic character. The ^1^H and ^13^C­{^1^H} NMR spectra for **[I­(cat** ^**Cl**^ **)]­Cl**, **[I­(cat** ^**Cl**^ **)]­[OTf]** in DCM-*d*_2_ and **[I­(cat** ^**Cl**^ **)]­[SbF**_**6**_ **]** in MeCN-*d*_3_ (due to the insolubility in DCM-*d*_2_) showed the expected set of signals, including the *CH*=N moiety of the ligand (see ). The respective ^125^Te­{^1^H} NMR spectra revealed one signal at 1484.8/1540.4/1611.1 ppm for **[I­(cat** ^**Cl**^ **)]­Cl**/**[I­(cat** ^**Cl**^ **)]­[OTf]**/**[I­(cat** ^**Cl**^ **)]­[SbF**_**6**_ **]**, respectively, being noticeably shifted compared to the tellurium­(II) precursors (cf. 1302.1/1735.2/1896.5 ppm for **[I]­Cl**/**[I]­[OTf]**/**[I]­[SbF**_**6**_ **]**). These values are more deshielded in comparison to related tellurium­(IV) species synthesized earlier, i.e., 2-(*t*BuNCH)­C_6_H_4_TeCl_3_ (1176.9 ppm)^14^ or 2-(*t*BuNCH)­C_6_H_4_TeCl_2_OTf (1373.1 ppm).^15^ Interestingly, while **[I]­Cl** serves as a precursor for **[I]­[OTf]** and **[I]­[SbF**_**6**_ **]** using AgX (X = OTf/SbF_6_; Scheme 2) as a chloride abstraction reagent,^13^ the same method does not apply for **[I­(cat** ^**Cl**^ **)]­Cl** which is inert toward AgX, indicating a rather strong Te–Cl bond.

**Scheme 2:**
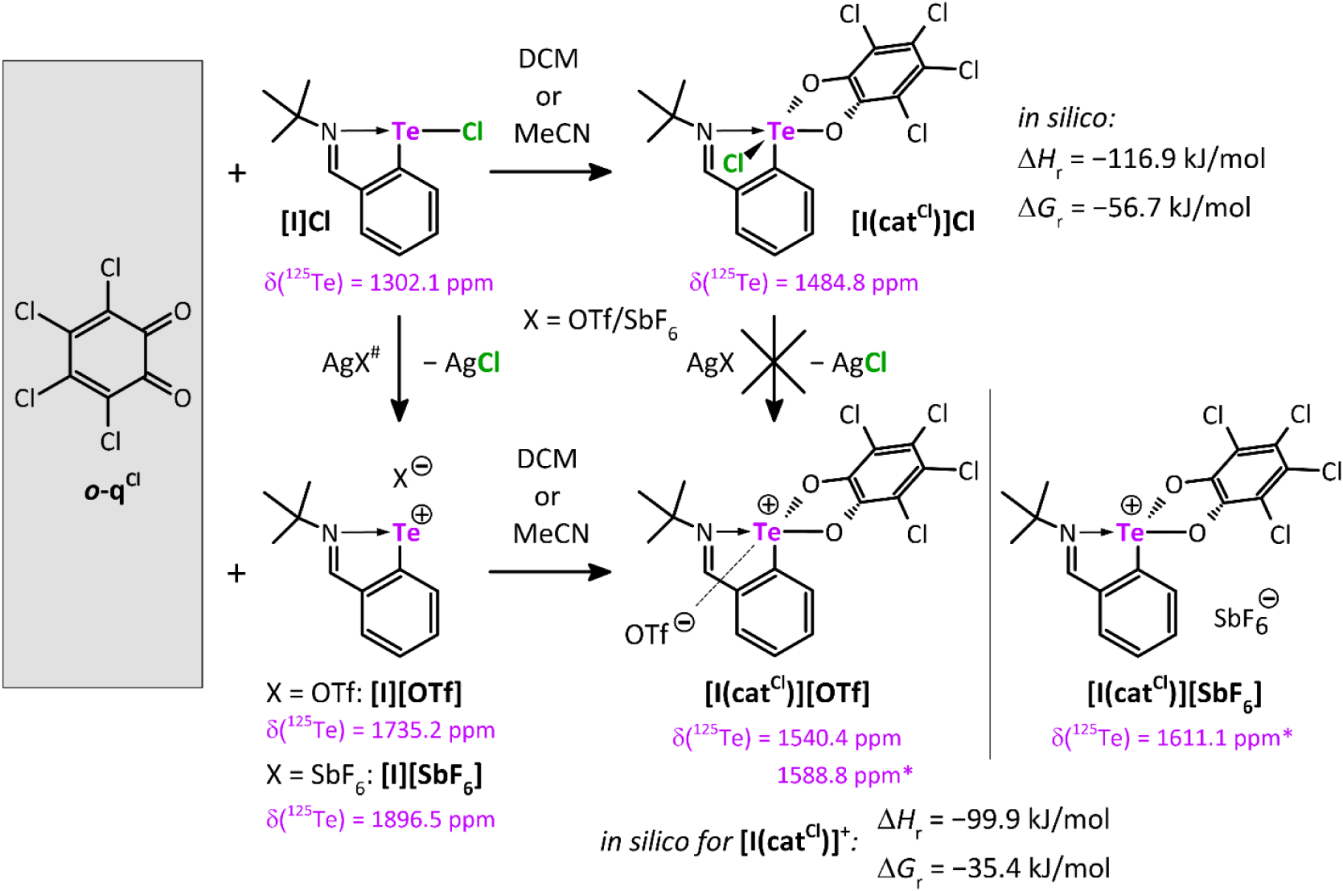
Reactivity of Tellurenyl Chloride/Triflate/Hexafluoroantimonate with ***o*** **-q** ^**Cl**^ ^a^

a Values noted by * are given in MeCN-*d*_3_, otherwise in DCM-*d*_2_. # See ref 13.

**Figure 1:**
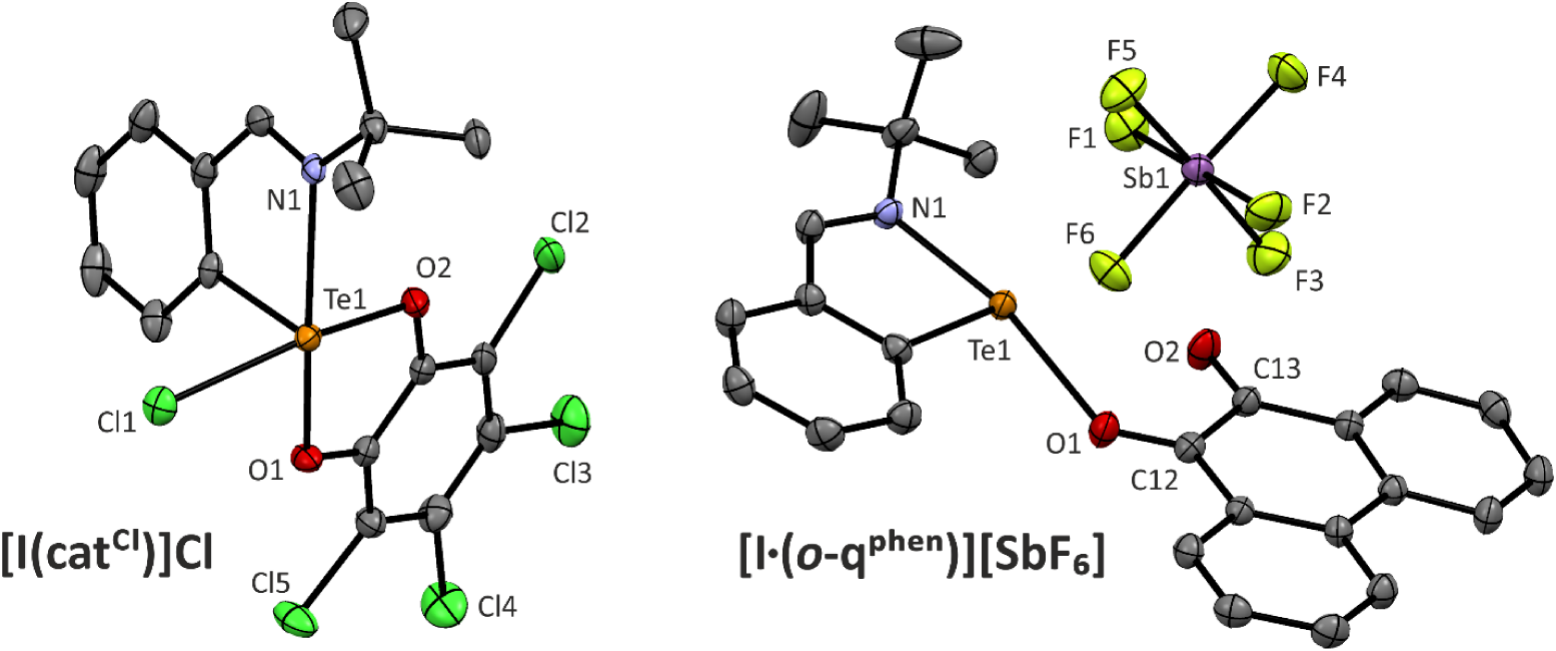
Molecular structures of Te­(IV) and Te­(II)^+^ compounds obtained by sc-X-ray diffraction. Hydrogen atoms are omitted for clarity.

Computed thermochemistry data at the B3PW91/6-311+G­(2df,p) level of theory and the inclusion of a DCM solvent environment indicate that the formation of **[I­(cat** ^**Cl**^ **)]­Cl** is exothermic (Δ*H* = −116.9 kJ mol^–1^) as well as exergonic (Δ*G* = −56.7 kJ mol^–1^). For comparison, the pure cationic **[I­(cat** ^**Cl**^ **)]** ^**+**^ tellurium­(IV) species was also investigated and leads to slightly more positive values of Δ*H* = −99.9 kJ mol^–1^ and Δ*G* = −35.4 kJ mol^–1^.

By stark contrast, the treatment of **[I]­Cl**, **[I]­[OTf],** and **[I]­[SbF**_**6**_ **]** with the more bulky and less oxidizing^16^ ***o*** **-q** ^***t*** **Bu**^ quinone resulted only in partial oxidation, giving rise to the analogous products **[I­(cat** ^***t*** **Bu**^ **)]­Cl**, **[I­(cat** ^*t* **Bu**^ **)]­[OTf],** and **[I­(cat** ^***t*** **Bu**^ **)]­[SbF**_**6**_ **]**. However, these exist in a dynamic equilibrium with the starting compounds based on ^1^H–^1^H NOESY/EXSY spectra indicating a reversible Te­(II)/Te­(IV) redox process in solution (Scheme 3). This was further supported by shifting these equilibria through changes in stoichiometry or temperature (see SI). It is also noteworthy that for the ionic species, these equilibria are established very quickly, whereas it takes two months for the neutral telluride **[I]­Cl**, indicating that kinetic factors play an important role (*vide infra*). Due to the substitution pattern of ***o*** **-q** ^***t*** **Bu**^, two regioisomers ***a*** and ***b*** are obtained, and NMR studies (see SI) proved that they interconvert mutually in solution, underlining the dynamic behavior of these systems. The ratio between both isomers deserves attention. In the case of **[I­(cat** ^***t*** **Bu**^ **)]­Cl** and **[I­(cat** ^***t*** **Bu**^ **)]­[OTf]**, both isomers are present in an unequal ratio of 0.28 (***a***):0.72 (***b***) and 0.35 (***a***):0.65 (***b***), respectively, most probably reflecting a steric clash between the *t*Bu group of **cat** ^***t*** **Bu**^ and the chlorine atom or weakly coordinated triflate moiety being located in a close vicinity, rendering the isomer ***a*** as less abundant. In the case of **[I­(cat** ^***t*** **Bu**^ **)]­[SbF**_**6**_ **]**, both isomers are obtained in a nearly equimolar ratio (0.46:0.54) being consistent with the [SbF_6_]^−^ anion located outside the tellurium coordination sphere. The ^125^Te­{^1^H} NMR spectra showed the presence of both isomers, indicated by two signals for all compounds, i.e., 1465.8/1441.5 ppm for ***a***/***b***-**[I­(cat** ^***t*** **Bu**^ **)]­Cl**, 1570.9/1535.8 ppm for ***a***/***b***-**[I­(cat** ^***t*** **Bu**^ **)]­[OTf],** and 1585.7/1556.5 ppm for ***a***/***b***-**[I­(cat** ^***t*** **Bu**^ **)]­[SbF**_**6**_ **]**. δ­(^15^N) for both ***a***/***b*** isomers are either identical or almost identical (−62.6, −80.4/–79.4, and −83.6 ppm, respectively) and are slightly deshielded compared to the Te­(IV) **cat** ^**Cl**^-complexes (−71.2, −87.7, and −88.6 ppm, respectively), but still in the range for similar Te­(IV) cationic complexes such as 2-(*t*BuNCH)­C_6_H_4_TeCl_2_OTf and 2-(*t*BuNCH)­C_6_H_4_TeCl_2_SbF_6_ (−86.8 and −87.1 ppm, respectively).^15^

**Scheme 3:**
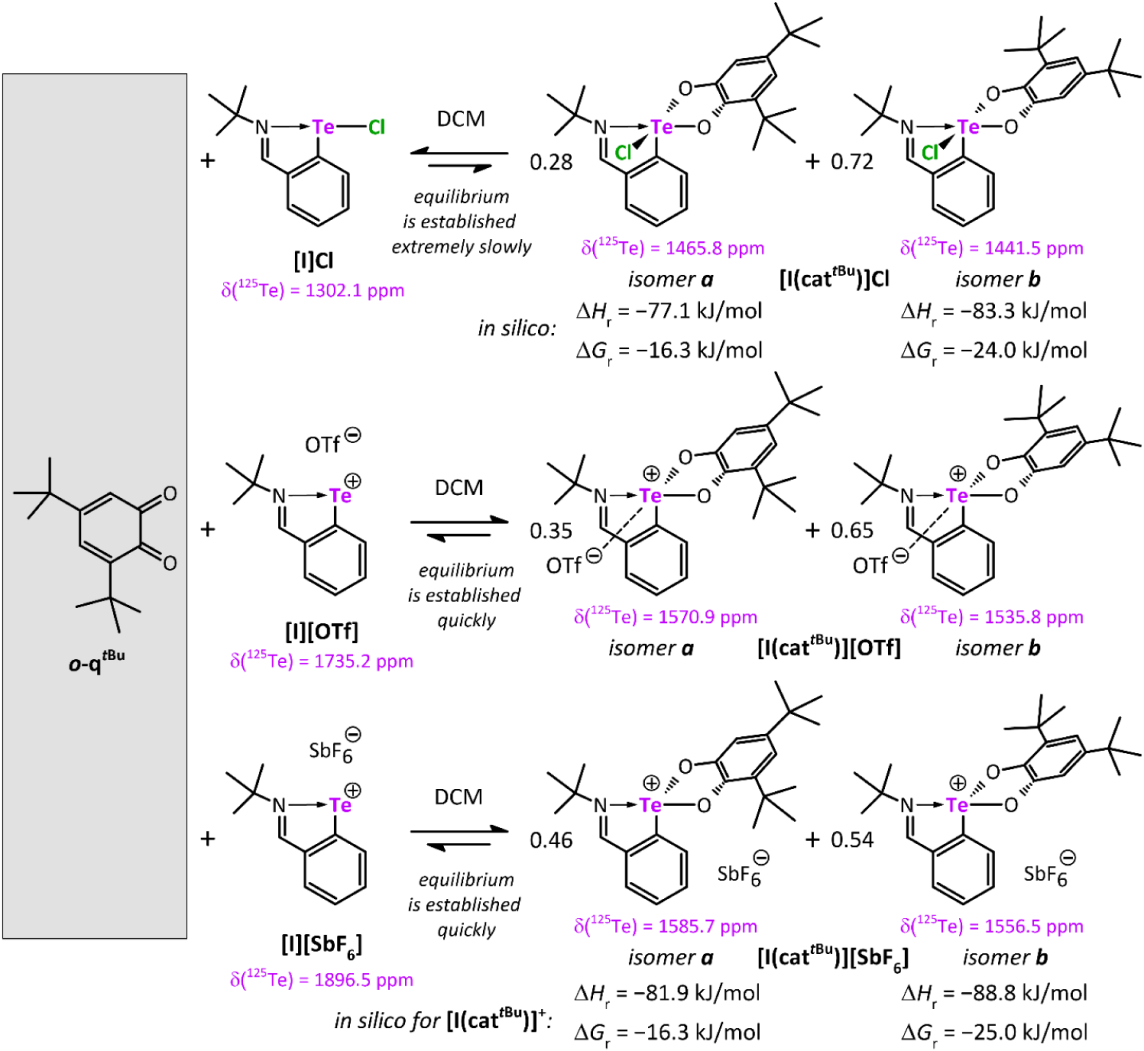
Reactivity of Tellurenyl Chloride/Triflate/Hexafluoroantimonate with ***o*** **-q** ^***t*** **Bu**^

Similar to **[I­(cat** ^**Cl**^ **)]­Cl**, the calculated thermochemistry data for **[I­(cat** ^***t*** **Bu**^ **)]­Cl** and the pure cationic **[I­(cat** ^*t* **Bu**^ **)]** ^**+**^ species indicate both exothermic and exergonic processes, with the isomer ***b*** being slightly more favored by ΔΔ*H* = 6.2 and 6.9 kJ mol^–1^ as well as ΔΔ*G* = 7.9 and 8.7 kJ mol^–1^, respectively.

To further extend the scope of this study, we examined the reactivity of ***o*** **-q** ^**phen**^ as the least oxidizing *ortho*-quinone from the studied series.^16^ This *ortho*-quinone turned out to be inert toward **[I]­Cl** and **[I]­[OTf]** based on NMR spectroscopy. However, its addition to the most Lewis acidic cation **[I]­[SbF**_**6**_ **]** in DCM-*d*_2_ resulted in an immediate color change from light orange to deep red, hinting toward an interaction. Surprisingly, the ^1^H and ^13^C­{^1^H} NMR spectra of this red solution revealed no substantial changes compared to isolated ***o*** **-q** ^**phen**^ and **[I]­[SbF**_**6**_ **]** and the chemical shifts in the ^125^Te NMR (1922.4 ppm) and ^15^N NMR spectra (−133.2 ppm) closely resemble those of **[I]­[SbF**_**6**_ **]** (1896.5 and −130.2 ppm, respectively; see SI).^13^ Fortunately, deep red single crystals crystallized out from this solution, and the molecular structure (Figure 1) revealed the formation of the Lewis adduct **[I·(** ***o*** **-q** ^**phen**^ **)]­[SbF**_**6**_ **]** (Scheme 4). Thus, although the NMR data clearly indicate the dissociation into free **[I]­[SbF**_**6**_ **]** and ***o*** **-q** ^**phen**^, being dominant in solution, the complex crystallized out due to its low solubility. In the structure of **[I·(** ***o*** **-q** ^**phen**^ **)]­[SbF**_**6**_ **]** (Figure 1), the aromatic system of the cation **[I]** ^**+**^ is almost coplanar with the phenanthrenequinone unit. A roughly linear arrangement of N1–Te1–O1 atoms (162.71°) was obtained, presumably favored by a σ-hole at the Te atom opposite to the donating N atom in the **[I]** ^+^ cation,^3b,13,17^ with the Te1–N1 bond (2.118(2) Å) being slightly elongated in comparison to the precursor **[I]­[SbF**_**6**_] (2.076(2) Å).^13^ The Te1–O1 distance (2.542(2) Å) is longer compared to that in **[I·THF]­[** ***closo*-CB**_**11**_ **H**_**12**_ **]** (2.403(1) Å),^18^ underlining the weak nature of this chalcogen-type bonding contact. The Te1–O2 distance is even longer with 2.985(2) Å (cf. ∑_cov_(Te–O) = 1.99 Å).^19^ The C12–C13 bond distance (1.537(3) Å) corresponds to a single bond along with the double bond character of the C12–O1/C13–O2 bonds (1.224(2)/1.216(3) Å), suggesting only weak σ-donation of the phenanthrenequinone to **[I]** ^**+**^, without any redox changes. This conclusion can also be deduced from FT-IR and Raman spectra in the solid state, showing no substantial changes in quinoid valence C=C and C=O bond vibrations^20^ for **[I·(** ***o*** **-q** ^**phen**^ **)]­[SbF**_**6**_ **]** (ν­(C=C) 1591 cm^–1^; ν­(C=O) 1641 cm^–^1) in comparison to free ***o*** **-q** ^**phen**^ (ν­(C=C) 1592 cm^–1^; ν­(C=O) 1673 cm^–1^).

**Scheme 4:**
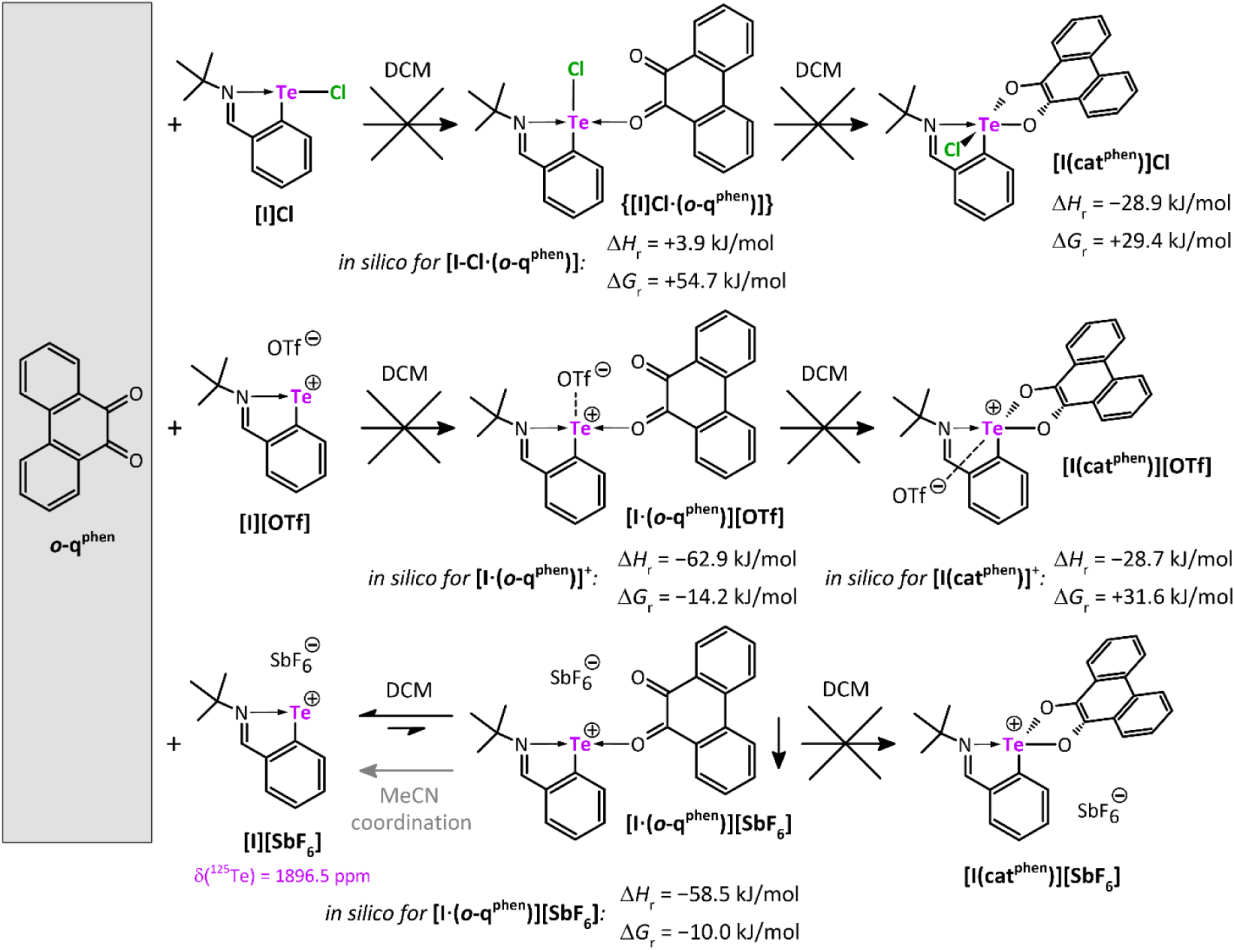
Reactivity of Tellurenyl Chloride/Triflate/Hexafluoroantimonate with ***o*** **-q** ^**phen**^

The computed thermochemistry data back up the experimental observations. Although the formation of the experimentally inaccessible tellurium­(IV) species **[I­(cat** ^**phen**^ **)]­Cl** or **[I­(cat** ^**phen**^ **)]** ^**+**^ was found to be exothermic, the Gibbs free energy proved to be endergonic (Scheme 4). The situation becomes more complex considering the formation of the Lewis adduct with ***o*** **-q** ^**phen**^ as plausible initial intermediates, i.e., **{[I]­Cl·(** ***o*** **-q** ^**phen**^ **)}**, **[I·(** ***o*** **-q** ^**phen**^ **)]­[OTf],** and **[I·(** ***o*** **-q** ^**phen**^ **)]­[SbF**_**6**_ **]**. This process was calculated to be both endothermic and endergonic for **{[I]­Cl·(** ***o*** **-q** ^**phen**^ **)}**, but exothermic and slightly exergonic for **[I·(** ***o*** **-q** ^**phen**^ **)]­[SbF**_**6**_ **]** and the pure cationic **[I·(** ***o*** **-q** ^**phen**^ **)]** ^**+**^, both in line with the experiment. As no reaction occurred for the triflate, we assume that the triflate anion is still connected more closely to the Te atom in comparison to the [SbF_6_] case, as has been reported for other tellurenyl triflates.^13,15,21^ The reluctance to form these Lewis complexes may explain why ***o*** **-q** ^**phen**^ is not able to oxidize these tellurium­(II).

The inspection of the experimentally obtained molecular structure of **[I·(** ***o*** **-q** ^**phen**^ **)]­[SbF**_**6**_ **]** and the optimized gas-phase structure (with inclusion of a DCM solvent environment) revealed substantial differences (Figure 2). Whereas the experimental geometry shows a rather coplanar arrangement of the tellurenyl **[I]­[SbF**_**6**_ **]** and ***o*** **-q** ^**phen**^ fragments, the optimized geometry shows substantial bending of both units, as exemplified by the angle between the mean planes spanned by the central (NTeC_3_) core of the **[I]­[SbF**_**6**_ **]** unit and the C_14_ core of the ***o*** **-q** ^**phen**^ unit being 171.9° for the experiment and 124.6° for the optimization. Interestingly, the geometry of the optimized pure cationic **[I·(** ***o*** **-q** ^**phen**^ **)]** ^**+**^ compares well to the experimental structure, with a plane angle of 170.7°. However, the Te–O distances of 2.537/3.023 Å (**[I·(** ***o*** **-q** ^**phen**^ **)]­[SbF**_**6**_ **]**) seem to be independent of the bending and agree fairly well with the experiment (2.542(2)/2.985(2) Å), while the distances of the cationic **[I·(** ***o*** **-q** ^**phen**^ **)]** ^**+**^ are little shorter with 2.475/2.925 Å. AIM and NCI analysis of the optimized **[I·(** ***o*** **-q** ^**phen**^ **)]­[SbF**_**6**_ **]** and **[I·(** ***o*** **-q** ^**phen**^ **)]** ^**+**^ show bond critical points between the Te-atom and both O-atoms with overall AIM parameters reminiscent of weak, predominantly ionic bonding contributions, with the shorter Te–O bond being slightly more pronounced compared to the longer bond (Table S12). The NCI analysis underlines this trend with a disk-shaped bluish area on the direct Te–O connection line indicative for attractive noncovalent interaction (chalcogen bonding).^2a^ The long Te···O bond as well as the Te···F contacts show green areas reminiscent of weak dispersive interaction. In addition, the AIM parameters found for the carbonyl and respective C–C bond differ only marginally from the parent **o-q** ^**phen**^, further indicating that the carbonyl and C–C single bond character remains intact (Table S12). It is known that chalcogen bonding between Te species and *O* donors often leads to the formation of various supramolecular organic frameworks in the solid state.^2b,22^ Interestingly, this is not the case of **[I·(** ***o*** **-q** ^**phen**^ **)]­[SbF**_**6**_ **]**.

**Figure 2:**
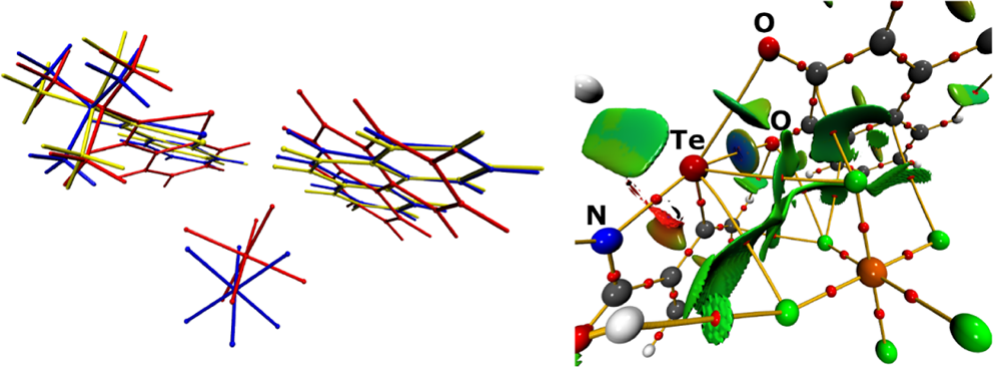
Left: Superposition of the experimentally derived structure of **[I·(** ***o*** **-q^phen^)]­[SbF_6_]**, computationally optimized structure **[I·(** ***o*** **-q^phen^)]­[SbF_6_]** (red), and **[I·(** ***o*** **-q^phen^)]^+^** (yellow). Right: AIM molecular graphs of **[I·(** ***o*** **-q^phen^)]­[SbF_6_]** with bond critical points as red spheres and bond paths in orange as well as NCI *iso*-surfaces at s­(r) = 0.5, color-coded with sign­(λ2)­ρ in a.u.

When crystals of **[I·(** ***o*** **-q** ^**phen**^ **)]­[SbF**_**6**_ **]** were dissolved in MeCN-*d*_3_, the dark red color immediately faded, and the corresponding NMR spectra proved the presence of the acetonitrile adduct **[I·(MeCN)]­[SbF**_**6**_ **]** (δ­(^125^Te) = 1718.2 ppm).^7^ This behavior indicates that the **[I·(** ***o*** **-q** ^**phen**^ **)]­[SbF**_**6**_ **]** complex is very labile in the presence of other donor(s).

Finally, to assess the importance of aromatization upon the reaction from *ortho*-quinones to the corresponding catecholates during the oxidation of the tellurium atom, **[I]­Cl**, **[I]­[OTf],** and **[I]­[SbF**_**6**_ **]** were combined with the structurally related aliphatic 1,2-cyclohexanedione (**1,2-hde**). In all cases, no reaction was observed, underlining the decisive contribution of the aromatic system formation (Scheme S1 and Figure S54 in SI). Additionally, also the computed thermochemistry data indicate endothermic and endergonic processes.

### Catalytic Mono Si–H Bond Activation

**[I]­[OTf]** was previously applied as an effective catalyst for the unprecedented Si–H bond activation and redox transfer of various silanes to *para*-quinones (Scheme 1B).^7,8^ We now extend the same catalytic strategy toward the *ortho*-quinones ***o*** **-q** ^**Cl**^, ***o*** **-q** *^**t**^* **^Bu^,** and ***o*** **-q** ^**phen**^.

The catalyzed reaction between Et_3_SiH and ***o*** **-q** ^**Cl**^ or ***o*** **-q** ^***t*** **Bu**^ at RT using 1 mol % of **[I]­[OTf]** (acetonitrile, 12 h or 10 min, respectively; Scheme 5) provided the expected silylated catechols **1** and **2**, respectively.^23^ By contrast, the analogous reaction using ***o*** **-q** ^**phen**^ provided a mixture not only of the expected catechol **3** (major product, 80%) but also of the double-silylated byproduct **3′**, while the mechanism of the formation of the latter remains unclear. Importantly, the mutual molar ratio between **3** and **3′** turned out to be almost independent of the reaction conditions (see SI).

**Scheme 5:**
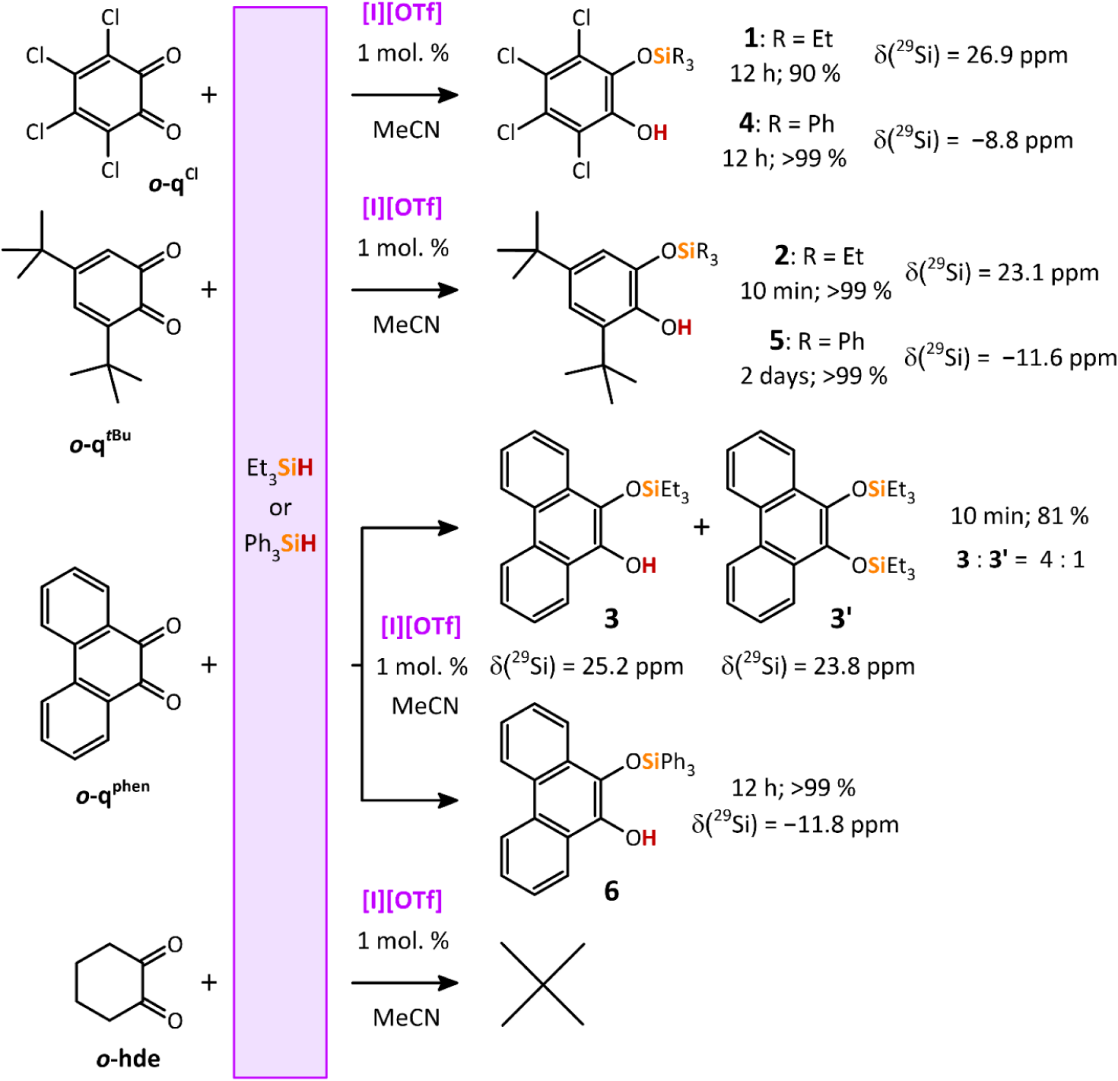
Redox Catalytic Activation of Si–H Bond in Et_3_SiH Using Tellurenyl Triflate **[I]­[OTf]** in MeCN at Room Temperature^a^

a Conversion rates of reactions are based on ^1^H NMR integration related to the starting *ortho*-quinone; isolated yields are given in Experimental Section.

The identity of **1**-**3′** was proven by ^1^H, ^13^C, and ^29^Si NMR spectra, the latter revealing one resonance at 26.9/23.1/25.2/23.8 ppm for **1**/**2**/**3**/**3′**, respectively.

To shed some light on the mechanism of the catalysis, we examined the reaction leading to the silylated catechol **2** by performing stoichiometric reactions on all three components of the reaction, i.e., Et_3_SiH, **[I]­[OTf],** and ***o*** **-q** ^***t*** **Bu**^. The results suggest that the overall reaction mechanism of the catalysis is analogous to the previously proposed mechanism for the catalysis using *para*-quinones.^8^ However, as the Te­(IV) species ***a***/***b***-**[I­(cat** ^*t* **Bu**^ **)]­[OTf]** is formed reversibly, it can serve as a temporary “storage buffer” for both the catalyst **[I]­[OTf]** and quinone ***o*** **-q** ^***t*** **Bu**^ (see Chapter *Mechanistic study of the catalysis* in SI with Schemes S2–S6 for details).

Expanding the reactivity studies toward Ph_3_SiH, the silylated catechols **4**–**6** were produced in high isolated yields. Notably, no byproduct analogous to **3′** is formed in the reaction with ***o*** **-q** ^**phen**^ (Scheme 5). The ^29^Si NMR spectra revealed resonances at −8.8/–11.6/–11.8 ppm for **4**/**5/6**, respectively, and the molecular structures of **5** and **6** are depicted in Figure 3.

**Figure 3:**
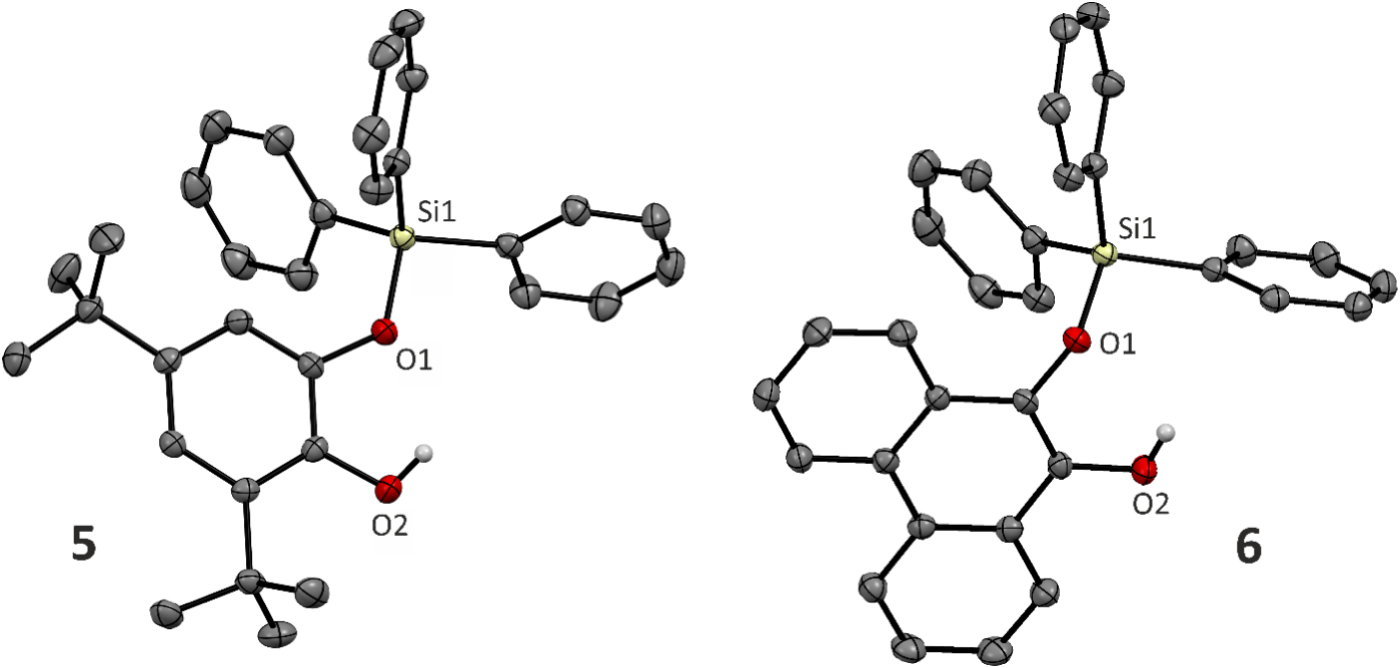
Molecular structures of products of catalysis obtained by sc-X-ray diffraction. Hydrogen atoms are omitted for clarity, except OH protons.

The analogous reactions between Et_3_SiH and ***o*** **-q** ^***t*** **Bu**^ in MeCN in the absence of **[I]­[OTf]** showed no reaction even after prolonged heating (80 °C), whereas in the case of ***o*** **-q^phen^**, the silylated catechol **3** is formed at RT, but the reaction is very slow (conversion less than 3% after 1 month). Interestingly, the 1:1 stoichiometric reaction between Et_3_SiH and ***o*** **-q** ^**Cl**^ at RT did not provide the silylated catechol **1** but gave rise to the hexachloro-dibenzo­[1,4]­dioxine-2,3-dione **7**, along with catechol, Et_3_SiCl, and unreacted Et_3_SiH as byproducts. Setting the stoichiometry of Et_3_SiH: ***o*** **-q** ^**Cl**^ to 3:2 made this reaction almost quantitative after 7 days (Scheme 6; for molecular structure, see Figure 4), which proves that this reaction follows an entirely different reaction mechanism than the catalysis using **[I]­[OTf]**.

**Scheme 6:**
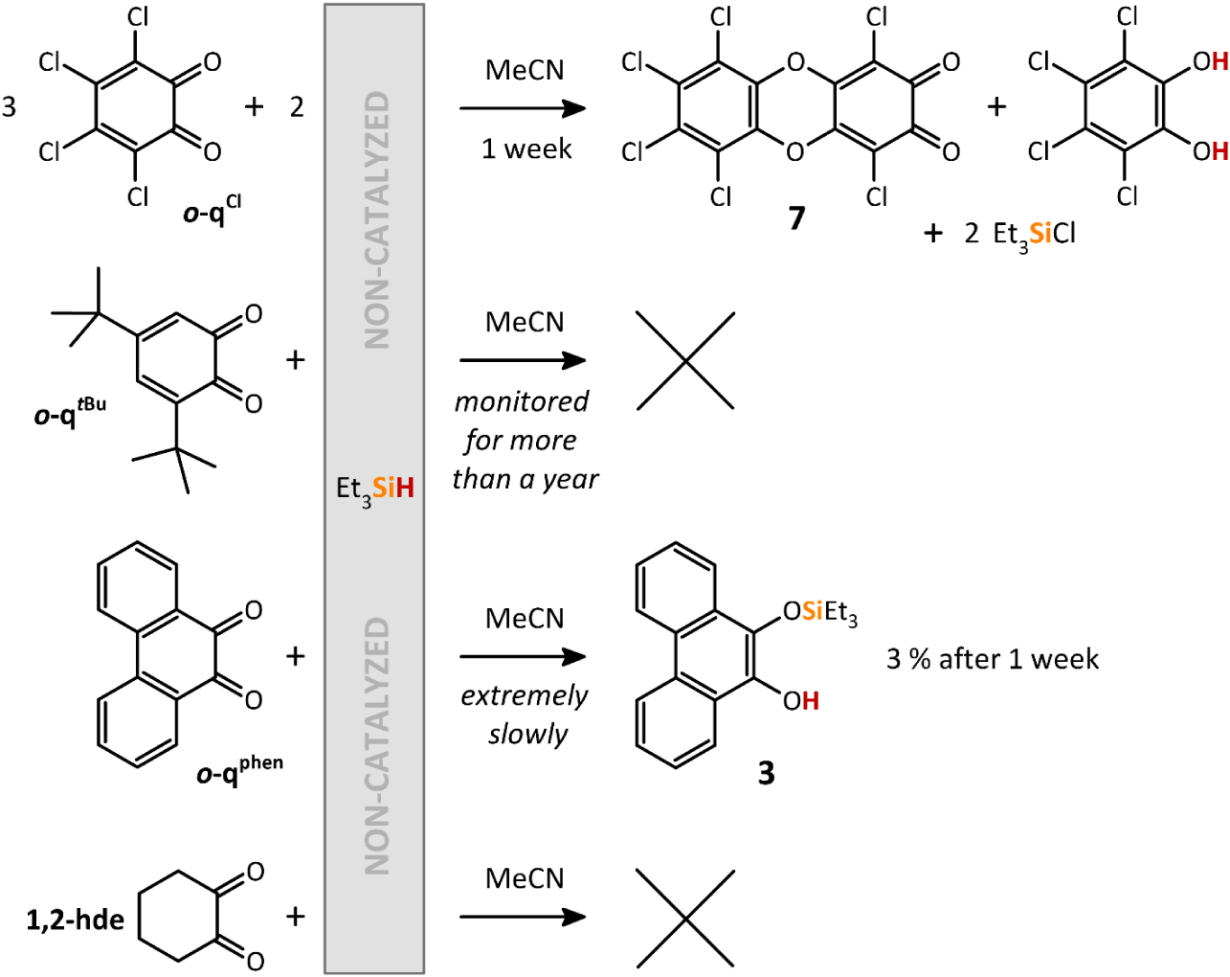
Non-Catalyzed Reactions of Various *ortho*-Quinones and **1,2-hde** with Et_3_SiH in MeCN at Room Temperature

**Figure 4:**
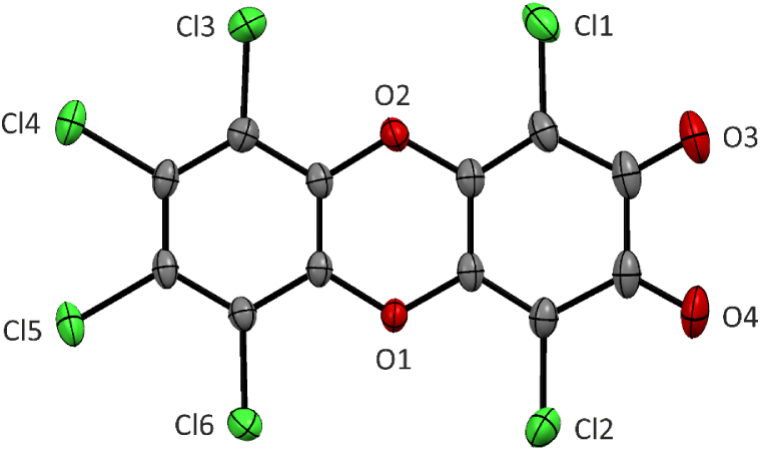
Molecular structure of dioxin **7** obtained by sc-X-ray diffraction. Cocrystallized molecules of benzene are omitted for clarity.

It is to note that synthesis of **7** was previously accomplished by the oxidative coupling of ***o*** **-q** ^**Cl**^ with the corresponding catechol^24^ by treatment of ***o*** **-q** ^**Cl**^ with CuCl_2_ in the presence of elemental copper,^25^ Co_2_(CO)_8_, or elemental magnesium^26^ and was recently discussed as a possible intermediate in the formation of other polychlorinated compounds.^27^

### Catalytic Double Si–H Bond Activation

Further, we focused on the catalysis using Ph_2_SiH_2_, possessing two geminal Si–H bonds, with two equivalents of ***o*** **-q** ^**Cl**^, ***o*** **-q** *^**t**^* **^Bu^**, or ***o*** **-q** ^**phen**^ under the same catalytic conditions. These reactions furnished a set of cyclic catecholatosilanes **8**, **9**, and **10**, along with the corresponding catechols in a 1:1 molar ratio, and could be easily isolated from the reaction mixtures in very high yields of 71–94% owing to their low solubility in MeCN (Scheme 7, details in Experimental Section).

**Scheme 7:**
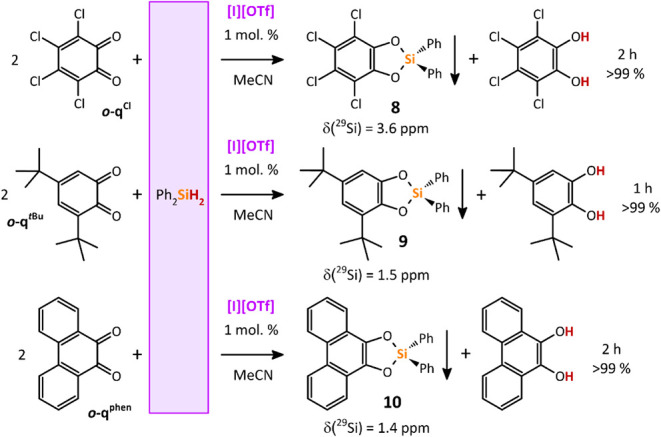
Redox Catalytic Activation of Both Geminal Si–H Bonds in Ph_2_SiH_2_ Using Tellurenyl Triflate **[I]­[OTf]** in MeCN at Room Temperature^a^

a Conversion rates of reactions are based on ^1^H NMR integration related to the starting *ortho*-quinone; isolated yields are given in Experimental Section.

The stepwise activation of both Si–H bonds in Ph_2_SiH_2_ was proven by an equimolar reaction between Ph_2_SiH_2_ and ***o*** **-q** ^***t*** **Bu**^, leading to a mixture of **9**, noncyclic silylated-catechol **9a**, catechol, and unreacted Ph_2_SiH_2_ in a mutual ratio of 0.55:0.23:0.22:0.22. The Si–H bond in **9a** revealed a doublet at δ­(^29^Si) = −11.7 ppm (^1^ *J*(^29^Si,^1^H) = 222 Hz) in the corresponding ^29^Si NMR spectra. Importantly, the addition of a second equivalent of ***o*** **-q** ^***t*** **Bu**^ led to the activation of the remaining Si–H bond in **9a,** most probably giving an elusive **9b**, that transforms to a 1:1 mixture of **9** (precipitating from the reaction mixture) and catechol (Scheme 8).

**Scheme 8:**
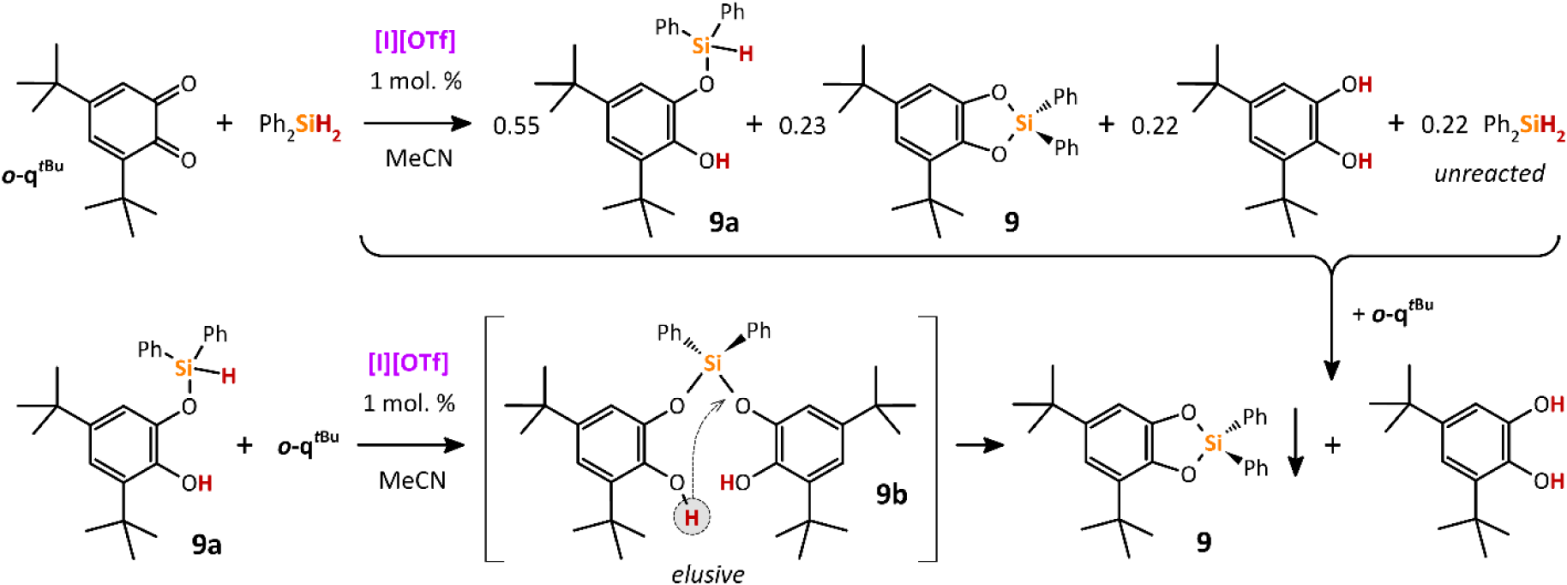
Result of **[I]­[OTf]**-Catalyzed Reaction of ***o*** **-q** ^***t*** **Bu**^ with Ph_2_SiH_2_ in 1:1 Molar (Top Part) and after Addition of Another Equiv. of ***o*** **-q** ^***t*** **Bu**^ (Vertical Down Arrow), along with Supposed Reactivity of the Key Intermediate **9a** with ***o*** **-q** ^***t*** **Bu**^ at the Same Conditions (Bottom Part)

Compounds **8**, **9**, and **10** featuring a Ph_2_SiO_2_ fragment are not the first representatives of this class of siloxanes; however, their monomeric structure is unique, as other known members of this class form dimeric structures with 10-membered C_4_O_4_Si_2_ rings. A dynamic equilibrium between the monomeric and dimeric forms was proposed in solution for the siloxane with a Me_2_SiO_2_ fragment,^28^ while the structurally most related catecholatosilane Ph_2_SiO_2_C_6_H_4_ is monomeric only at elevated temperatures (>59 °C).^29^ By contrast, **8**, **9**, and **10** retain their monomeric nature in solution, showing unusual chemical shifts of δ­(^29^Si) = 3.6/1.5/1.4 ppm, respectively, deshielded by ca. 36 ppm in comparison to noncyclic siloxanes, such as Ph_2_Si­(O-2,6-(*t*Bu)_2_C_6_H_2_OH)_2_ (−38.0 ppm). Interestingly, these values match very well with the chemical shift of −1.37 ppm^29a^ proposed for the monomeric Ph_2_SiO_2_C_6_H_4_.

The molecular structures of **8** and **9** (Figure 5) revealed that the 5-membered C_2_O_2_Si rings are strained, and all Si–O bonds (range 1.673(1)–1.698(1) Å) are elongated by ca. 0.01–0.03 Å in comparison to noncyclic **5** and **6** (cf. 1.6647(7) and 1.674(1) Å, respectively). The ring strain is also reflected by the deformation of O1–Si1–O2 bond angles to 94.84° and 96.34° for **8** and **9**, respectively. Nevertheless, these structural parameters are similar to other species with 5-membered C_2_O_2_Si rings.^30^

**Figure 5:**
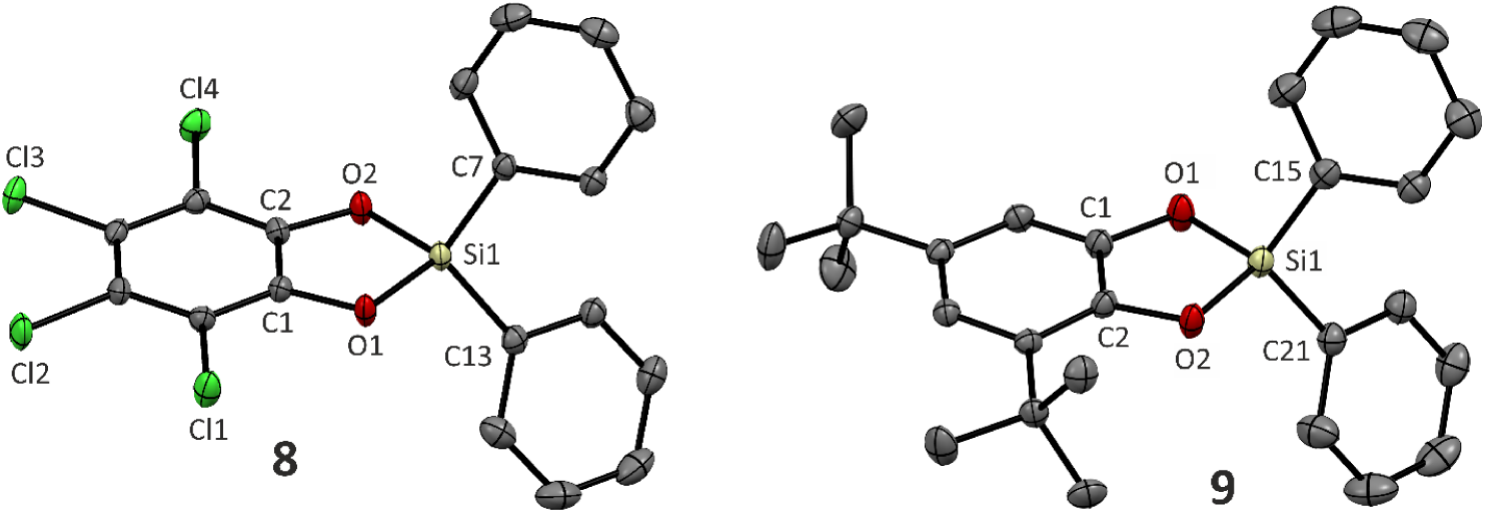
Molecular structures of products of catalysis obtained by sc-X-ray diffraction. Hydrogen atoms are omitted for clarity, except OH protons.

Compounds **8**, **9**, and **10** easily hydrolyze to disiloxanes **11**–**13** upon the opening of the 5-membered C_2_O_2_Si ring (Scheme 9). The ^1^H, ^13^C NMR spectra of the siloxanes **11**–**13** showed the expected set of signals, including the O*H* function, with δ­(^1^H) being in the range of 4.78–5.97 ppm. ^29^Si NMR spectra contained resonances at −30.4/–37.6/–37.8 ppm for **11**/**12/13**, respectively, i.e., with typical values for Ph_2_SiO_2_ fragments and similar to reported values for (C_6_H_4_(OH)­OSiPh_2_)_2_O (δ­(^29^Si) = −37.98 ppm).^29b^ The molecular structures of **12** and **13** are shown in Figure 6. All Si–O bonds are in the range of 1.6233(9)–1.6510(9) Å in **12** (1.6206–1.653(1) Å in **13**) and are shorter compared to **9** (1.673(1) and 1.6783(9) Å), reflecting a more unstrained bonding situation compared to the parent compounds.

**Scheme 9:**
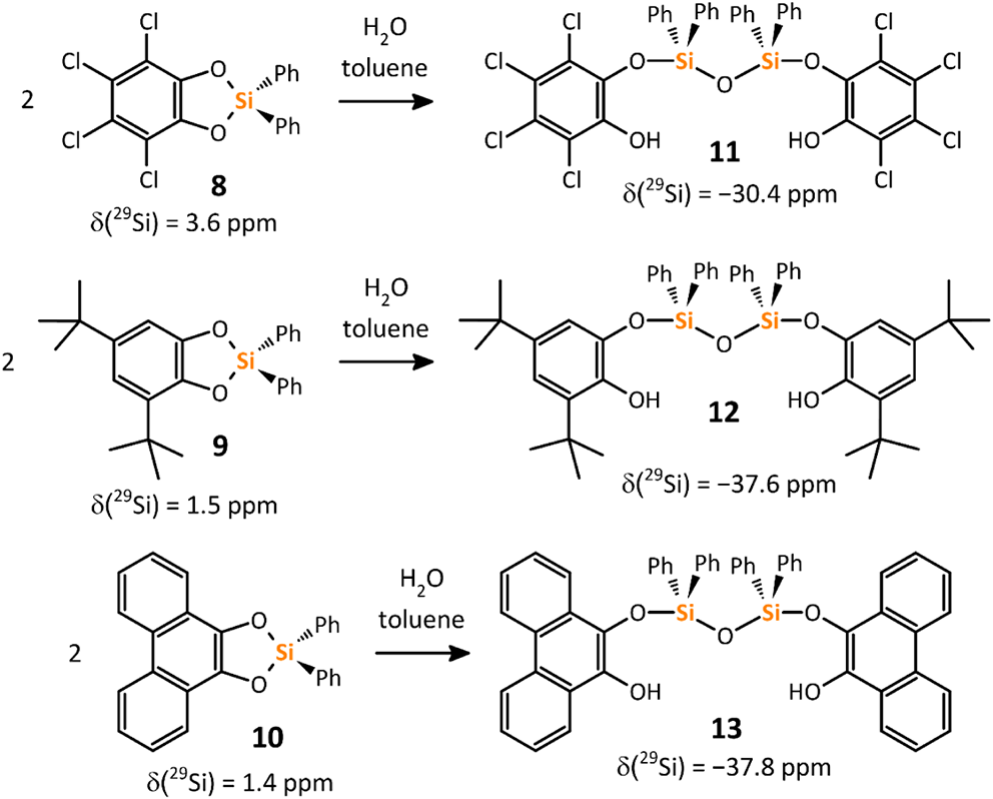
Result of Intended Hydrolysis of Cyclic Catecholatosilanes **8**–**10**

**Figure 6:**
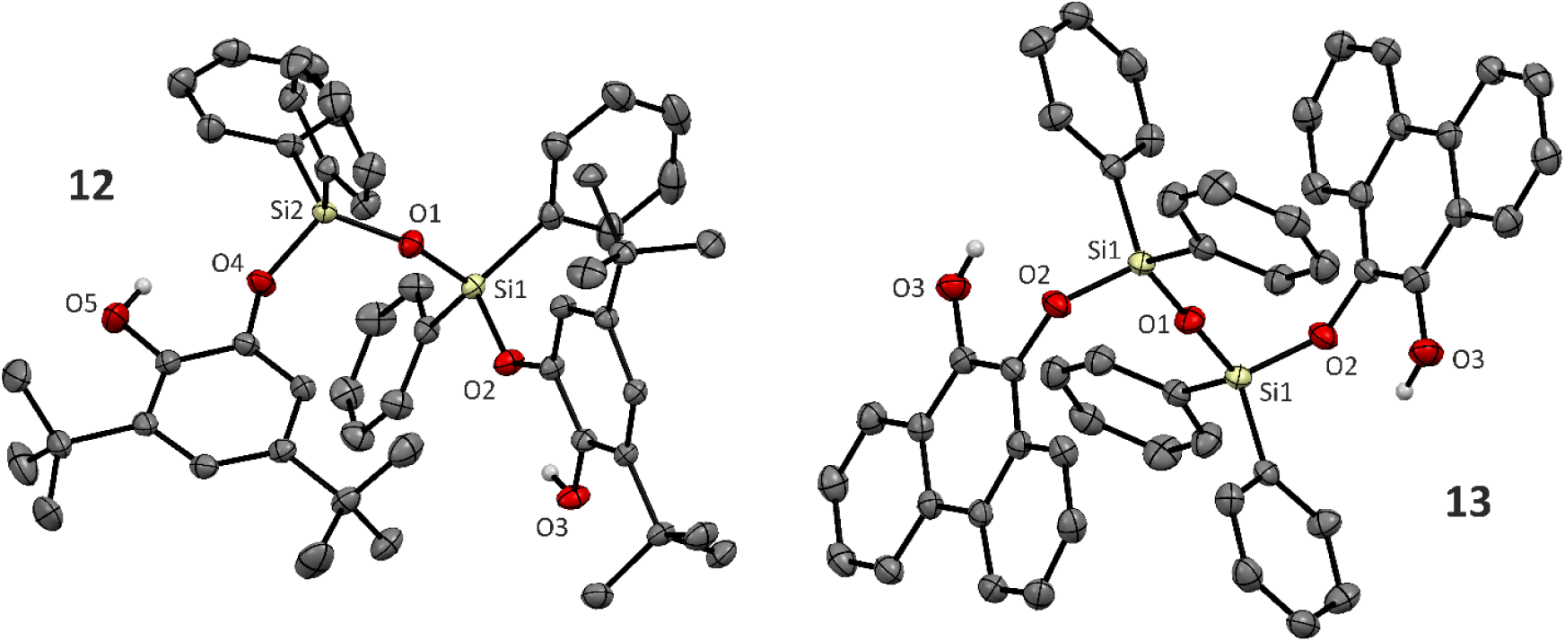
Molecular structures of disiloxanes **12** and **13** being products of hydrolysis of cyclic catecholatosilanes **9** and **10**, respectively, obtained by sc-X-ray diffraction. Hydrogen atoms are omitted for clarity, except OH protons.

Surprisingly, the reaction of Ph_2_SiH_2_ with ***o*** **-q** ^**Cl**^ in the absence of **[I]­[OTf]** as a catalyst led to the precipitation of the cyclic catecholatosilane **8** within 5 days in moderate yield (Scheme 10). In contrast, ***o*** **-q** ^***t*** **Bu**^ showed no conversion even after prolonged heating at 80 °C, whereas ***o*** **-q** ^**phen**^ formed the cyclic catecholatosilane **10** (Scheme 10), but only with extremely low conversion. It is important to note that compared to the catalyzed reactions shown in Scheme 7, these noncatalyzed reactions leading to **8** and **10** did not provide any corresponding catechol as a byproduct. This indicates a completely different mechanism, such as a spontaneous hydrosilylation of the C=O bond, followed by the elimination of dihydrogen upon enclosure of the 5-membered C_2_O_2_Si ring. Such a reaction mechanism is proposed for the reaction between *ortho*-quinones and BH_3_.^31^

**Scheme 10:**
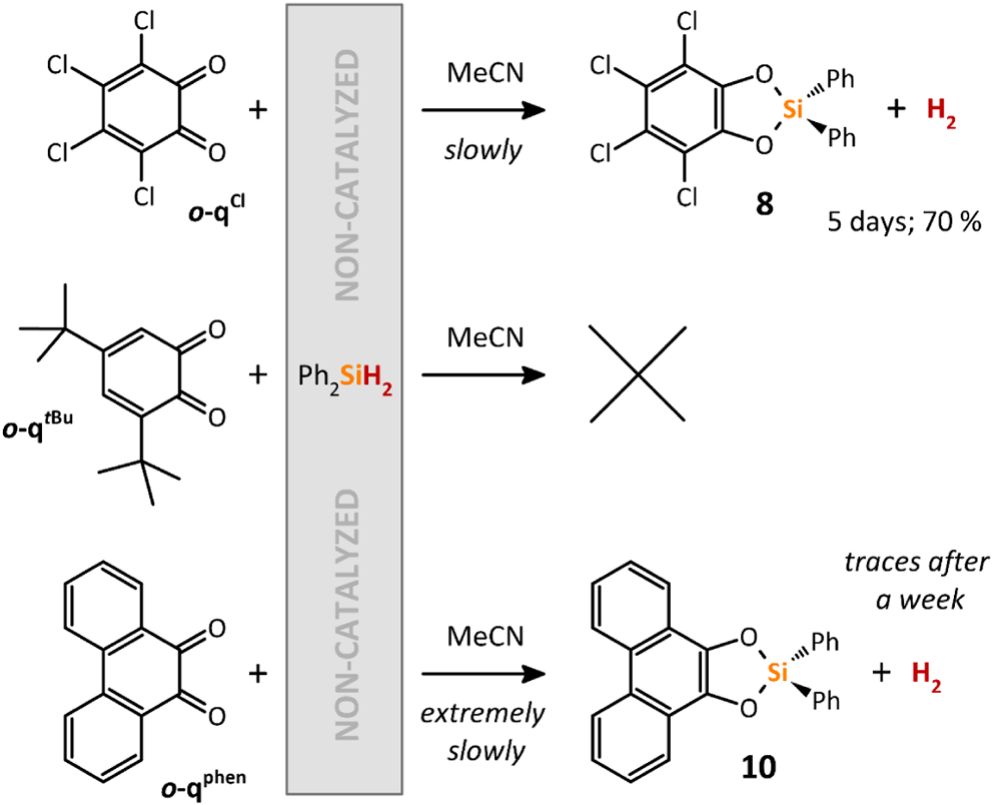
Non-Catalyzed Reactions of Various *ortho*-Quinones with Ph_2_SiH_2_ in MeCN at Room Temperature

### Catalytic Si–H Bond Activation of Trialkoxysilanes

Finally, the catalyzed reactions between (EtO)_3_SiH and ***o*** **-q** ^**Cl**^, ***o*** **-q** ***^t^*** **^Bu^**, or ***o*** **-q** ^**phen**^ were examined (Scheme 11). The reaction with ***o*** **-q** ^**Cl**^ resulted in the precipitation of an insoluble material overnight. This solid was tentatively assigned as the EtOH adduct of the bis­(catecholato)­silane **14**, i.e., [**14**·(EtOH)], *vide infra*. Importantly, the recrystallization of this solid from boiling dmso provided the hexacoordinated dmso bis-adduct of bis­(catecholato)­silane, i.e., [**14**·(dmso)_2_] (see SI). [**14**·(dmso)_2_] showed a typical highly shielded chemical shift at −138.1 ppm in the ^29^Si NMR spectrum, similar to the value of −150.3 ppm for solid [**14**·(MeCN)_2_] determined by ^29^Si MAS NMR spectroscopy.^9e^

**Scheme 11:**
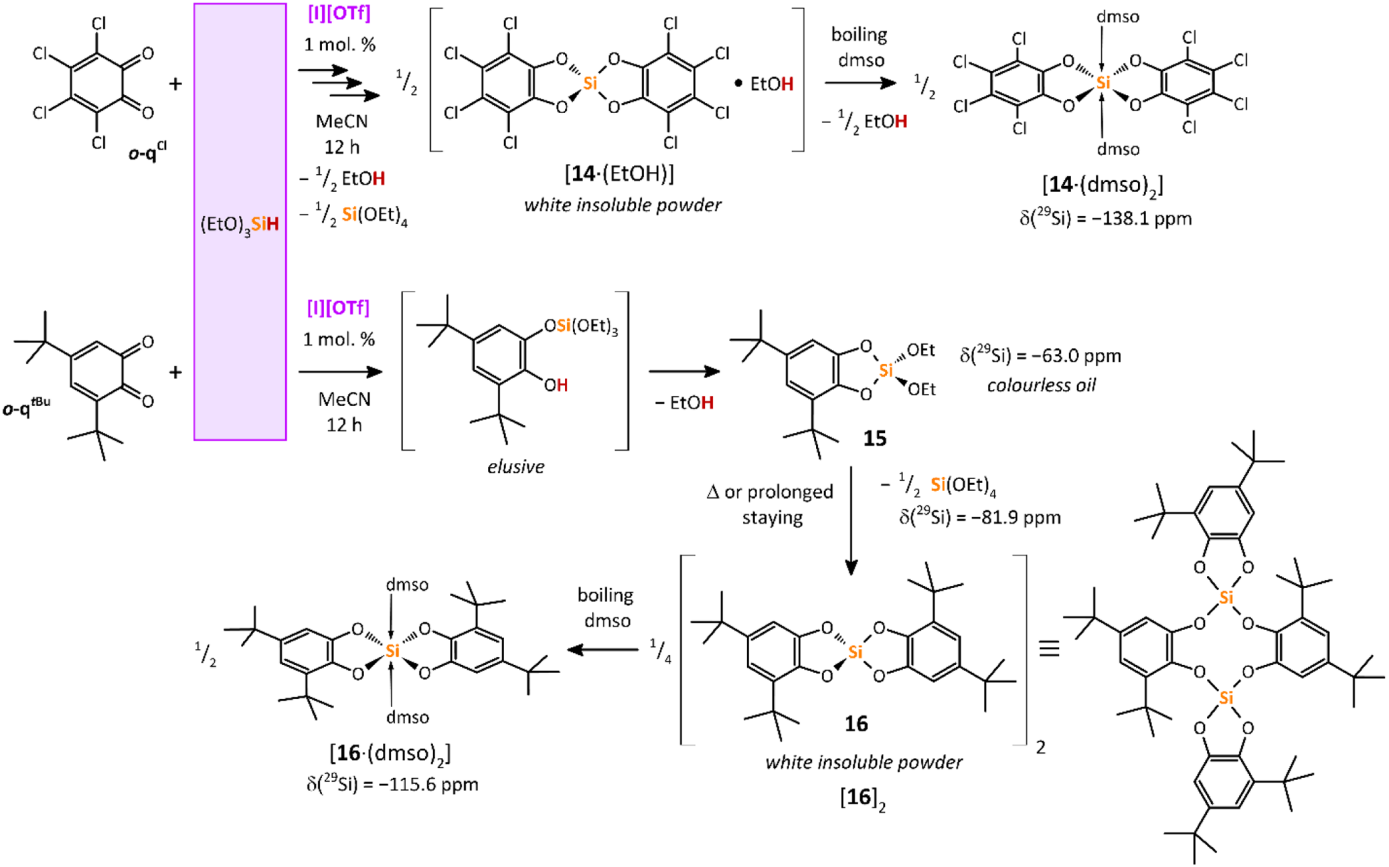
Redox Catalytic Activation of Si–H Bond in (EtO)_3_SiH Using Tellurenyl Triflate **[I]­[OTf]** in MeCN at Room Temperature with Multiple Follow-Up Reactions Ultimately Leading to Bis­(catecholato)­silanes

Importantly, the same catalyzed reaction with ***o*** **-q** ^***t*** **Bu**^ provided the oily cyclic catecholatosilane **15** as a result of Si–H bond activation and elimination of 1 eq. of ethanol (Scheme 11). The ^1^H, ^13^C NMR spectra are consistent with the proposed structure, while the ^29^Si NMR spectrum showed one signal at −63.0 ppm. Prolonged staying (or heating at 100 °C for 1 h) of **15** resulted in the formation of an insoluble white material, while (EtO)_4_Si formed as a byproduct, as judged by the ^29^Si NMR spectrum containing the characteristic signal at −81.9 ppm. Surprisingly, recrystallization of the insoluble material from dry boiling dmso furnished single crystals of the hexacoordinated bis­(catecholato)­silane [**16**·(dmso)_2_] (Figure 7). This finding suggests that compound **15** decomposed in solution to (EtO)_4_Si and the dimeric **16**, i.e., [**16**]_2_,^9e^ which upon recrystallization provided the monomeric [**16**·(dmso)_2_]. The ^1^H, ^13^C NMR spectra of [**16**·(dmso)_2_] showed the expected set of signals corresponding to a centrosymmetric molecule similarly as obtained in the solid state (*vide infra*). The ^29^Si NMR spectrum contained a signal at −115.6 ppm, resembling the highly shielded values for other known hexacoordinated bis­(catecholato)­silanes.^9e^ We propose that the initial steps in the tandem reaction sequence for the formation of [**16**·(dmso)_2_] go through a cyclic catecholatosilane **15**, which intermolecularly reacts with another molecule of **15** under formation of **16** and (EtO)_4_Si, which might also be a plausible mechanism leading to [**14**·(dmso)_2_].

**Figure 7:**
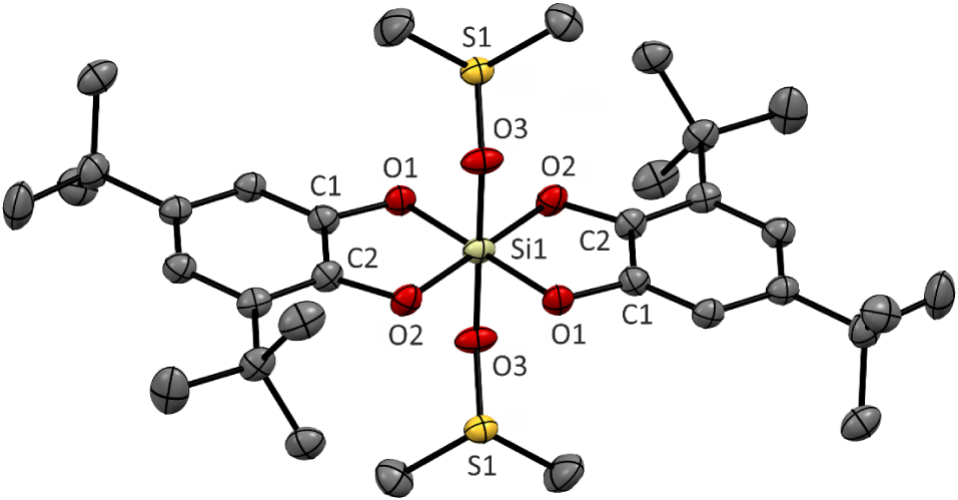
Molecular structures of bis­(catecholato)­silane [**16**·(dmso)_2_] obtained by sc-X-ray diffraction, showing only one of two independent molecules. Hydrogen atoms and two molecules of dmso are omitted for clarity.

Two independent (but structurally similar) centrosymmetric molecules with hexacoordinated silicon atoms were obtained in the structure of [**16**·(dmso)_2_]. The catecholato units and the Si1 atom form an equatorial plane of a tetragonal bipyramid, with Si1–O1 1.750 Å and Si1–O2 1.738 Å bond lengths. The axial positions are occupied by dmso molecules, with the Si1–O3 (1.889 Å) bonds being slightly elongated compared to the equatorial Si–O bonds, but all values compare well with other bis­(catecholato)­silanes containing hexacoordinated Si atoms.^9b–9d^

Unfortunately, all attempts to perform a similar catalyzed reaction between ***o*** **-q** ^**phen**^ and (EtO)_3_SiH, potentially yielding bis­(catecholato)­silane derived from phenanthrenequinone, remained unsuccessful, as we always obtained a complicated reaction mixture.

Surprisingly, the treatment of ***o*** **-q** ^**Cl**^ with (EtO)_3_SiH in MeCN without the catalyst **[I]­[OTf]** also led to a white precipitate, which, after recrystallization, again furnished [**14**·(dmso)_2_]. Despite this, the noncatalyzed reaction takes 14 days; it is an efficient alternative synthetic route toward bis­(catecholato)­silanes, which are usually prepared by the conversion of catechols with HSiCl_3_.^9b,9e^ On the contrary, no reaction was observed in the cases of ***o*** **-q** ^***t*** **Bu**^ and ***o*** **-q** ^**phen**^ with (EtO)_3_SiH in MeCN at noncatalyzed conditions (Scheme 12).

**Scheme 12:**
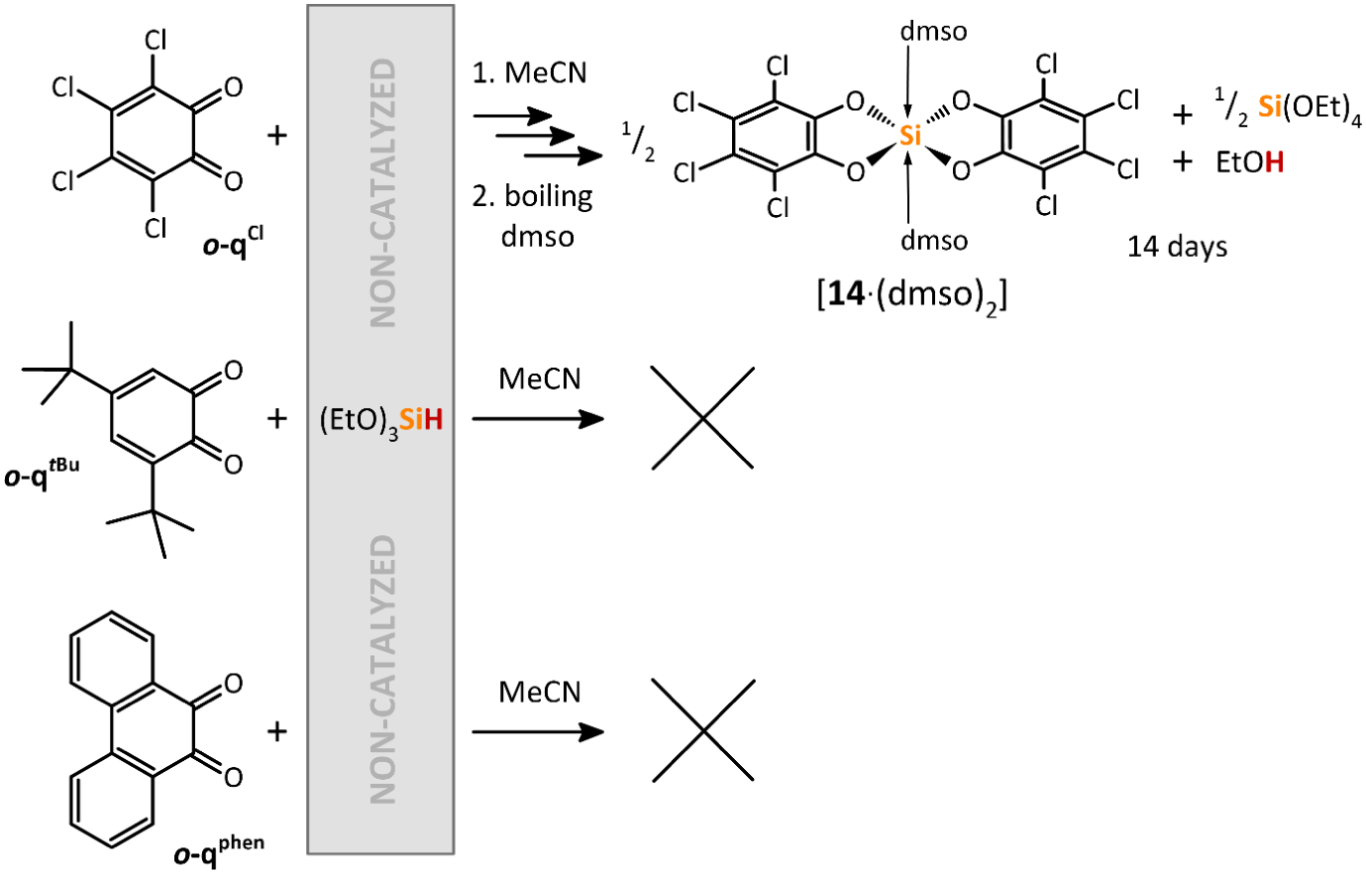
Non-Catalyzed Reactions of Selected *ortho*-Quinones with (EtO)_3_SiH in MeCN

## Conclusions

We report the first chemically reversible, two-electron Te­(II)/Te­(IV) redox process observed during the oxidation of tellurium­(II) compounds and cations by selected *ortho*-quinones. This process is influenced by both the relative Lewis acidity of the tellurium compounds and the oxidizing ability of the respective *ortho*-quinone, as corroborated by DFT-computed thermochemistry data.

Furthermore, the tellurenyl triflate **[I]­[OTf]** was successfully utilized for the redox transfer of various silanes to *ortho*-quinones, resulting in the formation of silylated catechols, monomeric cyclic catecholatosilanes, or bis­(catecholato)­silanes. A mechanistic study of the reaction between *ortho*-quinone ***o*** **-q** ^***t*** **Bu**^ and Et_3_SiH suggests a catalytic cycle analogous to that previously proposed for related reactions involving *para*-quinones.^8^ However, while catalysis with *para*-quinones^8^ proceeds via tellurium species in the +I and +II oxidation states, the current system also involves the +IV oxidation state.

By coincidence, this work also represents a novel synthetic pathway to bis­(catecholato)­silanes, which are usually prepared by the reaction of catechol with HSiCl_3_ ^9b,9e^ and are of interest to the silicon community in view of their high Lewis acidity and catalytic activity. Notably, the specific reactivity of *ortho*-chloranil (***o*** **-q** ^**Cl**^) with (EtO)_3_SiH produces bis­(catecholato)­silane [**14**·(dmso)_2_] directly without a catalyst.

## Experimental Section

### Hazards

**Caution!** Redox catalysis using highly reactive silanes and *ortho*-quinones may potentially lead to a thermal runaway reaction if carried out on a larger scale or at higher reagent concentrations than described in the synthetic protocols shown below for each compound.

**Caution!** Potential extreme toxicity hazard! Although no toxicity data are available for compound **7** (hexachloro-dibenzo­[1,4]­dioxine-2,3-dione), a dioxine-like compound, it should be handled with the utmost caution to avoid skin contact or inhalation of dust, due to the potential for biotransformation into highly toxic polychlorinated dioxins.

### General Procedures

All air- and moisture-sensitive manipulations were performed under argon (99.9996%) using standard Schlenk techniques with rubber septa and cannula transfer for liquid handling and filtration. All glassware was flame-annealed in vacuum prior to use. All solvents for reactions were dried using Pure Solv–Innovative Technology equipment. The starting compounds: *ortho*-chloranil (***o*** **-q** ^**Cl**^) (97%), 3,5-di-*tert*-butyl-*ortho*-benzoquinone (***o*** **-q** ^***t*** **Bu**^) (98%), 9,10-phenanthrenequinone (***o*** **-q** ^**phen**^) (>99%), 1,2-cyclohexanedione (**1,2-hde**) (97%), Et_3_SiH (99%), Ph_3_SiH (97%), Ph_2_SiH_2_ (97%), (EtO)_3_SiH (95%) were purchased from Merck. Compounds **[I]­Cl**, **[I]­[OTf],** and **[I]­[SbF**_**6**_ **]** were synthesized according to our recently published procedures.^13,14^

### Solution NMR Spectroscopy

^1^H, ^13^C, ^15^N, ^29^Si, and ^125^Te NMR spectra were recorded on a Bruker Avance 500 MHz spectrometer, using a 5 mm tunable broadband probe. Appropriate chemical shifts in ^1^H and ^13^C NMR spectra are given relative to the residual signals of the solvent [DCM-*d*_2_: δ­(^1^H) = 5.32 ppm and δ­(^13^C) = 54.0 ppm; MeCN-*d*_3_: δ­(^1^H) = 1.94 ppm and δ­(^13^C) = 118.69 ppm; benzene-*d*_6_: δ­(^1^H) = 7.16 ppm and δ­(^13^C) = 128.39 ppm]; ^15^N NMR spectra were related to external neat nitromethane [δ­(^15^N) = 0.0 ppm]; ^19^F NMR spectra were related to external neat CFCl_3_ [δ­(^19^F) = 0.0 ppm]; ^29^Si NMR spectra were related to dilute TMS in CDCl_3_ [δ­(^29^Si) = 0.0 ppm]; and ^125^Te NMR chemical shifts were referenced to an external CDCl_3_ solution of Ph_2_Te_2_ [δ­(^125^Te) = 422 ppm relative to Me_2_Te]. NMR solvents (DCM-*d*_2_ (99.96% D), MeCN-*d*_3_ (99.80% D), and C_6_D_6_ (99.6% D)) were dried by storing over activated molecular sieves (3Å, 20 wt%) for a couple of days and then degassed by three cycles *freeze–pump–thaw.* NMR samples were prepared under argon and measured in annealed flame-sealed NMR tubes. The full assignment of all signals in the measured NMR spectra was managed with the help of various techniques, including ^1^H, ^1^H–^1^H COSY, ^1^H–^1^H NOESY, ^13^C­{^1^H} APT, ^1^H–^13^C HSQC, and ^1^H–^13^C HMBC. ^15^N NMR chemical shifts and ^1^ *J*(^15^N,^1^H) were obtained from ^1^H–^15^N HMBC spectra (value of cnst13 = 5 Hz).

### IR and Raman Spectroscopy

FT-IR spectra of solid samples were recorded using a single-bounce diamond ATR crystal on Nicolet iS50. FT-Raman spectra of solid samples or liquids were recorded in the range 4000–100 cm^–1^ with a Nicolet iS50 equipped with an iS50 Raman module equipped with 1064 nm excitation laser.

### Elemental Analysis

Elemental analysis was performed on a calibrated Flash 2000 CHNS organic automatic microanalyzer (Thermo Fisher Scientific) and was performed only on compounds that are sufficiently stable to allow brief handling in air, although most of them decompose upon prolonged air exposure. Unfortunately, for most of the tested compounds, the required deviation of <0.4% was not achieved. This was observed even for air-stable samples prepared from high-vacuum-dried single-crystalline material that was confirmed by sc-XRD to be free of solvent molecules, which might otherwise be a source of error. This phenomenon has been discussed in the literature recently.^32^

### sc-X-ray Crystallography

Diffraction data for the studied compounds were collected using a Bruker Venture D8 diffractometer at 150 K with graphite-monochromated Mo-Kα (0.7107 Å) radiation. The frames were integrated with the Bruker SAINT software package using a narrow frame algorithm. Data were corrected for absorption effects using the Multi-Scan method (SADABS). The obtained data were treated by XT-version 2014/5 and SHELXL-2017/1 software implemented in the APEX4 v2022.10–0 (Bruker AXS) system.^33^ All nonhydrogen atoms were refined using anisotropic displacement parameters. In the structure of [**16**·(dmso)_2_], one of the dmso solvent molecules is disordered around the inversion center. This disorder was treated by classical methods, and the appropriate atoms were split into two positions with equal occupancy. Crystallographic data (excluding structure factors) for the structural analyses have been deposited with the Cambridge Crystallographic Data Center; the CCDC numbers are mentioned in Table S13.

### Computational Methodology

Geometry optimizations of the isolated molecular structures were carried out using density functional theory (DFT) at the B3PW91/6-311+G­(2df,p)^34^ level of theory using the Gaussian16 ^35^ software package. For the Te atom, effective core potentials (ECP28MDF) and corresponding cc-pVTZ basis sets^36^ were used. Dispersion effects were modeled using Grimme’s GD3BJ parameters.^37^ Solvent effects were modeled by including the polarizable continuum model (IEF-PCM^38^ and universal force field (UFF) atomic radii) with dichloromethane as the solvent for the geometry optimization at the level of theory specified above. For **[I·(** ***o*** **-q** ^**phen**^ **)]­[SbF**_**6**_ **]**, **[I·(** ***o*** **-q** ^**phen**^ **)]^+^,** and the quinone ***o*** **-q^phen^**, the wave function file of the optimized structures was used for a topological analysis of the electron density according to the Atoms-In-Molecules partitioning scheme^39^ using AIMAll.^40^ The NCI^41^ grids were computed with NCIplot.^42^ Figures are displayed using Multiwfn^43^ and VMD.^44^

### Syntheses

#### [I­(cat^Cl^)]­Cl

Yellow crystals of **[I]­Cl** (362 mg, 1.12 mmol) and red powder of ***o*** **-q** ^**Cl**^ (275 mg, 1.12 mmol) were loaded into a Schlenk tube under an argon atmosphere. After the addition of DCM (20 mL), a dark orange solution was formed. The reaction mixture was stirred for 30 min and then evaporated at low pressure to get **[I­(cat** ^**Cl**^ **)]­Cl** in the form of a dark orange powder (637 mg, >99%, melting point 181–183 °C). Recrystallization from a concentrated DCM solution gave dark-orange single crystals of **[I­(cat** ^**Cl**^ **)]­Cl** (cocrystallized with two molecules of DCM, as confirmed by sc-XRD). ^1^H NMR (500 MHz, DCM-*d*_2_) δ (ppm): 1.67 [9H, s, (C*H*_3_)_3_C−]; 7.67 [1H, t, Ar­(C)*H*]; 7.78 [1H, t, Ar­(C)*H*]; 7.80 [1H, d, Ar­(C3/C6)*H*]; 7.96 [1H, d, Ar­(C6/C6)*H*]; 8.87 [1H, s, C*H*=N, ^1^ *J*(^13^C,^1^H) = 172.5 Hz]. ^13^C­{^1^H} NMR (125.78 MHz, DCM-*d*_2_) δ (ppm): 30.3 [s, (*C*H_3_)_3_C−]; 62.6 [qC, s, (CH_3_)_3_ *C*−]; 119.0 [s, qC, Ar­(*C*)]; 119.5 [s, qC, Ar­(*C*)]; 122.3 [s, qC, Ar­(*C*)]; 123.4 [s, qC, Ar­(*C*)]; 130.9 [s, Ar­(*C*)­H]; 132.9 [s, Ar­(*C*)­H]; 133.0 [s, Ar­(*C*)­H]; 134.6 [s, qC, Ar­(*C*)]; 136.0 [s, Ar­(*C*)­H]; 146.6 [s, qC, Ar­(*C*)]; 147.5 [s, qC, Ar­(*C*)]; 161.4 [s, *C*H=N]. ^15^N NMR (40.54 MHz, DCM-*d*_2_) δ: −71.2 ppm. ^125^Te­{^1^H} NMR (157.79 MHz, DCM-*d*_2_) δ: 1484.8 ppm.

#### [I­(cat^Cl^)]­[OTf]

Light-orange polycrystalline **[I]­[OTf]** (91.6 mg, 0.210 mmol) and red powder of ***o*** **-q** ^**Cl**^ (51.6 mg, 0.210 mmol) were loaded into a Schlenk tube under an argon atmosphere. After the addition of dry DCM-*d*_2_ (3 mL), a fine orange suspension was formed. The reaction mixture was vigorously stirred for 30 min; then, the clear orange solution was decanted from the precipitate directly into an NMR tube, proving the formation of **[I­(cat** ^**Cl**^ **)]­[OTf]**. The orange precipitate was dried in vacuo, dissolved in MeCN-*d*_3_, and analyzed by NMR, proving the same composition as the solution, being **[I­(cat** ^**Cl**^ **)]­[OTf]**. Based on these NMR spectra (see ), the formation of **[I­(cat** ^**Cl**^ **)]­[OTf]** was quantitative. No recrystallization was possible due to the extraordinarily high tendency for hydrolysis. ^1^H NMR (500 MHz, DCM-*d*_2_) δ (ppm): 1.75 [9H, s, (C*H*_3_)_3_C−]; 7.83 [1H, t, Ar­(C)*H*]; 7.91 [1H, t, Ar­(C)*H*]; 7.96 [1H, d, Ar­(C3/C6)*H*]; 8.07 [1H, d, Ar­(C6/C6)*H*]; 9.04 [1H, s, C*H*=N, ^*n*^ *J*(Te,^1^H) = 29.6 Hz, ^1^ *J*(^13^C,^1^H) = 175.7 Hz]. ^13^C­{^1^H} NMR (125.78 MHz, DCM-*d*_2_) δ (ppm): 30.8 [s, (*C*H_3_)_3_C−]; 64.0 [qC, s, (CH_3_)_3_ *C*−]; 119.1 [s, qC, Ar­(*C*)]; 120.0 [q, qC, −*C*F_3_, ^1^ *J*(^13^C,^1^H) = 319.1 Hz]; 120.3 [s, qC, Ar­(*C*)]; 123.6 [s, qC, Ar­(*C*)]; 125.0 [s, qC, Ar­(*C*)]; 133.4 [s, Ar­(*C*)­H]; 133.7 [s, Ar­(*C*)­H]; 134.8 [s, Ar­(*C*)­H]; 136.5 [s, Ar­(*C*)­H]; 137.6 [s, qC, Ar­(*C*)]; 142.8 [s, qC, Ar­(*C*)]; 144.5 [s, qC, Ar­(*C*)]; 148.0 [s, qC, Ar­(*C*)]; 164.8 [s, *C*H=N]. ^15^N NMR (40.54 MHz, DCM-*d*_2_) δ: −85.2 ppm. ^19^F NMR (470.66 MHz, DCM-*d*_2_) δ: −79.0 ppm [s, OTf, −C*F*_3_]. ^125^Te­{^1^H} NMR (157.79 MHz, DCM-*d*_2_) δ: 1540.4 ppm (s). ^1^H NMR (500 MHz, MeCN-*d*_3_) δ (ppm): 1.72 [9H, s, (C*H*_3_)_3_C−]; 7.88 [1H, t, Ar­(C)*H*]; 7.94 [1H, t, Ar­(C)*H*]; 8.08 [1H, d, Ar­(C3/C6)*H*]; 8.09 [1H, d, Ar­(C6/C6)*H*]; 9.36 [1H, s, C*H*=N, ^*n*^ *J*(Te,^1^H) = 33.0 Hz, ^1^ *J*(^13^C,^1^H) = 180.4 Hz]. ^13^C­{^1^H} NMR (125.78 MHz, MeCN-*d*_3_) δ (ppm): 30.9 [s, (*C*H_3_)_3_C−]; 64.7 [qC, s, (CH_3_)_3_ *C*−]; 119.4 [s, qC, Ar­(*C*)]; q, qC, −*C*F_3_ not observed; 120.2 [s, qC, Ar­(*C*)]; 123.4 [s, qC, Ar­(*C*)]; 125.0 [s, qC, Ar­(*C*)]; 133.4 [s, Ar­(*C*)­H]; 135.1 [s, Ar­(*C*)­H]; 135.9 [s, Ar­(*C*)­H]; 137.0 [s, Ar­(*C*)­H]; 139.8 [s, qC, Ar­(*C*)]; 143.4 [s, qC, Ar­(*C*)]; 145.1 [s, qC, Ar­(*C*)]; 149.6 [s, qC, Ar­(*C*)]; 168.3 [s, *C*H=N]. ^15^N NMR (40.54 MHz, MeCN-*d*_3_) δ: −87.7 ppm. ^19^F NMR (470.66 MHz, MeCN-*d*_3_) δ: −79.5 ppm [s, OTf, −C*F*_3_]. ^125^Te­{^1^H} NMR (157.79 MHz, MeCN-*d*_3_) δ: 1588.8 ppm.

#### [I­(cat^Cl^)]­[SbF_6_]

Orange polycrystalline **[I]­[SbF**_**6**_ **]** (122 mg, 0.233 mmol) and red powder of ***o*** **-q** ^**Cl**^ (57.0 mg, 0.233 mmol) were loaded into a Schlenk tube under an argon atmosphere. After the addition of dry DCM (3 mL), a fine orange suspension was formed. After 30 min of vigorous stirring, the suspension was filtered via cannula, and the orange precipitate of **[I­(cat** ^**Cl**^ **)]­[SbF**_**6**_ **]** was dried in vacuo to yield an orange powder (168 mg, 94%). Due to extremely low solubility in DCM, NMR samples were prepared by dissolving the orange powder of **[I­(cat** ^**Cl**^ **)]­[SbF**_**6**_ **]** in MeCN-*d*_3_. No recrystallization was possible as this compound exhibits an extraordinary tendency for hydrolysis. ^1^H NMR (500 MHz, MeCN-*d*_3_) δ (ppm): 1.74 [9H, s, (C*H*_3_)_3_C−]; 7.89 [1H, t, Ar­(C)*H*]; 7.95 [1H, t, Ar­(C)*H*]; 8.05 [1H, d, Ar­(C3/C6)*H*]; 8.11 [1H, d, Ar­(C6/C6)*H*]; 9.38 [1H, s, C*H*=N, ^*n*^ *J*(Te,^1^H) = 35.6 Hz, ^1^ *J*(^13^C,^1^H) = 180.8 Hz]. ^13^C­{^1^H} NMR (125.78 MHz, MeCN-*d*_3_) δ (ppm): 31.1 [s, (*C*H_3_)_3_C−]; 64.8 [qC, s, (CH_3_)_3_ *C*−]; 119.3 [s, qC, Ar­(*C*)]; 120.1 [s, qC, Ar­(*C*)]; 123.3 [s, qC, Ar­(*C*)]; 125.1 [s, qC, Ar­(*C*)]; 135.0 [s, Ar­(*C*)­H]; 135.3 [s, Ar­(*C*)­H]; 136.2 [s, Ar­(*C*)­H]; 137.1 [s, Ar­(*C*)­H]; 140.3 [s, qC, Ar­(*C*)]; 142.1 [s, qC, Ar­(*C*)]; 144.6 [s, qC, Ar­(*C*)]; 149.5 [s, qC, Ar­(*C*)]; 168.7 [s, *C*H=N]. ^15^N NMR (40.54 MHz, MeCN-*d*_3_) δ: −88.6 ppm. ^19^F NMR (470.66 MHz, MeCN-*d*_3_) δ: −123.5 ppm [m, Sb*F*_6_]. ^125^Te­{^1^H} NMR (157.79 MHz, MeCN-*d*_3_) δ: 1611.1 ppm.

#### *a*/*b*-[I­(cat^*t*Bu^)]­Cl

Yellow crystals of **[I]­Cl** (89.0 mg, 0.28 mmol) and dark red crystals of ***o*** **-q** ^***t*** **Bu**^ (60.7 mg, 0.28 mmol) were loaded into a Schlenk tube under an argon atmosphere. After the addition of DCM-*d*_2_ (2 mL), a reddish-brown solution was formed. The reaction mixture was stirred for 2 min and then was transferred into an NMR tube, which was flame-sealed and then subjected to NMR analysis, which showed extremely slow formation of ***a***- and ***b*** **-[I­(cat** ^***t*** **Bu**^ **)]­Cl** in a 0.28 to 0.72 molar ratio being in a dynamic equilibrium with the starting compounds. Unfortunately, all crystallization attempts of ***a*** **/** ***b*** **-[I­(cat** ^***t*** **Bu**^ **)]­Cl** led to the formation of polycrystalline material, not suitable for *sc*-XRD. *NMR data for* ***a-[** **I­(cat*** ^***tBu***^ ***)]­Cl*** *(minor isomer):* ^1^H NMR (500 MHz, DCM-*d*_2_) δ (ppm): 1.18 [9H, s, (C*H*_3_)_3_C−]; 1.55 [9H, s, (C*H*_3_)_3_C−]; 1.60 [9H, s, (C*H*_3_)_3_C–N]; 6.46 [1H, d, Ar­(C4′/C6′)*H*]; 6.67 [1H, d, Ar­(C4′/C6′)*H*]; 7.53 [1H, t, Ar­(C4/C5)*H*]; 7.59 [1H, t, Ar­(C4/C5)*H*]; 7.73 [1H, d, Ar­(C3/C6)*H*]; 8.10 [1H, d, Ar­(C3/C6)*H*]; 8.73 [1H, s, C*H*=N]. ^13^C­{^1^H} NMR (125.78 MHz, DCM-*d*_2_) δ (ppm): 30.1 [s, (*C*H_3_)_3_C−]; 31.9 [s, (*C*H_3_)_3_C−]; 34.9 [qC, s, (CH_3_)_3_ *C*– *C3′*/*C5*′]; 35.2 [qC, s, (CH_3_)_3_ *C*–*C3′*/*C5*′]; 61.5 [qC, s, (CH_3_)_3_ *C*–N]; 110.8 [s, Ar­(*C4′*/*C*6*′*)­H]; 114.5 [s, Ar­(*C4′*/*C*6*′*)­H]; 131.3 [s, Ar­(*C*)­H]; 132.0 [s, Ar­(*C*)­H]; 132.3 [s, Ar­(*C*)­H]; 134.6 [s, Ar­(*C*)­H]; 137.0 [s, qC, Ar­(*C*)]; 143.4 [s, qC, Ar­(*C*)]; 144.5 [s, qC, Ar­(*C*)]; 145.7 [s, qC, Ar­(*C*)]; 147.3 [s, qC, Ar­(*C*)]; 149.8 [s, qC, Ar­(*C*)]; 160.6 [s, *C*H=N]. ^15^N NMR (40.54 MHz, DCM-*d*_2_) δ: −62.6 ppm. ^125^Te­{^1^H} NMR (157.79 MHz, DCM-*d*_2_) δ: 1465.8 ppm (s). *NMR data for* ***b-[** **I­(cat*** ^***tBu***^ ***)]­Cl*** *(major isomer):* ^1^H NMR (500 MHz, DCM-*d*_2_) δ (ppm): 1.10 [9H, s, (C*H*_3_)_3_C−]; 1.22 [9H, s, (C*H*_3_)_3_C−]; 1.63 [9H, s, (C*H*_3_)_3_C–N]; 6.61 [1H, d, Ar­(C4′/C6′)*H*]; 6.99 [1H, d, Ar­(C4′/C6′)*H*]; 7.57 [1H, t, Ar­(C4/C5)*H*]; 7.65 [1H, t, Ar­(C4/C5)*H*]; 7.68 [1H, d, Ar­(C3/C6)*H*]; 7.99 [1H, d, Ar­(C3/C6)*H*]; 8.71 [1H, s, C*H*=N]. ^13^C­{^1^H} NMR (125.78 MHz, DCM-*d*_2_) δ (ppm): 29.4 [s, (*C*H_3_)_3_C−]; 30.3 [s, (*C*H_3_)_3_C−]; 32.0 [s, (*C*H_3_)_3_C−]; 34.8 [qC, s, (CH_3_)_3_ *C*– *C3′* + *C5*′]; 61.5 [qC, s, (CH_3_)_3_ *C*–N]; 111.5 [s, Ar­(*C4′*/*C*6′)­H]; 115.3 [s, Ar­(*C4′*/*C*6′)­H]; 131.5 [s, Ar­(*C*)­H]; 132.0 [s, Ar­(*C*)­H]; 132.1 [s, Ar­(*C*)­H]; 134.5 [s, Ar­(*C*)­H]; 135.1 [s, qC, Ar­(*C*)]; 136.5 [s, qC, Ar­(*C*)]; 142.7 [s, qC, Ar­(*C*)]; 145.7 [s, qC, Ar­(*C*)]; 148.6 [s, qC, Ar­(*C*)]; 160.5 [s, *C*H=N]. ^15^N NMR (40.54 MHz, DCM-*d*_2_) δ: −62.6 ppm. ^125^Te­{^1^H} NMR (157.79 MHz, DCM-*d*_2_) δ: 1441.5 ppm (s).

#### *a*/*b*-[I­(cat^*t*Bu^)]­[OTf]

Light-orange polycrystalline **[I]­[OTf]** (120.0 mg, 0.275 mmol) and dark red crystals of ***o*** **-q** ^***t*** **Bu**^ (60.5 mg, 0.275 mmol) were loaded into a Schlenk tube under an argon atmosphere. After the addition of DCM-*d*_2_ (3 mL), a reddish-brown solution was formed. The reaction mixture was stirred for 2 min and then transferred into an NMR tube, which was subsequently flame-sealed and analyzed by NMR, indicating the immediate formation of ***a*** **/** ***b*** **-[I­(cat** ^***t*** **Bu**^ **)]­[OTf]** in a 0.35 to 0.65 molar ratio being in a dynamic equilibrium with the starting compounds. All attempts to crystallize this mixture failed due to the extraordinary tendency for hydrolysis of the formed ***a*** **/** ***b*** **-[I­(cat** ^***t*** **Bu**^ **)]­[OTf]**. *NMR data for* ***a-[** **I­(cat*** ^***tBu***^ ***)]­[OTf]*** *(minor isomer):* ^1^H NMR (500 MHz, DCM-*d*_2_) δ (ppm): 1.20 [9H, s, (C*H*_3_)_3_C−]; 1.54 [9H, br. s, (C*H*_3_)_3_C−]; 1.73 [9H, s, (C*H*_3_)_3_C–N]; 6.68 [1H, br. s, Ar­(C4′/C6′)*H*]; 6.81 [1H, br. s, Ar­(C4′/C6′)*H*]; 7.73 [1H, t, Ar­(C4/C5)*H*]; 7.77 [1H, t, Ar­(C4/C5)*H*]; 7.96 [1H, d, Ar­(C3/C6)*H*]; 8.09 [1H, d, Ar­(C3/C6)*H*]; 9.04 [1H, s, C*H*=N, ^*n*^ *J*(Te,^1^H) = ∼30 Hz (overlapped), ^1^ *J*(^13^C,^1^H) = 175.1 Hz]. ^15^N NMR (40.54 MHz, DCM-*d*_2_) δ: −80.4 ppm. ^19^F NMR (470.66 MHz, DCM-*d*_2_) δ: −79.0 ppm [s, OTf].^125^Te­{^1^H} NMR (157.79 MHz, DCM-*d*_2_) δ: 1570.9 ppm (s). *NMR data for* ***b-[** **I­(cat*** ^***tBu***^ ***)]­[OTf]*** *(major isomer):* ^1^H NMR (500 MHz, DCM-*d*_2_) δ (ppm): 1.20 [9H, s, (C*H*_3_)_3_C−]; 1.27 [9H, s, (C*H*_3_)_3_C−]; 1.73 [9H, s, (C*H*_3_)_3_C–N]; 6.68 [1H, br. s, Ar­(C4′/C6′)*H*]; 7.01 [1H, br. s, Ar­(C4′/C6′)*H*]; 7.73 [1H, t, Ar­(C4/C5)*H*]; 7.77 [1H, t, Ar­(C4/C5)*H*]; 7.91 [1H, d, Ar­(C3/C6)*H*]; 8.00 [1H, d, Ar­(C3/C6)*H*]; 9.02 [1H, s, C*H*=N, ^*n*^ *J*(Te,^1^H) = ∼30 Hz (overlapped), ^1^ *J*(^13^C,^1^H) = 174.4 Hz]. ^15^N NMR (40.54 MHz, DCM-*d*_2_) δ: −79.4 ppm. ^19^F NMR (470.66 MHz, DCM-*d*_2_) δ: −79.0 ppm [s, OTf]. ^125^Te­{^1^H} NMR (157.79 MHz, DCM-*d*_2_) δ: 1535.8 ppm (s).

#### *a*/*b*-[I­(cat^tBu^)]­[SbF_6_]

Orange polycrystalline **[I]­[SbF**_**6**_ **]** (68.5 mg, 0.131 mmol) and dark red crystals of ***o*** **-q** ^***t*** **Bu**^ (29.0 mg, 0.131 mmol) were loaded into a Schlenk tube under an argon atmosphere. After the addition of DCM-*d*_2_ (2 mL), a reddish-brown solution was formed. The reaction mixture was stirred for 2 min and then was transferred into an NMR tube, which was subsequently flame-sealed and analyzed by NMR, which showed immediate formation of ***a*** **/** ***b*** **-[I­(cat** ^***t*** **Bu**^ **)]­[SbF**_**6**_ **]** in a 0.46 to 0.54 molar ratio being in a dynamic equilibrium with the starting compounds. All attempts to crystallize this mixture failed due to the extraordinary tendency for hydrolysis of the formed ***a*** **/** ***b*** **-[I­(cat** ^***t*** **Bu**^ **)]­[SbF**_**6**_]. *NMR data for* ***a-[I­(cat*** ^***tBu***^ ***)]­[SbF*** _***6***_ ***]*** *(minor isomer):* ^1^H NMR (500 MHz, DCM-*d*_2_) δ (ppm): 1.23 [9H, s, (C*H*_3_)_3_C−]; 1.47 [9H, vbr. s, (C*H*_3_)_3_C−]; 1.78 [9H, s, (C*H*_3_)_3_C–N]; 6.72–6.90 [2H, vbr. s, Ar­(C4′ + C6′)*H*]; 7.82 [2H, m, Ar­(C4 + C5)*H*]; 8.02 [2H, vbr. m, Ar­(C3 + C6)*H*]; 9.11 [1H, s, C*H*=N, ^*n*^ *J*(Te,^1^H) = 35.9 Hz, ^1^ *J*(^13^C,^1^H) = 176.3 Hz]. ^13^C­{^1^H} NMR (125.78 MHz, DCM-*d*_2_) δ (ppm): 29.7 [s, (*C*H_3_)_3_C−]; 31.4 [s, (*C*H_3_)_3_C−]; 32.2 [s, (*C*H_3_)_3_C−]; 34.8 [qC, s, (CH_3_)_3_ *C*– *C3′*/*C5*′]; 35.0 [qC, s, (CH_3_)_3_ *C*–*C3′*/*C5*′]; 65.7 [qC, vbr. s, (CH_3_)_3_ *C*–N]; 110.1 [s, Ar­(*C4′*/*C*6′)­H]; 116.8 [s, Ar­(*C4′*/*C*6′)­H]; 132.8 [s, Ar­(*C*)­H]; 133.9 [s, Ar­(*C*)­H]; 134.9 [s, Ar­(*C*)­H]; 135.7 [s, Ar­(*C*)­H]; 137.4 [s, qC, Ar­(*C*)]; 139.1 [s, qC, Ar­(*C*)]; 140.3 [s, qC, Ar­(*C*)]; 141.4 [s, qC, Ar­(*C*)]; 143.4 [s, qC, Ar­(*C*)]; 146.8 [s, qC, Ar­(*C*)]; 165.2 [s, *C*H=N]. ^15^N NMR (40.54 MHz, DCM-*d*_2_) δ: −83.6 ppm. ^125^Te­{^1^H} NMR (157.79 MHz, DCM-*d*_2_) δ: 1585.7 ppm (s). *NMR data for* ***b-[I­(cat*** ^***tBu***^ ***)]­[SbF*** _***6***_ ***]*** *(major isomer):* ^1^H NMR (500 MHz, DCM-*d*_2_) δ (ppm): 1.23 [18H, s, (C*H*_3_)_3_C−]; 1.78 [9H, s, (C*H*_3_)_3_C–N]; 6.72 [1H, br. s, Ar­(C4′/C6′)*H*]; 6.90 [1H, br. s, Ar­(C4′/C6′)*H*]; 7.82 [2H, m, Ar­(C4 + C5)*H*]; 8.02 [2H, vbr. m, Ar­(C3 + C6)*H*]; 9.11 [1H, s, C*H*=N, ^*n*^ *J*(Te,^1^H) = 35.9 Hz, ^1^ *J*(^13^C,^1^H) = 176.3 Hz]. ^13^C­{^1^H} NMR (125.78 MHz, DCM-*d*_2_) δ (ppm): 29.2 [s, (*C*H_3_)_3_C−]; 30.6 [s, (*C*H_3_)_3_C−]; 30.9 [s, (*C*H_3_)_3_C−]; 34.7 [qC, s, (CH_3_)_3_ *C*– *C3′* + *C5*′]; 65.7 [qC, vbr. s, (CH_3_)_3_ *C*–N]; 111.9 [s, Ar­(*C4′*/*C*6′)­H]; 115.8 [s, Ar­(*C4′*/*C*6′)­H]; 132.7 [s, Ar­(*C*)­H]; 133.9 [s, Ar­(*C*)­H]; 134.9 [s, Ar­(*C*)­H]; 135.9 [s, Ar­(*C*)­H]; 136.4 [s, qC, Ar­(*C*)]; 137.6 [s, qC, Ar­(*C*)]; 138.2 [s, qC, Ar­(*C*)]; 145.4 [s, qC, Ar­(*C*)]; 150.5 [s, qC, Ar­(*C*)]; 165.2 [s, *C*H=N]. ^15^N NMR (40.54 MHz, DCM-*d*_2_) δ: −83.6 ppm. ^125^Te­{^1^H} NMR (157.79 MHz, DCM-*d*_2_) δ: 1556.5 ppm (s).

#### [I·(*o*-q^phen^)]­[SbF_6_]

Orange polycrystals of **[I]­[SbF**_**6**_ **]** (213 mg, 0.407 mmol) and orange powder of ***o*** **-q** ^**phen**^ (84.7 mg, 0.407 mmol) were loaded into a Schlenk tube under an argon atmosphere. After the addition of DCM-*d*_2_ (3 mL), a deep red (almost black) solution was formed. This process was quite exothermic as the solution started to boil. From this hot solution, large amounts of deep red single crystals of **[I·(** ***o*** **-q** ^**phen**^ **)]­[SbF**_**6**_ **]** formed upon cooling to RT, while the reddish mother liquor contained only a mixture of starting compounds as judged by multinuclear NMR spectroscopy. The isolated yield of **[I·(** ***o*** **-q** ^**phen**^ **)]­[SbF**_**6**_ **]** in the form of reddish-black single crystals was 277 mg (93%, mp 192–194 °C). Elemental analysis calcd for C_25_H_22_F_6_NO_2_SbTe: C, 41.03; H, 3.03; N, 1.91; found: C, 40.64 ± 0.25; H, 3.13 ± 0.30; N, 1.65 ± 0.23.

### Compound **1**

17.6 mg (40.3 μmol, 1 mol % vs Si–H) of **[I]­[OTf]** and 991 mg (4.03 mmol) of ***o*** **-q** ^**Cl**^ were loaded into a Schlenk tube and dissolved in 25 mL of dry and degassed acetonitrile under an argon atmosphere. Subsequently, 0.64 mL (4.03 mmol) of neat Et_3_SiH was added to this dark red solution under vigorous stirring. Within 12 h, the dark red color of the reaction mixture changed to brown-ginger. Then, this solution was evaporated at low pressure and dried in vacuo to give a ginger-green oil. This oil was analyzed by NMR in C_6_D_6_, showing the presence of not only the expected product **1** (in ca. 90% yield) but also hydrolyzed catechol and traces of other species. All attempts to crystallize compound **1** from this mixture resulted only in decomposition (probably hydrolysis or intermolecular proton attack to the Si–O bond), outwardly manifesting by green colorization of the oil caused by unknown species. ^1^H NMR (500.20 MHz, C_6_D_6_) δ (ppm): 0.65 [6H, q, (CH_3_–C*H*_2_)_3_Si−]; 0.92 [9H, t, (C*H*_3_–CH_2_)_3_Si−]; 5.00 [1H, s, O*H*]. ^13^C­{^1^H} NMR (125.78 MHz, C_6_D_6_) δ (ppm): 6.1 [s, (CH_3_–*C*H_2_)_3_Si–, ^1^ *J*(^29^Si,^13^C) = 59.7 Hz]; 7.2 [s, (*C*H_3_–CH_2_)_3_Si−]; 119.4 [qC, Ar–(*C*)­H]; 124.5 [qC, Ar–(*C*)­H]; 125.1 [qC, Ar–(*C*)­H]; 125.5 [qC, Ar–(*C*)­H]; 141.2 [qC, Ar–(*C1 or 2*)­H]; 144.5 [qC, Ar–(*C1 or 2*)­H]. ^29^Si­{^1^H} NMR (99.38 MHz, C_6_D_6_) δ: 26.9 (s) ppm.

### Compound **2**

13.0 mg (29.8 μmol, 1 mol % vs Si–H) of **[I]­[OTf]** and 656 mg (2.98 mmol) of ***o*** **-q** ^***t*** **Bu**^ were loaded into a Schlenk tube and dissolved in 25 mL of dry and degassed acetonitrile under an argon atmosphere. Subsequently, 476 μL (2.98 mmol) of neat Et_3_SiH was added to this dark red solution under vigorous stirring. After a minute, the reaction mixture spontaneously heated up while the color of the solution gradually faded, resulting in the formation of a yellow solution after ca. 10 min. Then, this solution was evaporated at low pressure and dried in vacuo to give a yellow oil. From this oil, compound **2** was extracted by hexane (10 mL). After evaporation of the hexane at low pressure and drying in vacuo, a colorless oil of **2** was obtained (1.00 g, >99%). Elemental analysis calcd for C_20_H_36_O_2_Si: C, 71.37; H, 10.78; found: C, 72.21 ± 0.18; H, 10.25 ± 0.06. ^1^H NMR (500.20 MHz, C_6_D_6_) δ (ppm): 0.64 [6H, q, (CH_3_–C*H*_2_)_3_Si−]; 0.90 [9H, t, (C*H*_3_–CH_2_)_3_Si−]; 1.32 [9H, s, (C*H*_3_)_3_C–Ar­(C5)]; 1.61 [9H, s, (C*H*_3_)_3_C–Ar­(C3)]; 5.97 [1H, s, O*H*]; 6.93 [1H, m, Ar­(C6)*H*]; 7.14 [1H, m, Ar­(C4)*H*]. ^13^C­{^1^H} NMR (125.78 MHz, C_6_D_6_) δ (ppm): 5.8 [s, (CH_3_–*C*H_2_)_3_Si–, ^1^ *J*(^29^Si,^13^C) = 59.7 Hz]; 7.1 [s, (*C*H_3_–CH_2_)_3_Si−]; 30.2 [s, (*C*H_3_)_3_C–Ar­(C3)]; 32.2 [s, (*C*H_3_)_3_C–Ar­(C5)]; 34.8 [s, qC, (CH_3_)_3_ *C*–Ar­(C5)]; 35.6 [s, qC, (CH_3_)_3_ *C*–Ar­(C3)]; 113.2 [s, Ar–(*C6*)­H]; 116.8 [s, Ar–(*C4*)­H]; 135.7 [s, qC, Ar–(*C3*)]; 141.8 [s, qC, Ar–(*C5*)]; 142.9 [s, qC, Ar–(*C1 or 2*)]; 144.3 [s, qC, Ar–(*C1 or 2*)]. ^29^Si­{^1^H} NMR (99.38 MHz, C_6_D_6_) δ: 23.1 (s) ppm.

### Compounds **3** and **3′**

22.3 mg (51.1 μmol, 1 mol % vs Si–H) of **[I]­[OTf]** (a catalyst) and 1.06 g (5.11 mmol) of ***o*** **-q** ^**phen**^ were loaded into a Schlenk tube and suspended in 25 mL of dry and degassed acetonitrile under an argon atmosphere. Subsequently, 0.82 mL (5.11 mmol) of neat Et_3_SiH was added to this orange solution under vigorous stirring. After a minute, the reaction mixture spontaneously heated up to ca. 50 °C, while the suspension dissolved, resulting in the formation of an orange solution. This solution was stirred for ca. 10 min, to allow to cool down to RT. Then, it was evaporated at low pressure and dried in vacuo to give an orange oil. This oil was extracted with hexane (20 mL). After evaporation of the hexane at low pressure and drying in vacuo, an orange oil consisting of **3** and **3′** in a 4:1 molar ratio was obtained (1.45 g, 98%). This oil was successfully transformed to a crystalline light-orange powder by repeated freezing (using liquid nitrogen) and melting in vacuo. Unfortunately, all attempts to separate **3** and **3′** by fractional crystallization failed, probably due to the very similar high solubility even in hexane. Interestingly, the molar ratio of **3** and **3′** (4:1) turned out to be almost independent of the reaction conditions. We tested homogenization of the reaction mixture from the beginning by adding dichloromethane to the reaction mixture prior to the addition of the silane (as ***o*** **-q** ^**phen**^ is highly soluble in DCM while almost insoluble in MeCN); however, no effect on the **3**:**3′** molar ratio was observed. Likewise, no impact was observed when an excess of silane (300%) was used. The same applies for lowering the reaction temperature down to −20 °C for 12 h. *NMR data for* ***3*** *(major product):* ^1^H NMR (500.20 MHz, C_6_D_6_) δ (ppm): 0.65 [6H, q, (CH_3_–C*H*_2_)_3_Si−]; 0.84 [9H, t, (C*H*_3_–CH_2_)_3_Si−]; 5.53 [1H, s, O*H*]; 7.35 [1H, t, Ar­(C)*H*]; 7.38 [1H, t, Ar­(C)*H*]; 7.49 [1H, t, Ar­(C)*H*]; 7.51 [1H, t, Ar­(C)*H*]; 8.21 [1H, d, Ar­(C)*H*]; 8.44 [1H, d, Ar­(C)*H*]; 8.46 [1H, d, Ar­(C)*H*]; 8.55 [1H, d, Ar­(C)*H*]. ^13^C­{^1^H} NMR (125.78 MHz, C_6_D_6_) δ (ppm): 6.1 [s, (CH_3_–*C*H_2_)_3_Si–, ^1^ *J*(^29^Si,^13^C) = 59.2 Hz]; 7.2 [s, (*C*H_3_–CH_2_)_3_Si−]; 122.1, 122.7, 123.3, 123.7, 125.0, 126.1, 127.1, 127.3 [8× s, Ar–(*C*)­H]; 126.9, 127.7, 129.6, 130.9, 133.1 [5× s, qC, Ar­(*C*)]; 139.1 [s, qC, Ar­(*C1 and/or 2*)]. ^29^Si­{^1^H} NMR (99.38 MHz, C_6_D_6_) δ: 25.2 (s) ppm. *NMR data for* ***3′*** *(minor product):* ^1^H NMR (500.20 MHz, C_6_D_6_) δ (ppm): 0.86 [12H, q, 2× (CH_3_–C*H*_2_)_3_Si−]; 0.94 [18H, t, 2× (C*H*_3_–CH_2_)_3_Si−]; 7.38 [2H, t, 2× Ar­(C)*H*]; 7.50 [2H, t, 2× Ar­(C)*H*]; 8.40 [2H, d, 2× Ar­(C)*H*]; 8.47 [2H, d, 2× Ar­(C)*H*]. ^13^C­{^1^H} NMR (125.78 MHz, C_6_D_6_) δ (ppm): 6.2 [s, (CH_3_–*C*H_2_)_3_Si–, ^1^ *J*(^29^Si,^13^C) = 59.4 Hz]; 7.4 [s, (*C*H_3_–CH_2_)_3_Si−]; 123.4, 123.4, 125.7, 126.9 [4× s, Ar–(*C*)­H]; 133.1 [s, qC, Ar­(*C*)]; 138.6 [s, qC, Ar­(*C1*)]. ^29^Si­{^1^H} NMR (99.38 MHz, C_6_D_6_) δ: 23.8 (s) ppm.

### Compound **4**

14.3 mg (32.8 μmol, 1 mol % vs Si–H) of **[I]­[OTf]** and 806 mg (3.28 mmol) of ***o*** **-q** ^**Cl**^ were loaded into a Schlenk tube and dissolved in 25 mL of dry and degassed acetonitrile under an argon atmosphere. Subsequently, this dark red solution was quickly added to solid Ph_3_SiH (854 mg, 3.28 mmol) in another Schlenk tube under vigorous stirring. The reaction mixture was stirred overnight, i.e., until the dark red solution turned to an orange suspension. Then, this suspension was filtrated to give a ginger powder and an orange mother liquor. The ginger powder was washed with 1 mL of MeCN and dried in vacuo to give the first fraction of **4**. The orange mother liquor was concentrated, and after staying at 6 °C for two days, ginger polycrystals of **4** (the second fraction, mp = 161–163 °C) were obtained. Elemental analysis calcd for C_24_H_16_Cl_4_O_2_Si: C, 56.94; H, 3.19; found: C, 60.98 ± 0.07; H, 3.30 ± 0.07. The total isolated yield of **4** was 1.216 g (73%); however, NMR analysis of the final mother liquor proved signals for **4** with no presence of starting ***o*** **-q** ^**Cl**^, meaning that the consumption of the quinone was >99%. ^1^H NMR (500.20 MHz, C_6_D_6_) δ (ppm): 5.07 [1H, s, O*H*]; 7.09–7.14 [9H, m, *m*-Ph_3_Si–*H* and *p*-Ph_3_Si–*H*]; 7.74 [6H, dd, *o*-Ph_3_Si−]. ^13^C­{^1^H} NMR (125.78 MHz, C_6_D_6_) δ (ppm): 119.6 [s, qC, Ar–(*C–*Cl)]; 124.5 [s, qC, Ar–(*C–*Cl)]; 125.9 [s, qC, Ar–(*C–*Cl)]; 126.1 [s, qC, Ar–(*C–*Cl)]; 128.7 [s, *m*-*C*H, Ph_3_Si−]; 131.3 [s, *p*-*C*H, Ph_3_Si−]; 133.5 [qC, *ipso-C*, Ph_3_Si–, ^1^ *J*(^29^Si,^13^C) = 82.7 Hz]; 136.1 [s, *o-C*H, Ph_3_Si−]; 140.5 [s, qC, Ar–(*C1 or 2*)]; 144.7 [s, qC, Ar–(*C1 or 2*)]. ^29^Si­{^1^H} NMR (99.38 MHz, C_6_D_6_) δ: −8.8 (s) ppm.

### Compound **5**

10.1 mg (23.1 μmol, 1 mol % vs Si–H) of **[I]­[OTf]** and 509 mg (2.31 mmol) of ***o*** **-q** ^***t*** **Bu**^ were loaded into a Schlenk tube and dissolved in 25 mL of dry and degassed acetonitrile under an argon atmosphere. Subsequently, this dark red solution was quickly added to a solid Ph_3_SiH (602 mg, 2.31 mmol) in another Schlenk tube under vigorous stirring. The reaction mixture was stirred for 2 days, i.e., until the dark red color solution turned orange. Then, this solution was evaporated at low pressure and dried in vacuo to give an orange oil. From this oil, compound **5** was extracted from boiling hexane (20 mL), which led to the formation of a yellowish hot solution, from which colorless single crystals of **5** (756 mg, mp = 126–127 °C) formed upon storing at RT. The second fraction of crystalline **5** (287 mg) was obtained by keeping the mother liquor overnight at 6 °C. A combined 1.043 g (94%) of **5** was obtained. Elemental analysis calcd for C_32_H_36_O_2_Si: C, 79.95; H, 7.55; found: C, 80.41 ± 0.05; H, 7.99 ± 0.06. Importantly, NMR analysis of the final mother liquor proved only signals for **5** with no presence of starting ***o*** **-q** ^***t*** **Bu**^, meaning that the consumption of the quinone was >99%. ^1^H NMR (500.20 MHz, C_6_D_6_) δ (ppm): 1.10 [9H, s, (C*H*_3_)_3_C–Ar­(C5)]; 1.62 [9H, s, (C*H*_3_)_3_C–Ar­(C3)]; 6.16 [1H, s, O*H*]; 6.87 [1H, m, Ar­(C6)*H*]; 7.10 [6H, t, *m*-Ph_3_Si−]; 7.14 [1H, m, Ar­(C4)*H*]; 7.15 [3H, t, *p*-Ph_3_Si−]; 7.68 [6H, d, *o*-Ph_3_Si−]. ^13^C­{^1^H} NMR (125.78 MHz, C_6_D_6_) δ (ppm): 30.2 [s, (*C*H_3_)_3_C–Ar­(C3)]; 31.9 [s, (*C*H_3_)_3_C–Ar­(C5)]; 34.8 [s, qC, (CH_3_)_3_ *C*–Ar­(C5)]; 35.7 [s, qC, (CH_3_)_3_ *C*–Ar­(C3)]; 114.7 [s, Ar–(*C6*)­H]; 117.0 [s, Ar–(*C4*)­H]; 128.8 [s, *m*-*C*H, Ph_3_Si−]; 131.1 [s, *p*-*C*H, Ph_3_Si−]; 133.8 [qC, *ipso*-*C*, Ph_3_Si–, ^1^ *J*(^29^Si,^13^C) = 81.6 Hz]; 135.9 [s, qC, Ar–(*C3*)]; 135.9 [s, *o*-*C*H, Ph_3_Si−]; 141.6 [s, qC, Ar–(*C5*)]; 142.9 [s, qC, Ar–(*C1 or 2*)]; 144.1 [s, qC, Ar–(*C1 or 2*)]. ^29^Si­{^1^H} NMR (99.38 MHz, C_6_D_6_) δ: −11.6 (s) ppm.

### Compound **6**

6.7 mg (15.4 μmol, 1 mol % vs Si–H) of **[I]­[OTf]**, 321 mg (1.54 mmol) of ***o*** **-q^phen^**, and 402 mg of solid Ph_3_SiH (1.54 mmol) were loaded into a Schlenk tube and suspended in 25 mL of dry and degassed acetonitrile under an argon atmosphere. After 10 min of vigorous stirring, the orange suspension started to dissolve, and a white powder started to precipitate just a moment later. The reaction mixture was stirred overnight to give a very fine yellowish suspension. After filtration, the white precipitate was washed with 2 mL of MeCN and then dried in vacuo. The white powder was recrystallized from hot toluene (5 mL), and light-ginger single crystals of **6** were obtained upon cooling the solution at 6 °C (515 mg, 71%, mp = 183–185 °C). Elemental analysis calcd for C_32_H_24_O_2_Si: C, 82.02; H, 5.16; found: C, 82.79 ± 0.02; H, 5.47 ± 0.07. Importantly, NMR analysis of both the MeCN–mother liquor from the white suspension and toluene–mother liquor proved signals for **6** with no presence of starting ***o*** **-q** ^**phen**^, meaning that the consumption of the quinone was >99%. ^1^H NMR (500.20 MHz, C_6_D_6_) δ (ppm): 5.67 [1H, s, O*H*]; 7.05 [6H, t, *m*-Ph_3_Si−]; 7.09 [3H, t, *p*-Ph_3_Si−]; 7.19 [1H, t, Ar­(C)*H*]; 7.25 [1H, t, Ar­(C)*H*]; 7.31 [1H, t, Ar­(C)*H*]; 7.37 [1H, t, Ar­(C)*H*]; 7.79 [6H, d, *o*-Ph_3_Si−]; 8.33 [1H, d, Ar­(C)*H*]; 8.34 [1H, d, Ar­(C3)*H*]; 8.36 [1H, d, Ar­(C)*H*]; 8.42 [1H, d, Ar­(C)*H*]. ^13^C­{^1^H} NMR (125.78 MHz, C_6_D_6_) δ (ppm): 122.7 [s, Ar–(*C3*)­H]; 122.9 [s, Ar–(*C*)­H, belongs to 8.42 ppm (d) in ^1^H NMR]; 123.3 [s, Ar–(*C*)­H, belongs to 8.36 ppm (d) in ^1^H NMR]; 123.4 [s, Ar–(*C*)­H, belongs to 8.33 ppm (d) in ^1^H NMR]; 124.9 [s, Ar–(*C*)­H, belongs to 7.19 ppm (t) in ^1^H NMR]; 126.2 [s, Ar–(*C*)­H, belongs to 7.31 ppm (t) in ^1^H NMR]; 127.0 [s, qC, Ar–(*C*)]; 127.1 [s, Ar–(*C*)­H, belongs to 7.34 ppm (t) in ^1^H NMR]; 127.2 [s, Ar–(*C*)­H, belongs to 7.37 ppm (t) in ^1^H NMR]; 127.6 [s, qC, Ar–(*C*)]; 128.7 [s, qC, Ar–(*C*)]; 129.08 [s, *m*-*C*H, Ph_3_Si−]; 129.5 [s, qC, Ar–(*C*)]; 131.4 [s, *p*-*C*H, Ph_3_Si−]; 132.7 [s, qC, Ar–(*C*)]; 133.7 [qC, *ipso*-*C*, Ph_3_Si–, ^1^ *J*(^29^Si,^13^C) = 81.1 Hz]; 136.1 [s, *o*-*C*H, Ph_3_Si−]; 139.2 [s, qC, Ar–(*C*)]. ^29^Si­{^1^H} NMR (99.38 MHz, C_6_D_6_) δ: −11.8 (s) ppm.

### Compound **7**

***o*** **-q** ^**Cl**^ (3.10 g, 12.6 mmol) was loaded into a Schlenk tube sealable by a PTFE stopper under an argon atmosphere. This quinone was dissolved in dry MeCN (15 mL) under the formation of a dark red solution. To this solution, Et_3_SiH (1.34 mL, 8.41 mmol) was added, and the Schlenk tube was sealed. The reaction mixture was vigorously stirred, and a bright red powder started to precipitate the next day. After 7 days of stirring, the suspension was filtrated to give a bright red powder along with a light red filtrate containing the catechol and Et_3_SiCl (as proved by the reaction on a smaller scale in deuterated MeCN; see NMR spectra in ). The bright red powder was washed from traces of catechol first with cold MeCN (5 mL) and then with DCM (3 mL) and dried in vacuo, giving 1.707 g (97%) of **7**. Elemental analysis calcd for C_12_Cl_6_O_4_: C, 34.25; found: C, 34.63 ± 0.02. ^13^C­{^1^H} NMR (125.78 MHz, C_6_D_6_) δ (ppm): 114.6, 121.5, 129.8, 134.2, 145.5 [s, qC, Ar–(*C*)], 170.5 [s, qC, *C*=O]. **7** shows low solubility in DCM, MeCN, and aromatic solvents. Single crystals of **7** were grown from boiling benzene, leading to cocrystallization **7**·(C_6_H_6_)_3_ as proved by sc-X-ray diffraction.

### Compound **8**

22.5 mg (51.5 μmol, 1 mol % vs Si–H) of **[I]­[OTf]** and 1.27 g (5.15 mmol) of ***o*** **-q** ^**Cl**^ were loaded into a Schlenk tube and dissolved in 25 mL of dry and degassed acetonitrile under an argon atmosphere. Subsequently, 0.48 mL (2.58 mmol) of neat Ph_2_SiH_2_ was added to this dark red solution under vigorous stirring. After 10 min, the reaction mixture spontaneously heated up to ca. 50 °C, while a white precipitate of **8** started to form. This reaction mixture was stirred for 2 h in total, during which the dark red solution completely transformed to a light-yellow suspension. The suspension was filtrated to give a white powder, which was washed with cold MeCN (3 mL) and dried in vacuo, which was characterized by NMR spectroscopy as **8** (869 mg, 79%). Recrystallization of this amorphous solid from a hot saturated hexane solution led to the formation of colorless single crystals of **8** (mp = 156–158 °C). Because NMR analysis of the light-yellow mother liquor proved only catechol with a minor amount of **8** with no signals for starting ***o*** **-q** ^**Cl**^, this means that the consumption of the quinone was >99%. Compound **8** is highly sensitive to moisture. ^1^H NMR (500.20 MHz, C_6_D_6_) δ (ppm): 7.01 [4H, t, *m*-Ph_2_Si]; 7.13 [2H, t, *p*-Ph_2_Si]; 7.49 [4H, dd, *o*-Ph_2_Si]. ^13^C­{^1^H} NMR (125.78 MHz, C_6_D_6_) δ (ppm): 118.3 [qC, s, Ar-(*C3* and *C6*)]; 125.7 [qC, s, Ar-(*C4* and *C5*)]; 127.5 [qC, *ipso*-*C*, Ph_2_Si]; 129.1 [s, *m*-*C*H, Ph_2_Si]; 133.3 [s, *p*-*C*H, Ph_2_Si]; 135.7 [s, *o*-*C*H, Ph_2_Si]; 146.0 [qC, s, Ar–(*C1* and *C2*)]. ^29^Si­{^1^H} NMR (99.38 MHz, C_6_D_6_) δ: 3.6 (s) ppm.

### Compound **9**

35.2 mg (80.6 μmol, 1 mol % vs Si–H) of **[I]­[OTf]** and 1.77 g (8.06 mmol) of ***o*** **-q** ^***t*** **Bu**^ were loaded into a Schlenk tube and dissolved in 25 mL of dry and degassed acetonitrile under an argon atmosphere. Subsequently, 0.75 mL (4.03 mmol) of neat Ph_2_SiH_2_ was added to this dark red solution under vigorous stirring. After 10 min, the reaction mixture spontaneously heated up to ca. 50 °C, while a white precipitate of **9** started to form. This reaction mixture was stirred for a total of 1 h, during which the dark red solution completely transformed to a light-yellow suspension. The suspension was filtrated to give white powder, which was washed with cold MeCN (3 mL) and dried in vacuo, which was identified by NMR spectroscopy as **9** (1.52 g, 94%). Recrystallization of this amorphous solid from hot saturated hexane solution led to the formation of colorless single crystals of **9** (mp = 146–148 °C). Since NMR analysis of the light-yellow mother liquor proved only catechol with a minor amount of **9** with no signals for starting ***o*** **-q** ^***t*** **Bu**^, this means that the consumption of the quinone was >99%. Compound **9** is highly sensitive to moisture. ^1^H NMR (500.20 MHz, C_6_D_6_) δ (ppm): 1.32 [9H, s, (C*H*_3_)_3_C–Ar­(C5)]; 1.59 [9H, s, (C*H*_3_)_3_C–Ar­(C3)]; 7.07 [4H, t, *m*-Ph_2_Si]; 7.15 [2H, t, *p*-Ph_2_Si]; 7.15 [1H, d, Ar­(C4)*H*]; 7.29 [1H, d, Ar­(C6)*H*]; 7.72 [4H, dd, *o*-Ph_2_Si]. ^13^C­{^1^H} NMR (125.78 MHz, C_6_D_6_) δ (ppm): 30.2 [s, (*C*H_3_)_3_C–Ar­(C3)]; 32.3 [s, (*C*H_3_)_3_C–Ar­(C5)]; 35.2 [s, qC, (CH_3_)_3_ *C*–Ar­(C5)]; 35.3 [s, qC, (CH_3_)_3_ *C*–Ar­(C3)]; 110.4 [s, Ar–(*C6*)­H]; 115.9 [s, Ar–(*C4*)­H]; 128.9 [s, *m*-*C*H, Ph_2_Si]; 130.2 [qC, *ipso*-*C*, Ph_2_Si, ^1^ *J*(^29^Si,^13^C) = 95.4 Hz]; 132.4 [s, *p*-*C*H, Ph_2_Si]; 135.8 [s, *o*-*C*H, Ph_2_Si]; 135.8 [s, qC, Ar–(*C3*)]; 144.6 [s, qC, Ar–(*C5*)]; 145.3 [s, qC, Ar–(*C2*)]; 149.7 [s, qC, Ar–(*C1*)]. ^29^Si­{^1^H} NMR (99.38 MHz, C_6_D_6_) δ: −1.5 (s) ppm (^1^ *J*(^29^Si,^13^C) = 95.4 Hz).

### Compound **10**

22.1 mg (50.6 μmol, 1 mol % vs Si–H) of **[I]­[OTf]** and 1.05 g (5.06 mmol) of ***o*** **-q** ^**phen**^ were loaded into a Schlenk tube and suspended in 25 mL of dry and degassed acetonitrile under an argon atmosphere. Subsequently, 0.47 mL (2.53 mmol) of neat Ph_2_SiH_2_ was added to this orange suspension under vigorous stirring. Immediately after the addition of the silane, the orange suspension turned to a dark red suspension while spontaneously warming up to ca. 40 °C. This reaction mixture was stirred for 2 h in total, during which the red suspension first changed to an orange solution (after 7 min from the beginning), from which a beige powder slowly precipitated. The suspension was filtrated to give a beige solid, which was washed with cold MeCN (3 mL), dried in vacuo, and characterized by NMR spectroscopy as **10** (702 mg, 71%). Due to the fact that NMR analysis of the orange mother liquor proved only 9,10-phenanthrenediol with a minor amount of **10** and a trace amount of **13** (product of hydrolysis of **10**) with no signals for starting ***o*** **-q** ^**phen**^, this means that the consumption of the quinone was >99%. From the series of compounds **8**–**10**, compound **10** is the most sensitive to moisture; thus, the melting point could not be reliably determined. ^1^H NMR (500.20 MHz, C_6_D_6_) δ (ppm): 7.06 [4H, t, *m*-Ph_2_Si]; 7.15 [2H, t, *p*-Ph_2_Si]; 7.37 [2H, t, Ar­(C4 + 11 or C5 + 10)*H*]; 7.47 [2H, t, Ar­(C5 + 10 or C4 + 11)*H*]; 7.76 [4H, d, *o*-Ph_2_Si]; 8.47 [2H, d, Ar­(C3 + 12 or C6 + 9)*H*]; 8.50 [2H, d, Ar­(C6 + 9 or C3 + 12)*H*]. ^13^C­{^1^H} NMR (125.78 MHz, C_6_D_6_) δ (ppm): 121.7 [s, Ar–(*C3* + *12* or *C6* + *9*)­H, belongs to 8.47 ppm (d) in ^1^H NMR]; 123.9 [s, Ar–(*C6* + *9* or *C3* + *12*)­H, belongs to 8.50 ppm (d) in ^1^H NMR]; 125.4 [s, Ar–(*C4* + *11* or *C5* + *10*)­H, belongs to 7.37 ppm (t) in ^1^H NMR]; 125.6 [qC, Ar­(*C2* + *13* or *C7* + *8*)­H]; 127.5 [s, Ar–(*C5* + *10* or *C4* + *11*)­H, belongs to 7.47 ppm (t) in ^1^H NMR]; 128.0 [qC, Ar­(*C7* + *8* or *C2* + *13*)­H]; 129.0 [s, *m*-*C*H, Ph_2_Si]; 129.7 [qC, *ipso*-*C*, Ph_2_Si, ^1^ *J*(^29^Si,^13^C) = 96.0 Hz]; 132. [s, *p*-*C*H, Ph_2_Si]; 136.0 [s, *o*-*C*H, Ph_2_Si]; 140.1 [s, qC, Ar-(*C1 + 14*)]. ^29^Si­{^1^H} NMR (99.38 MHz, C_6_D_6_) δ: 1.4 (s) ppm.

### Compound **11**

869 mg (2.03 mmol) of compound **8** was loaded into a Schlenk tube and dissolved in 5 mL of dry and degassed toluene under an argon atmosphere. Subsequently, 18 μL (1.01 μmol) of distilled water was added. The colorless solution was stirred overnight; then, the solvent was removed under reduced pressure, and the resulting residue was dried in vacuo to give compound **11** as a white powder in quantitative yield. ^1^H NMR (500.20 MHz, C_6_D_6_) δ (ppm): 4.78 [2H, s, 2× O*H*]; 7.08–7.13 [12H, m, *m*-C*H* + *p*-C*H*, Ph_2_Si]; 7.89 [8H, d, 4× *o*-C*H*, Ph_2_Si]. ^13^C­{^1^H} NMR (125.78 MHz, C_6_D_6_) δ (ppm): 119.4, 124.2, 125.7, 127.1 [4× s, qC, Ar–(*C*)]; 128.9 [s, *m*-*C*H, Ph_2_Si]; 131.7 [s, qC, Ar–(*C*)]; 132.1 [s, *p*-*C*H, Ph_2_Si]; 135.3 [s, *o*-*C*H, Ph_2_Si]; 140.5 [s, qC, Ar–(*C2*)]; 142.7 [s, qC, Ar–(*C1*)]. ^29^Si­{^1^H} NMR (99.38 MHz, C_6_D_6_) δ: −30.4 (s) ppm.

### Compound **12**

755 mg (1.88 mmol) of compound **9** was loaded into a Schlenk tube and dissolved in 5 mL of dry and degassed toluene under an argon atmosphere. Subsequently, 17 μL (0.94 μmol) of distilled water was added. The colorless solution was stirred overnight; then, the solvent was removed under reduced pressure, and the resulting residue was dried in vacuo to give compound **12** as a white powder in quantitative yield. Crystallization from a concentrated hot toluene solution led to the formation of colorless single crystals (mp = 142–144 °C). Elemental analysis calcd for C_52_H_62_O_5_Si_2_: C, 75.87; H, 7.59; found: C, 76.73 ± 0.16; H, 8.07 ± 0.04. ^1^H NMR (500.20 MHz, C_6_D_6_) δ (ppm): 1.09 [18H, s, 2× (C*H*_3_)_3_C–Ar­(C5)]; 1.57 [18H, s, 2× (C*H*_3_)_3_C–Ar­(C3)]; 5.86 [2H, s, 2× O*H*]; 6.92 [2H, d, 2× Ar­(C6)*H*]; 7.07 [8H, t, 2× *m*-Ph_2_Si]; 7.12 [4H, t, 2× *p*-Ph_2_Si]; 7.12 [2H, d, 2× Ar­(C4)*H*]; 7.68 [8H, d, 2× *o*-Ph_2_Si]. ^13^C­{^1^H} NMR (125.78 MHz, C_6_D_6_) δ (ppm): 30.2 [s, (*C*H_3_)_3_C–Ar­(C3)]; 31.9 [s, (*C*H_3_)_3_C–Ar­(C5)]; 34.7 [s, qC, (CH_3_)_3_ *C*–Ar­(C5)]; 35.7 [s, qC, (CH_3_)_3_ *C*–Ar­(C3)]; 114.6 [s, Ar–(*C6*)­H]; 117.6 [s, Ar–(*C4*)­H]; 128.6 [s, *m*-*C*H, Ph_2_Si]; 131.6 [s, *p*-*C*H, Ph_2_Si]; 132.9 [qC, *ipso*-*C*, Ph_2_Si]; 135.4 [s, *o*-*C*H, Ph_2_Si]; 136.1 [s, qC, Ar–(*C3*)]; 141.8, 141.9, and 143.9 [3× s, qC, Ar–(*C1*), Ar–(*C2*), and Ar–(*C5*)]. ^29^Si­{^1^H} NMR (99.38 MHz, C_6_D_6_) δ: −37.6 (s) ppm (^1^ *J*(^29^Si,^13^C) = 100.4 Hz).

### Compound **13**

510 mg (1.31 mmol) of compound **10** was loaded into a Schlenk tube and dissolved in 5 mL of dry and degassed toluene under an argon atmosphere. Subsequently, 12 μL (0.65 μmol) of distilled water was added. The colorless solution was stirred overnight; then, the solvent was removed under reduced pressure, and the resulting residue was dried in vacuo to give compound **13** as ivory powder in quantitative yield. Crystallization from a concentrated hot toluene solution with few drops of hexane led to the formation of ivory single crystals (mp = 159–163 °C (decomp.)). Elemental analysis calcd for C_52_H_38_O_5_Si_2_: C, 78.17; H, 4.79; found: C, 80.31 ± 0.01; H, 4.42 ± 0.11. Compound **13** is virtually insoluble in hexane. ^1^H NMR (500.20 MHz, C_6_D_6_) δ (ppm): 5.97 [2H, s, 2× O*H*]; 6.90 [8H, t, 2× *m*-Ph_2_Si]; 7.00 [4H, t, 2× *p*-Ph_2_Si]; 7.13 [2H, t, 2× Ar­(C)*H*]; 7.16 [2H, t, 2× Ar­(C)*H*]; 7.30 [2H, t, 2× Ar­(C)*H*]; 7.34 [2H, t, 2× Ar­(C)*H*]; 7.67 [8H, d, 2× *o*-Ph_2_Si]; 8.22 [2H, d, 2× Ar­(C)*H*]; 8.28–8.33 [26H, m in shape of q, 6× Ar­(C)*H*]. ^13^C­{^1^H} NMR (125.78 MHz, C_6_D_6_) δ (ppm): 122.6, 123.0, 123.2, 123.3, 125.0, 126.2 [6× s, Ar–(*C*)­H]; 127.1 [s, qC, Ar­(*C*)]; 127.1, 127.2 [2× s, Ar–(*C*)­H]; 128.7 [s, qC, Ar­(*C*)]; 128.8 [s, *m*-*C*H, Ph_2_Si]; 129.1 [s, qC, Ar­(*C*)]; 131.6 [s, *p*-*C*H, Ph_2_Si]; 132.2, 132.5 [2× s, qC, Ar­(*C*)]; 135.3 [s, *o*-*C*H, Ph_2_Si]; 139.0 [s, qC, Ar­(*C1*)]. ^29^Si­{^1^H} NMR (99.38 MHz, C_6_D_6_) δ: −37.8 (s) ppm.

### Compound [**14**·(EtOH)]

30.6 mg (70.0 μmol, 1 mol % vs Si–H) of **[I]­[OTf]** and 1.72 g (7.00 mmol) of ***o*** **-q** ^**Cl**^ were loaded into a Schlenk tube and dissolved in 25 mL of dry and degassed acetonitrile under an argon atmosphere. Subsequently, 1.29 mL (7.00 mmol) of neat (EtO)_3_SiH was added to this dark red solution under vigorous stirring. After 10 min, an orange suspension started to form. The reaction mixture was stirred overnight to give a very fine light orange suspension. After filtration, the orange precipitate was washed with 3 × 2 mL of cold MeCN and then dried in vacuo to give white powder of **[14·(EtOH)]** (1.73 g). Compound **[14·(EtOH)]** is extremely sensitive to moisture, leading to hydrolysis under formation of tetrachlorocatechol.

### Compound [**14**·(dmso)_2_]

A thoroughly dried dmso (10 mL) was added to 1.73 g of amorphous powder of [**14**·(EtOH)], and the Schlenk tube was sealed and heated with a heat gun to 160 °C until all the white powder dissolved. Colorless crystals of [**14**·(dmso)_2_] grew at RT (isolated yield: 1.36 g, 58%; mp = 166 °C (decomp.)) by thermally insulating the Schlenk tube to allow for a slow cooling rate. ^13^C­{^1^H} NMR (125.78 MHz, dmso-*d*_6_) δ (ppm): 111.6 [s, qC, Ar­(*C3 and C6*)]; 116.6 [s, qC, Ar­(*C4 and C5*)]; 148.5 [s, qC, Ar­(*C1* and *C2*)]. ^29^Si­{^1^H} NMR (99.38 MHz, dmso-*d*_6_) δ: −138.1 (s) ppm. In some instances, we obtained these crystals directly from the deuterated-dmso solutions, thus obtaining a compound with the composition of [**14**·(dmso-*d*_6_)_2_]. Compound [**14**·(dmso)_2_] is extremely sensitive toward moisture, leading to hydrolysis under formation of tetrachlorocatechol.

### Compound **15**

55.0 mg (126 μmol, 1 mol % vs Si–H) of **[I]­[OTf]** and 2.77 g (12.6 mmol) of ***o*** **-q** ^***t*** **Bu**^ were loaded into a Schlenk tube and dissolved in 25 mL of dry and degassed acetonitrile under an argon atmosphere. Subsequently, 2.32 mL (12.6 mmol) of neat (EtO)_3_SiH was added to this dark brown solution under vigorous stirring. The color of the solution gradually faded, which resulted in the formation of a light-orange solution after overnight stirring. Solvent from this solution was removed under reduced pressure, and the resulting orange opaque oil was dried in vacuo at temperatures not exceeding 40 °C. From this oil, compound **15** was extracted by hexane (10 mL) at RT. After evaporation of the hexane at low pressure and drying in vacuo, a colorless oil of **15** was obtained (4.13 g, >97%). ^1^H NMR (500.20 MHz, C_6_D_6_) δ (ppm): 1.00 [6H, s, 2× C*H*_3_CH_2_O]; 1.25 [9H, s, (C*H*_3_)_3_C–Ar­(C5)]; 1.53 [9H, s, (C*H*_3_)_3_C–Ar­(C3)]; 3.70 [4H, s, 2× CH_3_C*H*_2_O]; 7.08 [1H, d, Ar­(C4)*H*]; 7.15 [1H, d, Ar­(C6)*H*]. ^13^C­{^1^H} NMR (125.78 MHz, C_6_D_6_) δ (ppm): 18.3 [s, 2× *C*H_3_CH_2_O]; 30.1 [s, (*C*H_3_)_3_C–Ar­(C3)]; 32.2 [s, (*C*H_3_)_3_C–Ar­(C5)]; 35.2 [s, qC, (CH_3_)_3_ *C*–Ar­(C5)]; 35.2 [s, qC, (CH_3_)_3_ *C*–Ar­(C3)]; 61.1 [s, 2× CH_3_ *C*H_2_O]; 109.9 [s, Ar–(*C6*)­H]; 115.8 [s, Ar–(*C4*)­H]; 135.4 [s, qC, Ar–(*C3*)]; 144.2 [s, qC, Ar–(*C5*)]; 144.6 [s, qC, Ar–(*C2*)]; 148.6 [s, qC, Ar–(*C1*)]. ^29^Si­{^1^H} NMR (99.38 MHz, C_6_D_6_) δ: −63.0 (s) ppm. Compound **15** is heat sensitive and transforms into a white powder of [**16**]_2_ upon heating under elimination of oily (EtO)_4_Si (see below).

### Compound [**16**·(dmso)_2_]

3.95 g (11.7 mmol) of neat compound **15** was heated at 100 °C for 1 h in a Schlenk tube under an argon atmosphere. Right from the beginning of the heating, a white powder of [**16**]_2_ started to appear. Then, the white powder of [**16**]_2_ was washed twice with hexane (10 mL) to remove (EtO)_4_Si and dried in vacuo (2.61 g, 95%). Subsequently, thoroughly dried dmso (20 mL) was added to this amorphous powder (of [**16**]_2_) and the Schlenk tube was sealed and heated with a heat gun to 160 °C until all the white powder dissolved. Colorless crystals of [**16**·(dmso)_2_] were obtained at RT (isolated yield 2.89 g, 86%; mp = 142–144 °C) by thermally insulating the Schlenk tube to slow down the cooling rate. ^1^H NMR (500.20 MHz, dmso-*d*_6_) δ (ppm): 1.22 [9H, s, (C*H*_3_)_3_C–Ar­(C5)]; 1.37 [9H, s, (C*H*_3_)_3_C–Ar­(C3)]; 6.50 [1H, d, Ar­(C4)*H*]; 6.57 [1H, br. s, Ar­(C6)*H*]. ^13^C­{^1^H} NMR (125.78 MHz, dmso-*d*_6_) δ (ppm): 29.6 [s, (*C*H_3_)_3_C–Ar­(C3)]; 31.8 [s, (*C*H_3_)_3_C–Ar­(C5)]; 33.9 [s, qC, (CH_3_)_3_ *C*–Ar­(C5)]; 34.0 [s, qC, (CH_3_)_3_ *C*–Ar­(C3)]; 106.4 [s, Ar–(*C6*)­H]; 110.6 [s, Ar–(*C4*)­H]; 130.6 [s, qC, Ar–(*C3*)]; 138.8 [s, qC, Ar–(*C5*)]; 144.6 [s, qC, Ar–(*C2*)]; 148.9 [s, qC, Ar–(*C1*)]. ^29^Si­{^1^H} NMR (99.38 MHz, dmso-*d*_6_) δ: −115.6 (s) ppm. In some instances, we crystallized [**16**]_2_ directly from deuterated-dmso, thus obtaining a compound with the composition of [**16**·(dmso-*d*_6_)_2_].

## Supplementary Material





## Notes

NMR and FT-IR/Raman spectra, details for *sc* X-ray crystallography, discussion on the catalytic mechanism, and details for DFT (PDF)
Calculated geometries (XYZ)
