# Supplementary material for: A Substrate-Dependent Redox Catalysis of Tellurenyl Species Involving Oxidation States +I, +II, and +IV
Source: Inorg Chem. 2026 Mar 26;65(13):7224–42. doi: 10.1021/acs.inorgchem.5c05915 (PMC13058883; doi:10.1021/acs.inorgchem.5c05915)
Supplement: Supplementary file 1 [file ic5c05915_si_001.pdf]

SUPPORTING INFORMATION

A Substrate-Dependent Redox Catalysis of Tellurenyl Species Involving Oxidation States +I, +II and +IV

Martin Hejda,[a,] Lukáš Doležal,[a] Aleš Růžička,[a] Emanuel Hupf,[b,*]*

Jens Beckmann[b] and Libor Dostál[a]

[a]Department of General and Inorganic Chemistry, University of Pardubice, Studentská 573, CZ 532 10 Pardubice, Czech Republic, *E-mail: martin.hejda@upce.cz

[b]Institut für Anorganische Chemie und Kristallographie, Universität Bremen, Leobener Straße 7, 28359 Bremen, Germany, *E-mail: hupf@uni-bremen.de

Contents:

Oxidation of Te(II) complexes S3
- NMR spectra of **[I(cat^{Cl})]Cl** S3
- NMR spectra of **[I(cat^{Cl})][OTf]** S7
- NMR spectra of **[I(cat^{Cl})][SbF$_6$]** S12
- NMR spectra proving reversibility in the formation of ***a*/*b*-[I(cat^{tBu})]Cl** S15
- NMR spectra proving reversibility in the formation of ***a*/*b*-[I(cat^{tBu})][OTf]** S20
    - Influencing the equilibrium S24
- NMR spectra proving reversibility in the formation of ***a*/*b*-[I(cat^{tBu})][SbF$_6$]** S26
- Reaction of **[I]Cl** with ***o*-q^{phen}** and corresponding NMR spectra S30
- Reaction of **[I][OTf]** with ***o*-q^{phen}** and corresponding NMR data S32
- NMR and IR/Raman spectra of **[I·(o-q^{phen})][SbF$_6$]** S34
- NMR spectra obtained after dissolving **[I·(o-q^{phen})][SbF$_6$]** in MeCN-d_3 S37
- Reactions of **[I]Cl**, **[I][OTf]** and **[I][SbF$_6$]** with 1,2-cyclohexanedione (**1,2-hde**) S38

Catalyzed reactions of quinones with Et$_3$SiH using [I][OTf] as a catalyst S40
- NMR spectra of compound **1** S40
- NMR spectra of compound **2** S42
- NMR spectra of compounds **3** and **3'** S44

Catalyzed reactions of quinones with Ph$_3$SiH using [I][OTf] as a catalyst S47
- NMR spectra of compound **4** S47
- NMR spectra of compound **5** S49
- NMR spectra of compound **6** S51

Non-catalyzed reactions of quinones with Et$_3$SiH S53
- NMR spectra for reaction of ***o*-q^{Cl}** with Et$_3$SiH leading to formation of **7** S53
- Non-reactivity of ***o*-q^{tBu}** with Et$_3$SiH S55
- Non-catalyzed reaction of ***o*-q^{phen}** with Et$_3$SiH S56

Catalyzed reactions of quinones with Ph$_2$SiH$_2$ using [I][OTf] as a catalyst S58
- NMR spectra of compound **8** S58
- NMR spectra of compound **9** S60
    - Experiments proving stepwise Si-H bond activation during formation of compound **9** S62
- NMR spectra of compound **10** S65

Intentional hydrolysis of compounds 8-10 leading to compounds 11-13 S67
- NMR spectra of compound **11** S67
- NMR spectra of compound **12** S69
- NMR spectra of compound **13** S71

Non-catalyzed reactions of quinones with Ph_2SiH_2 S73
- Non-catalyzed reaction of ***o*-q**^{Cl} with Ph_2SiH_2 S73
- Non-reactivity of ***o*-q**^{tBu} with Ph_2SiH_2 S75
- Non-catalyzed reaction of ***o*-q**^{phen} with Ph_2SiH_2 S76

Catalyzed reactions of quinones with $(EtO)_3SiH$ using [I][OTf] as a catalyst S77
- NMR spectra of compound [**14**·(EtOH)] S77
- NMR spectra of compound [**14**·(dmso)$_2$] S79
- NMR spectra of compound **15** S81
- NMR spectra of compound [**16**·(dmso)$_2$] S83

Mechanistic study of the catalysis S88
- Addition of 1 eq. of Et_3SiH into a dynamic equilibrium of **[I][OTf]** + ***o*-q**^{tBu} ⇌ ***a*-[I(cat**^{tBu}**)][OTf]** + ***b*-[I(cat**^{tBu}**)][OTf]** (a stoichiometric reaction) S89
- Addition of 1 eq. of ***o*-q**^{tBu} into a mixture of **II** and Et_3SiOTf (a stoichiometric reaction) S91
- Stoichiometric reaction of ditelluride **II** and ***o*-q**^{tBu} leading to formation of oxonium species **III**^{tBu} S93
- Stoichiometric reaction of oxonium species **III**^{tBu} with Et_3SiOTf S96
- Proposed catalytic cycle for the **[I][OTf]**-catalyzed reaction of ***o*-q**^{tBu} with Et_3SiH S97

DFT computations S98
Crystallographic data for studied compounds S105
References S109

Oxidation of Te(II) complexes

NMR spectra of [I(cat^{Cl})]Cl

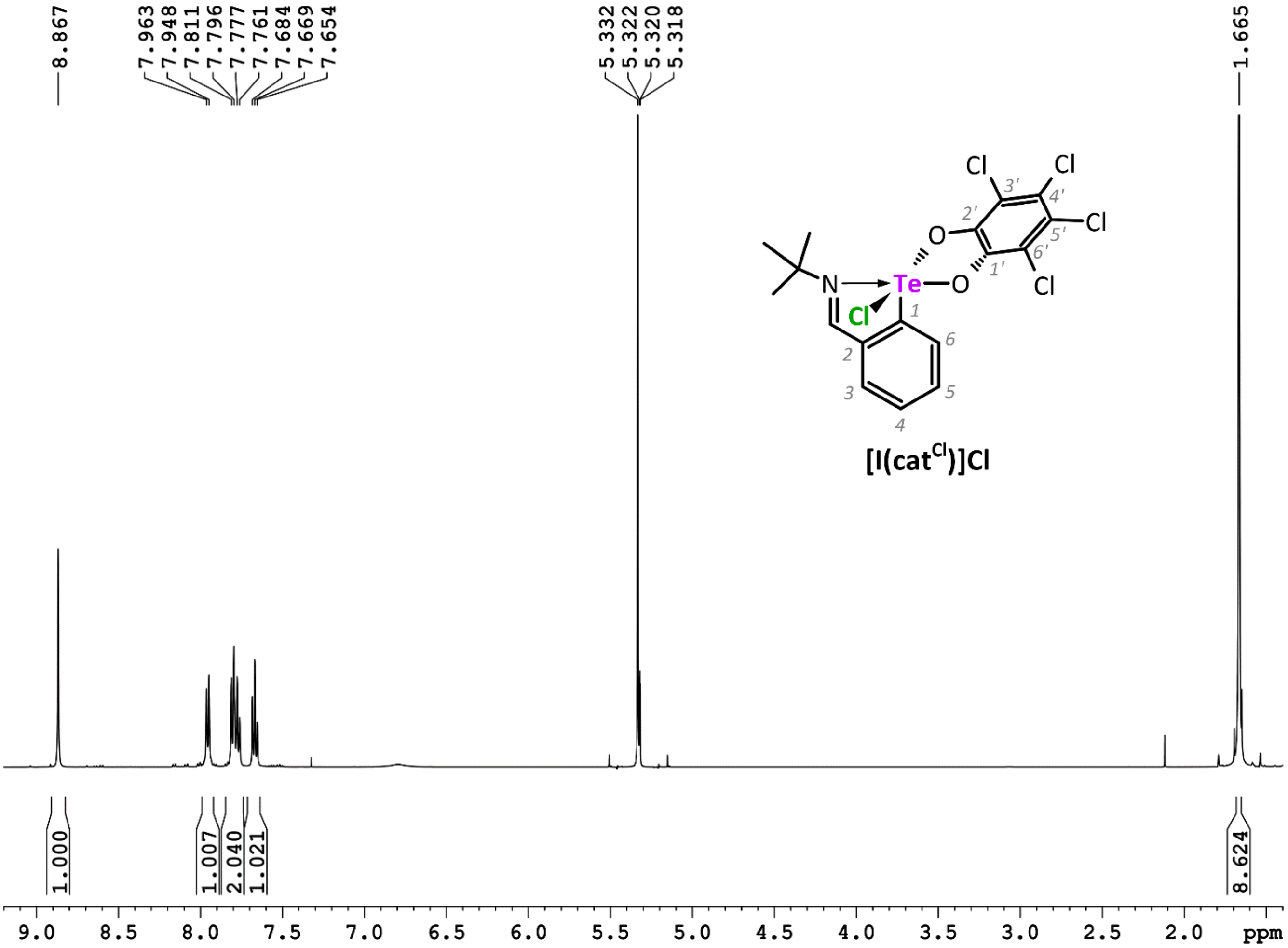

Figure S1: ^{1}**H NMR** spectrum of **[I(cat^{Cl})]Cl** in DCM-d_2 (500 MHz, 295 K).

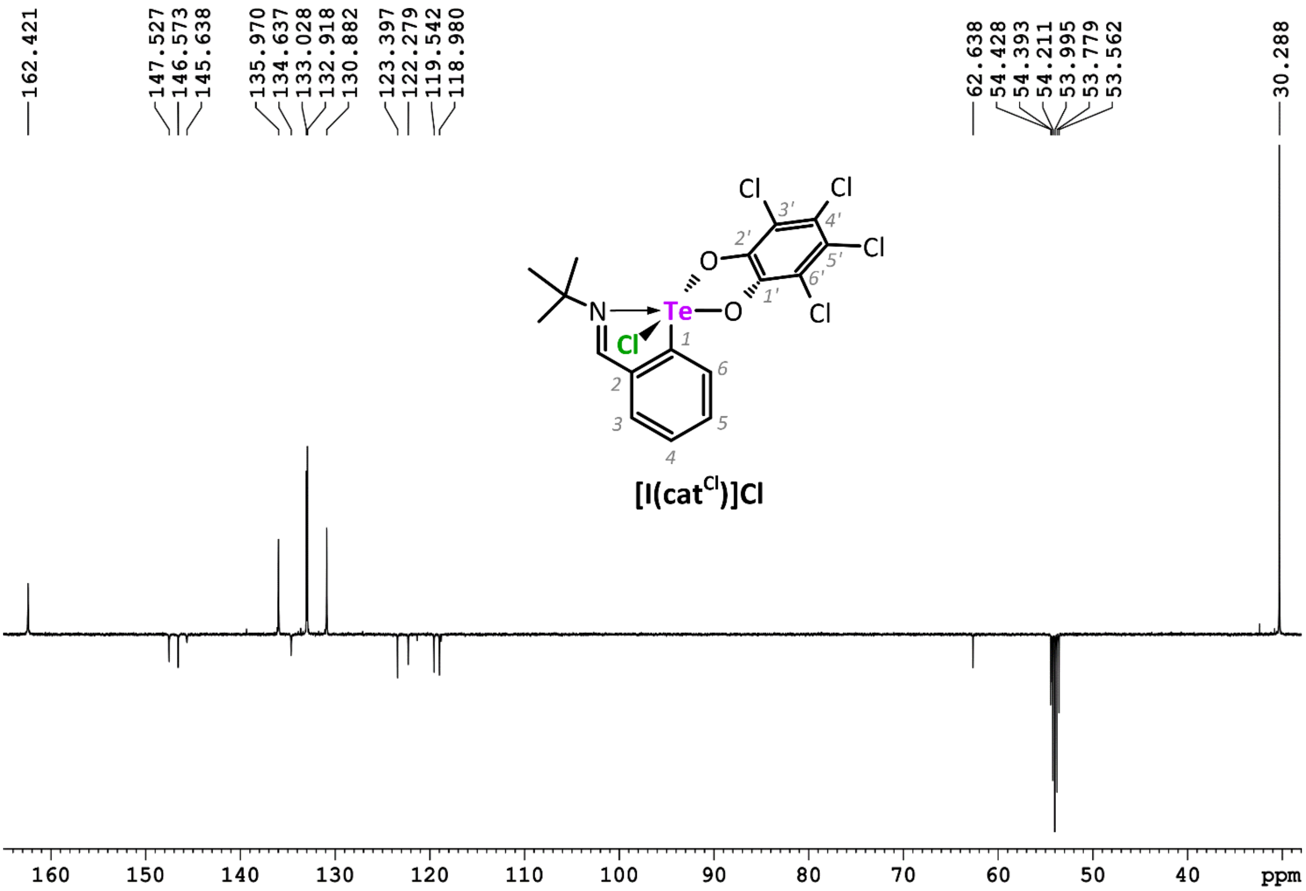

Figure S2: $^{13}C\{^{1}H\}$ **APT NMR** spectrum of **[I(cat^{Cl})]Cl** in DCM-d_2 (125.78 MHz, 295 K).

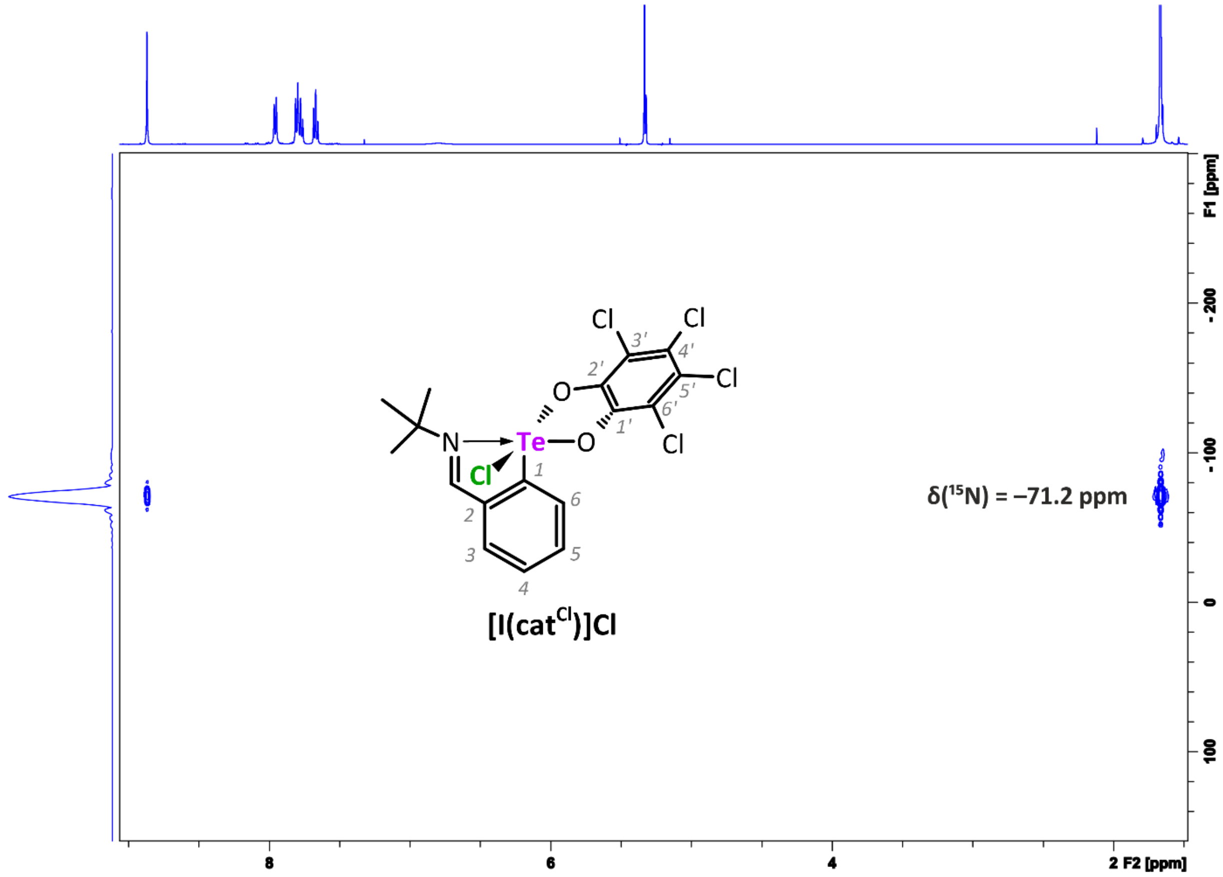

Figure S3: ^{1}H-^{15}N **HMBC NMR** spectrum of **[I(cat^{Cl})]Cl** in DCM-d_2 (500 MHz, 295 K, cnst13 = 5 Hz).

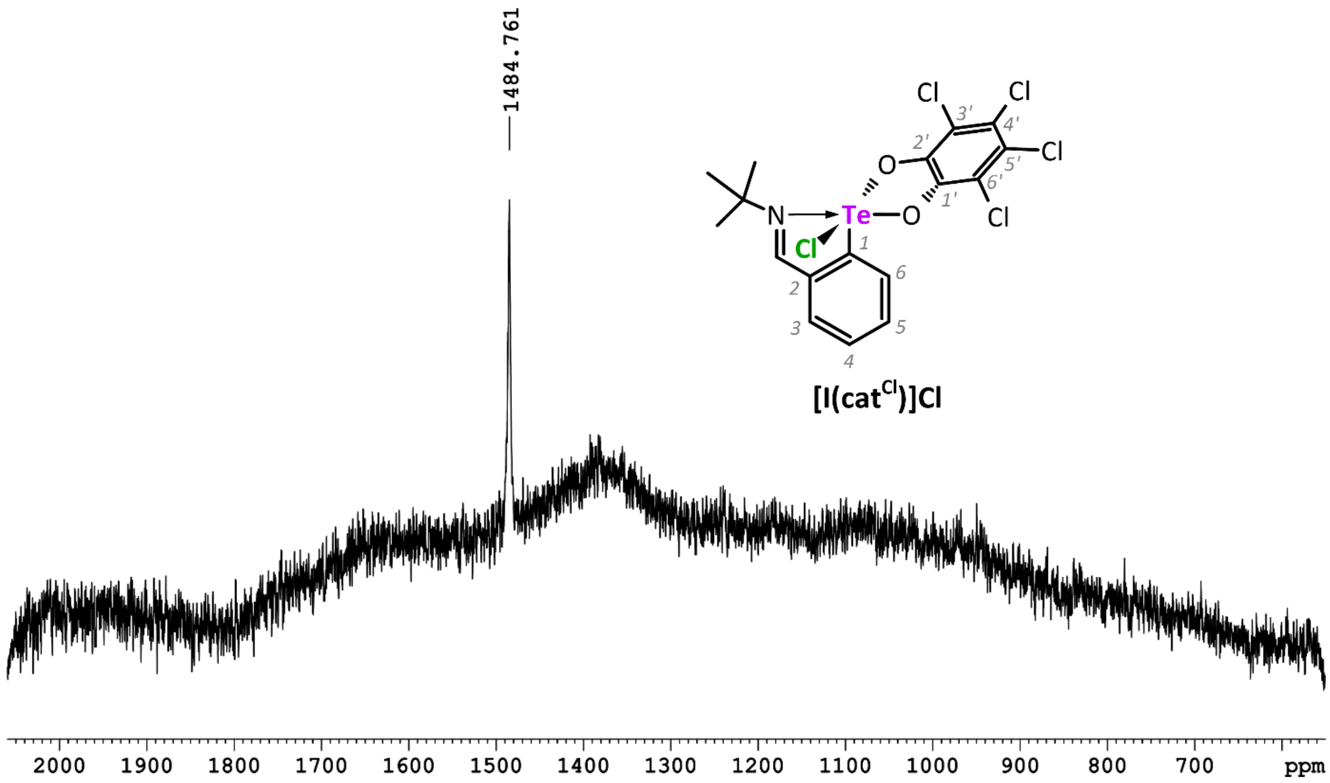

Figure S4: ^{125}Te{^{1}H} NMR spectrum of **[I(cat^{Cl})]Cl** in DCM-d_2 (157.7 MHz, 295 K, NS = 1908).

NMR data for **[I(cat^{Cl})]Cl** *in* <u>*$CDCl_3$:*</u>

^{1}H NMR (500 MHz, $CDCl_3$) δ (ppm): 1.67 [9H, s, (CH_3)$_3$C-]; 7.61 [1H, t, Ar(C)*H*]; 7.72 [1H, d, Ar(C3/C6)*H*]; 7.75 [1H, t, Ar(C)*H*]; 8.00 [1H, d, Ar(C6/C3)*H*]; 8.78 [1H, s, C*H*=N, $^{1}J(^{13}C,^{1}H)$ = 170.5 Hz)]. **^{13}C{^{1}H} NMR** (125.78 MHz, $CDCl_3$) δ (ppm): 30.2 [s, (*C*H_3)$_3$C-]; 62.0 [qC, s, (CH_3)$_3$*C*-]; 118.7 [s, qC, Ar(*C*)]; 119.3 [s, qC, Ar(*C*)]; 121.9 [s, qC, Ar(*C*)]; 123.4 [s, qC, Ar(*C*)]; 130.5 [s, Ar(*C*)H]; 132.3 [s, Ar(*C*)H]; 132.3 [s, Ar(*C*)H]; 133.8 [s, qC, Ar(*C*)]; 135.7 [s, Ar(*C*)H]; 145.8 [s, qC, Ar(*C*)]; 147.5 [s, qC, Ar(*C*)]; 161.2 [s, *C*H=N]. **^{125}Te{^{1}H} NMR** (157.79 MHz, $CDCl_3$) δ: 1474.9 ppm.

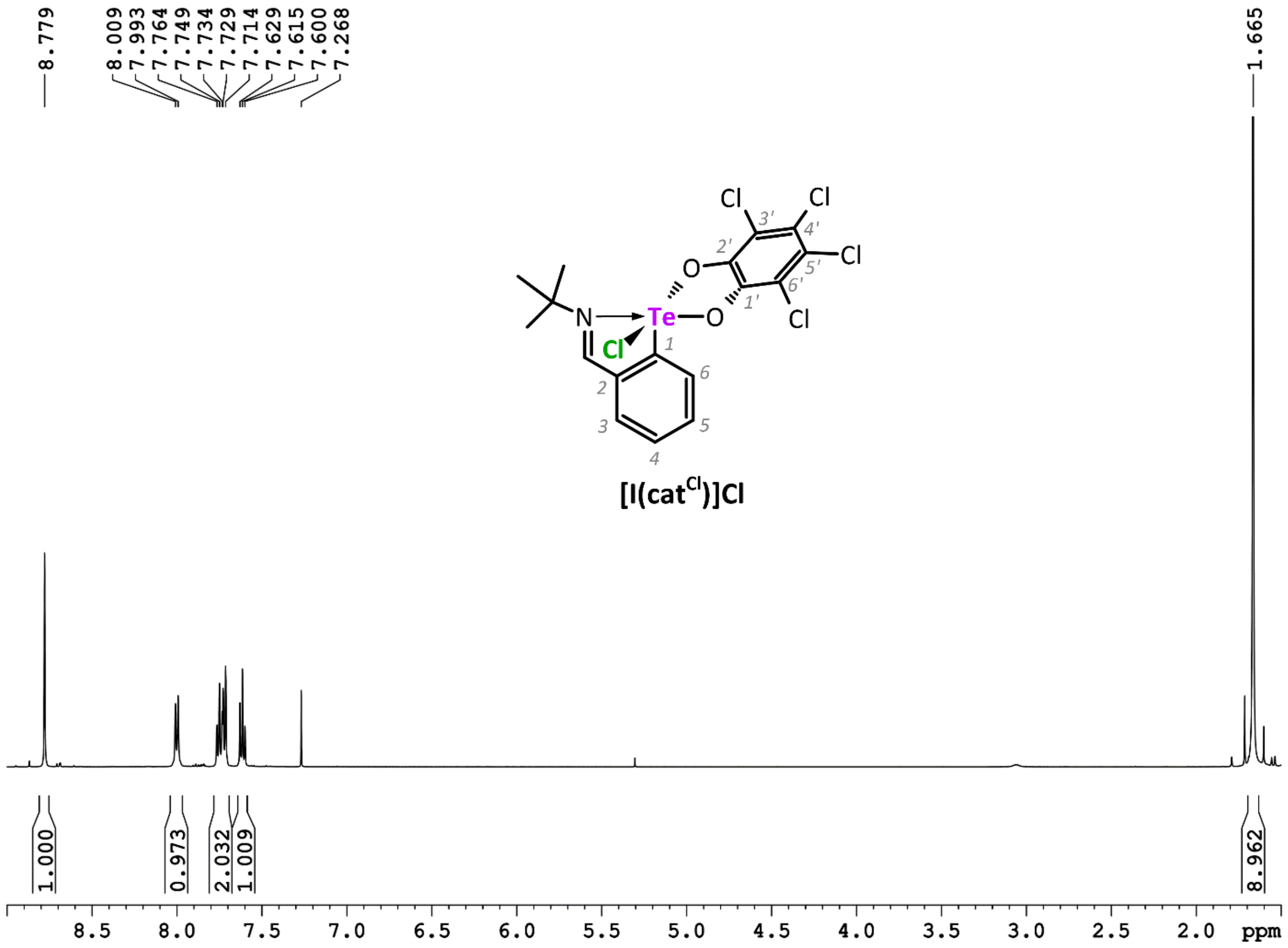

Figure S5: ^{1}**H NMR** spectrum of **[I(cat^{Cl})]Cl** in $CDCl_3$ (500 MHz, 295 K).

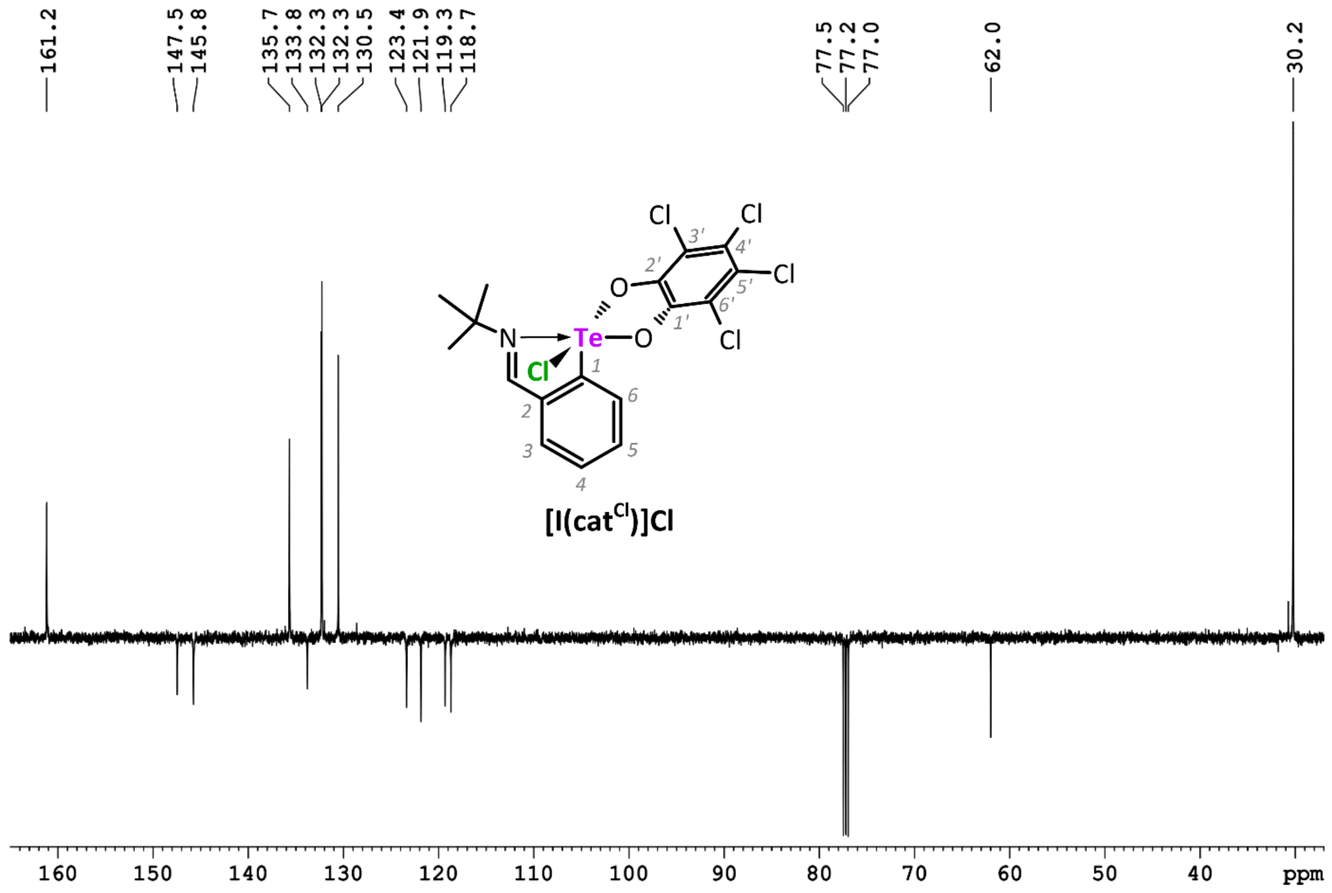

Figure S6: ^{13}**C{^{1}H} APT NMR** spectrum of **[I(cat^{Cl})]Cl** in $CDCl_3$ (125.78 MHz, 295 K).

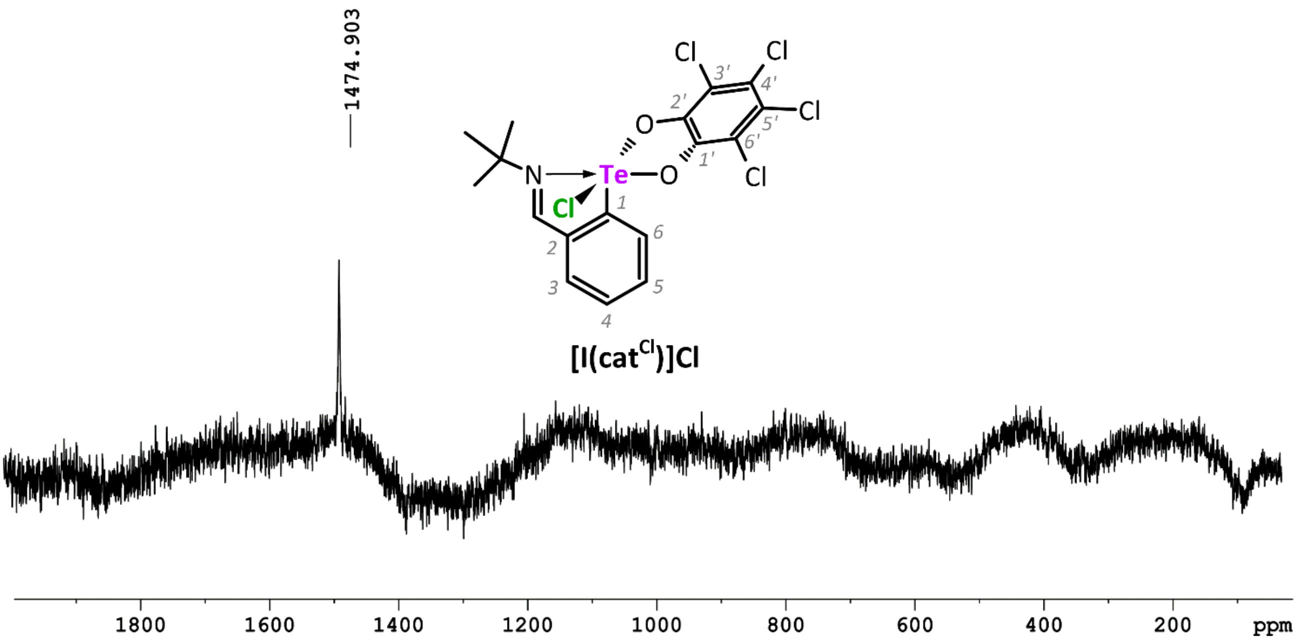

Figure S7: **^{125}Te{^{1}H} NMR** spectrum of **[I(cat^{Cl})]Cl** in $CDCl_3$ (157.7 MHz, 295 K, NS = 1024).

NMR spectra of [I(cat^{Cl})][OTf]

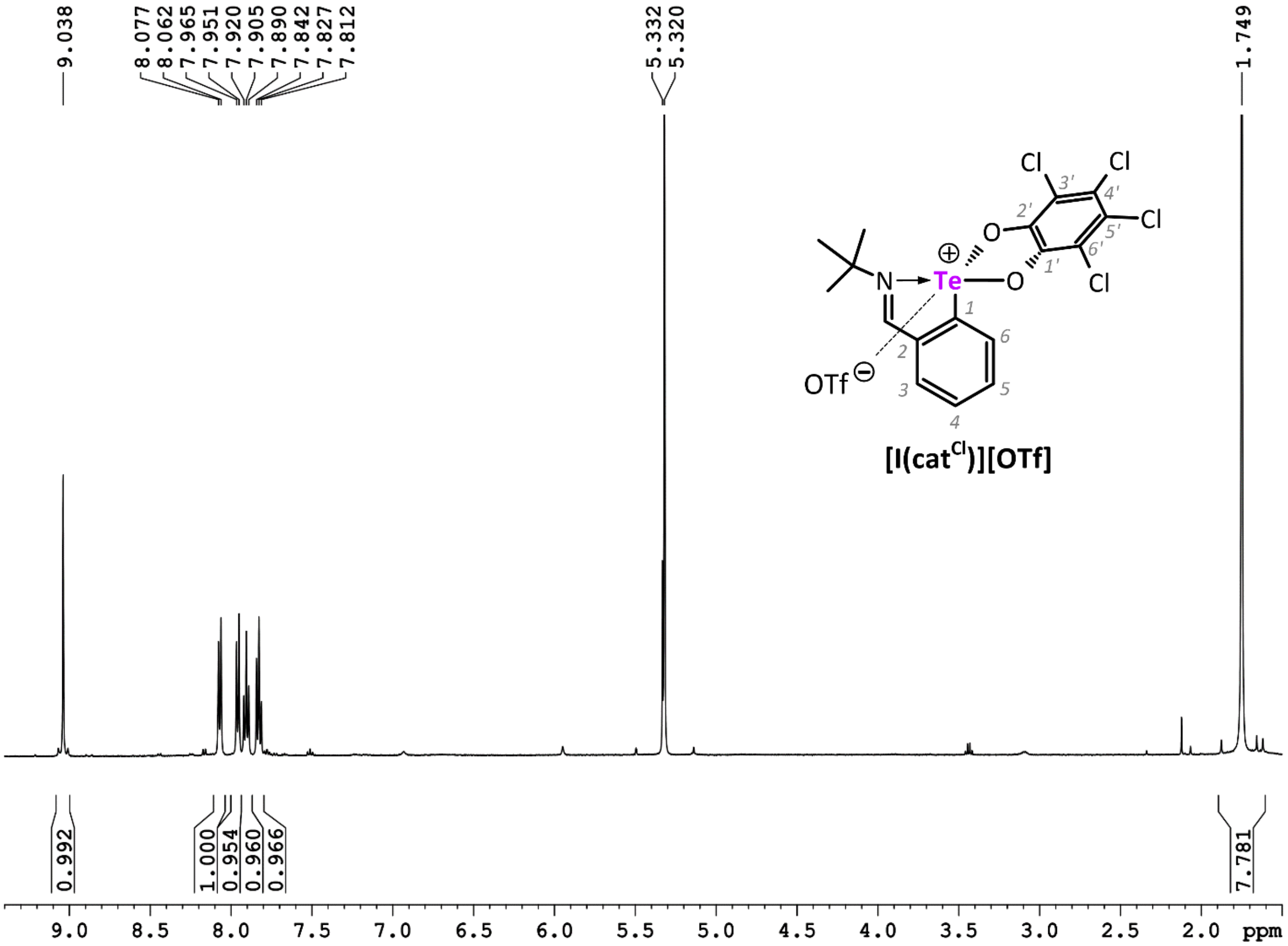

Figure S8: **^{1}H NMR** spectrum of **[I(cat^{Cl})][OTf]** in DCM-d_2 (500 MHz, 295 K).

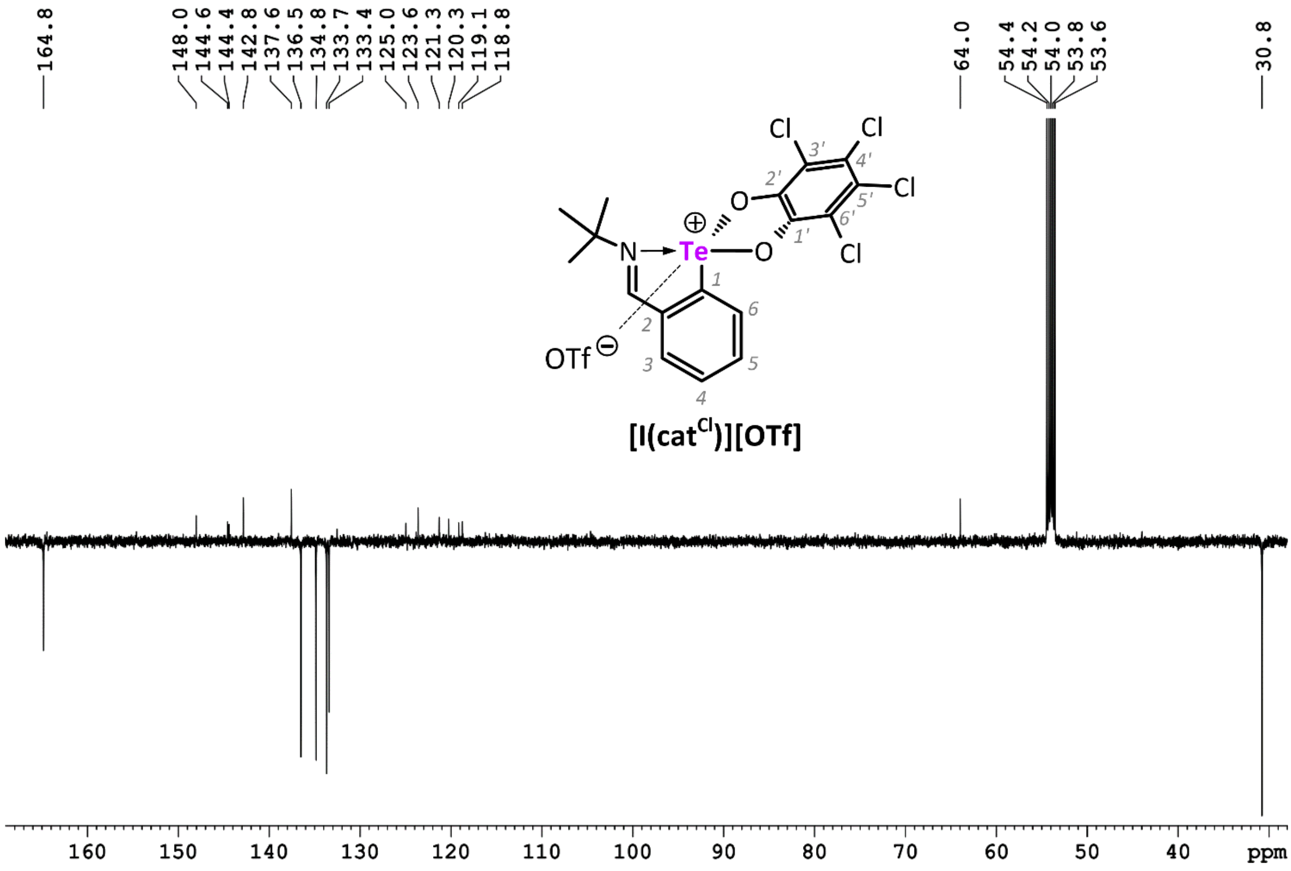

Figure S9: $^{13}C\{^{1}H\}$ **APT NMR** spectrum of **[I(cat^{Cl})][OTf]** in DCM-d_2 (125.78 MHz, 295 K).

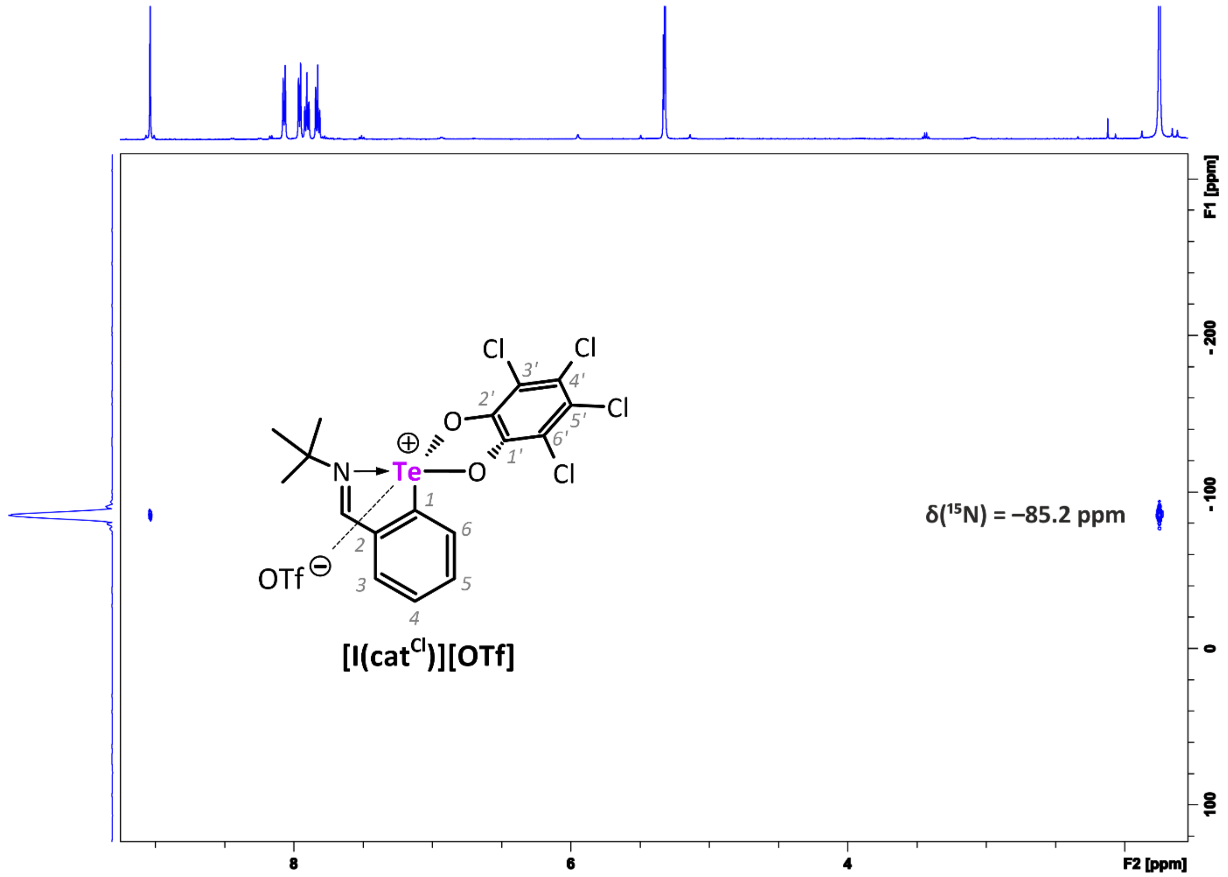

Figure S10: ^{1}H-^{15}N **HMBC NMR** spectrum of **[I(cat^{Cl})][OTf]** in DCM-d_2 (500 MHz, 295 K, cnst13 = 5 Hz).

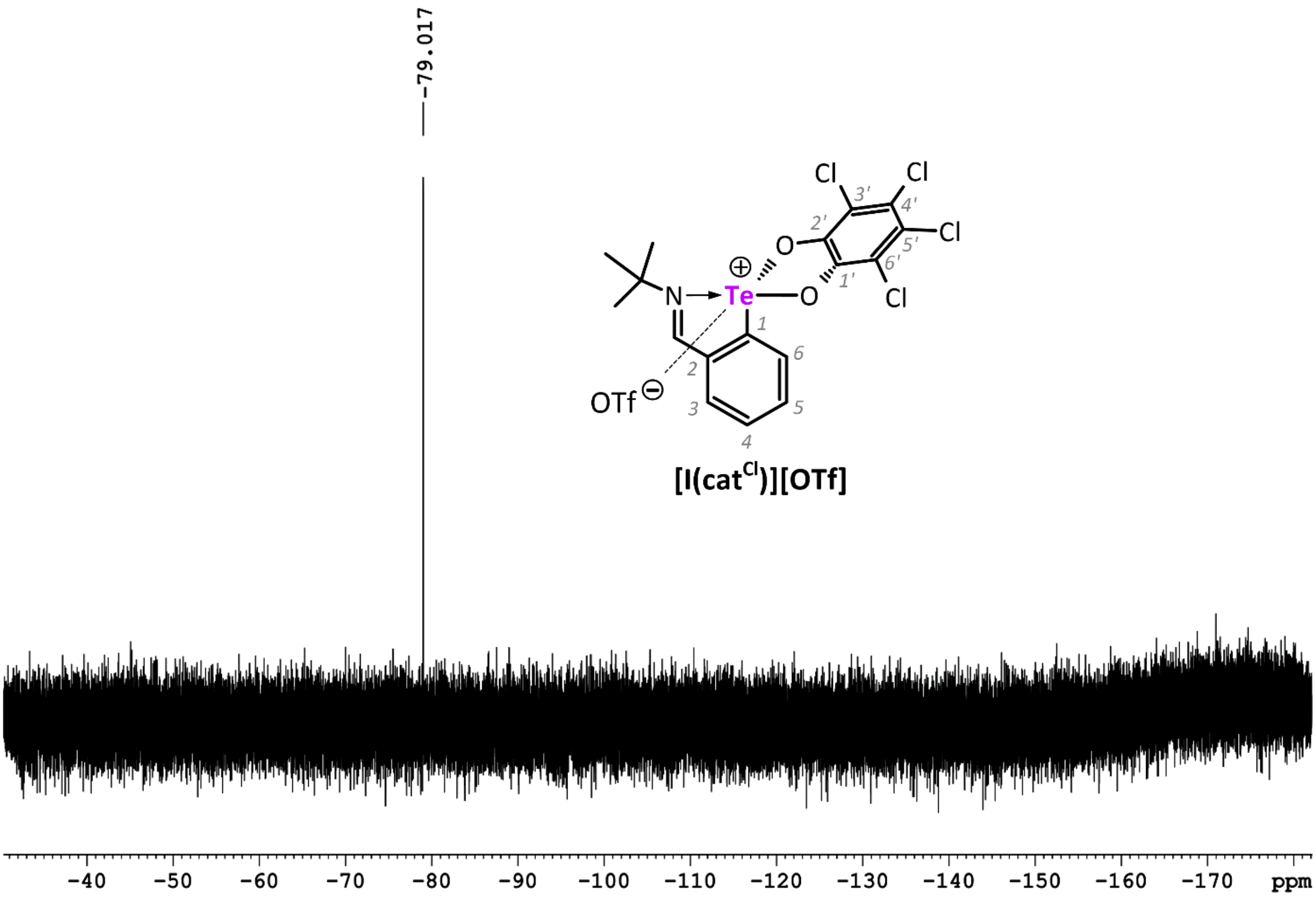

Figure S11: ^{19}F NMR spectrum of **[I(cat^{Cl})][OTf]** in DCM-d_2 (470.66 MHz, 295 K).

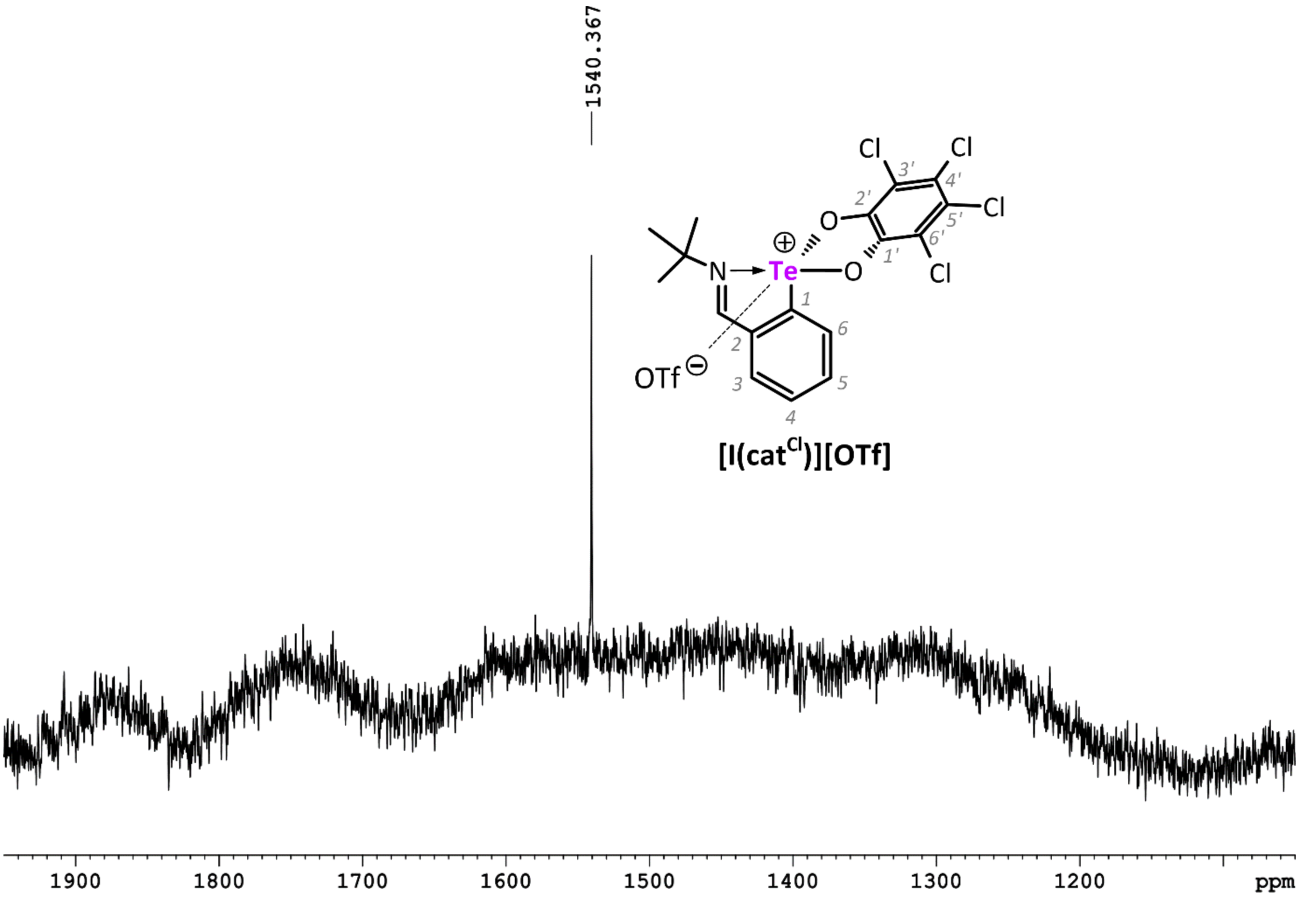

Figure S12: ^{125}Te{^{1}H} NMR spectrum of **[I(cat^{Cl})][OTf]** in DCM-d_2 (157.7 MHz, 295 K, NS = 5000).

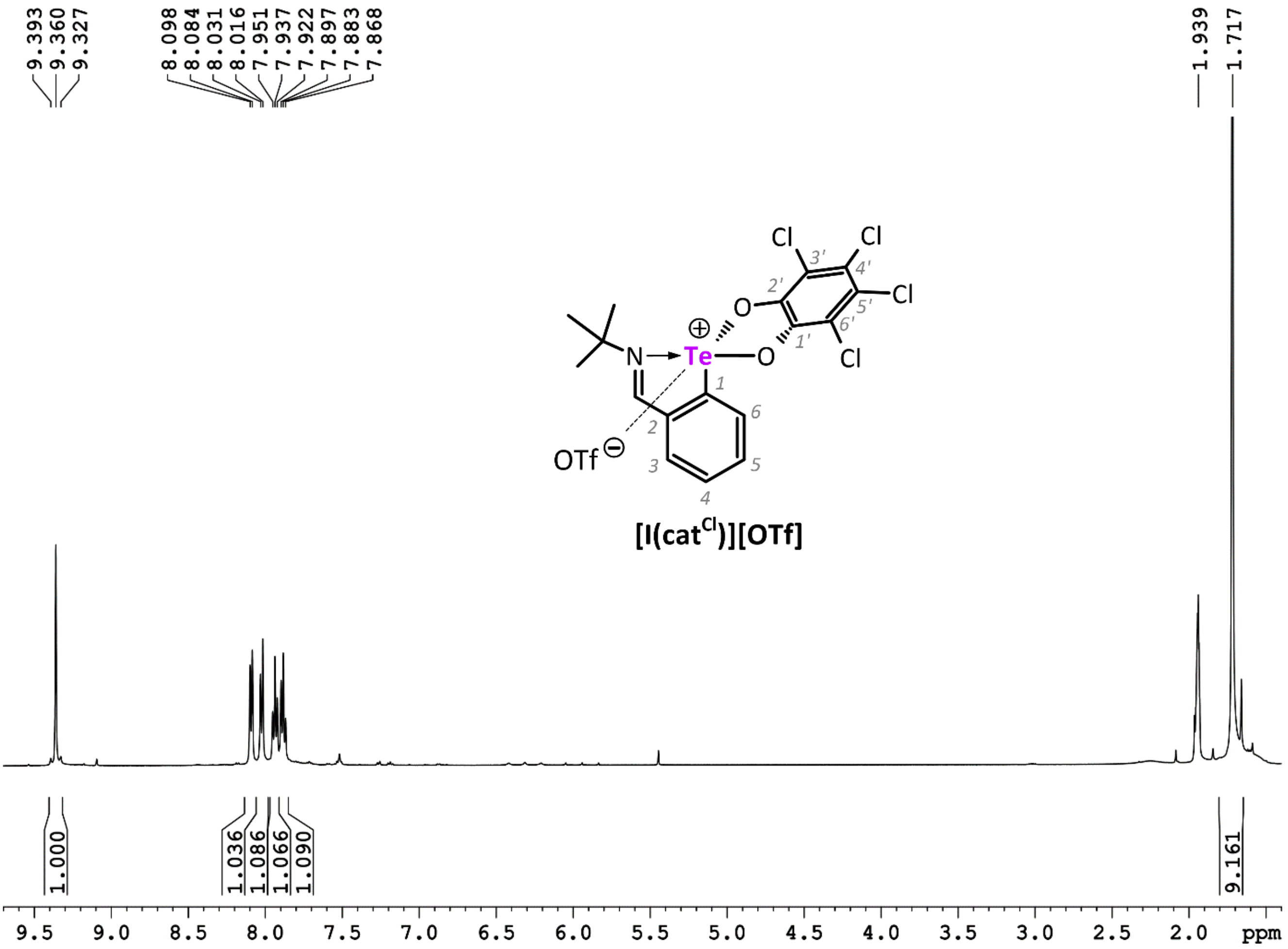

Figure S13: ^{1}H NMR spectrum of **[I(cat^{Cl})][OTf]** in MeCN-d_3 (500 MHz, 295 K).

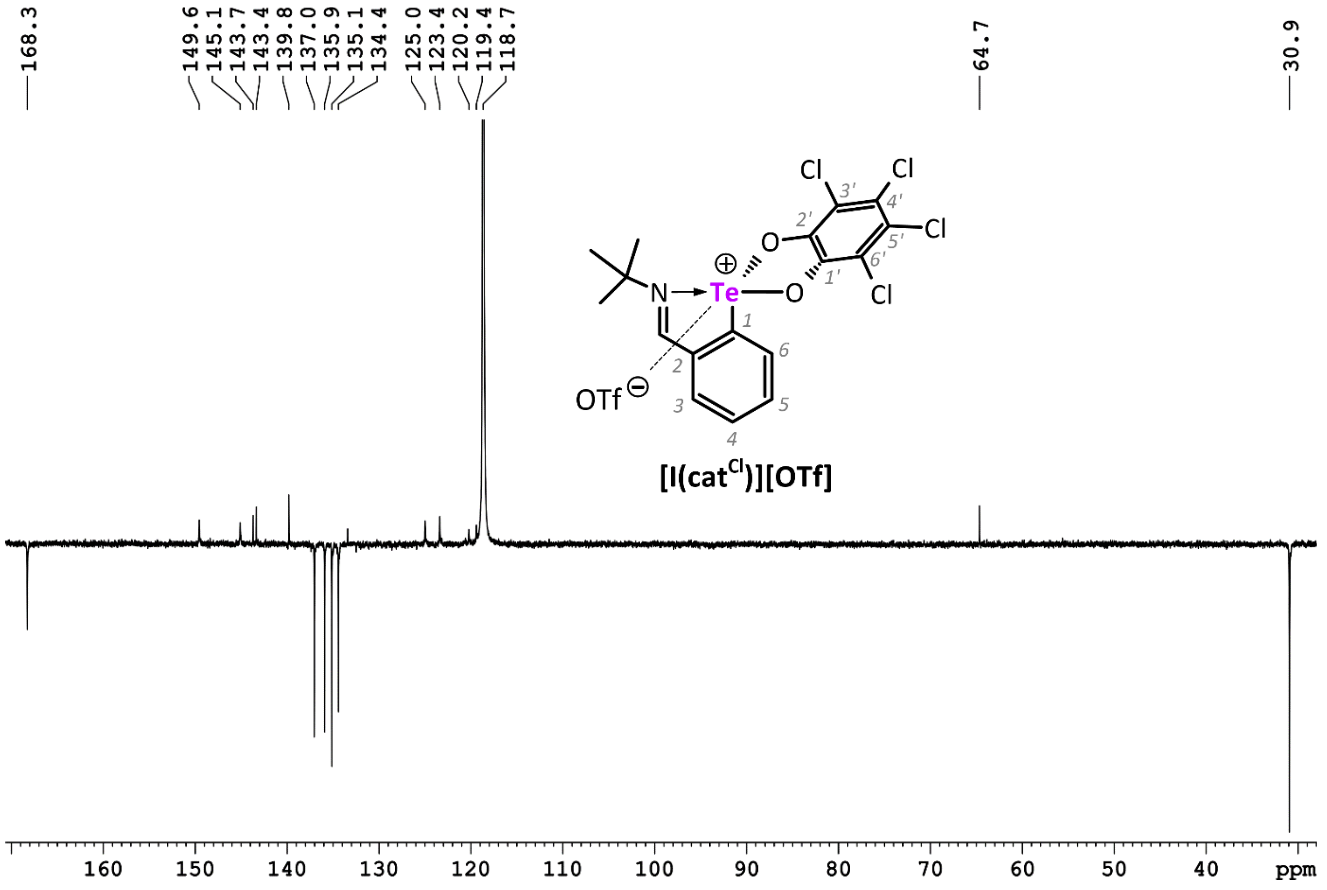

Figure S14: ^{13}C{^{1}H} APT NMR spectrum of **[I(cat^{Cl})][OTf]** in MeCN-d_3 (125.78 MHz, 295 K).

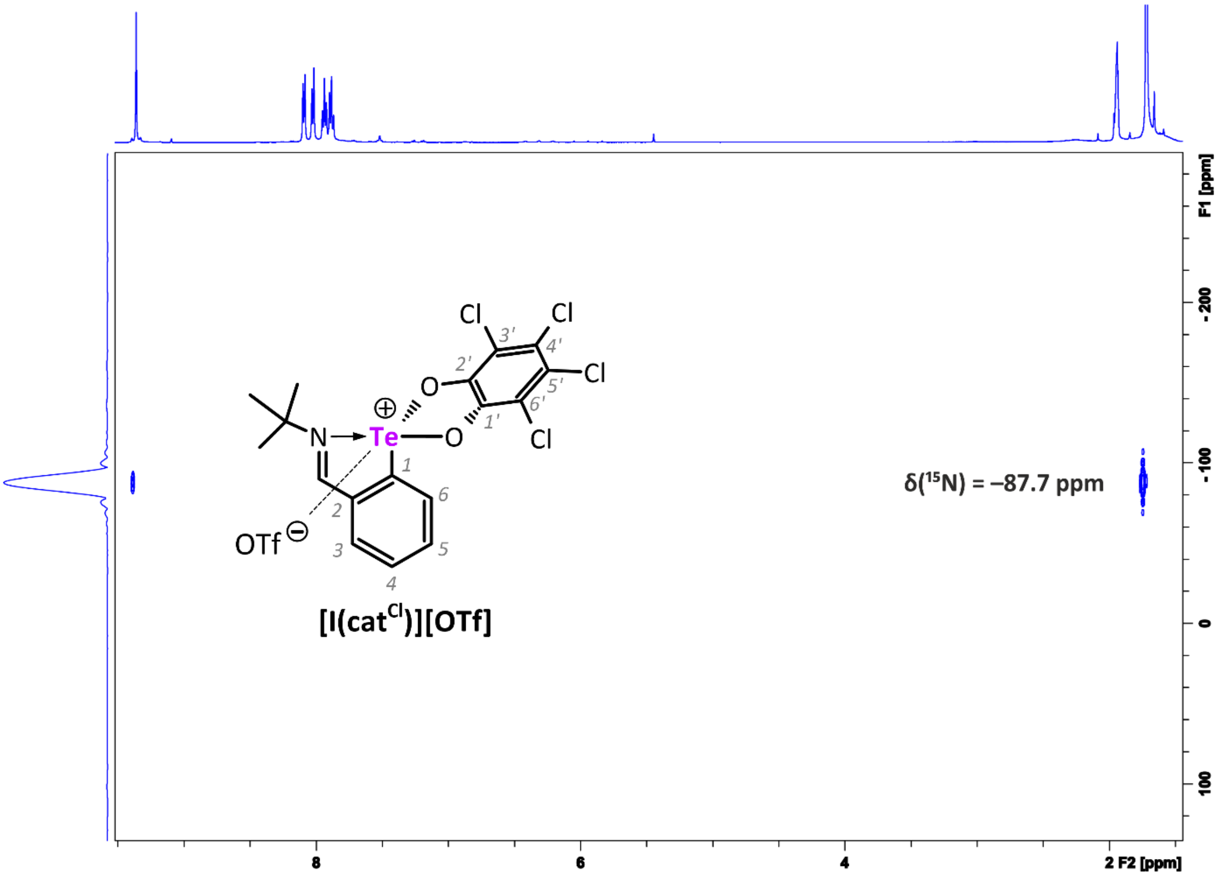

Figure S15: ^{1}**H-**^{15}**N HMBC NMR** spectrum of **[I(cat^{Cl})][OTf]** in MeCN-d_3 (500 MHz, 295 K, cnst13 = 5 Hz).

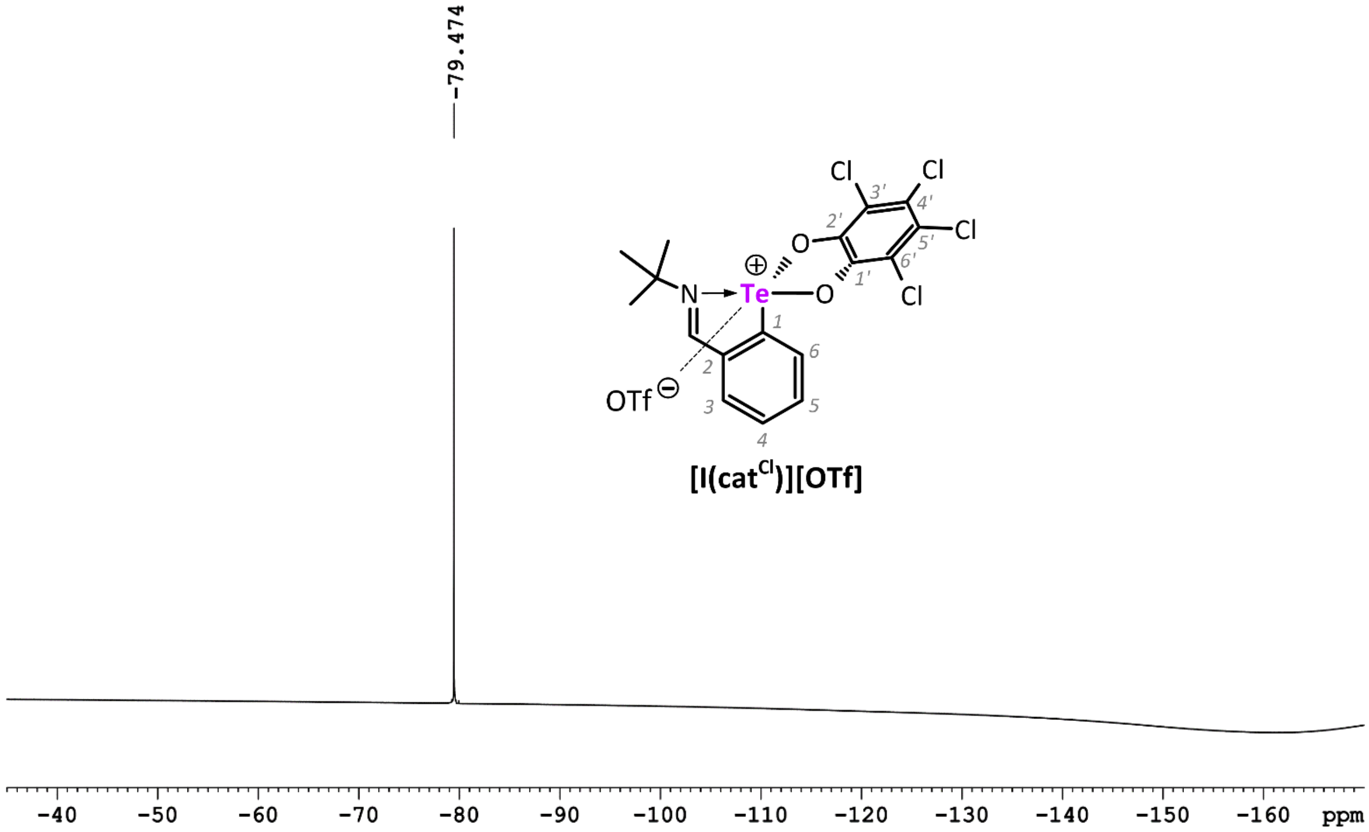

Figure S16: ^{19}**F NMR** spectrum of **[I(cat^{Cl})][OTf]** in MeCN-d_3 (470.66 MHz, 295 K).

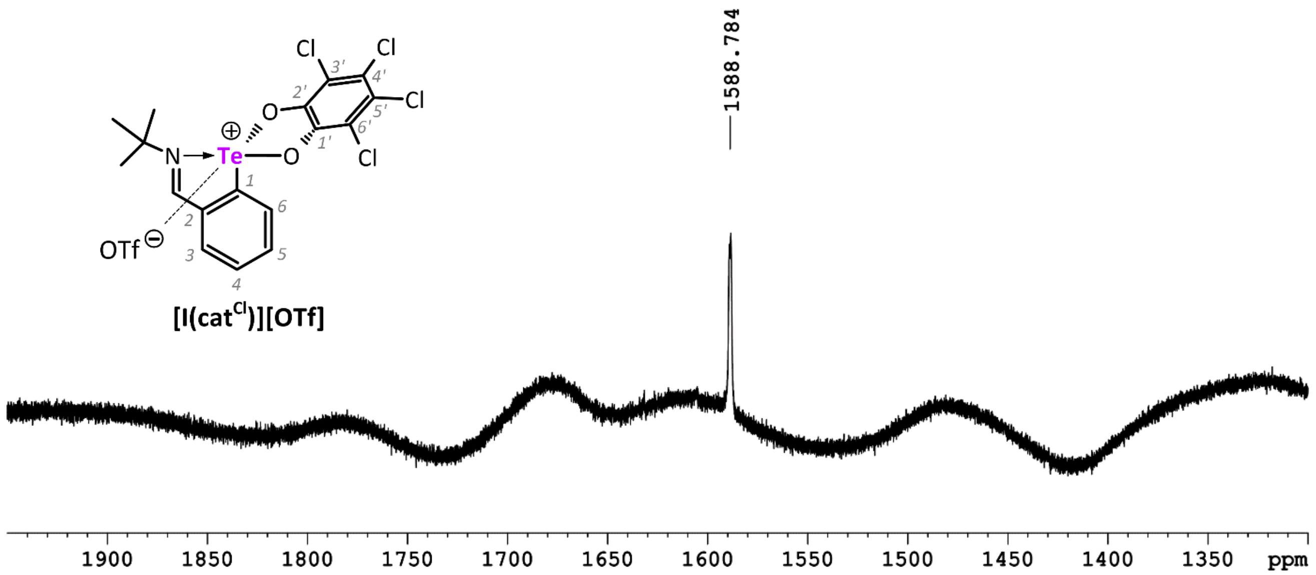

Figure S17: ^{125}**Te{**^{1}**H} NMR** spectrum of **[I(cat^{Cl})][OTf]** in MeCN-d_3 (157.7 MHz, 295 K, NS = 102400).

NMR spectra of [I(cat^{Cl})][SbF$_6$]

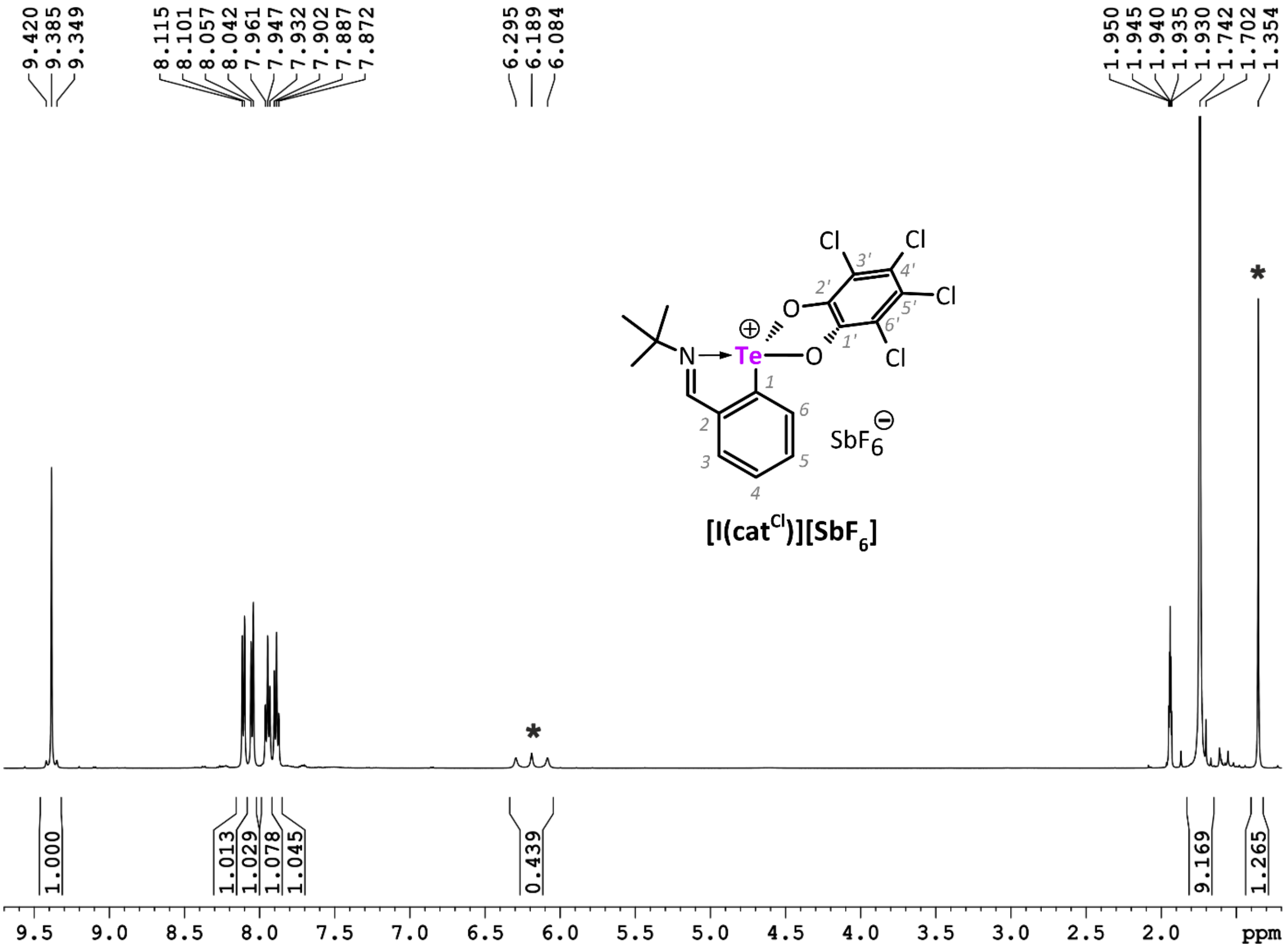

Figure S18: ^{1}**H NMR** spectrum of **[I(cat^{Cl})][SbF$_6$]** in MeCN-d_3 (500 MHz, 295 K). * denotes minor amount of *t*BuNH$_3^+$SbF$_6^-$ formed by partial hydrolysis despite by our best efforts.

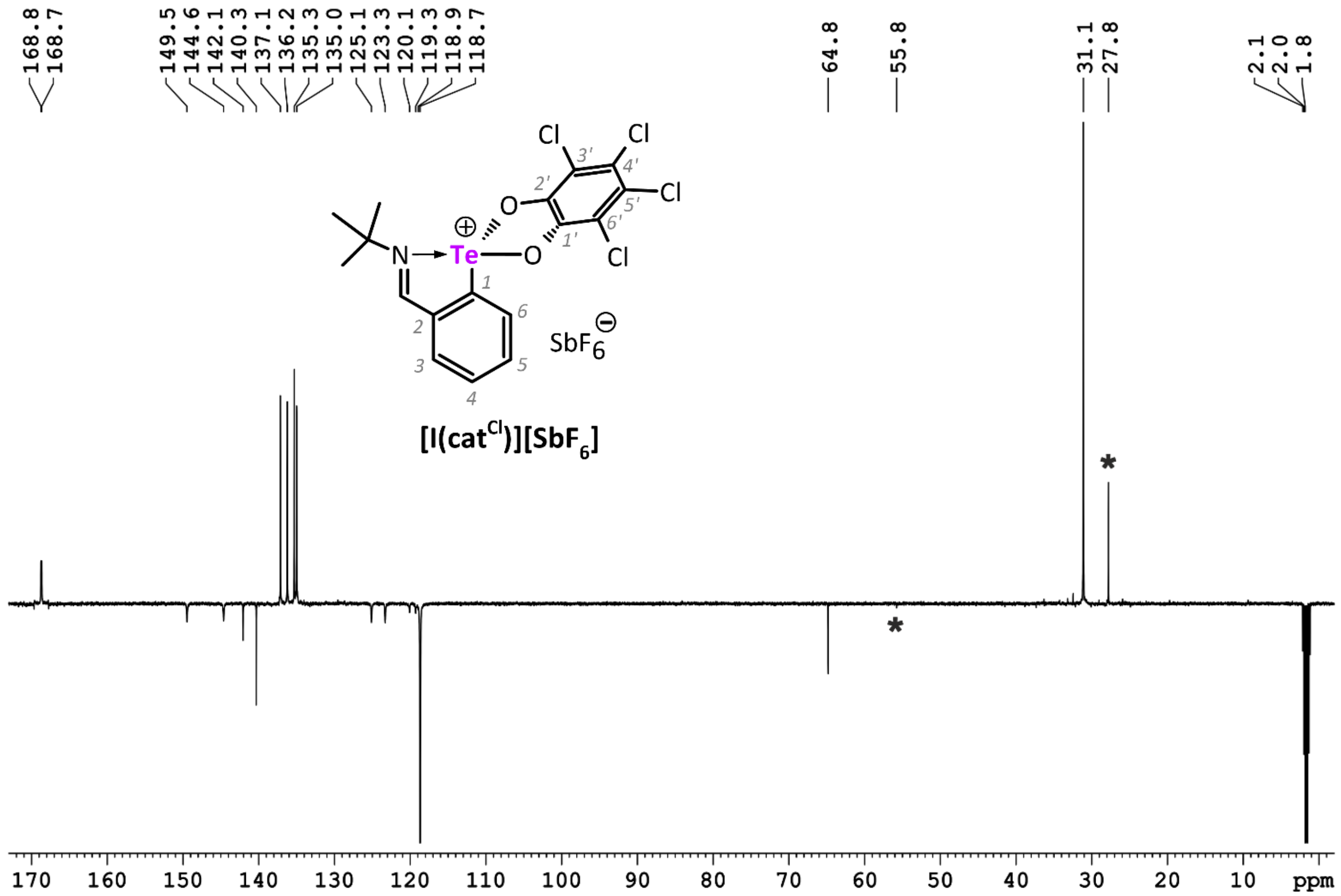

Figure S19: $^{13}C\{^1H\}$ APT NMR spectrum of **[I(cat^{Cl})][SbF$_6$]** in MeCN-d_3 (125.78 MHz, 295 K). * denotes minor amount of tBuNH$_3^+$SbF$_6^-$ formed by partial hydrolysis despite by our best efforts.

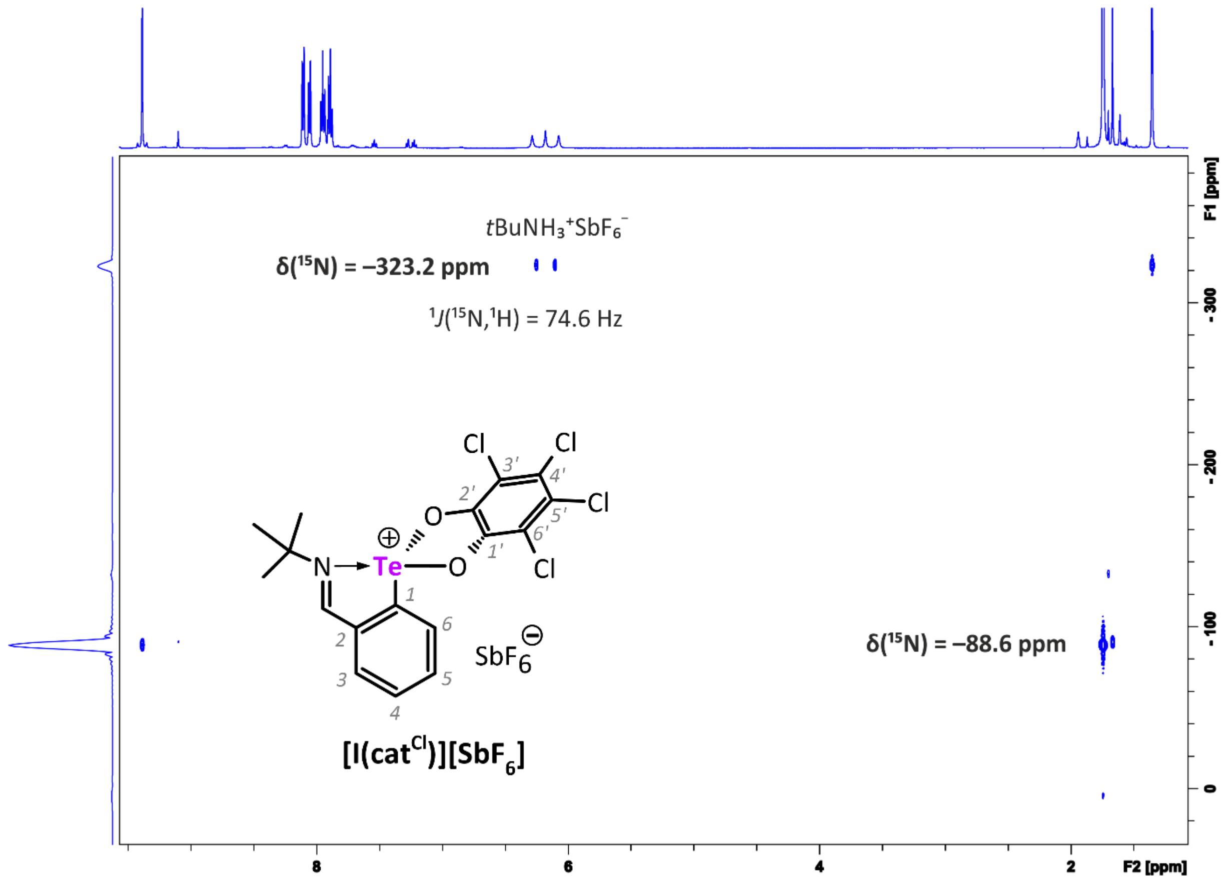

Figure S20: ^{1}H-^{15}N HMBC NMR spectrum of **[I(cat^{Cl})][SbF$_6$]** in MeCN-d_3 (500 MHz, 295 K, cnst13 = 5 Hz). Spectrum contains also signal of tBuNH$_3^+$SbF$_6^-$ formed by hydrolysis despite by our best efforts.

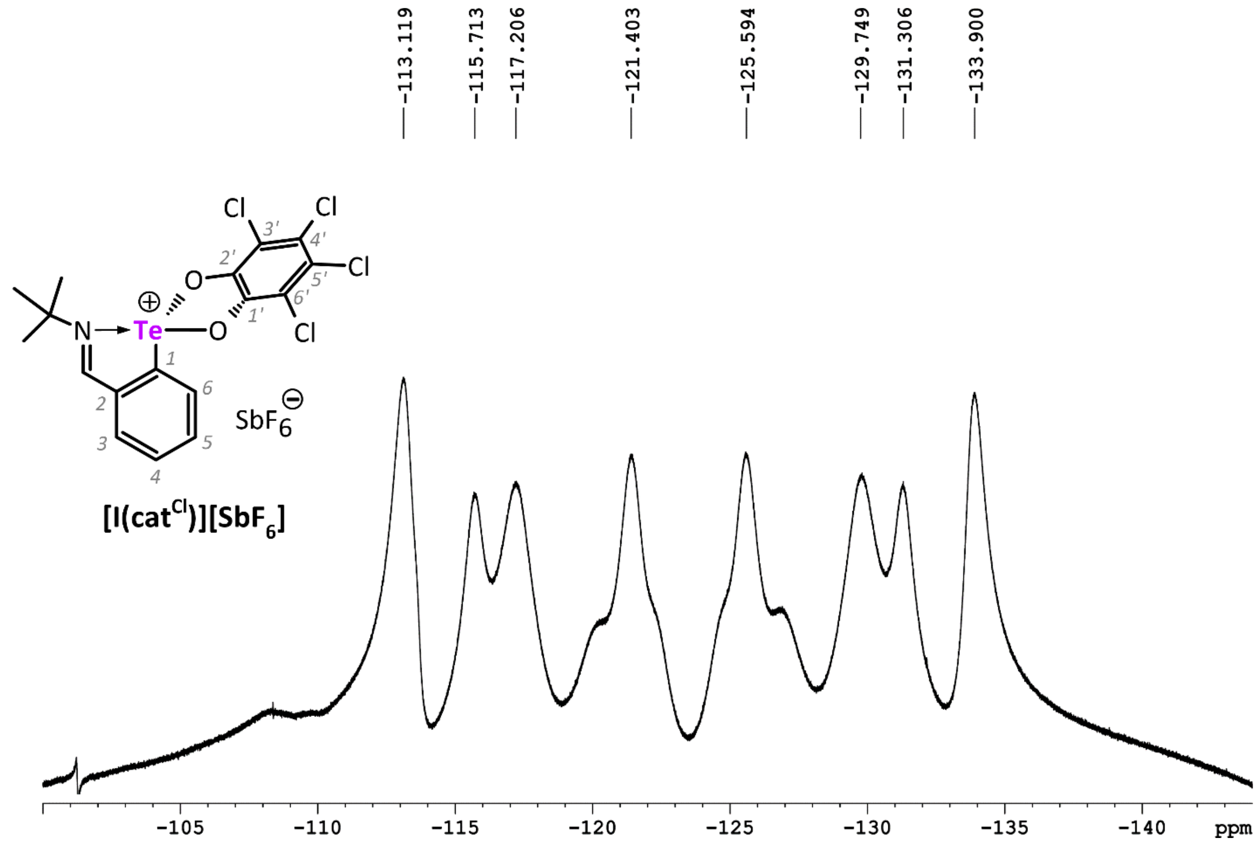

Figure S21: ^{19}**F NMR** spectrum of **[I(cat^{Cl})][SbF$_6$]** in MeCN-d_3 (470.66 MHz, 295 K).

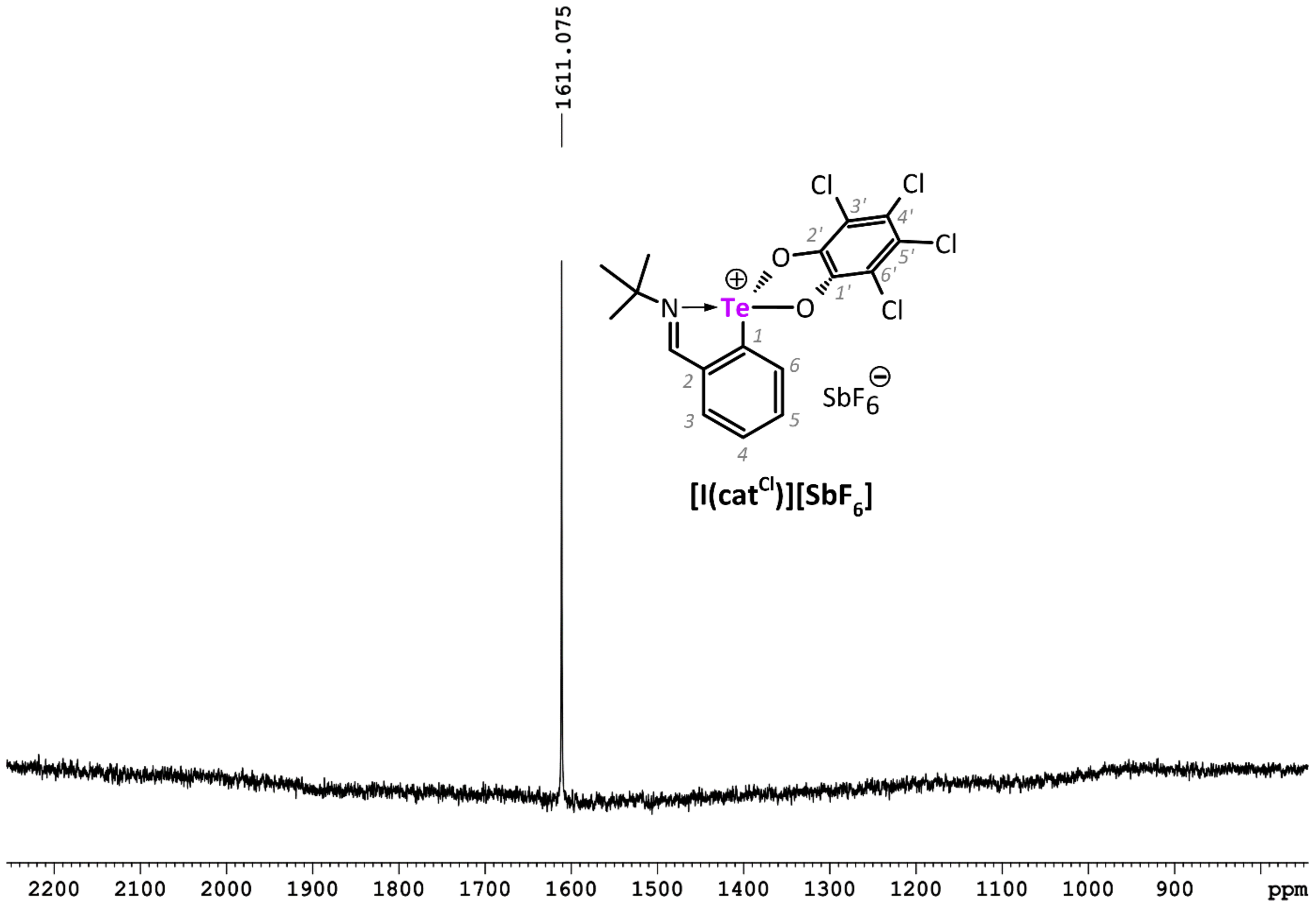

Figure S22: ^{125}**Te{^{1}H} NMR** spectrum of **[I(cat^{Cl})][SbF$_6$]** in MeCN-d_3 (157.7 MHz, 295 K, NS = 925).

NMR spectra proving reversibility in the formation of a/b-[I(cat^{tBu})]Cl

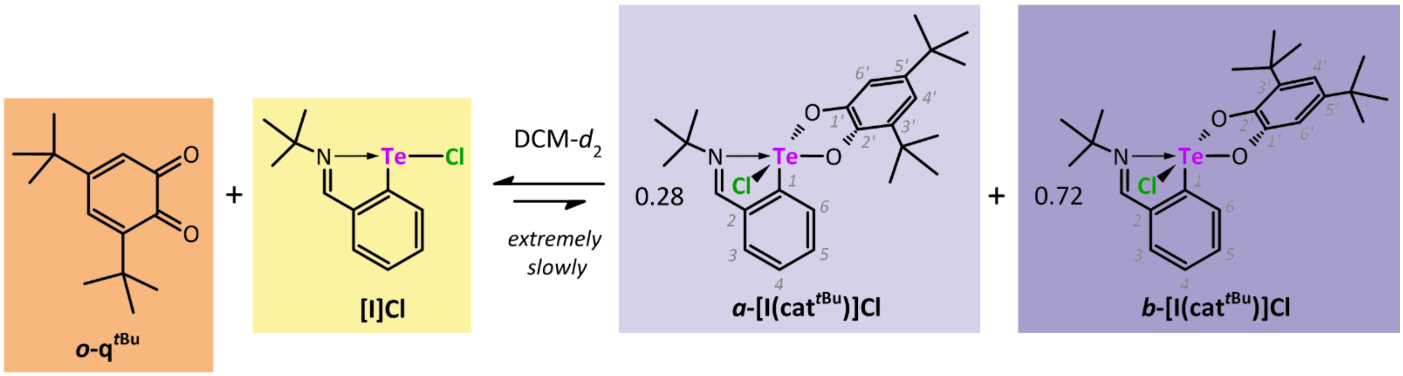

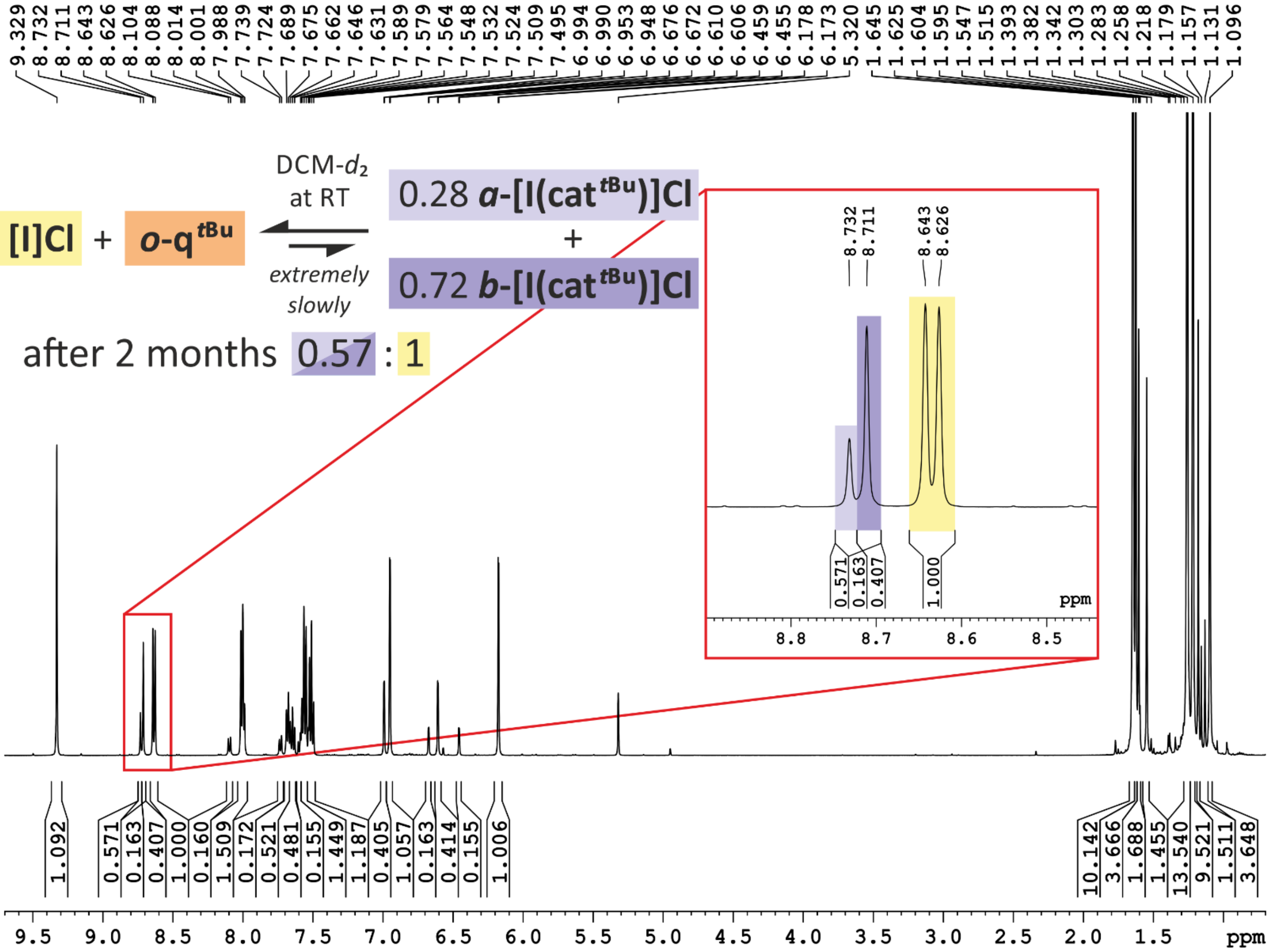

Figure S23: **^{1}H NMR** spectrum of mixture of two isomers ***a*/*b*-[I(cat^{tBu})]Cl** after 2 months of being in a dynamic equilibrium with starting **[I]Cl** and ***o*-q^{tBu}** (500.20 MHz, 295 K, DCM-d_2).

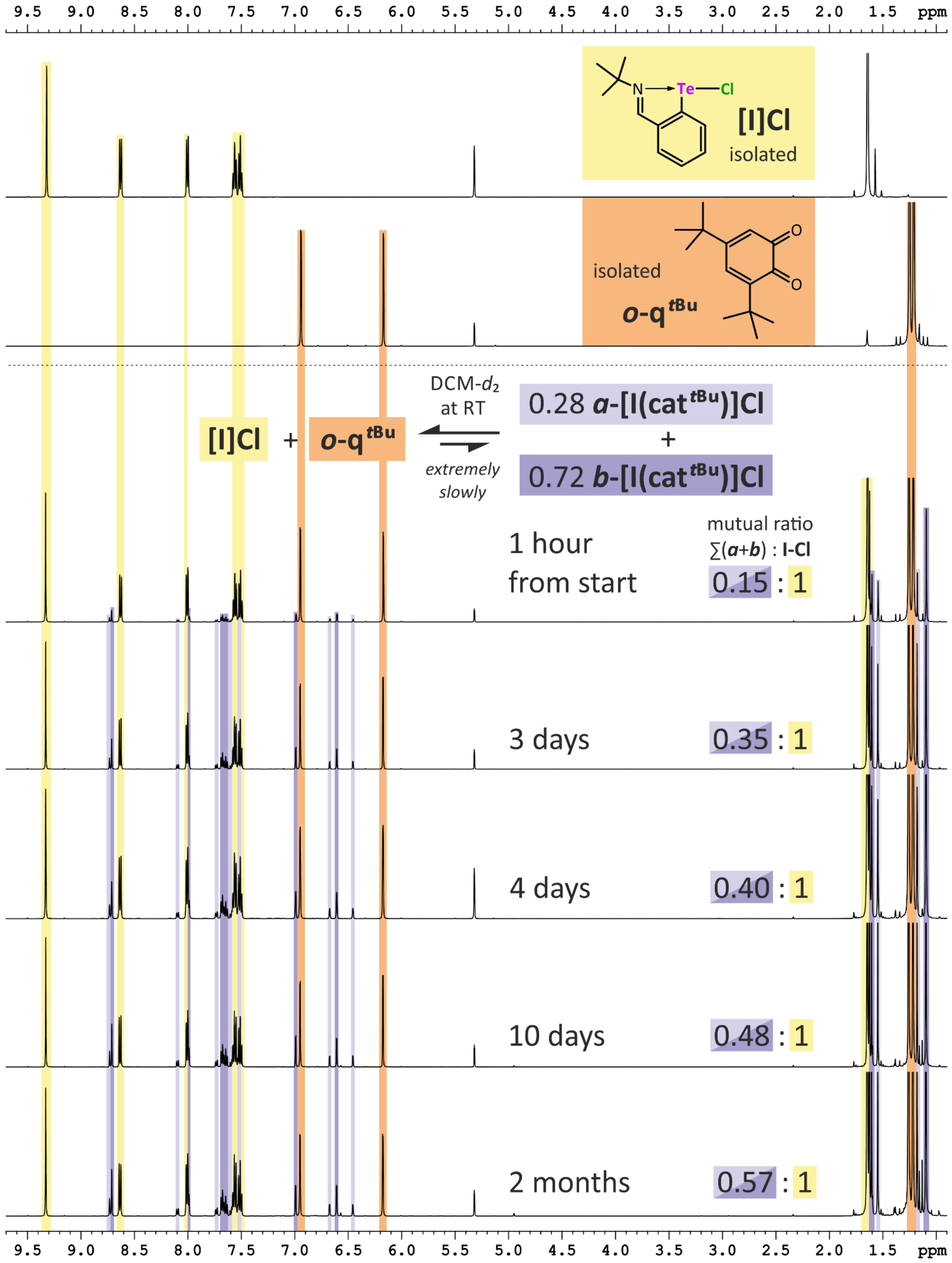

Figure S24: Time evolution of [1]**H NMR** spectra of the reaction between **[I]Cl** and ***o*-q*^tBu*** in 1:1 molar ratio in DCM-d_2 resulting in extreme slow formation of two isomers ***a*/*b*-[I(cat*^tBu*)]Cl** being in a dynamic equilibrium with starting **[I]Cl** and ***o*-q*^tBu*** (500.20 MHz, 295 K). The ratio of 0.57:1 for ***a*/*b*-[I(cat*^tBu*)]Cl** (after 2 months) turned out to be the final ratio, as any prolonged staying did not change it.

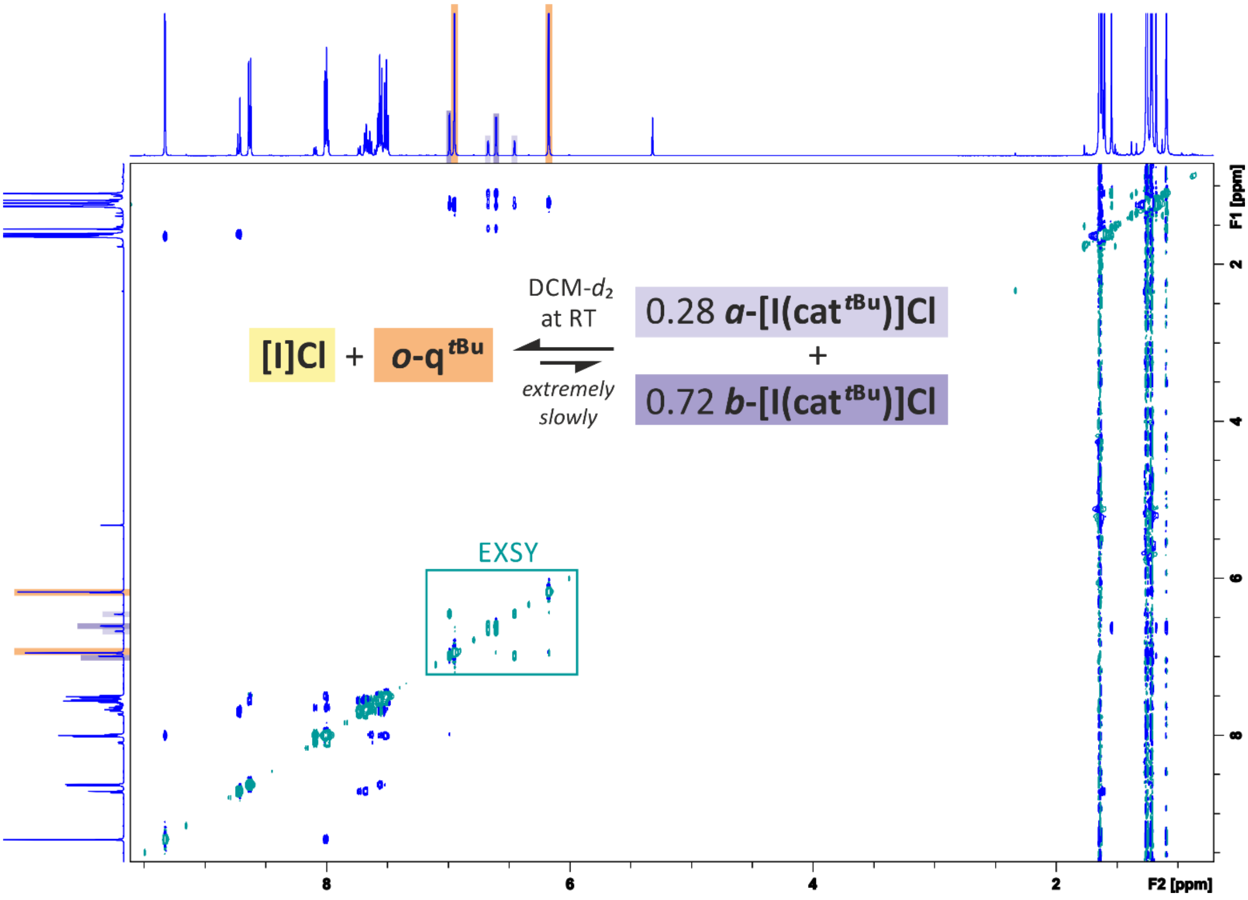

Figure S25: **^{1}H-^{1}H NOESY NMR** spectrum of mixture of two isomers ***a*/*b*-[I(cat^{tBu})]Cl** after 2 months of being in a dynamic equilibrium with starting **[I]Cl** and ***o*-q^{tBu}** (500.20 MHz, 295 K, DCM-d_2, d8 = 2 s).

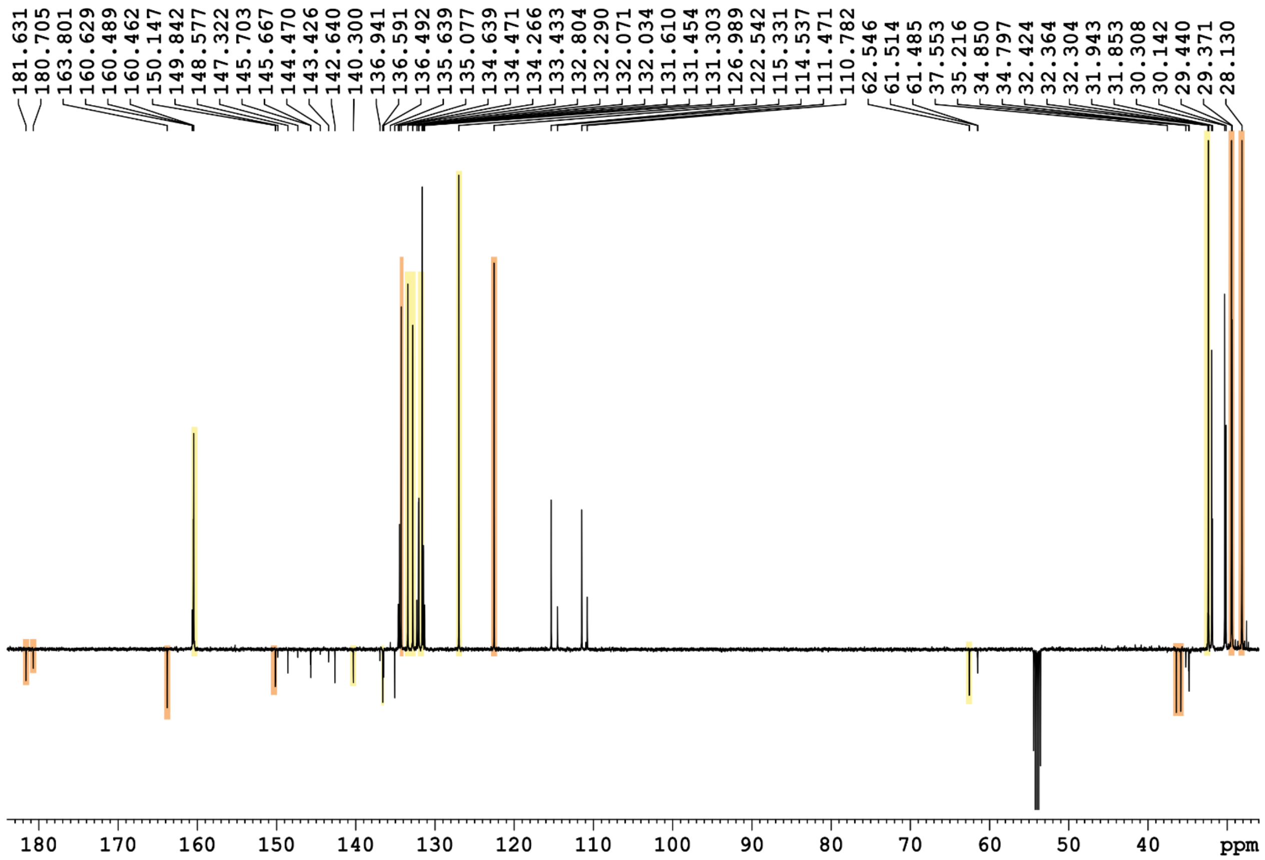

Figure S26: $^{13}C\{^{1}H\}$ **APT NMR** spectrum of mixture of two isomers ***a*/*b*-[I(cat^{tBu})]Cl** after 2 months of being in a dynamic equilibrium with starting **[I]Cl** and ***o*-q^{tBu}** (125.78 MHz, 295 K, DCM-d_2).

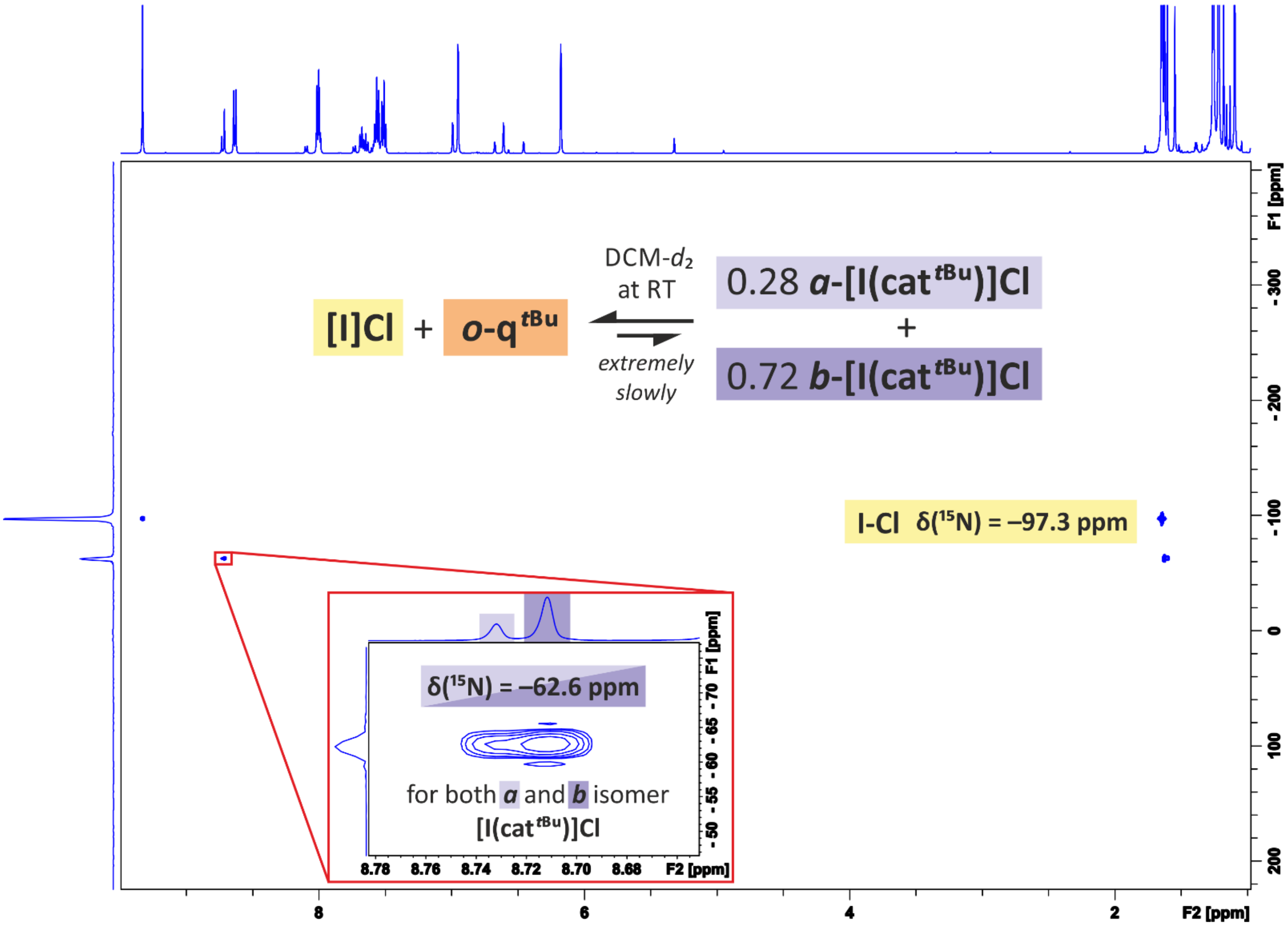

Figure S27: ^{1}H-^{15}N **HMBC NMR** spectrum of mixture of two isomers ***a*/*b*-[I(cat^{tBu})]Cl** after 2 months of being in a dynamic equilibrium with starting **[I]Cl** and ***o*-q^{tBu}** (DCM-d_2, 500 MHz, 295 K, cnst13 = 5 Hz).

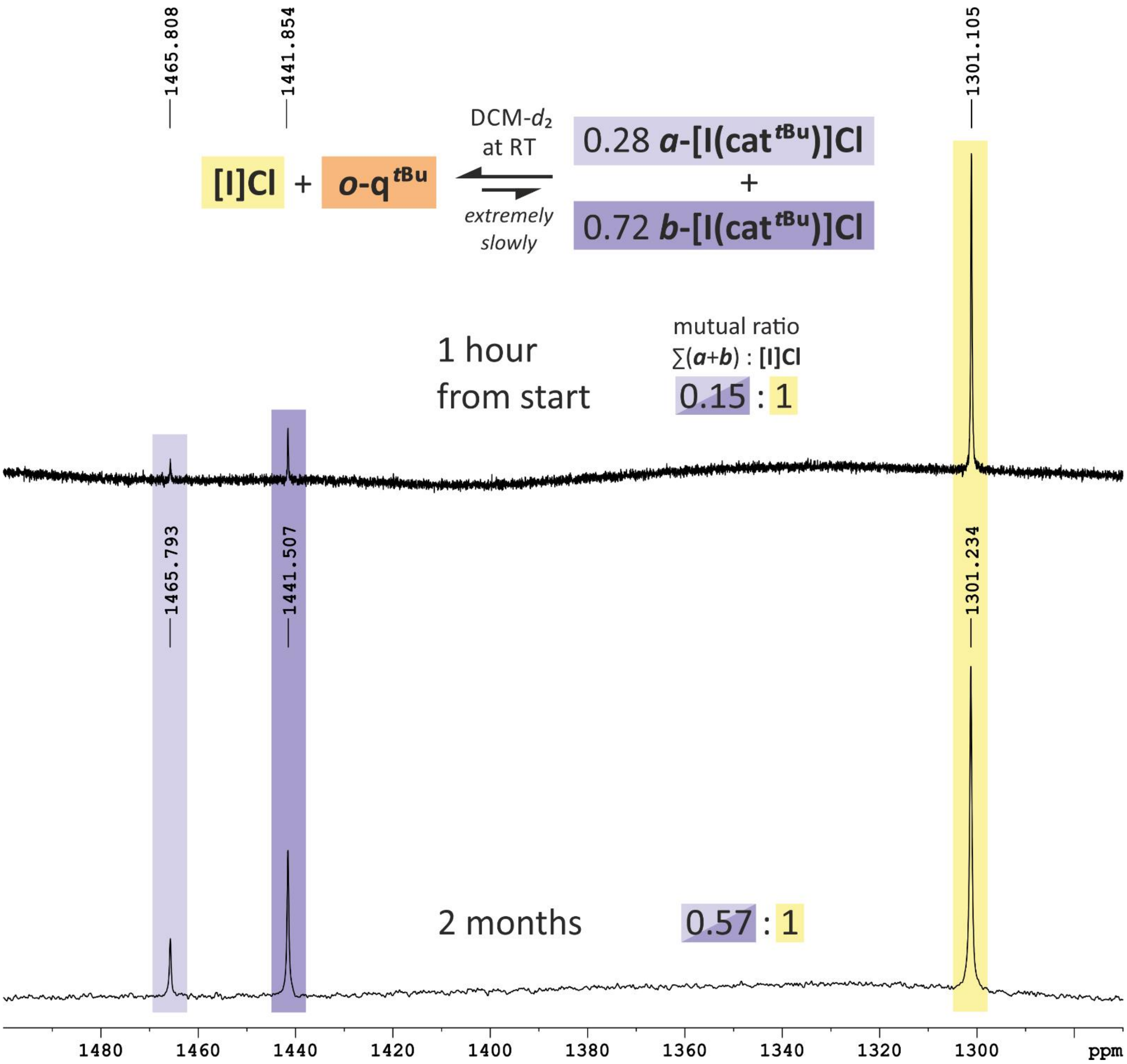

Figure S28: ^{125}**Te{**^{1}**H} NMR** spectrum of mixture of two isomers ***a*/*b*-[I(cat^{tBu})]Cl** after 2 months of being in a dynamic equilibrium with starting **[I]Cl** and ***o*-q^{tBu}** (157.7 MHz, 295 K, DCM-d_2).

NMR spectra proving reversibility in the formation of a/b-[I(cat^{tBu})][OTf]

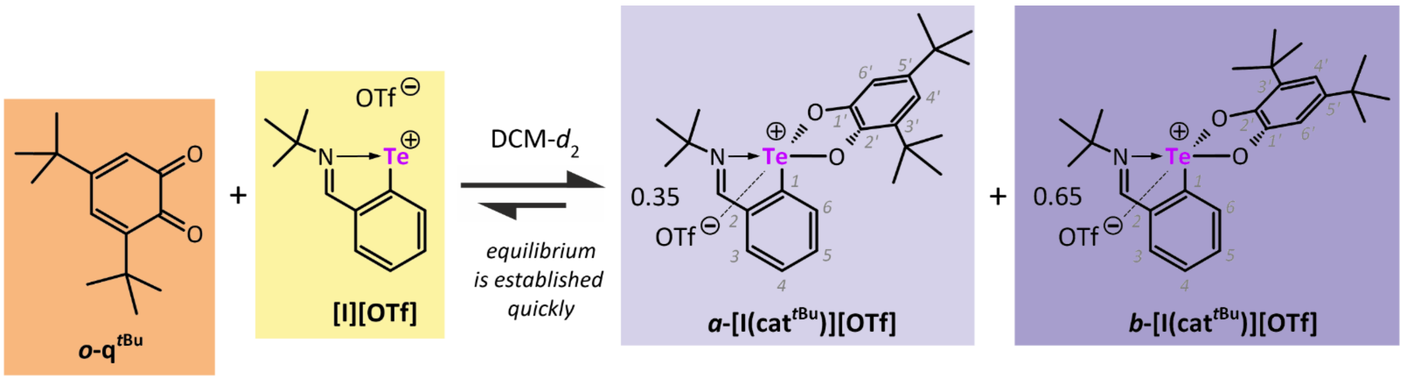

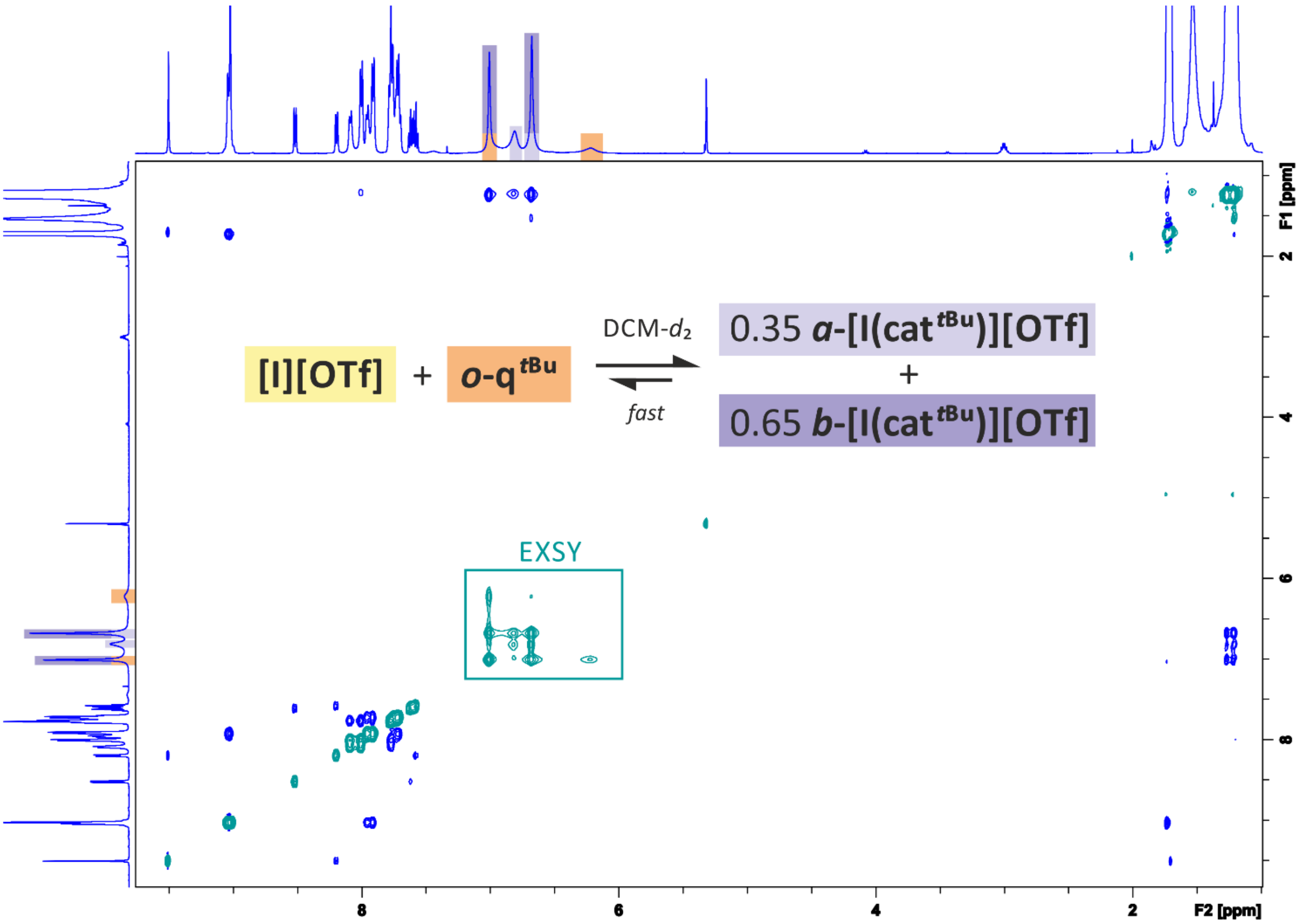

Figure S29: **^{1}H-^{1}H NOESY NMR** spectrum of mixture of two isomers ***a*/*b*-[I(cat^{tBu})][OTf]** in dynamic equilibrium with starting **[I][OTf]** and ***o*-q^{tBu}** (500.20 MHz, 295 K, DCM-d_2, d8 = 3 s).

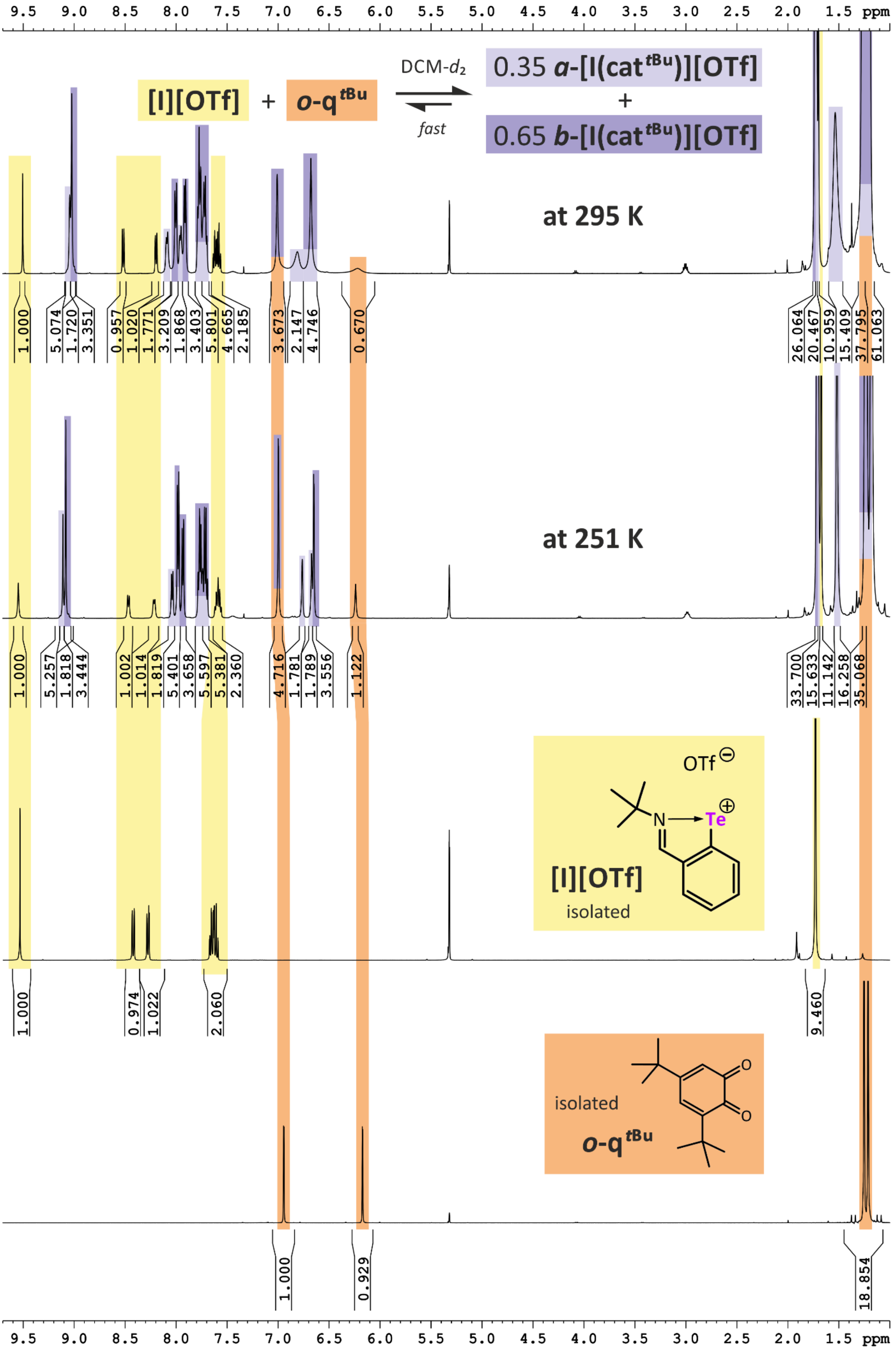

Figure S30: Stacked plot of ^{1}**H NMR** spectra at 295 and 251 K of the reaction between **[I][OTf]** and ***o*-q**^{tBu} in 1:1 molar ratio in DCM-d_2 resulting in fast formation of two isomers ***a*/*b*-[I(cat^{tBu})][OTf]** being in a dynamic equilibrium with starting **[I][OTf]** and ***o*-q**^{tBu} (500.20 MHz, 295 K) and comparison with spectra of isolated **[I][OTf]** and ***o*-q**^{tBu} at 295 K.

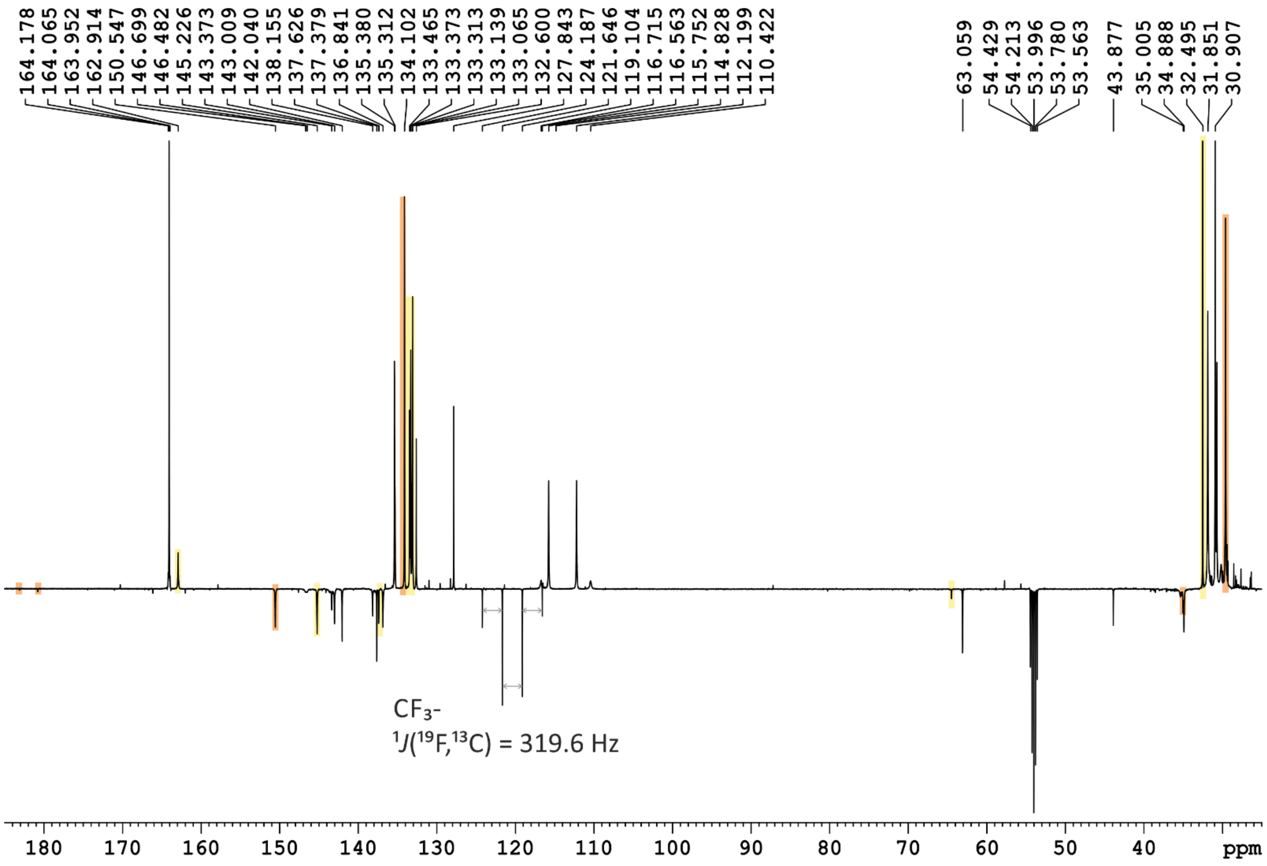

Figure S31: $^{13}C\{^{1}H\}$ **APT NMR** spectrum of mixture of two isomers ***a*/*b*-[I(cat^{tBu})][OTf]** in dynamic equilibrium with starting **[I][OTf]** and ***o*-q^{tBu}** (125.78 MHz, 295 K, DCM-d_2).

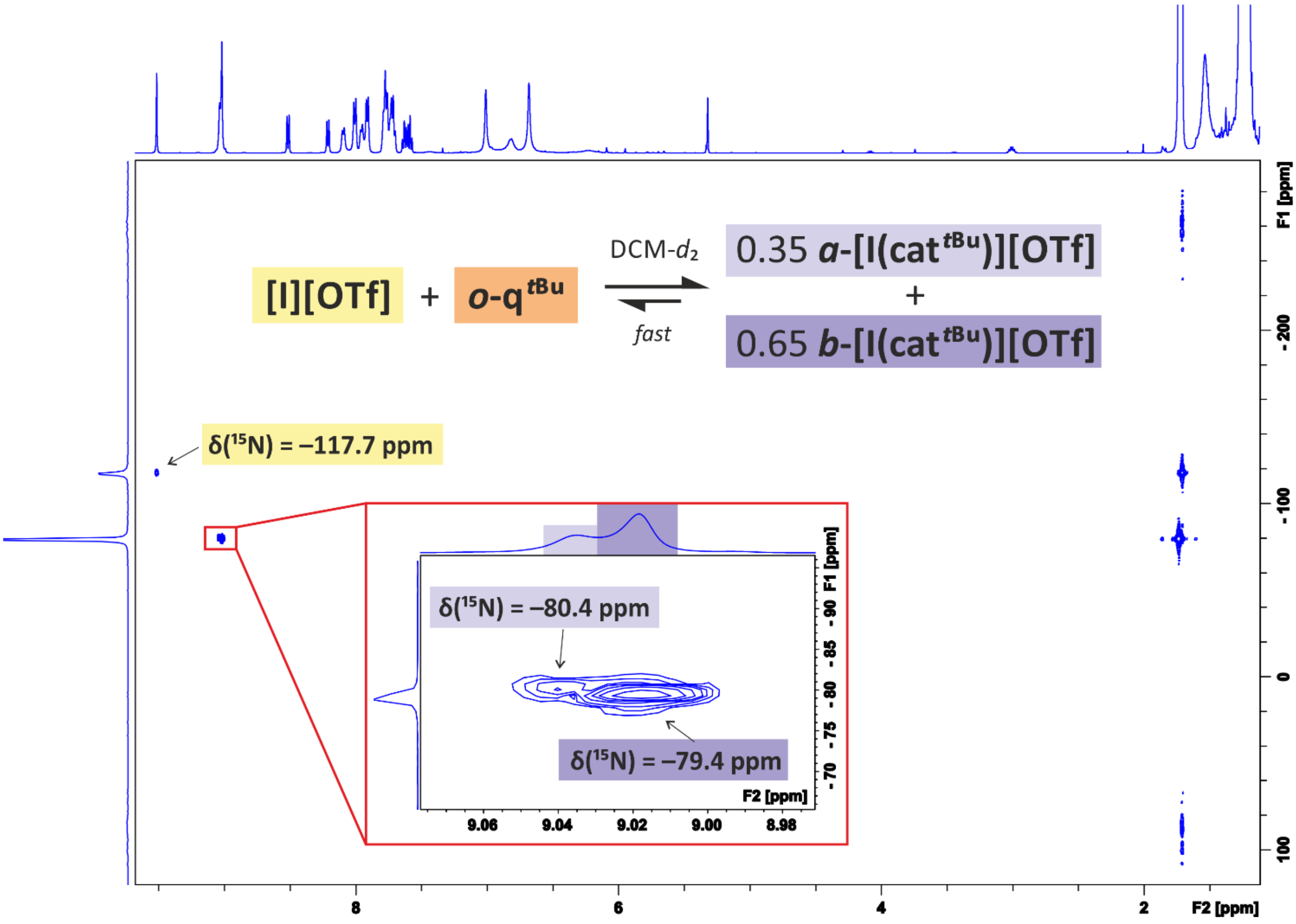

Figure S32: ^{1}H-^{15}N **HMBC NMR** spectrum of mixture of two isomers ***a*/*b*-[I(cat^{tBu})][OTf]** in dynamic equilibrium with starting **[I][OTf]** and ***o*-q^{tBu}** (DCM-d_2, 500 MHz, 295 K, cnst13 = 5 Hz).

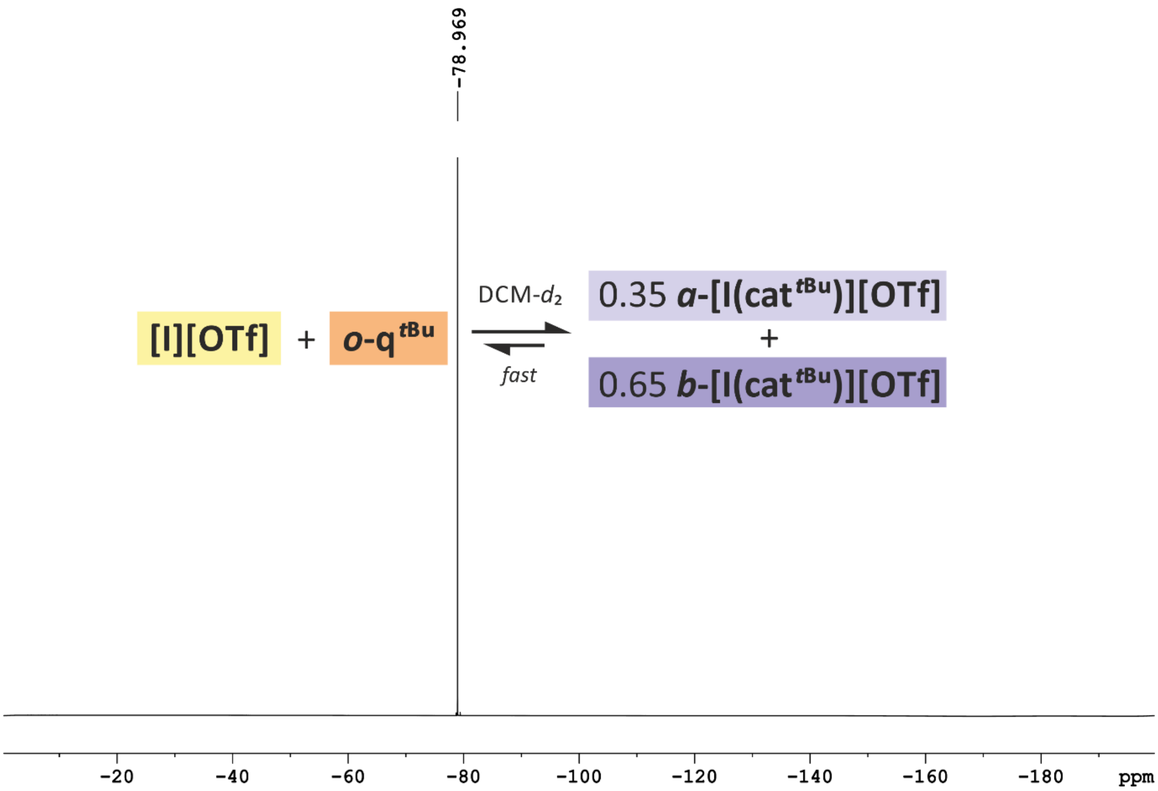

Figure S33: ^{19}**F NMR** spectrum of mixture of two isomers ***a*/*b*-[I(cat^{tBu})][OTf]** in dynamic equilibrium with starting **[I][OTf]** and ***o*-q^{tBu}** (470.66 MHz, 295 K, DCM-d_2).

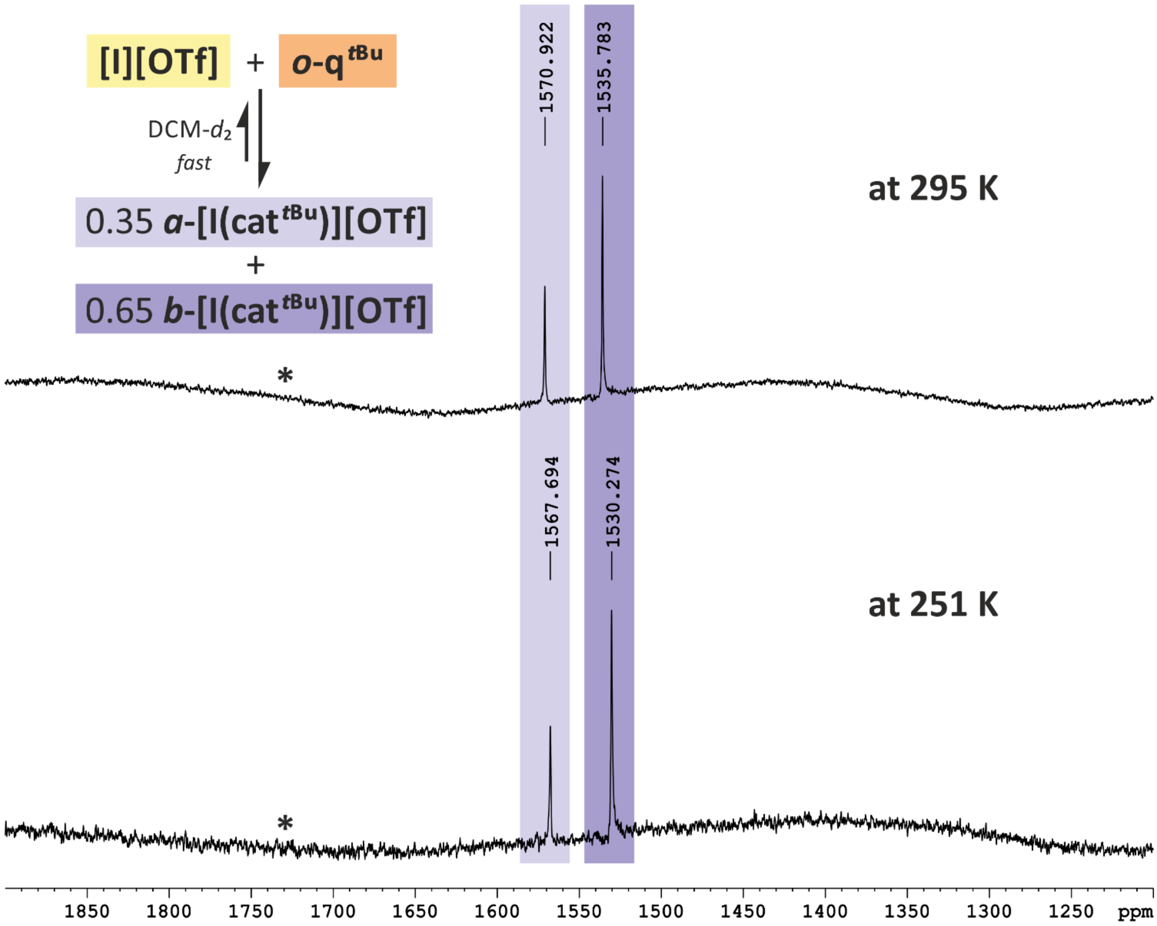

Figure S34: ^{125}**Te{**^{1}**H} NMR** spectrum of mixture of two isomers ***a*/*b*-[I(cat^{tBu})][OTf]** in dynamic equilibrium with starting **[I][OTf]** and ***o*-q^{tBu}** in DCM-d_2 (157.79 MHz, at 295 K (top spectrum) and 251 K (bottom spectrum), NS = 10240 (top spectrum) and 4096 (bottom spectrum). * Signal for the starting **[I][OTf]** (δ(^{125}Te) = in range of 1735 - 1750 ppm) is not observed probably due to the ongoing chemical exchange.

Influencing the equilibrium between a/b-[I(cat^{tBu})][OTf] and the starting compounds by a change in stoichiometry

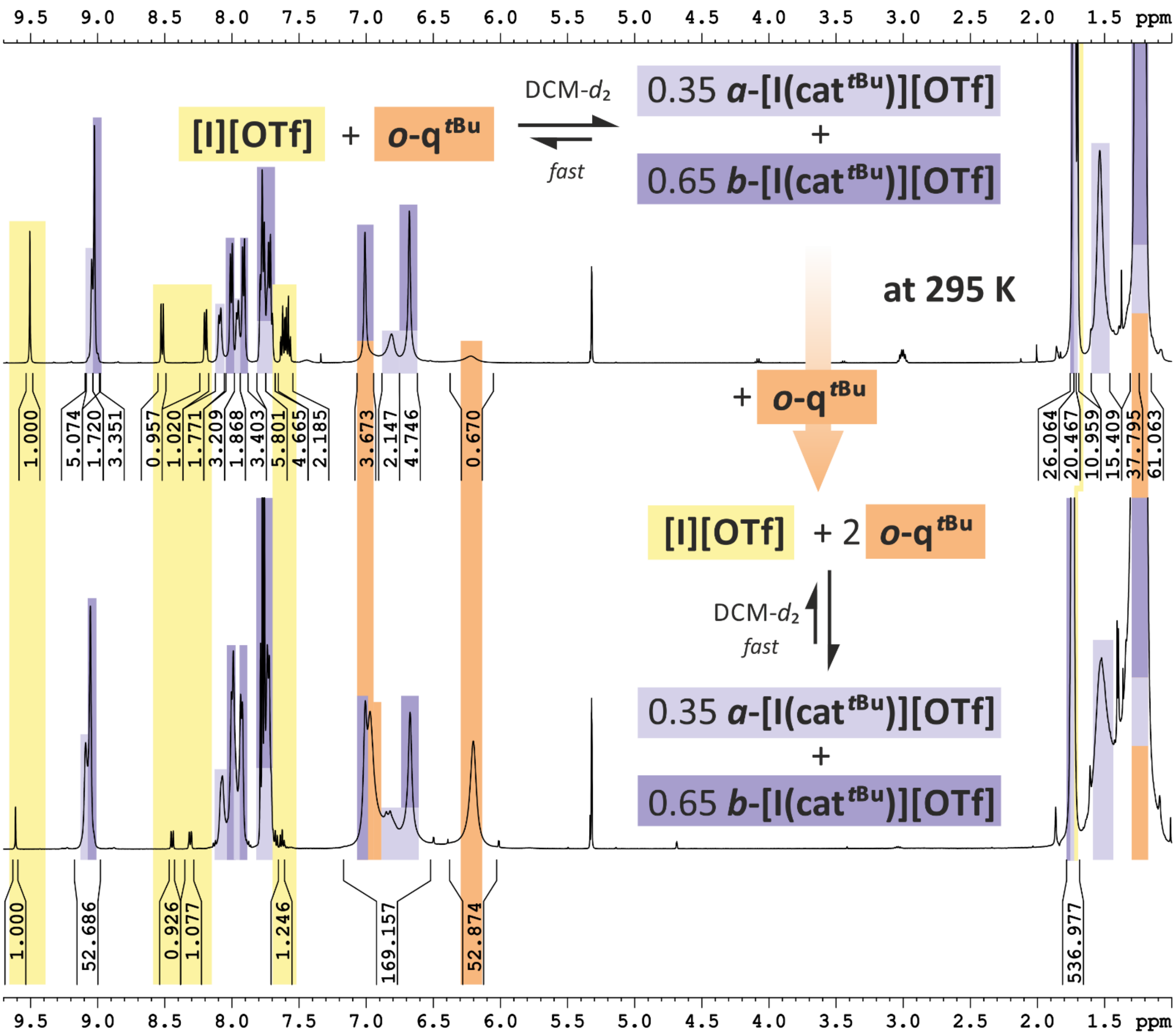

Figure S35: Stacked plot of ^{1}**H NMR** spectra of the dynamic equilibrium between **[I][OTf]** and ***o*-q^{tBu}** in 1:1 molar ratio resulting in formation of two isomers ***a*/*b*-[I(cat^{tBu})][OTf]** (top spectrum) and after addition of the second eq. of ***o*-q^{tBu}** (i.e. molar ratio 1:2) resulting in equilibrium shift (bottom spectrum) (500.20 MHz, 295 K, DCM-d_2).

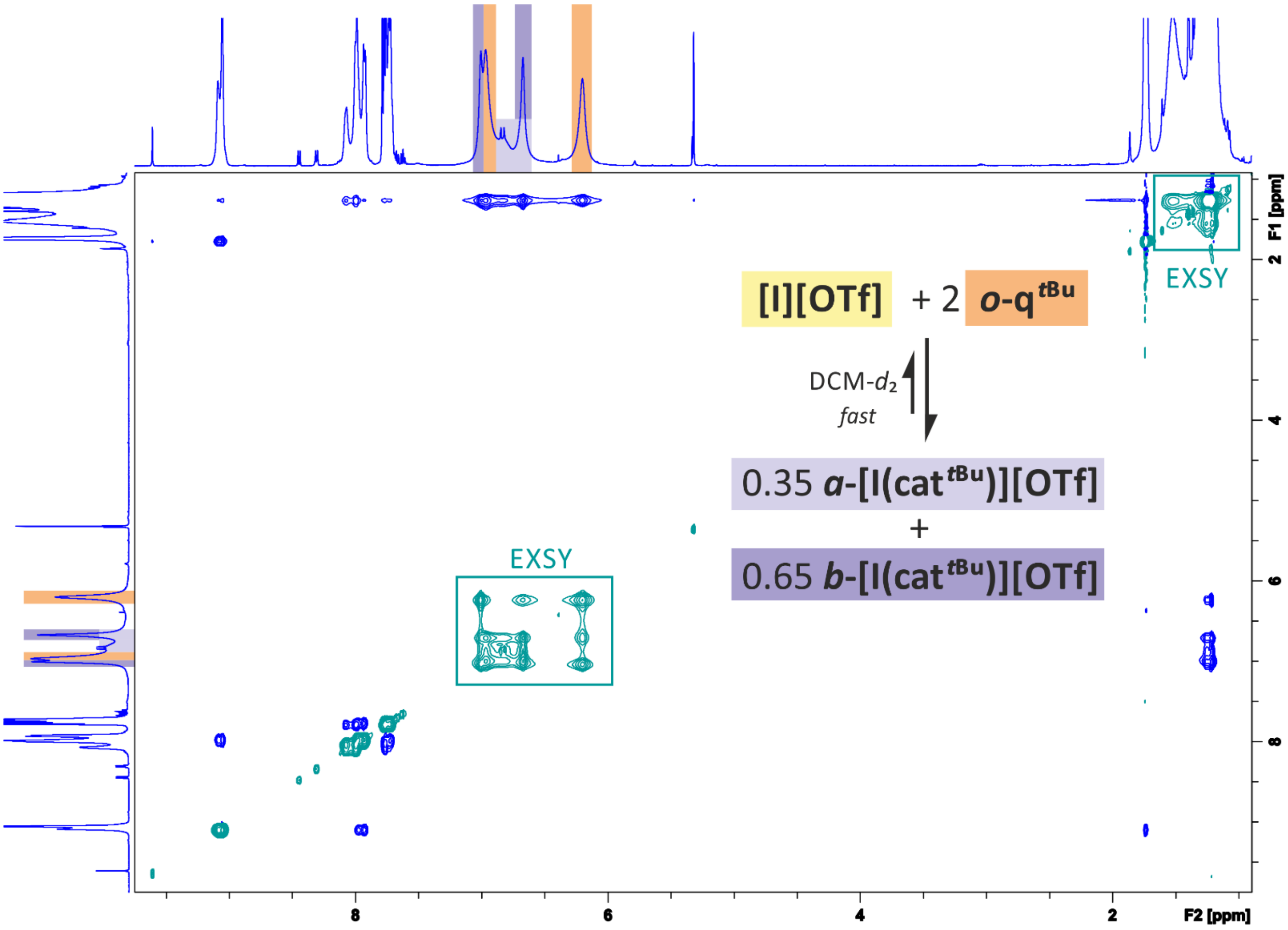

Figure S36: **^{1}H-^{1}H NOESY NMR** obtained after addition of the second eq. of ***o*-q^{tBu}** to a dynamic equilibrium between **[I][OTf]** and ***o*-q^{tBu}** in 1:1 molar ratio resulting in equilibrium shift in formation of two isomers ***a*/*b*-[I(cat^{tBu})][OTf]** (500.20 MHz, 295 K, DCM-d_2, d8 = 2 s).

NMR spectra proving reversibility in the formation of a/b-[I(cat^{tBu})][SbF$_6$]

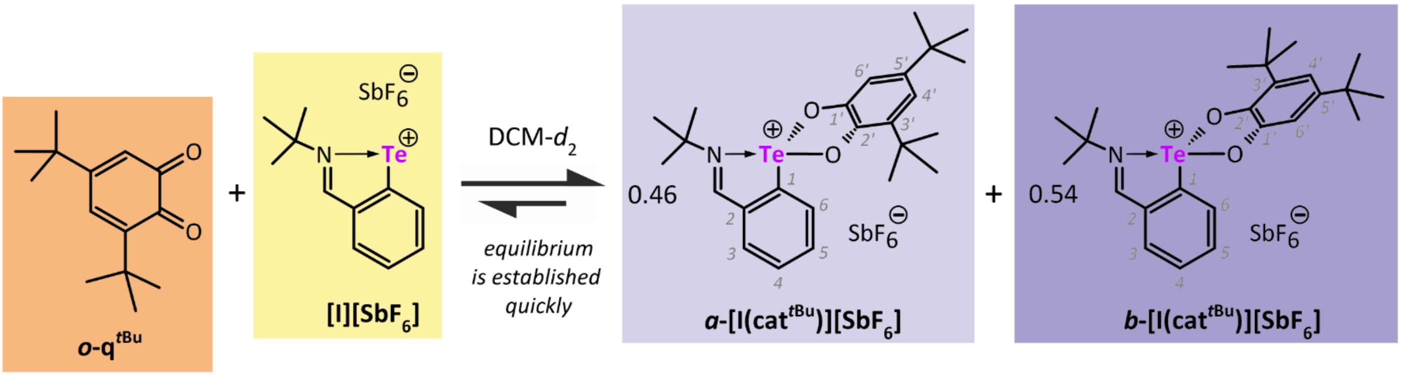

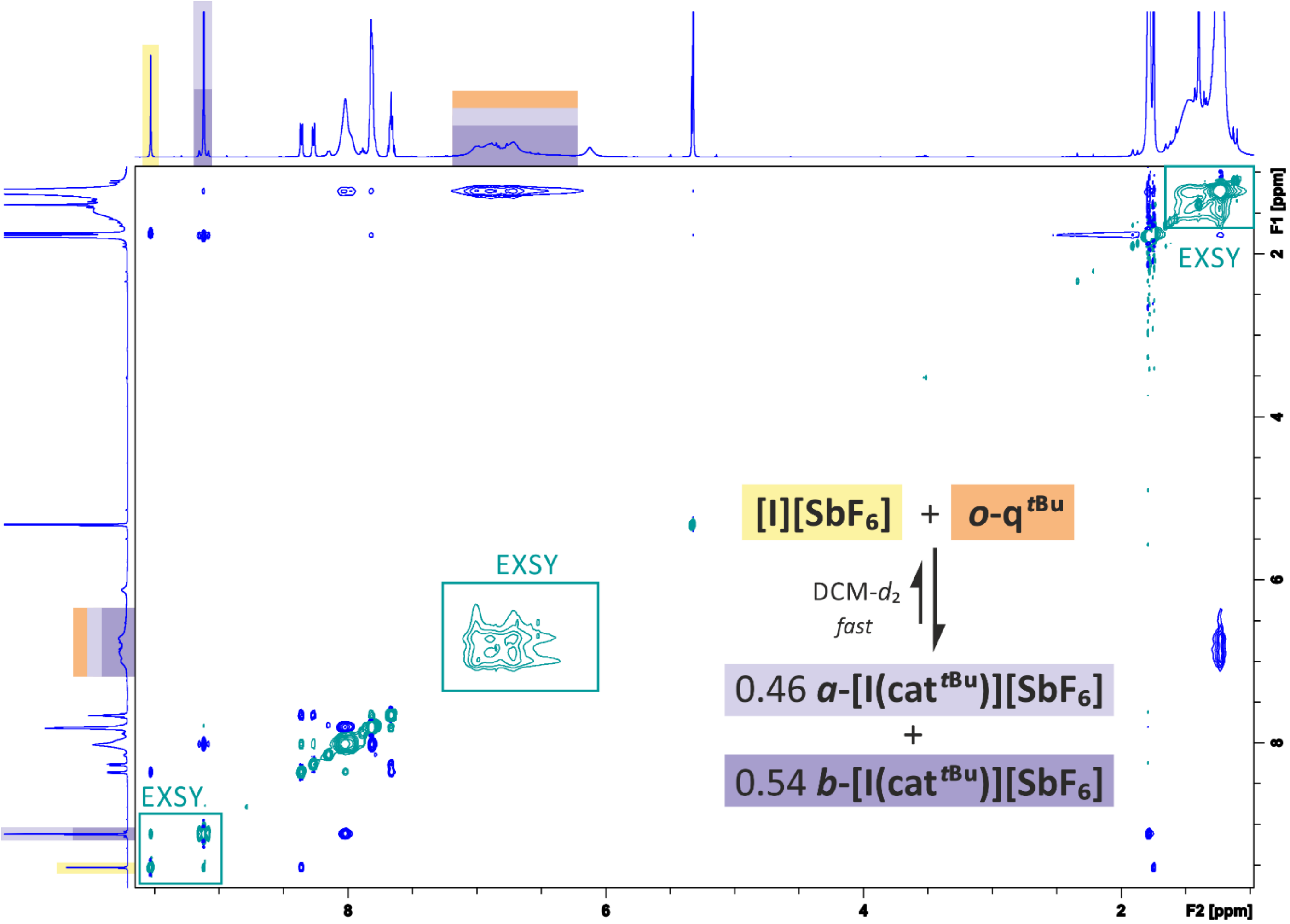

Figure S37: ^{1}H-^{1}H **NOESY NMR** spectrum of mixture of two isomers ***a*/*b*-[I(cat^{tBu})][SbF$_6$]** in dynamic equilibrium with starting **[I][SbF$_6$]** and ***o*-q^{tBu}** (500.20 MHz, 295 K, DCM-d_2, d8 = 2 s).

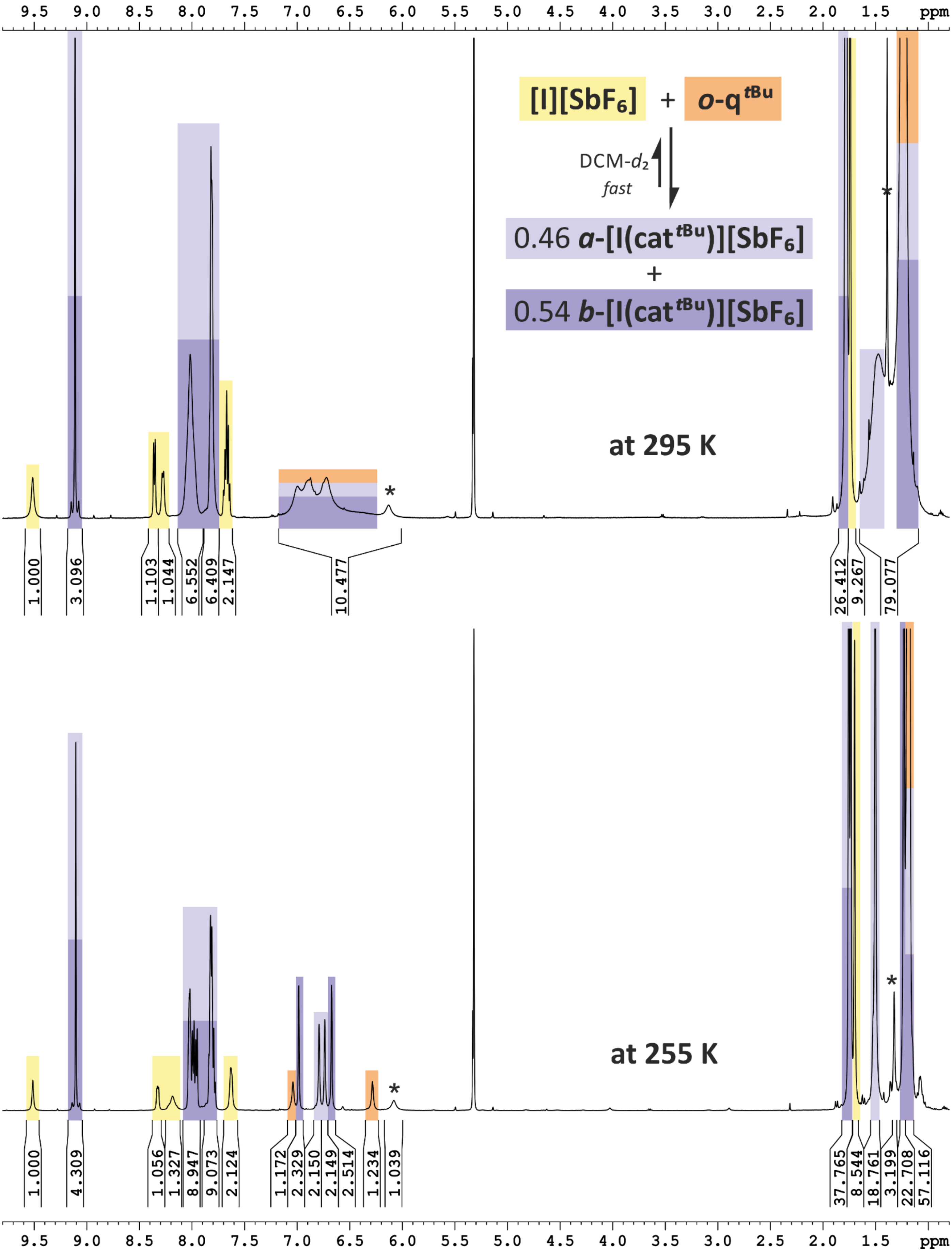

Figure S38: Stacked plot of ^{1}**H NMR** spectra at 295 and 255 K of the reaction between **[I][SbF$_6$]** and ***o*-q^{tBu}** in 1:1 molar ratio in DCM-d_2 resulting in fast formation of two isomers ***a*/*b*-[I(cat^{tBu})][SbF$_6$]** being in a dynamic equilibrium with starting **[I][SbF$_6$]** and ***o*-q^{tBu}** (500.20 MHz, 295 K). * denotes signals of unknown species.

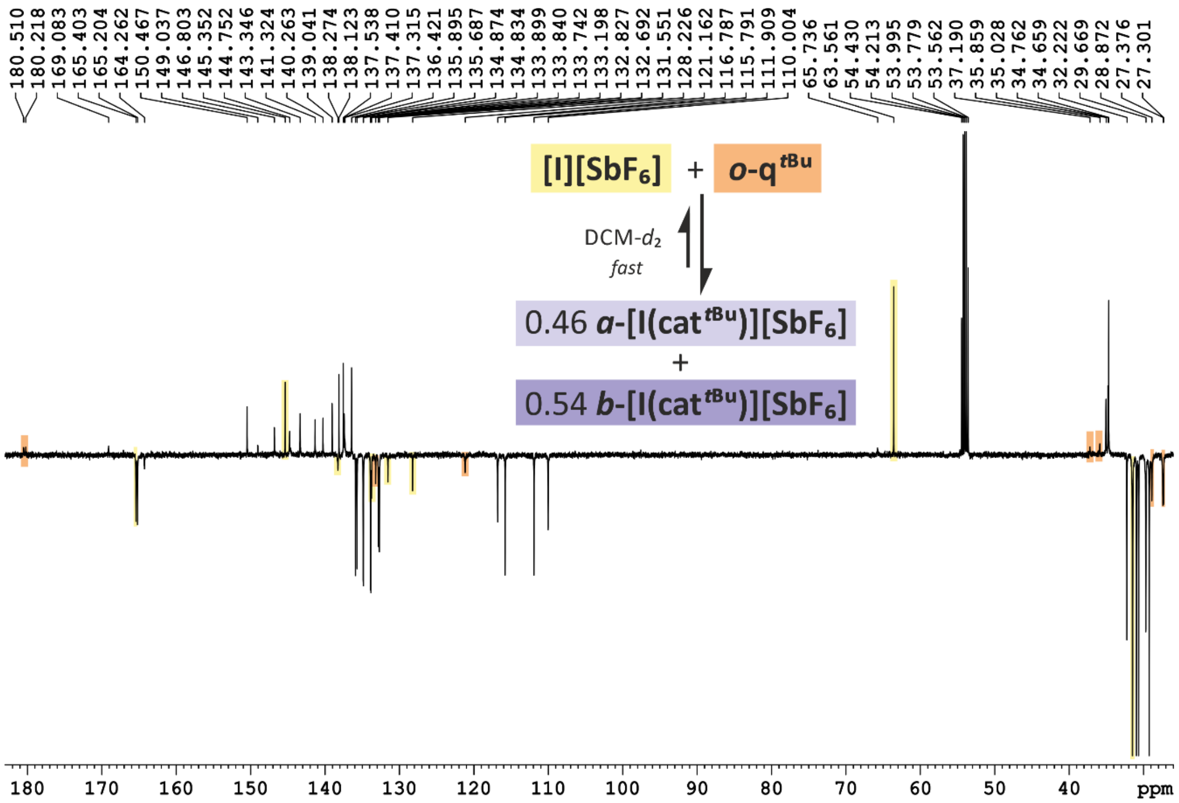

Figure S39: $^{13}C\{^1H\}$ **APT NMR** spectrum of mixture of two isomers ***a*/*b*-[I(cat^{tBu})][SbF$_6$]** in dynamic equilibrium with starting **[I][SbF$_6$]** and ***o*-q^{tBu}** (125.78 MHz, 295 K, DCM-d_2).

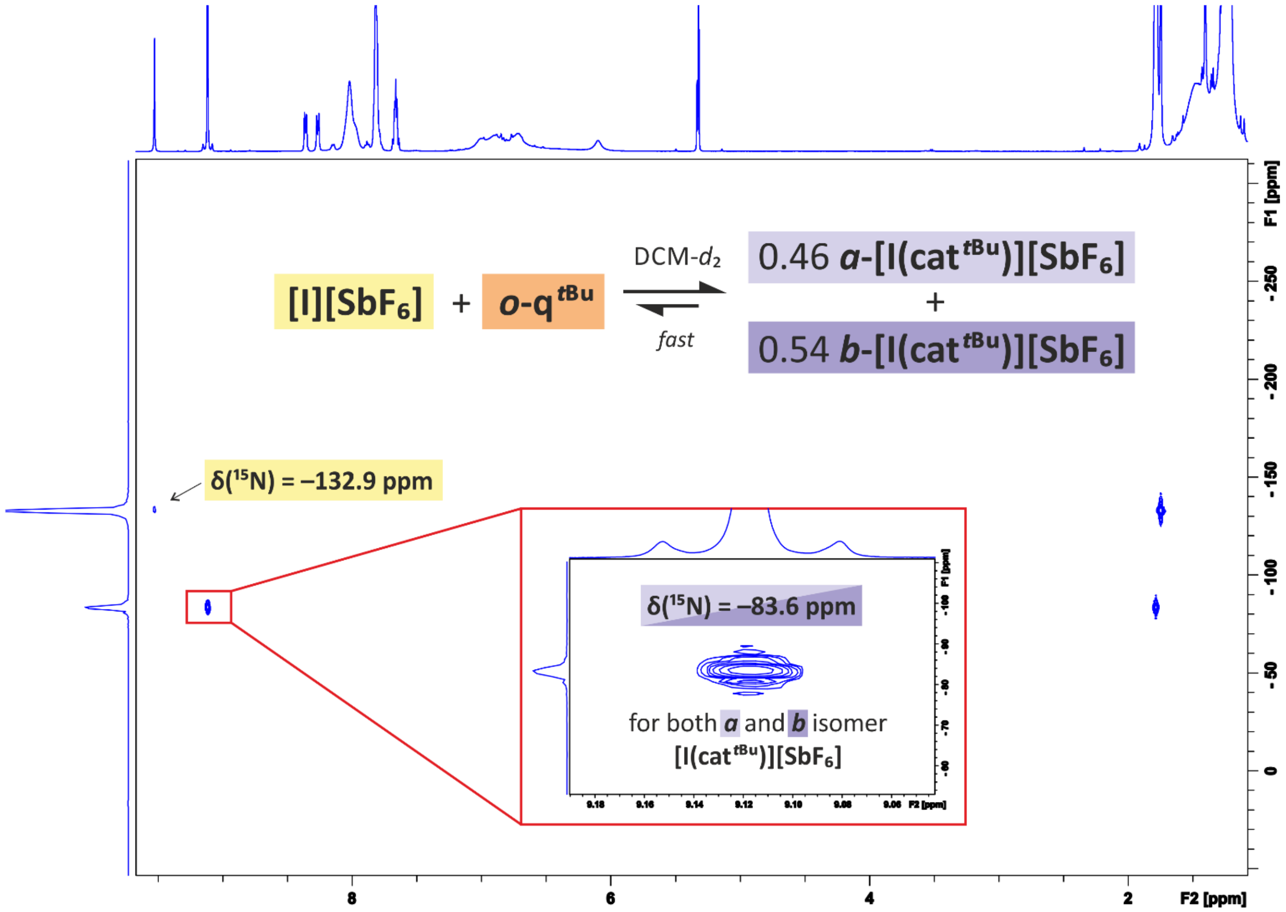

Figure S40: ^1H-^{15}N **HMBC NMR** spectrum of mixture of two isomers ***a*/*b*-[I(cat^{tBu})][SbF$_6$]** in dynamic equilibrium with starting **[I][SbF$_6$]** and ***o*-q^{tBu}** (DCM-d_2, 500 MHz, 295 K, cnst13 = 5 Hz).

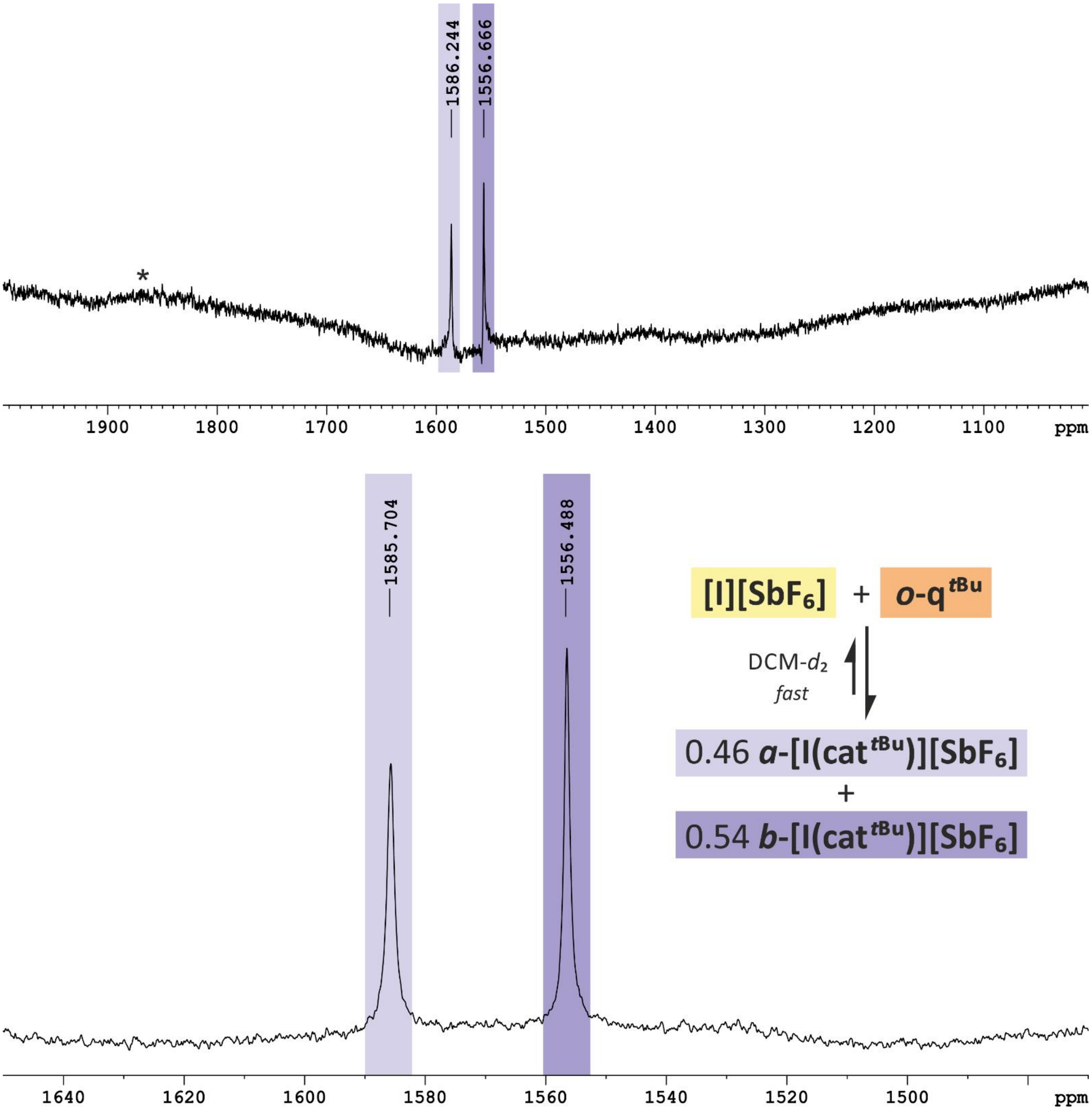

Figure S41: ^{125}Te{^{1}H} NMR spectrum of mixture of two isomers ***a*/*b*-[I(cat^{tBu})][SbF$_6$]** in dynamic equilibrium with starting **[I[[SbF$_6$]** and ***o*-q^{tBu}** in DCM-d_2 (157.79 MHz, at 295 K (top spectrum) and 251 K (bottom spectrum), NS = 7400 (top spectrum) and 51200 (bottom spectrum) with baseline correction. * Signal for the starting **[I][SbF$_6$]** (δ(^{125}Te) = 1896.5 ppm) is not observed probably due to the ongoing chemical exchange.

Reaction of [I]Cl with o-q^{phen} and corresponding NMR spectra

Yellow polycrystals of **[I]Cl** (140.0 mg, 0.433 mmol) and orange powder of ***o*-q^{phen}** (90.2 mg, 0.433 mmol) were loaded into a Schlenk tube under an argon atmosphere. After addition of DCM-d_2 (3 mL), an orange solution was formed. NMR analysis showed no reaction:

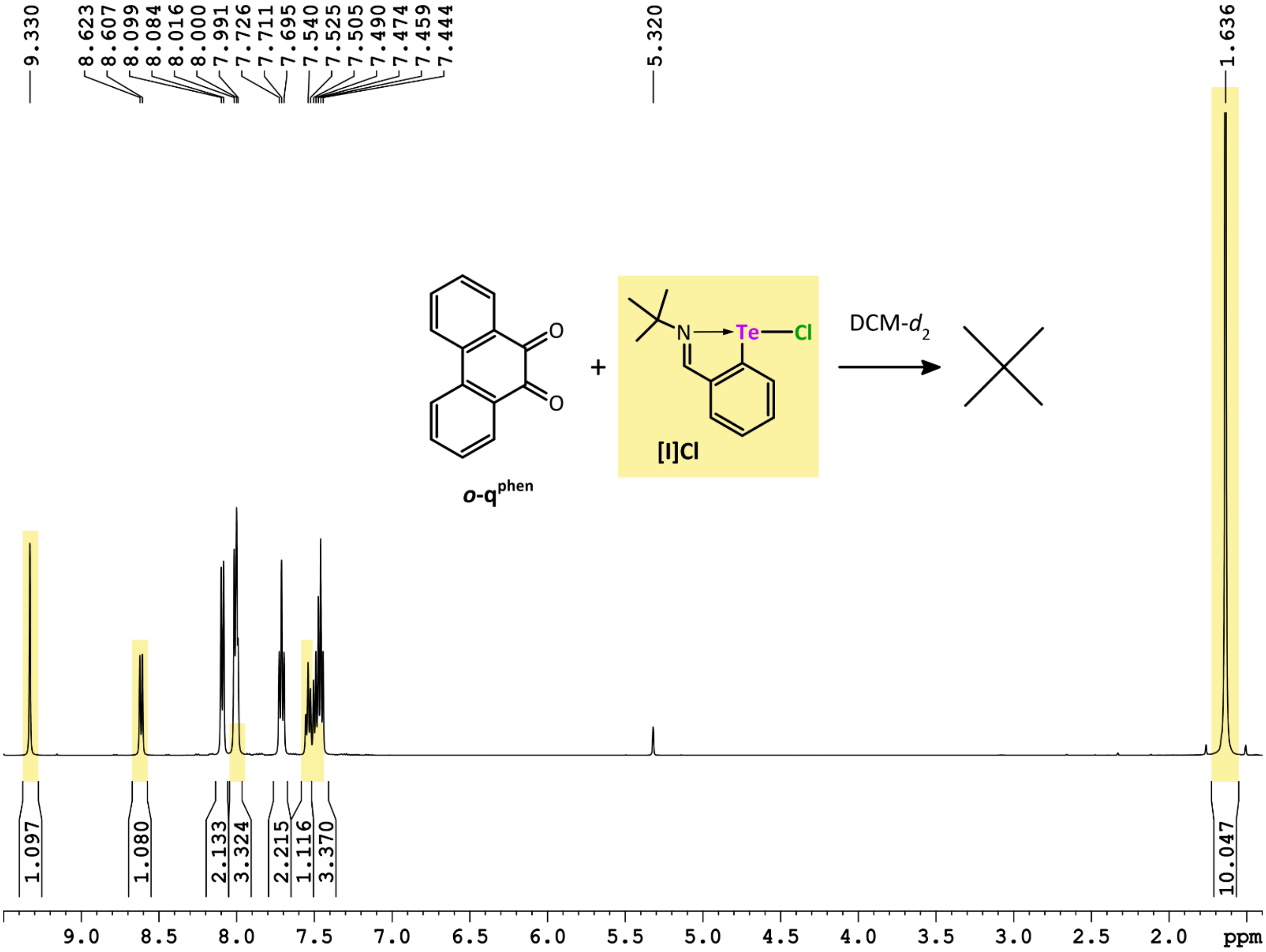

Figure S42: **^{1}H NMR** spectrum of 1:1 mixture of **[I]Cl** and ***o*-q^{phen}** in DCM-d_2 showing no reaction (500 MHz, 295 K).

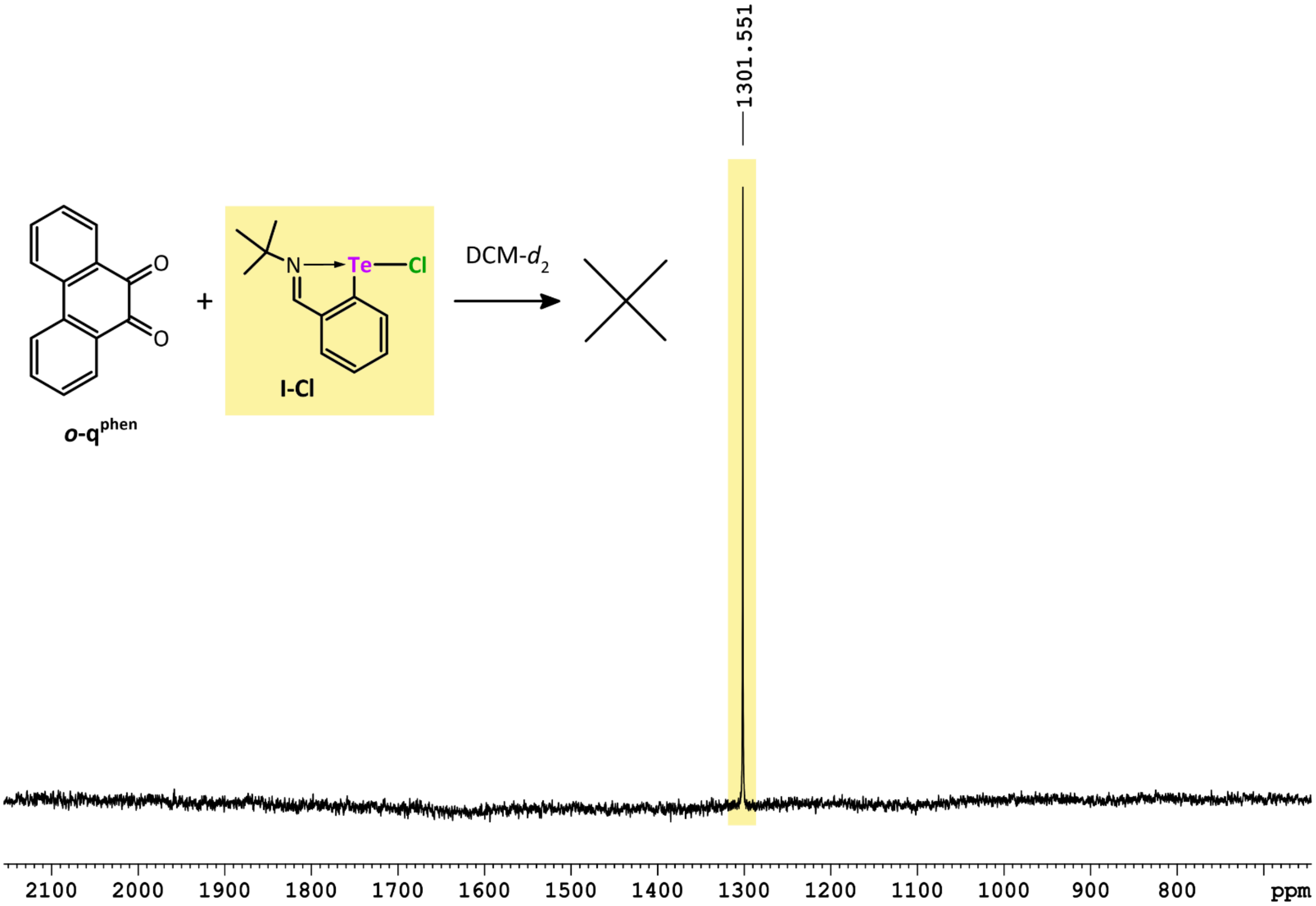

Figure S43: **^{125}Te{^{1}H} NMR** spectrum of 1:1 mixture of **[I]Cl** and ***o*-q^{phen}** in DCM-d_2 showing no reaction (157.7 MHz, 295 K, NS = 5000).

Reaction of [I][OTf] with o-q^phen and corresponding NMR spectra

Yellow polycrystals of **[I][OTf]** (133.1 mg, 0.304 mmol) and orange powder of ***o*-q^phen** (63.4 mg, 0.304 mmol) were loaded into a Schlenk tube under an argon atmosphere. After addition of DCM-d_2 (3 mL), an orange solution was formed. NMR analysis showed no reaction:

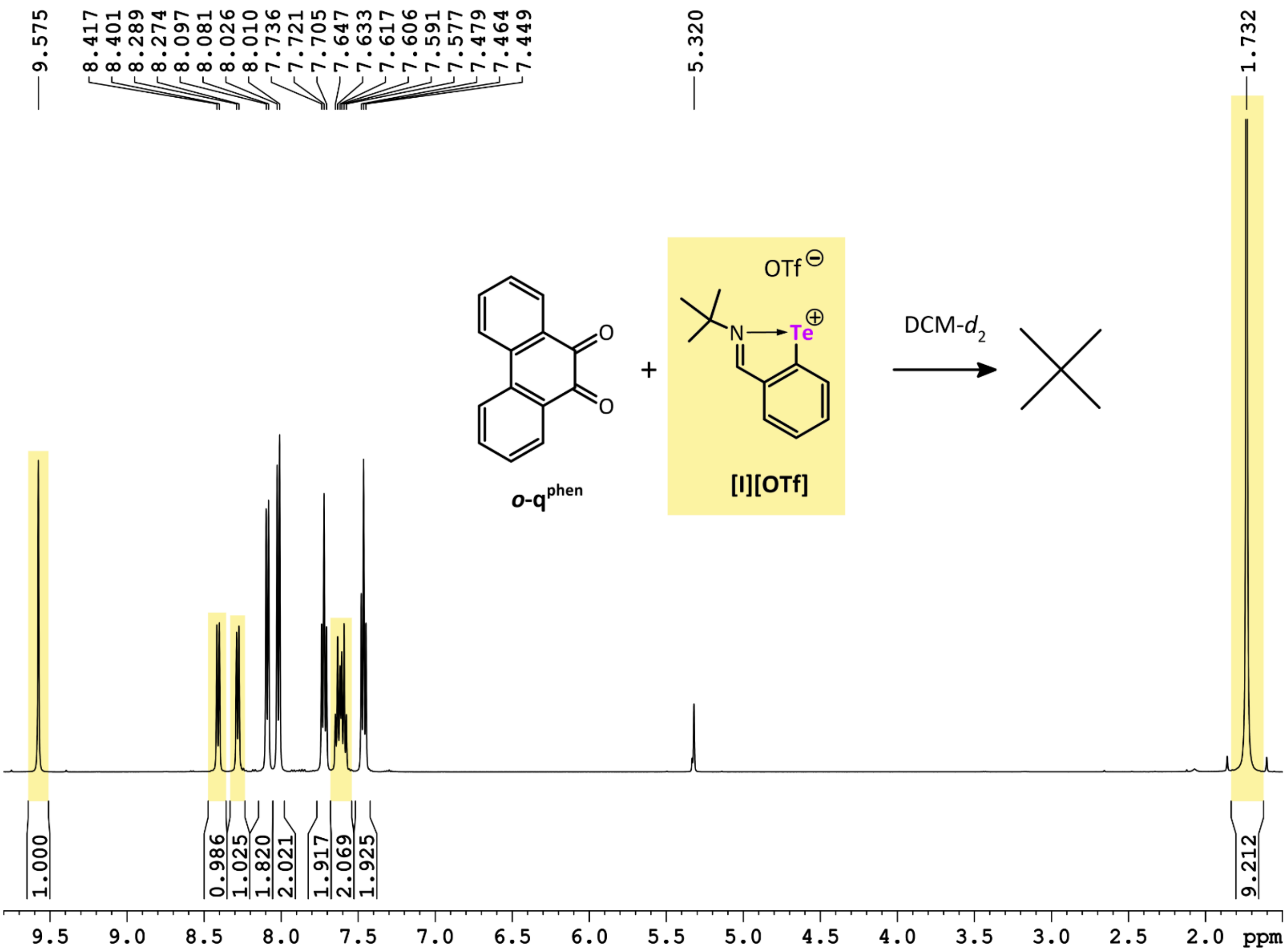

Figure S44: **^{1}H NMR** spectrum of 1:1 mixture of **[I][OTf]** and ***o*-q^phen** in DCM-d_2 showing no reaction (500 MHz, 295 K).

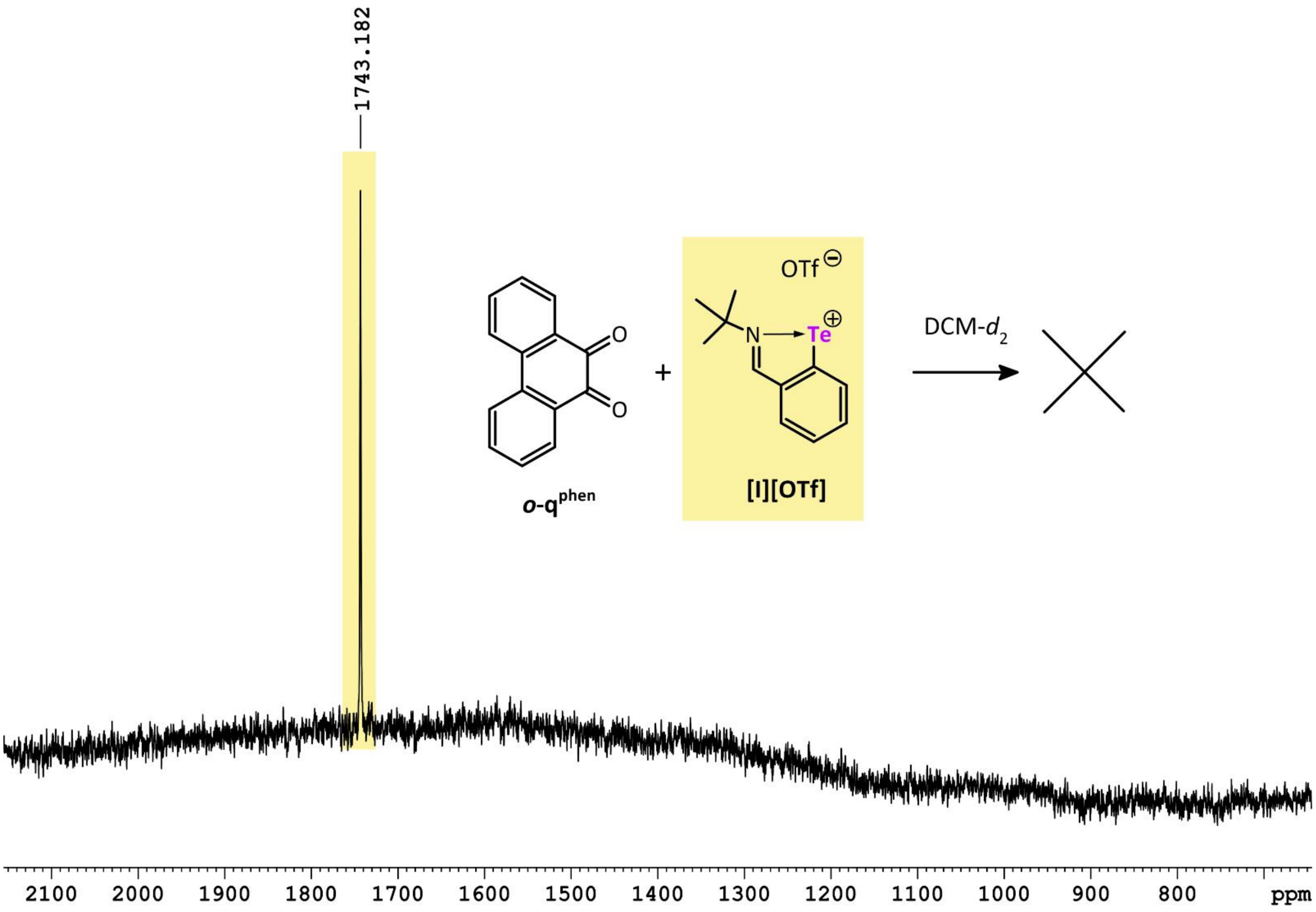

Figure S45: ^{125}Te{^{1}H} NMR spectrum of 1:1 mixture of **[I][OTf]** and ***o*-q^{phen}** in DCM-d_2 showing no reaction (157.7 MHz, 295 K, NS = 560).

NMR spectra of [I·(o-q^{phen})][SbF$_6$]

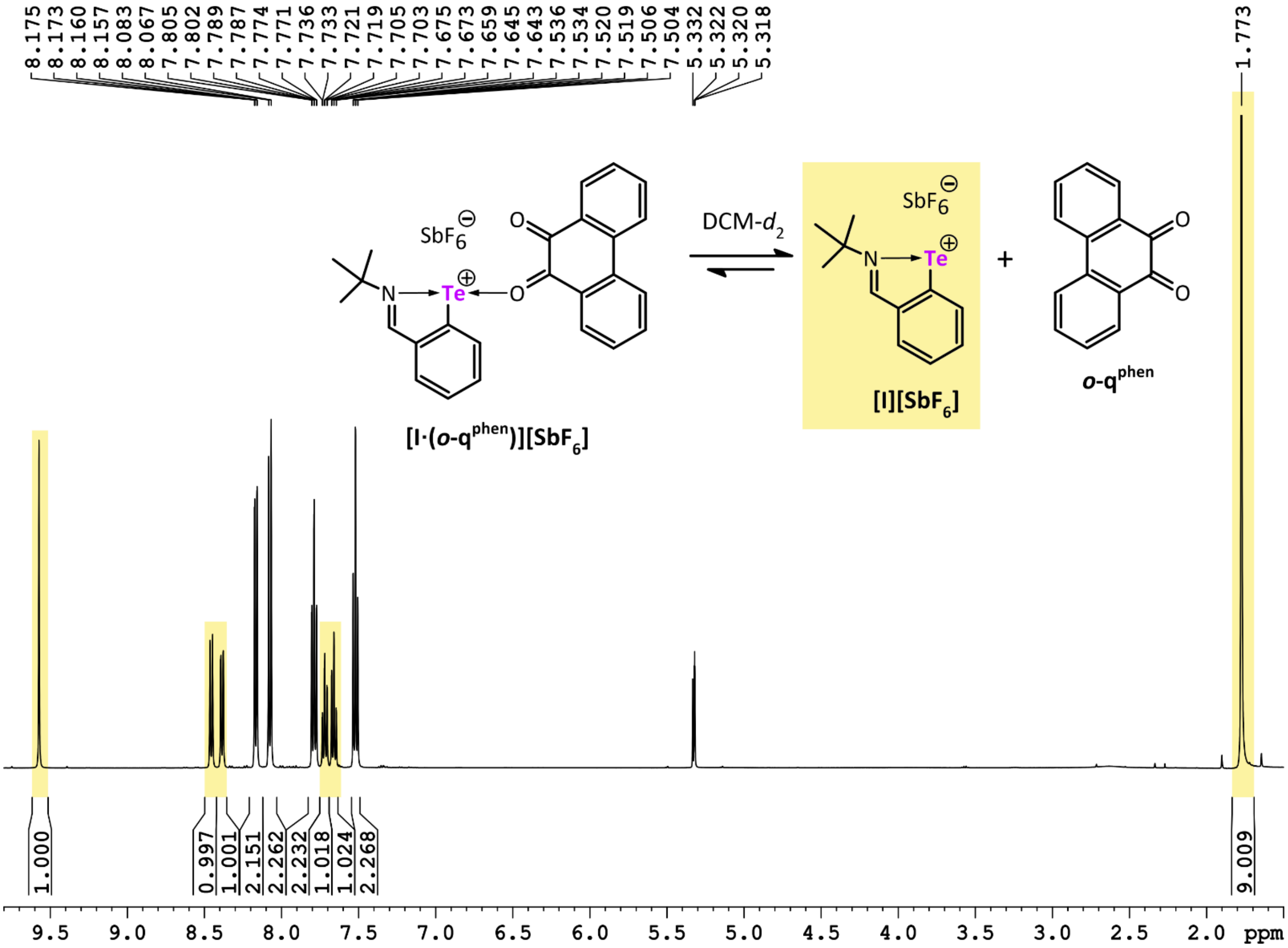

Figure S46: ^{1}**H NMR** spectrum obtained after dissolving single-crystals of **[I·(*o*-q^{phen})][SbF$_6$]** in DCM-d_2 under formation of deep red solution (500 MHz, 295 K).

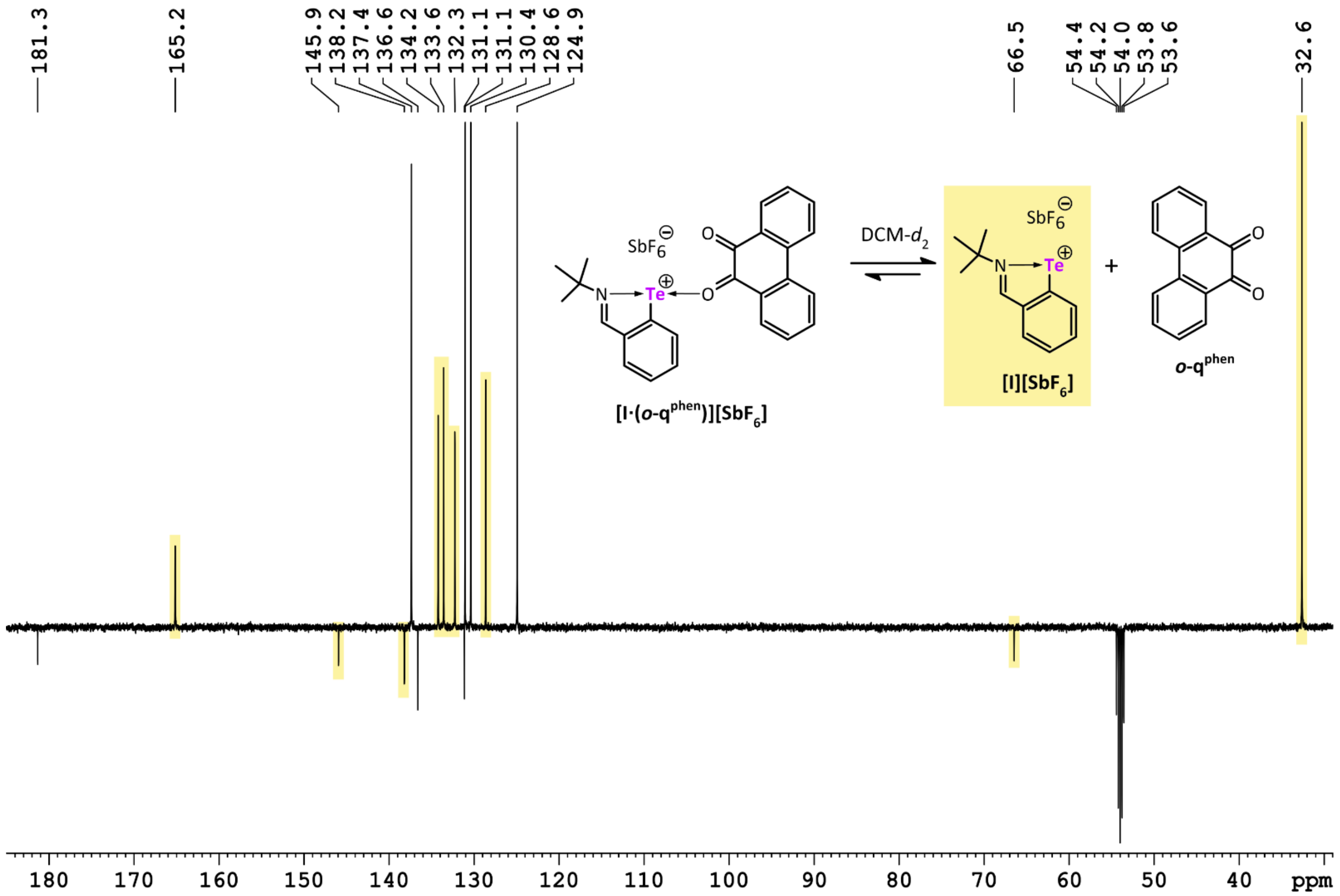

Figure S47: $^{13}C\{^{1}H\}$ **APT NMR** spectrum obtained after dissolving single-crystals of **[I·(*o*-q^{phen})][SbF$_6$]** in DCM-d_2 under formation of deep red solution (125.78 MHz, 295 K).

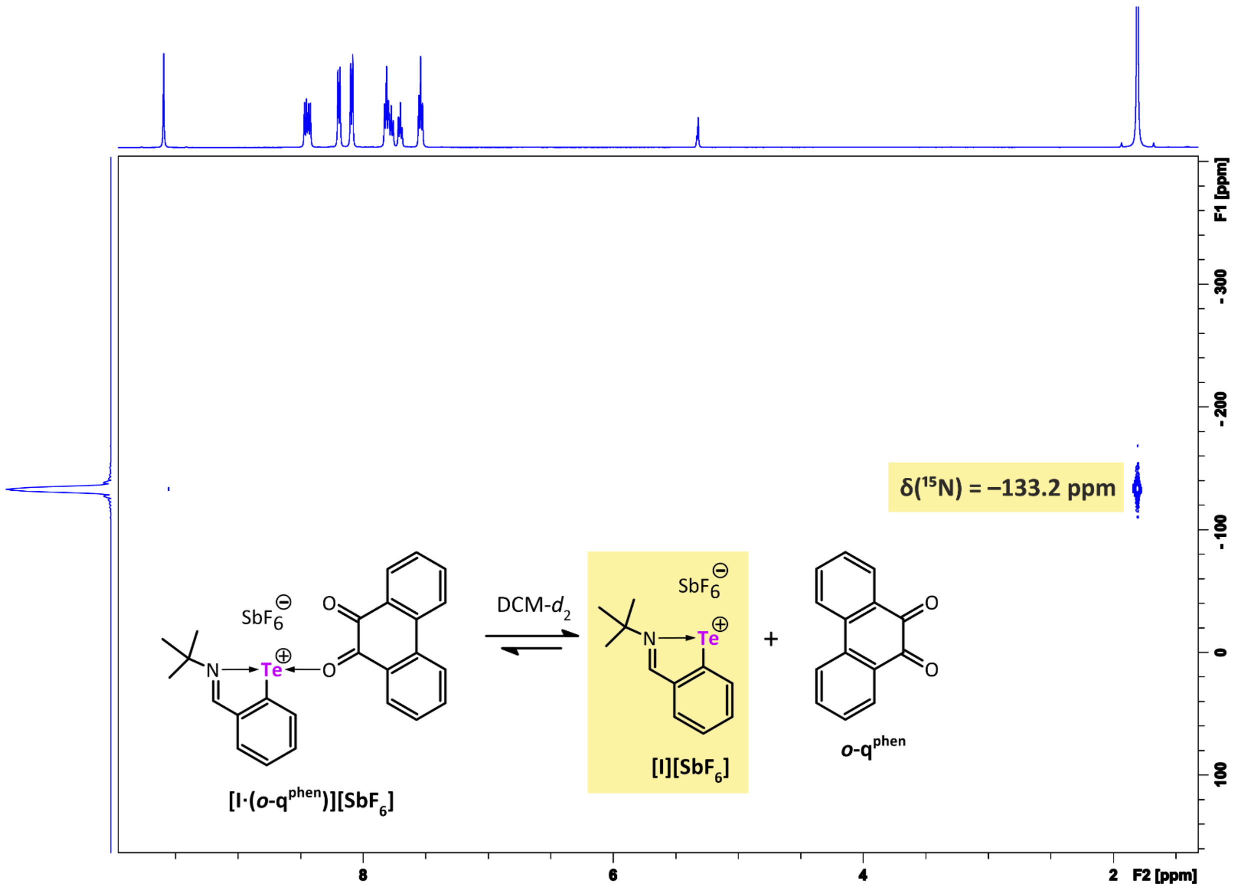

Figure S48: ^{1}H-^{15}N **HMBC NMR** spectrum obtained after dissolving single-crystals of **[I·(*o*-q^{phen})][SbF$_6$]** in DCM-d_2 under formation of deep red solution (500 MHz, 295 K, cnst13 = 5 Hz).

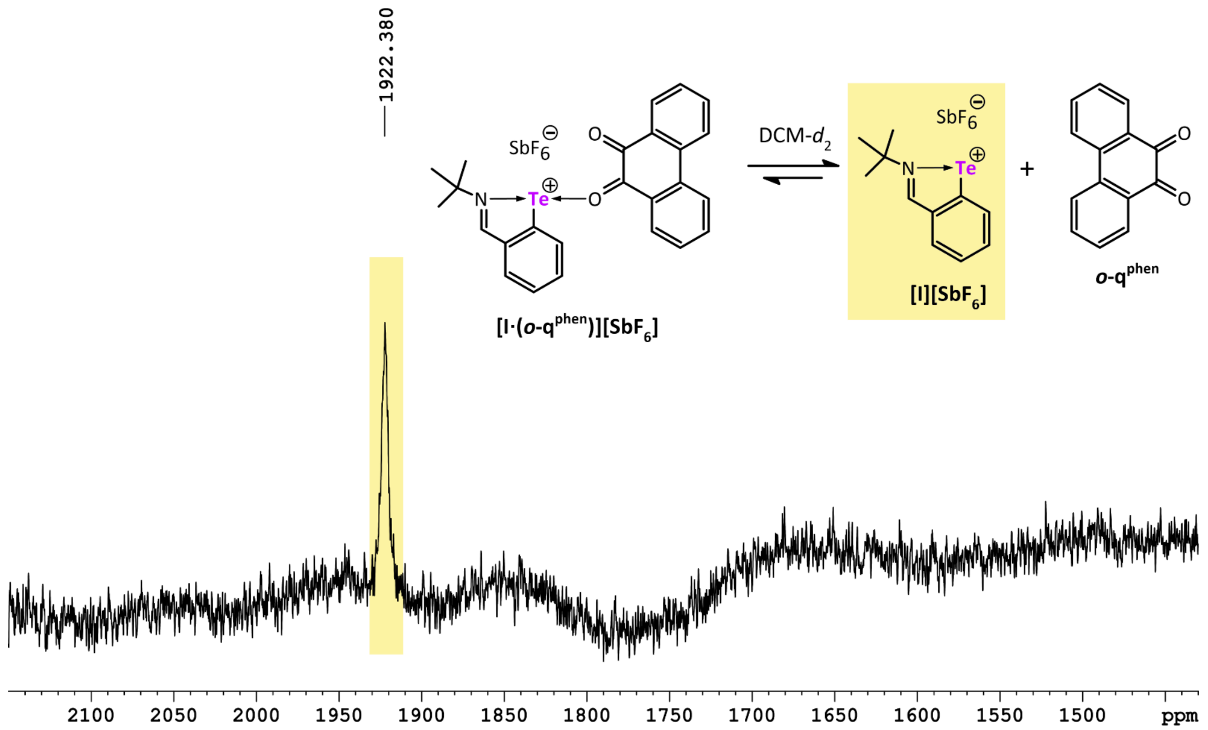

Figure S49: ^{125}Te{^{1}H} NMR spectrum obtained after dissolving single-crystals of **[I·(*o*-q^{phen})][SbF$_6$]** in DCM-d_2 under formation of deep red solution (157.7 MHz, 295 K, NS = 153600).

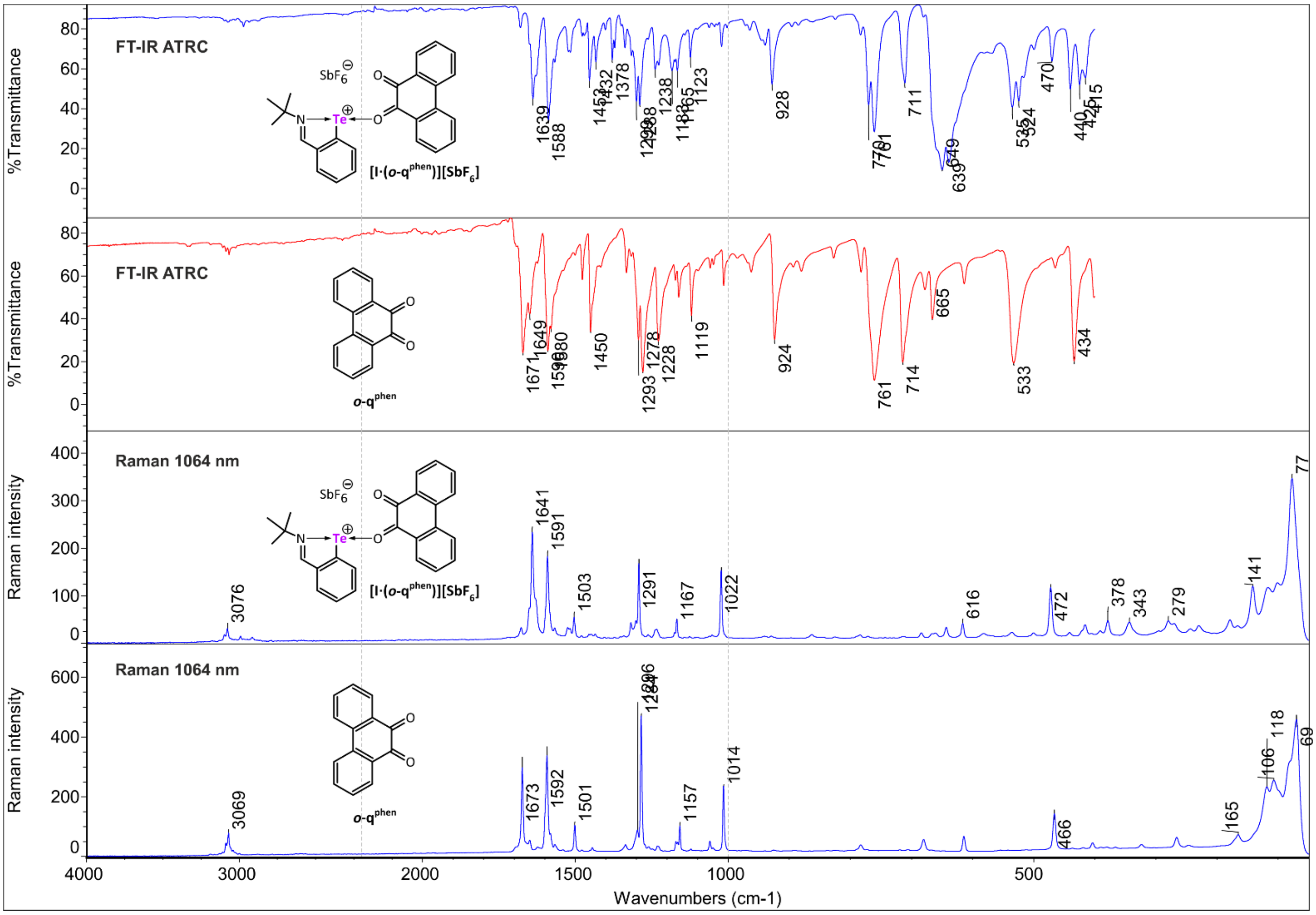

Figure S50: FT-IR and Raman spectra of single-crystalline form of **[I·(*o*-q^{phen})][SbF$_6$]** and of free ***o*-q^{phen}** proving quinone character of the ***o*-q^{phen}** moiety coordinated to Te atom within **[I·(*o*-q^{phen})][SbF$_6$]** adduct.

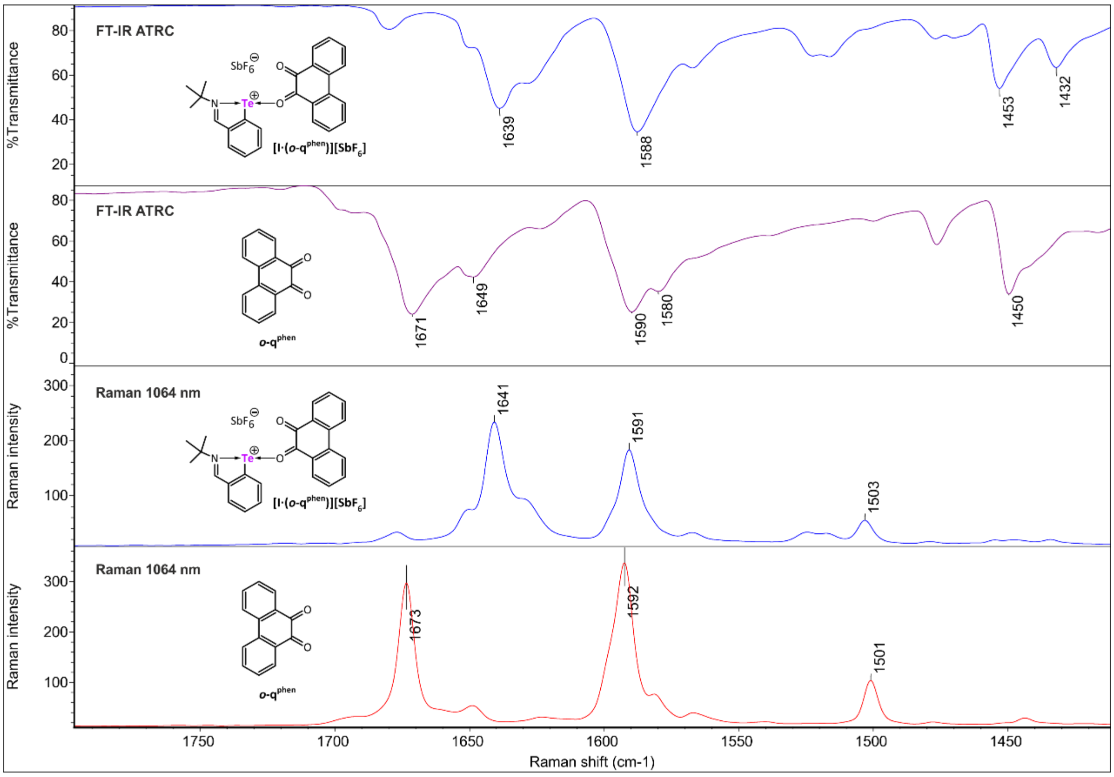

Figure S51: FT-IR and Raman spectra of single-crystalline form of **[I·(*o*-q^phen^)][SbF$_6$]** and of free ***o*-q^phen^** proving quinone character of the ***o*-q^phen^** moiety coordinated to Te atom within **[I·(*o*-q^phen^)][SbF$_6$]** adduct.

NMR spectra obtained after dissolving [I·(o-q^phen^)][SbF$_6$] in MeCN-d$_3$

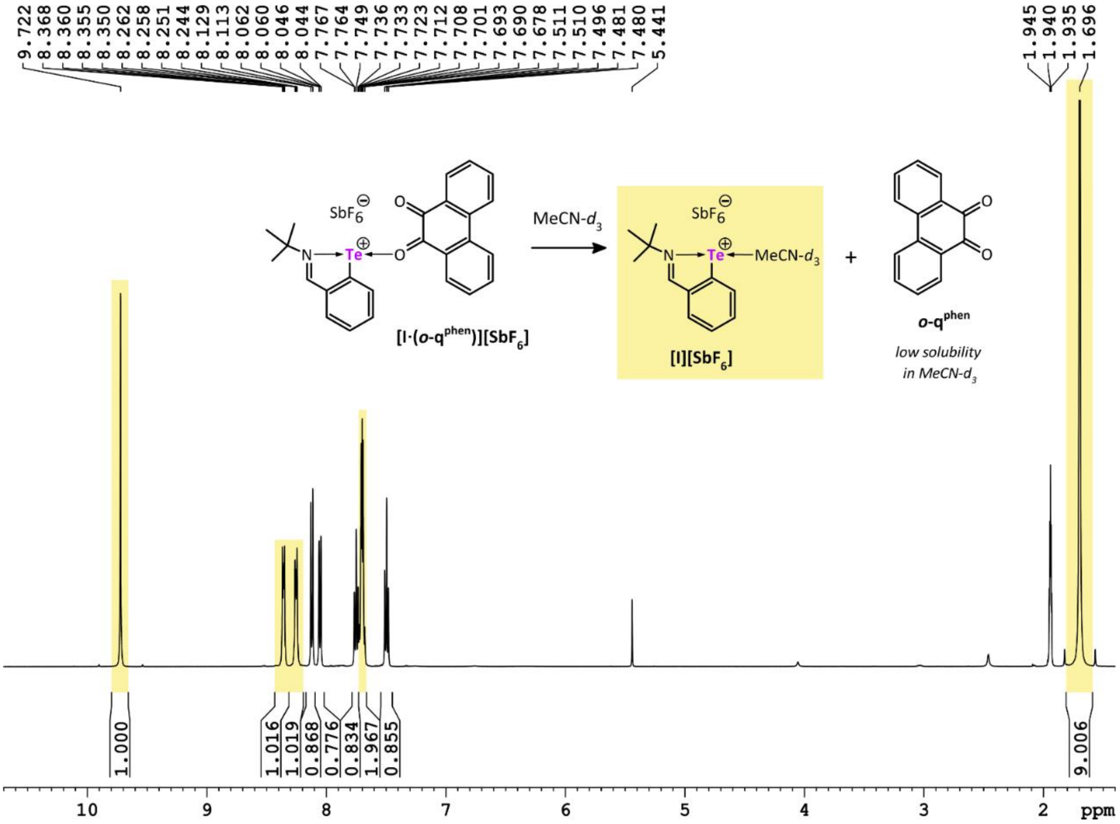

Figure S52: ^{1}H NMR spectrum obtained after dissolving single-crystals of **[I·(*o*-q^phen^)][SbF$_6$]** in MeCN-*d*$_3$ forming light orange suspension of **[I·(MeCN-*d*$_3$)][SbF$_6$]** and free ***o*-q^phen^** (which partially precipitated from the solution, thus ratio is no longer 1:1) (500 MHz, 295 K).

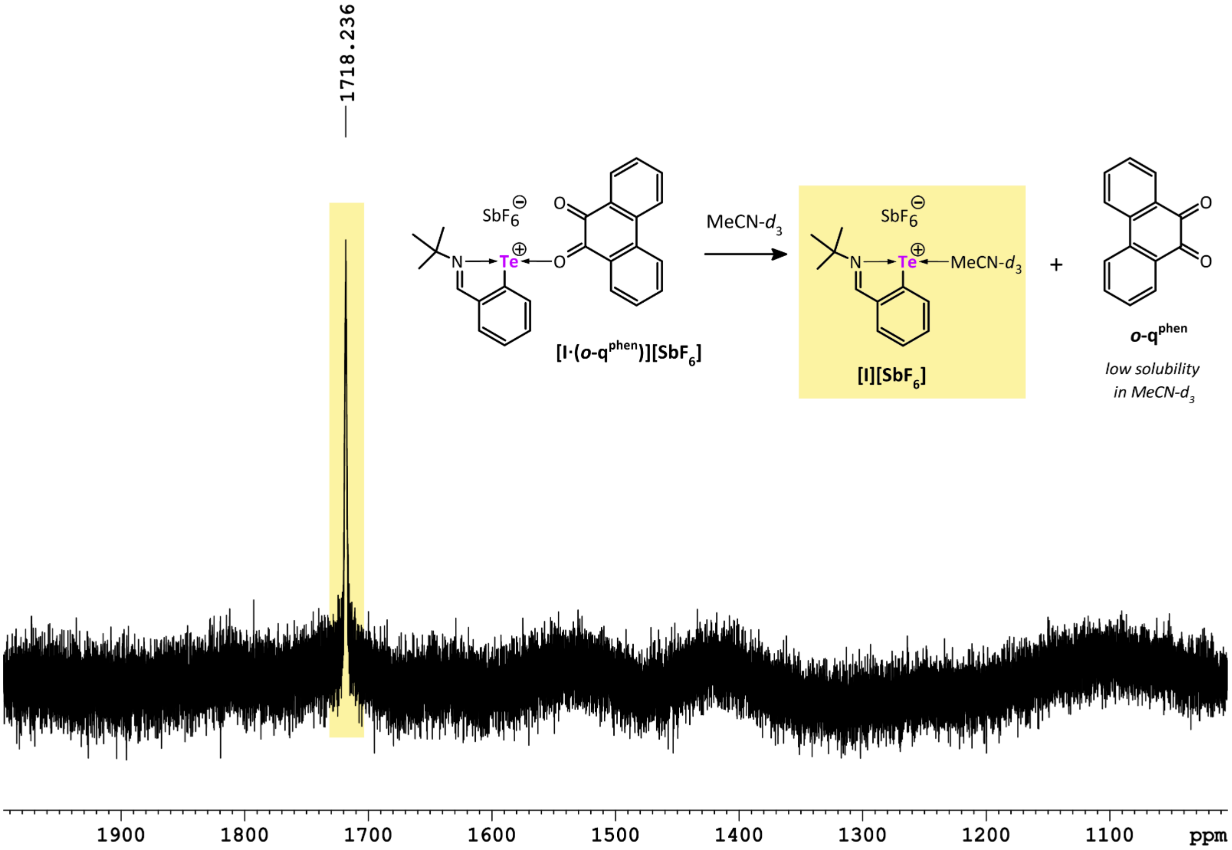

Figure S53: ^{125}**Te{**^{1}**H} NMR** spectrum obtained after dissolving single-crystals of **[I·(*o*-q^{phen})][SbF$_6$]** in MeCN-*d*$_3$ forming light orange suspension of **[I·(MeCN-*d*$_3$)][SbF$_6$]** and free ***o*-q^{phen}** (which partially precipitated from the solution, thus ratio is no longer 1:1) (157.7 MHz, 295 K, NS = 2000).

Reactions of [I]Cl, [I][OTf] and [I][SbF$_6$] with 1,2-cyclohexanedione (1,2-hde) and corresponding NMR spectra

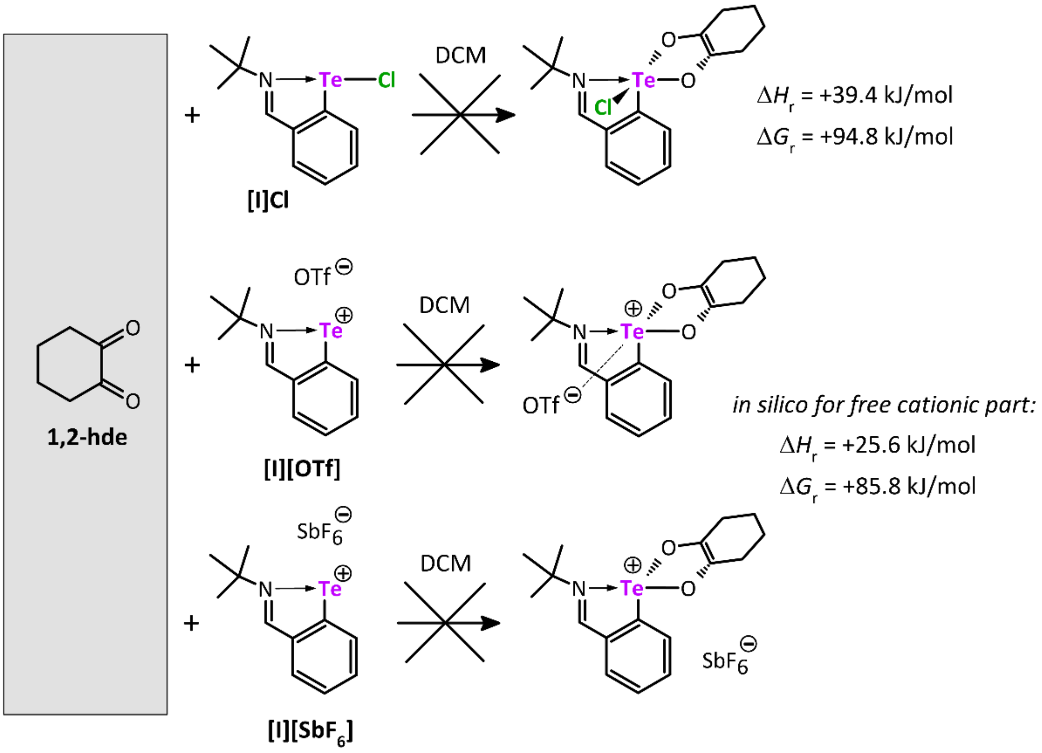

Scheme S1

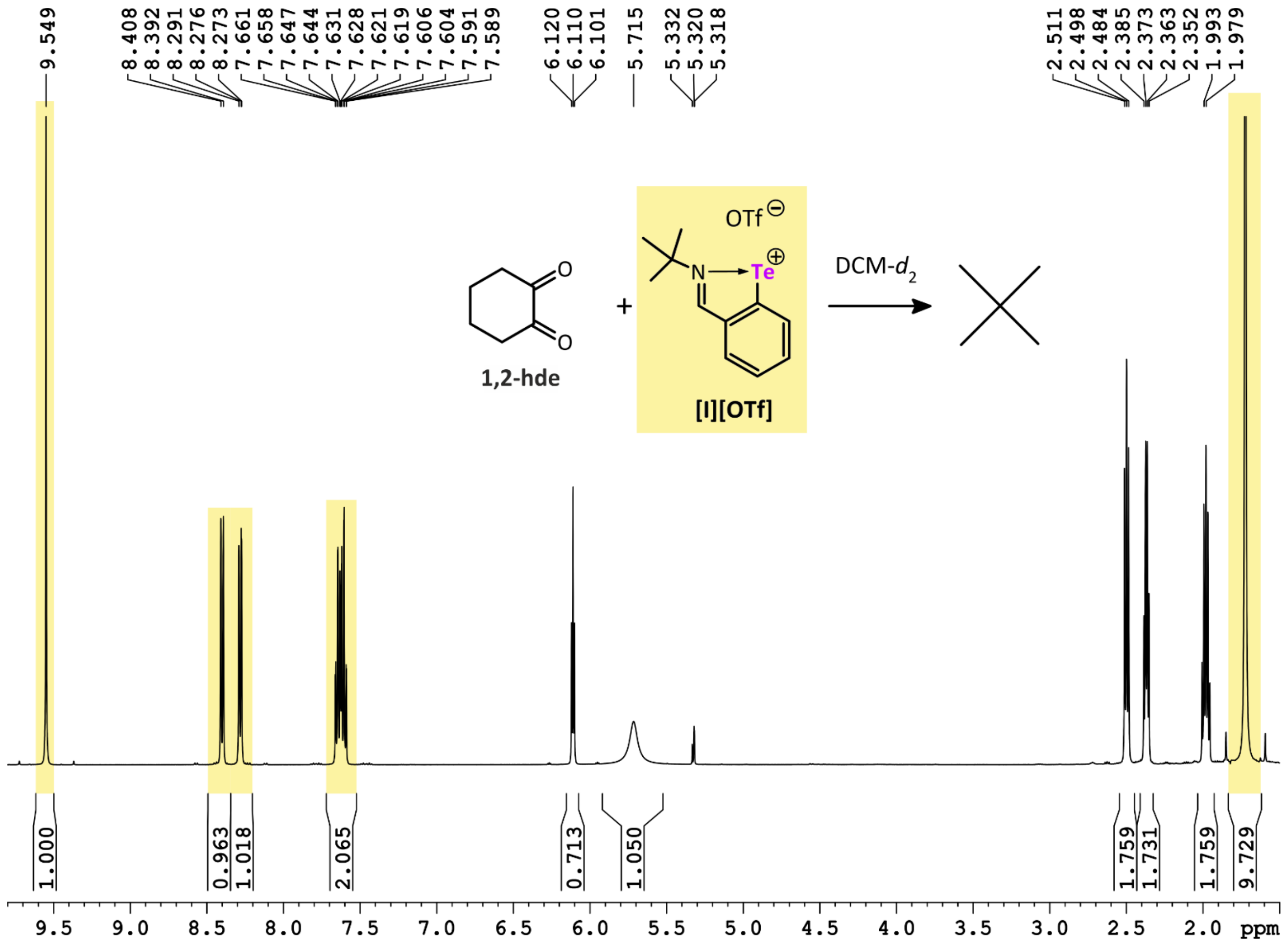

Figure S54: **^{1}H NMR** spectrum of 1:1 mixture of **[I][OTf]** and **1,2-hde** in DCM-d_2 showing no reaction (500 MHz, 295 K).

Catalyzed reactions of quinones with Et_3SiH using [I][OTf] as a catalyst

NMR spectra of compound 1

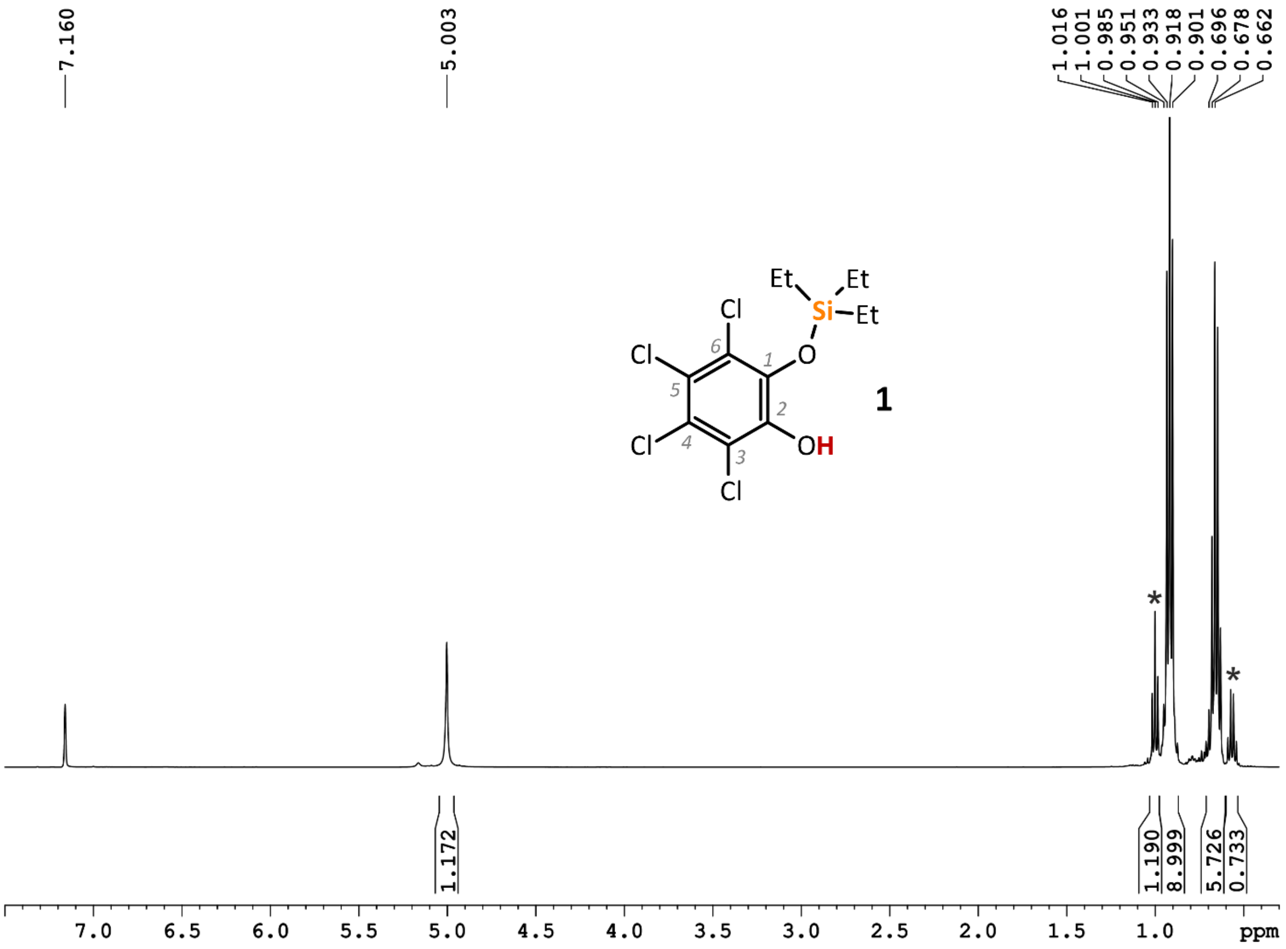

Figure S55: ^{1}H NMR spectrum of evaporated reaction mixture from catalysis producing compound **1** and dissolved in C_6D_6 (500.20 MHz, 295 K). * Denotes presence of $Et_3SiOSiEt_3$ due to hydrolysis.

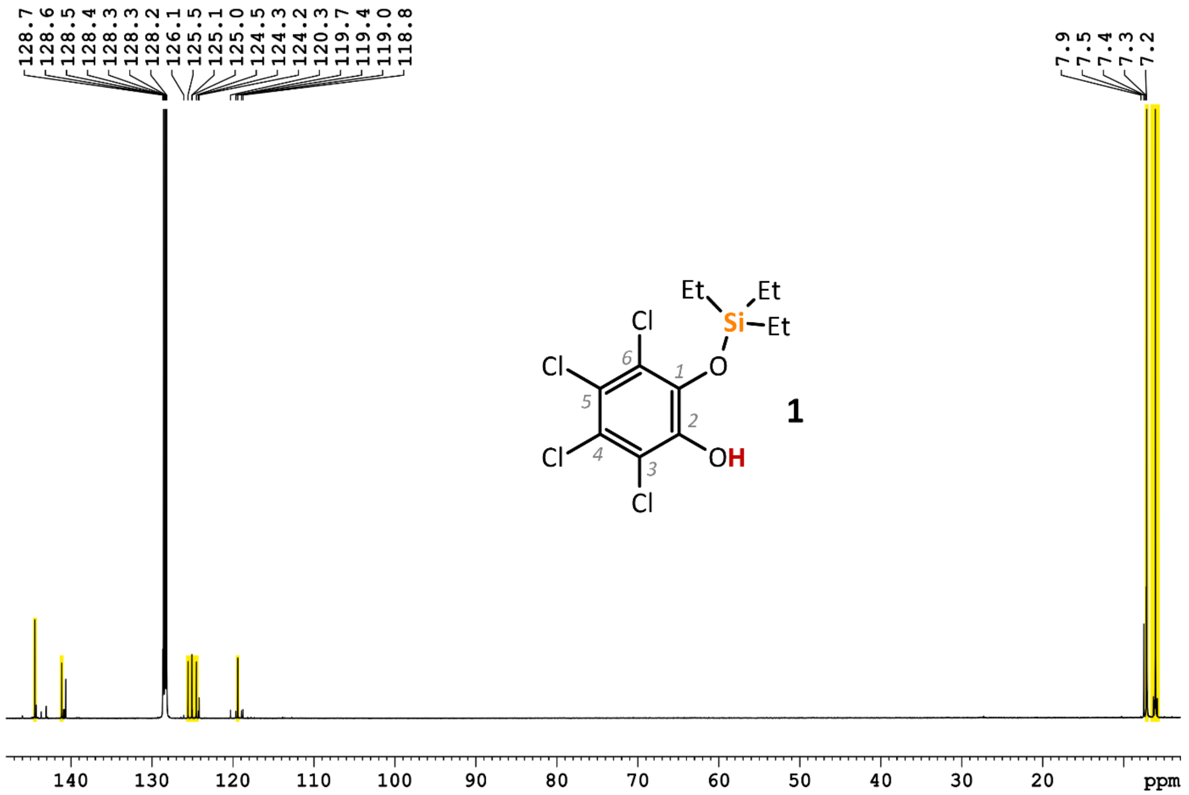

Figure S56: $^{13}C\{^{1}H\}$ NMR spectrum of evaporated reaction mixture from catalysis producing compound **1** and dissolved in C_6D_6 (125.78 MHz, 295 K). Signals of compound **1** are highlighted in yellow colour. Among the rest of presented minor signals, signals for hydrolysed corresponding catechol derivative can be distinguished.

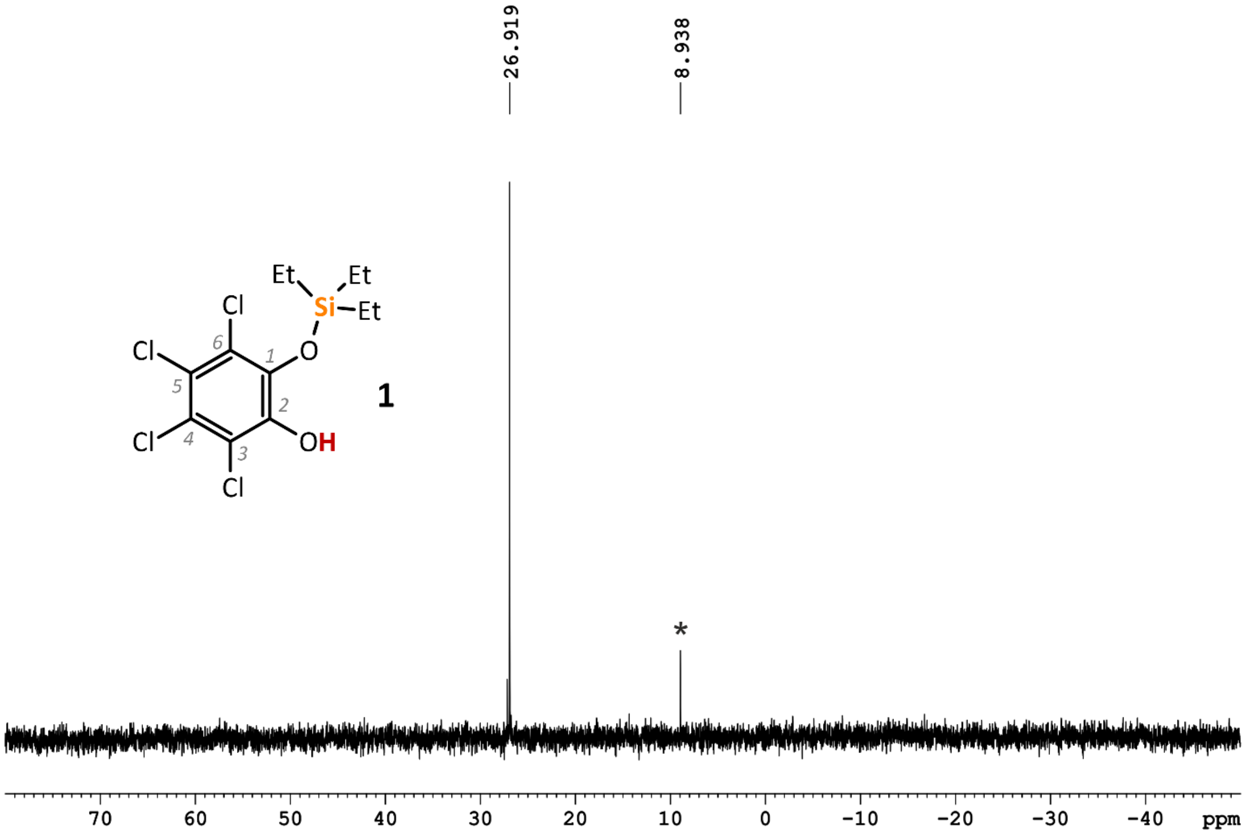

Figure S57: $^{29}Si\{^{1}H\}$ NMR spectrum of evaporated reaction mixture from catalysis producing compound **1** and dissolved in C_6D_6 (99.37 MHz, 295 K). * Denotes presence of $Et_3SiOSiEt_3$ due to hydrolysis.

NMR spectra of compound 2

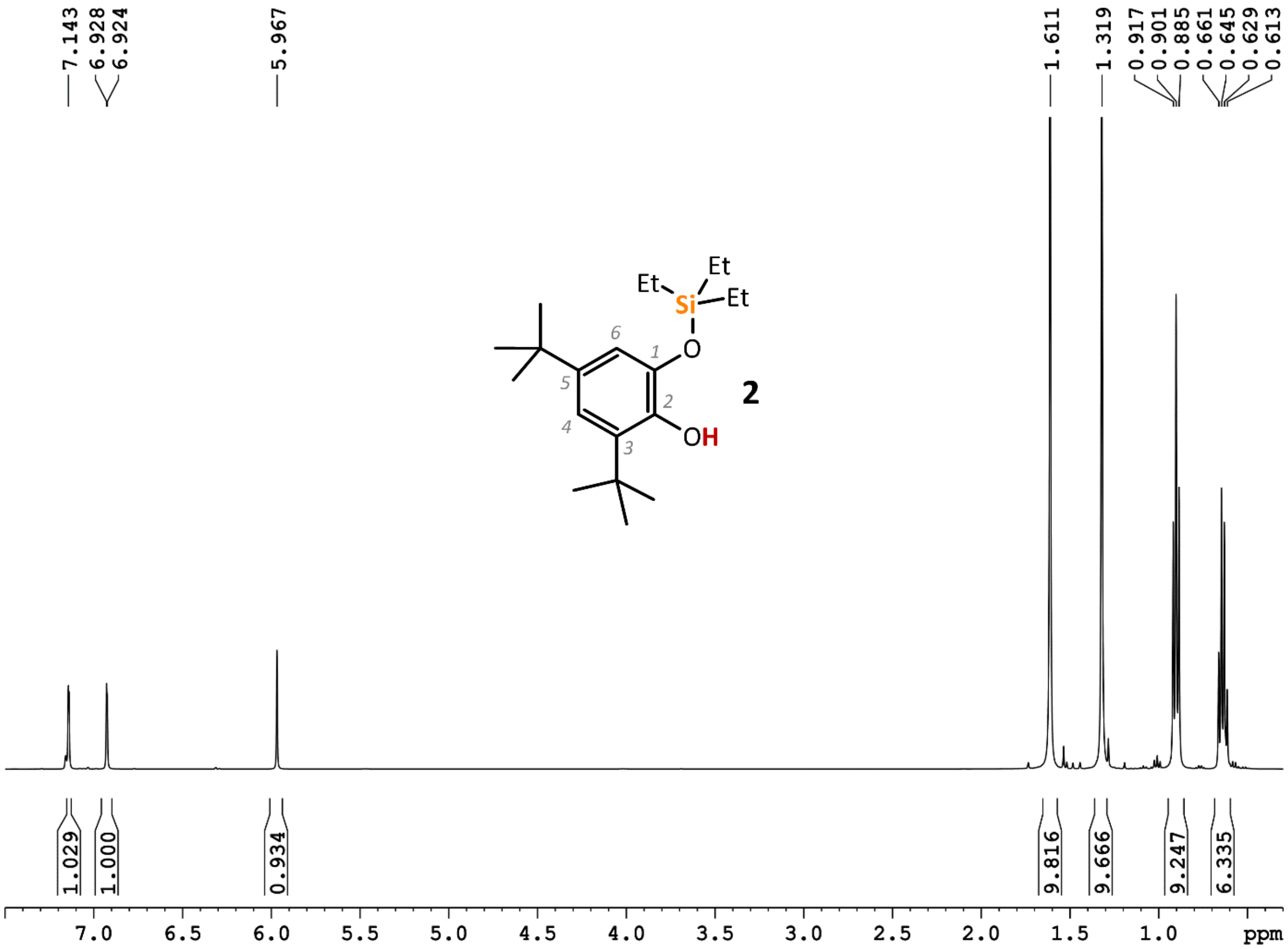

Figure S58: ^{1}H NMR spectrum of isolated compound **2** in C_6D_6 (500.20 MHz, 295 K).

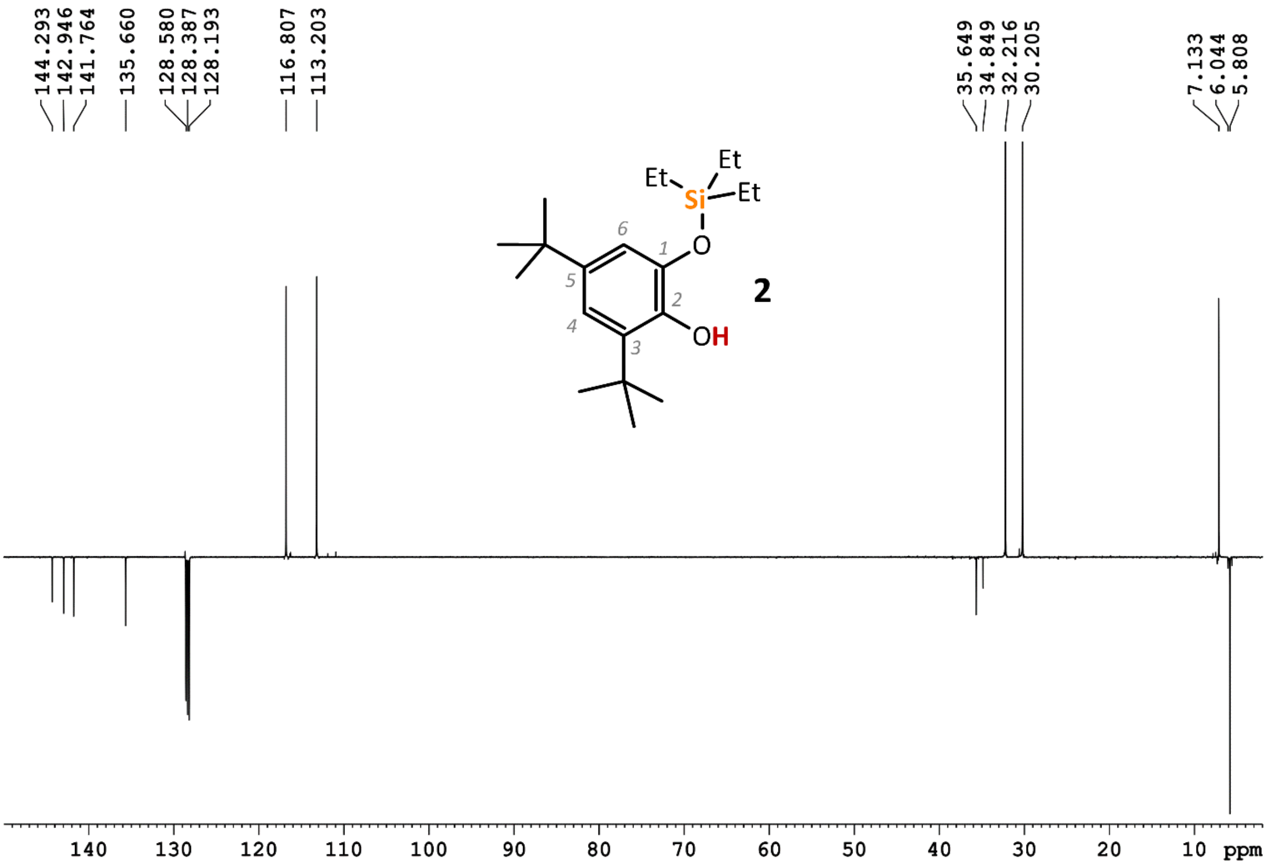

Figure S59: ^{13}**C{**^{1}**H} APT NMR** spectrum of isolated compound **2** in C_6D_6 (125.78 MHz, 295 K).

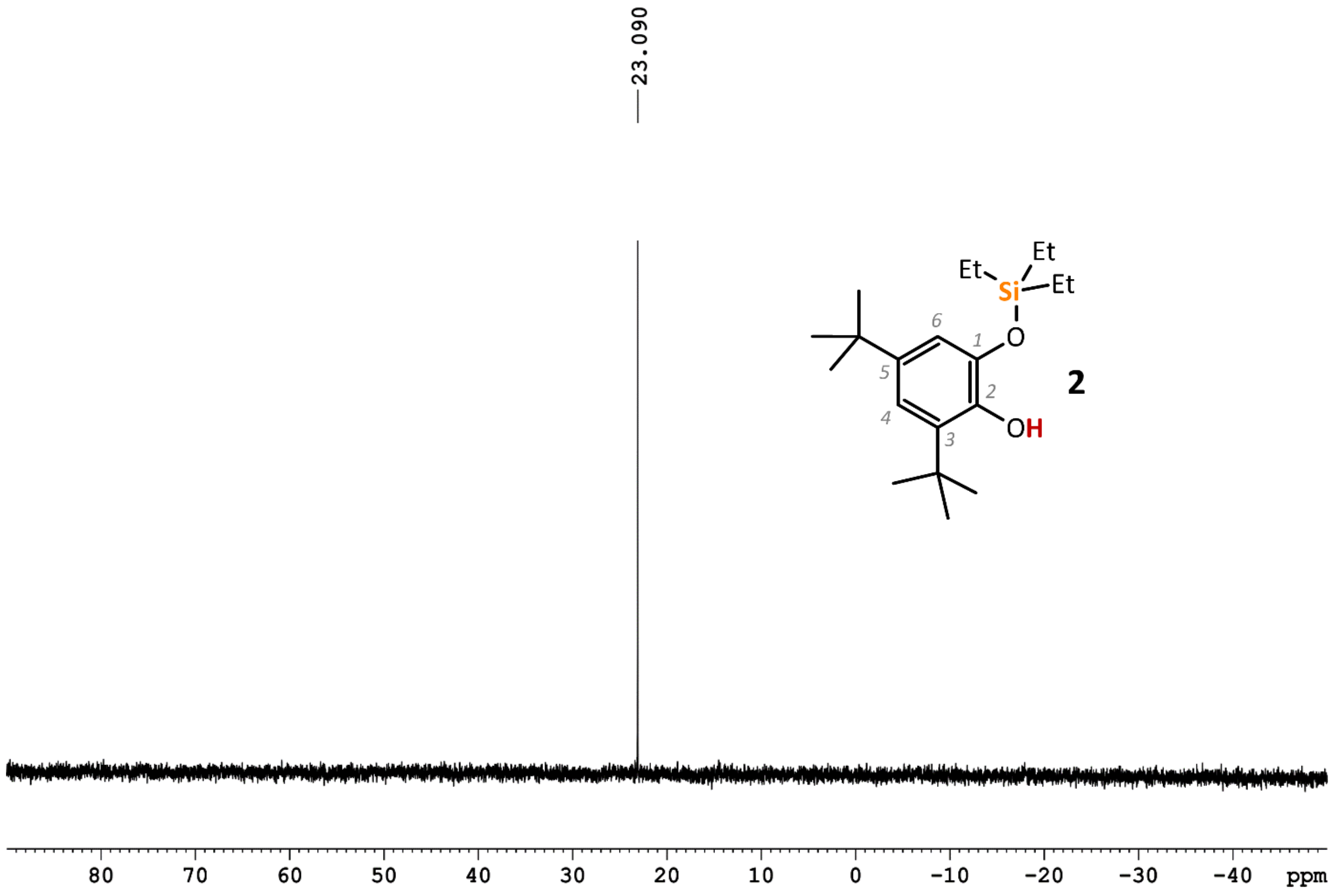

Figure S60: ^{29}**Si{**^{1}**H} NMR** spectrum of isolated compound **2** in C_6D_6 (99.37 MHz, 295 K).

NMR spectra of compounds 3 and 3’

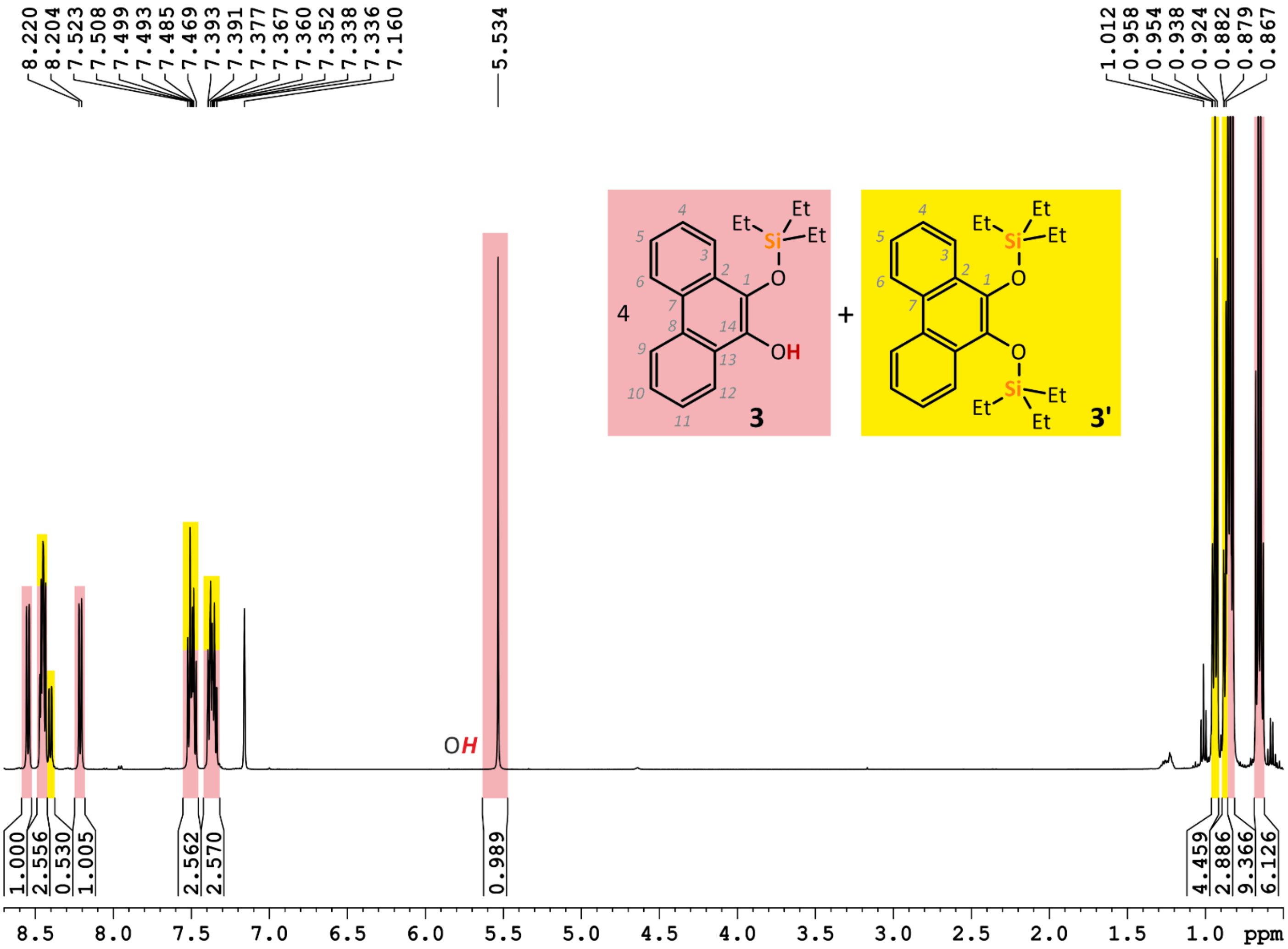

Figure S61: ^{1}H NMR spectrum of mixture of compounds **3** and **3’** (4:1 molar ratio) in C_6D_6 (500.20 MHz, 295 K).

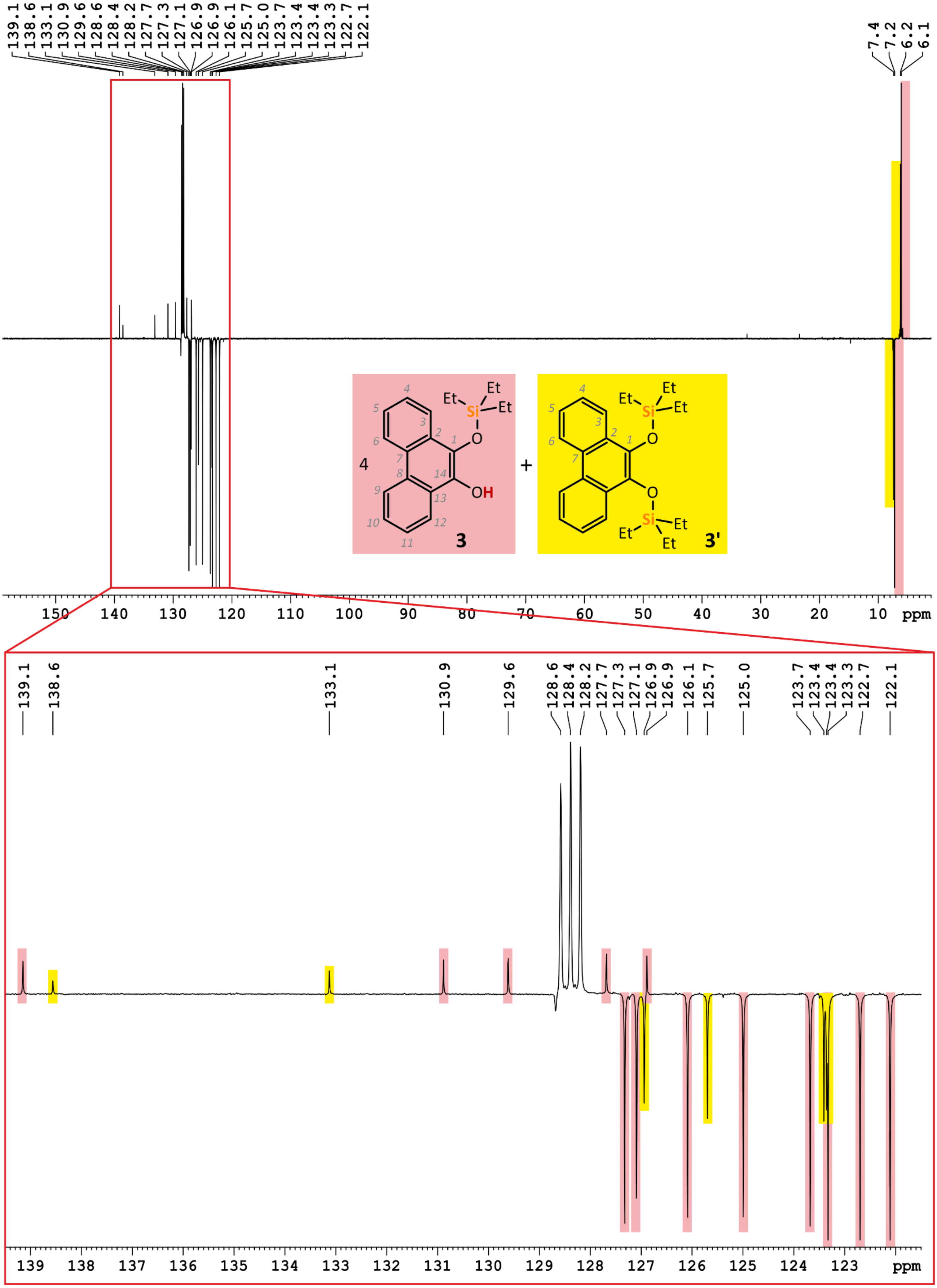

Figure S62: ^{13}**C{**^{1}**H} APT NMR** spectrum of compounds **3** and **3'** (4:1 molar ratio) in C_6D_6 (125.78 MHz, 295 K).

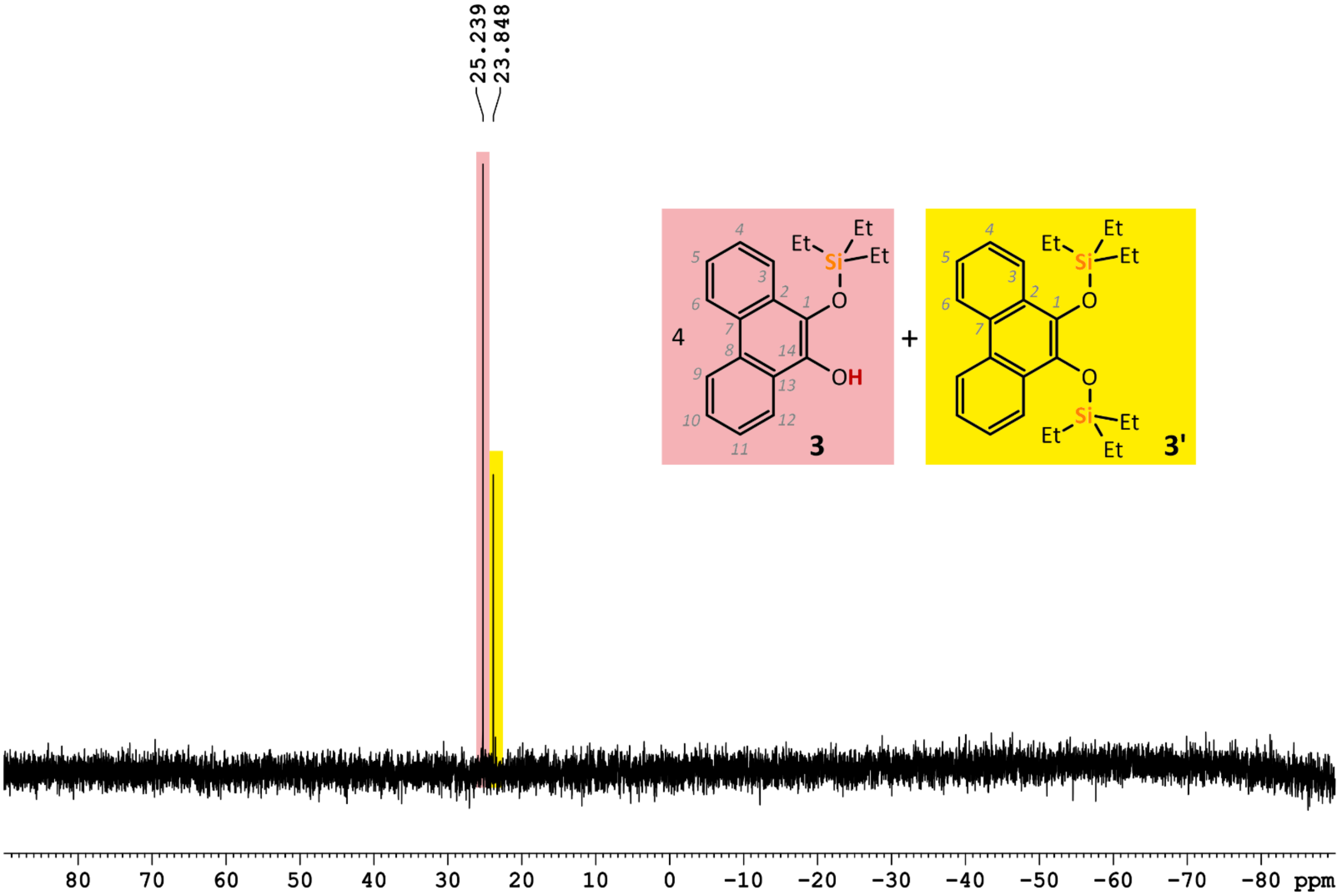

Figure S63: ^{29}**Si{^{1}H}** spectrum of isolated compounds **3** and **3'** (4:1 molar ratio) in C_6D_6 (99.37 MHz, 295 K).

NMR spectra of compound 4

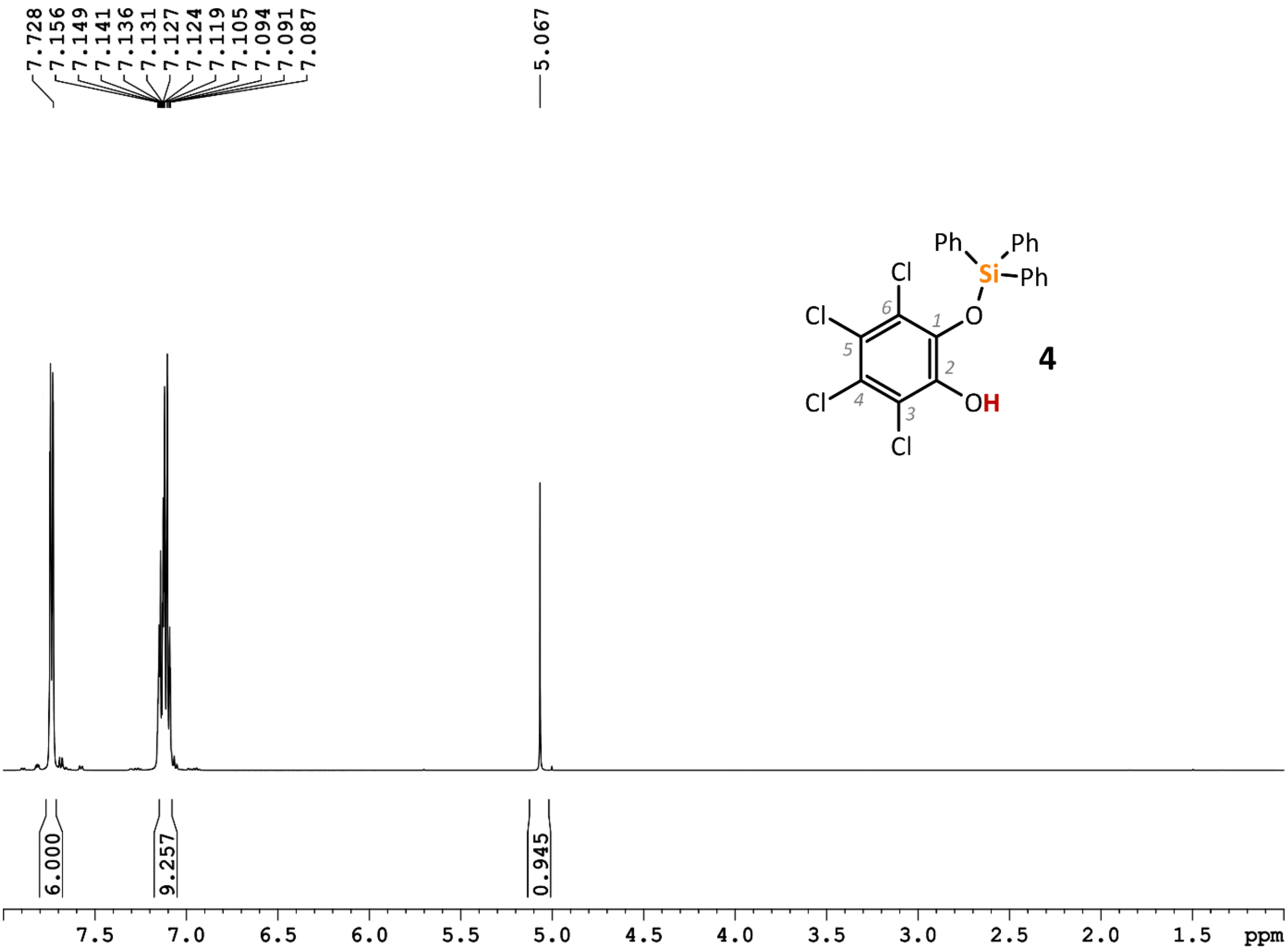

Figure S64: **^{1}H NMR** spectrum of isolated compound **4** in C_6D_6 (500.20 MHz, 295 K).

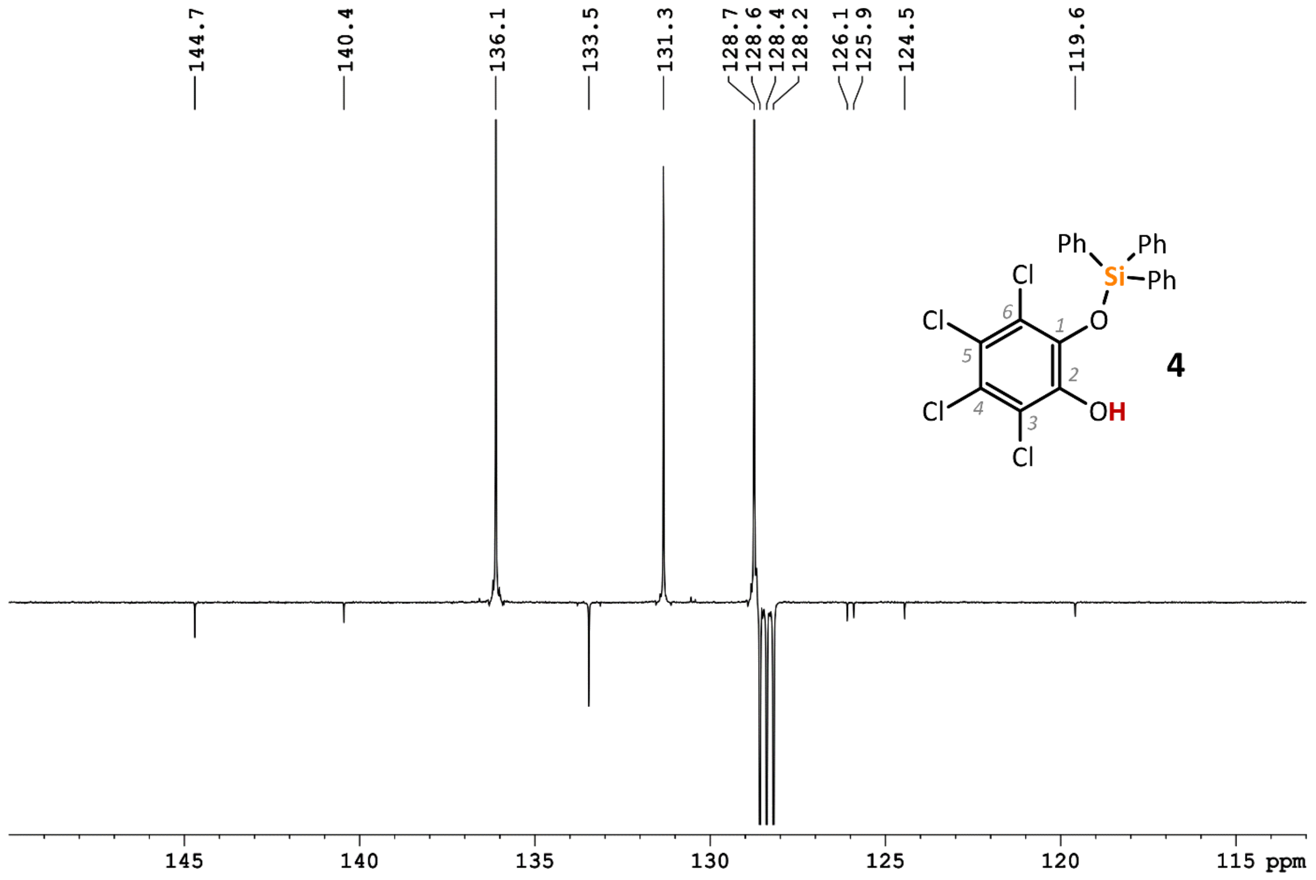

Figure S65: ^{13}**C{^{1}H} APT NMR** spectrum of isolated compound **4** in C_6D_6 (125.78 MHz, 295 K).

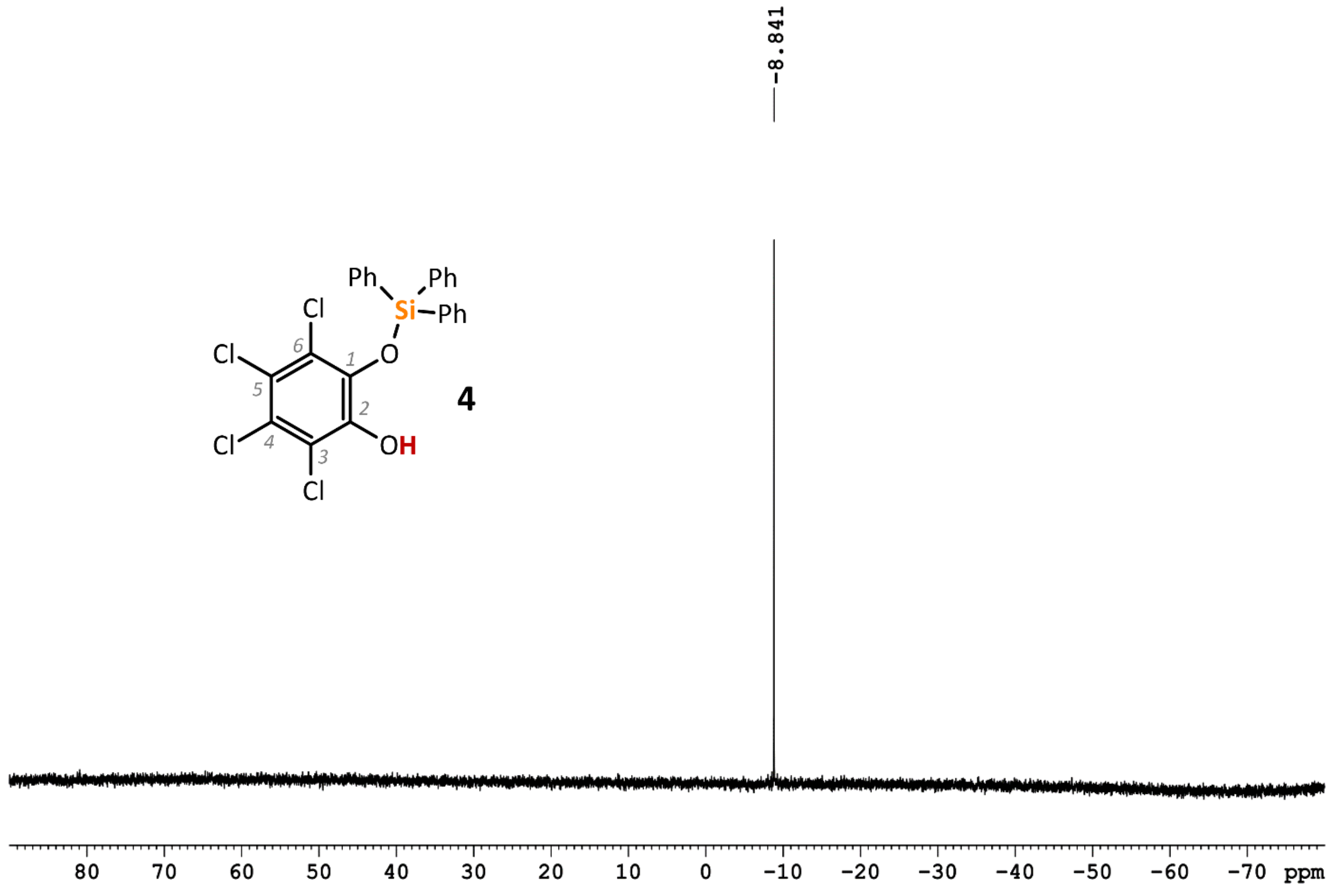

Figure S66: ^{29}**Si{^{1}H}** spectrum of isolated compound **4** in C_6D_6 (99.37 MHz, 295 K).

NMR spectra of compound 5

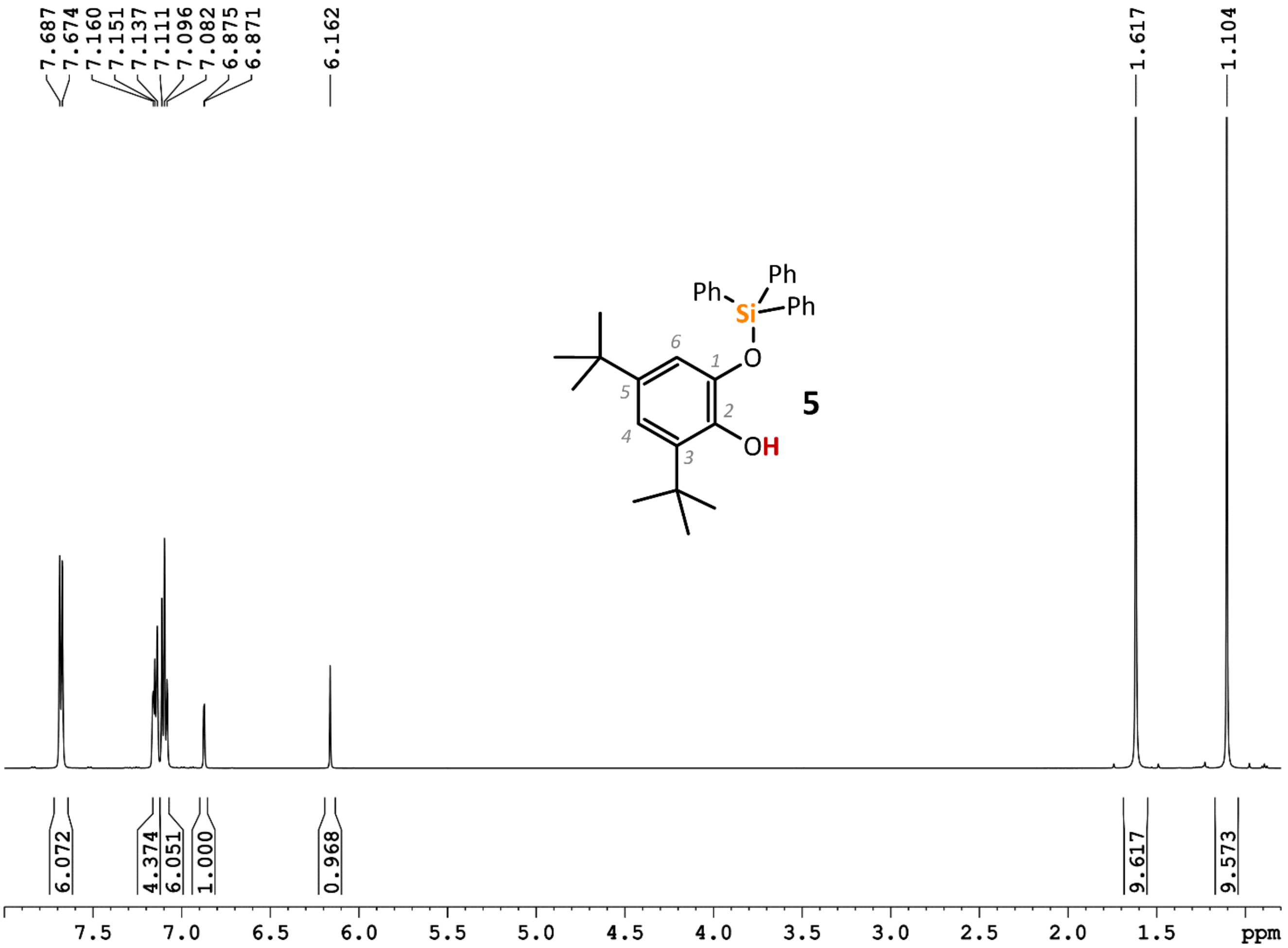

Figure S67: ^{1}H NMR spectrum of isolated compound **5** in C_6D_6 (500.20 MHz, 295 K).

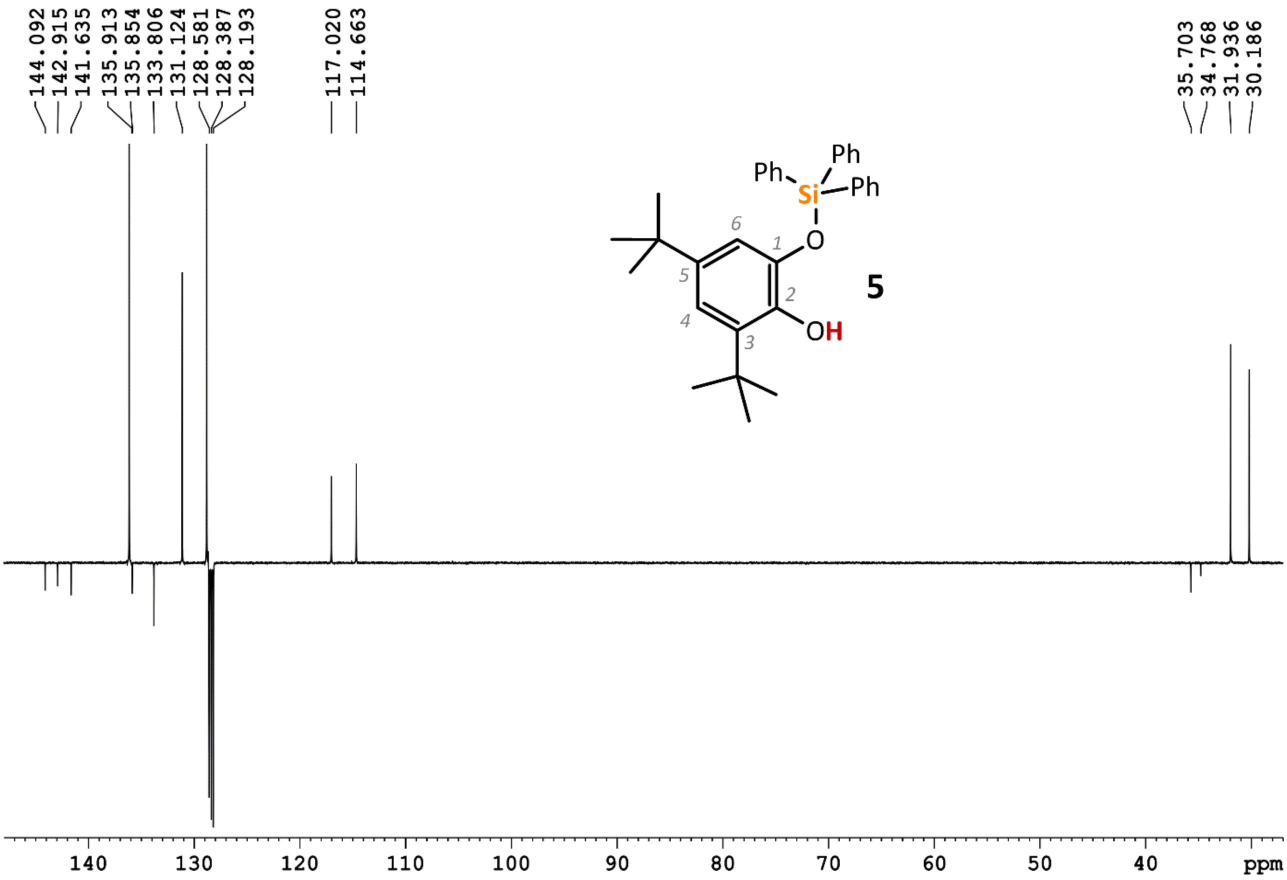

Figure S68: **$^{13}C\{^1H\}$ APT NMR** spectrum of isolated compound **5** in C_6D_6 (125.78 MHz, 295 K).

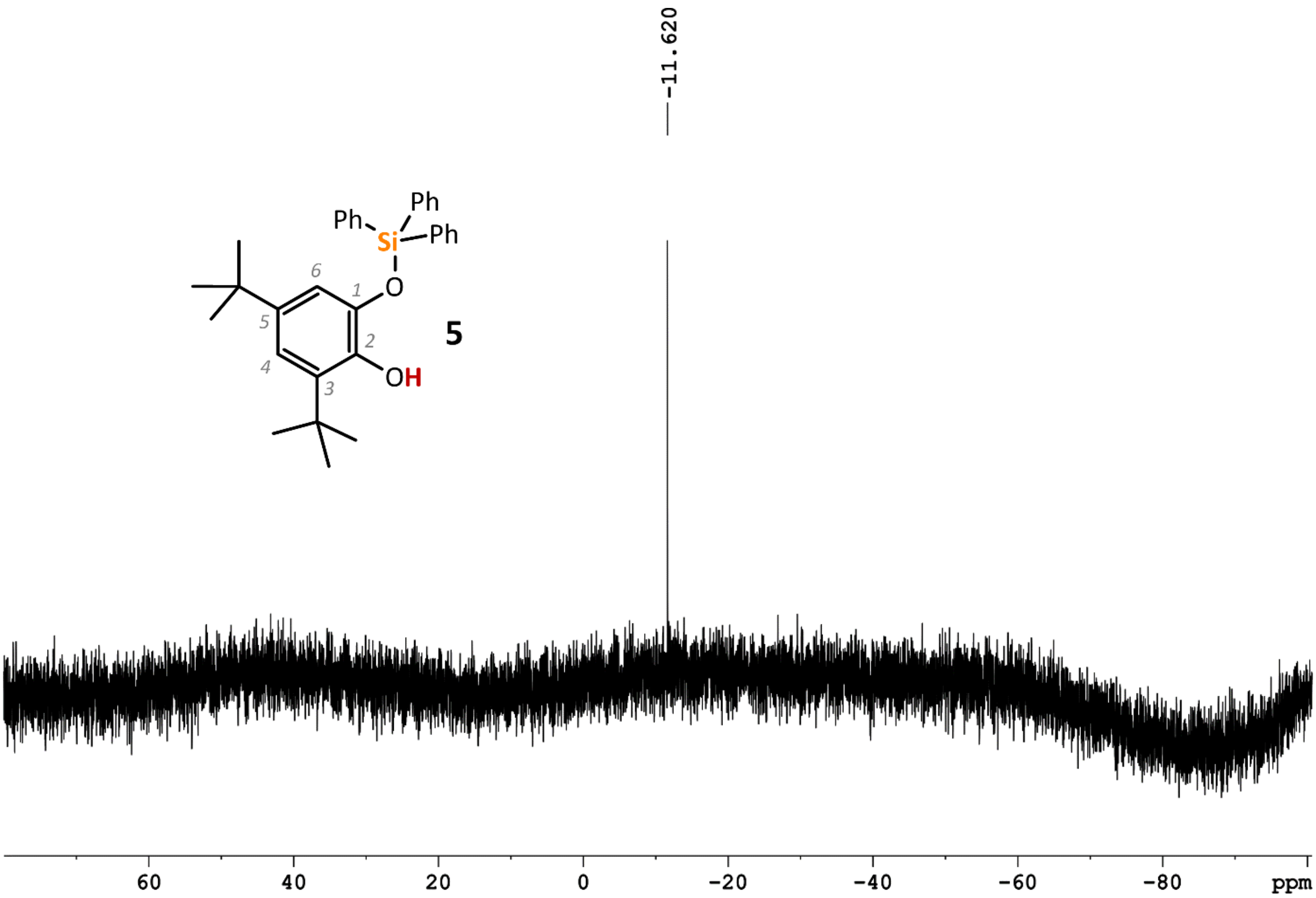

Figure S69: **$^{29}Si\{^1H\}$** spectrum of isolated compound **5** in C_6D_6 (99.37 MHz, 295 K).

NMR spectra of compound 6

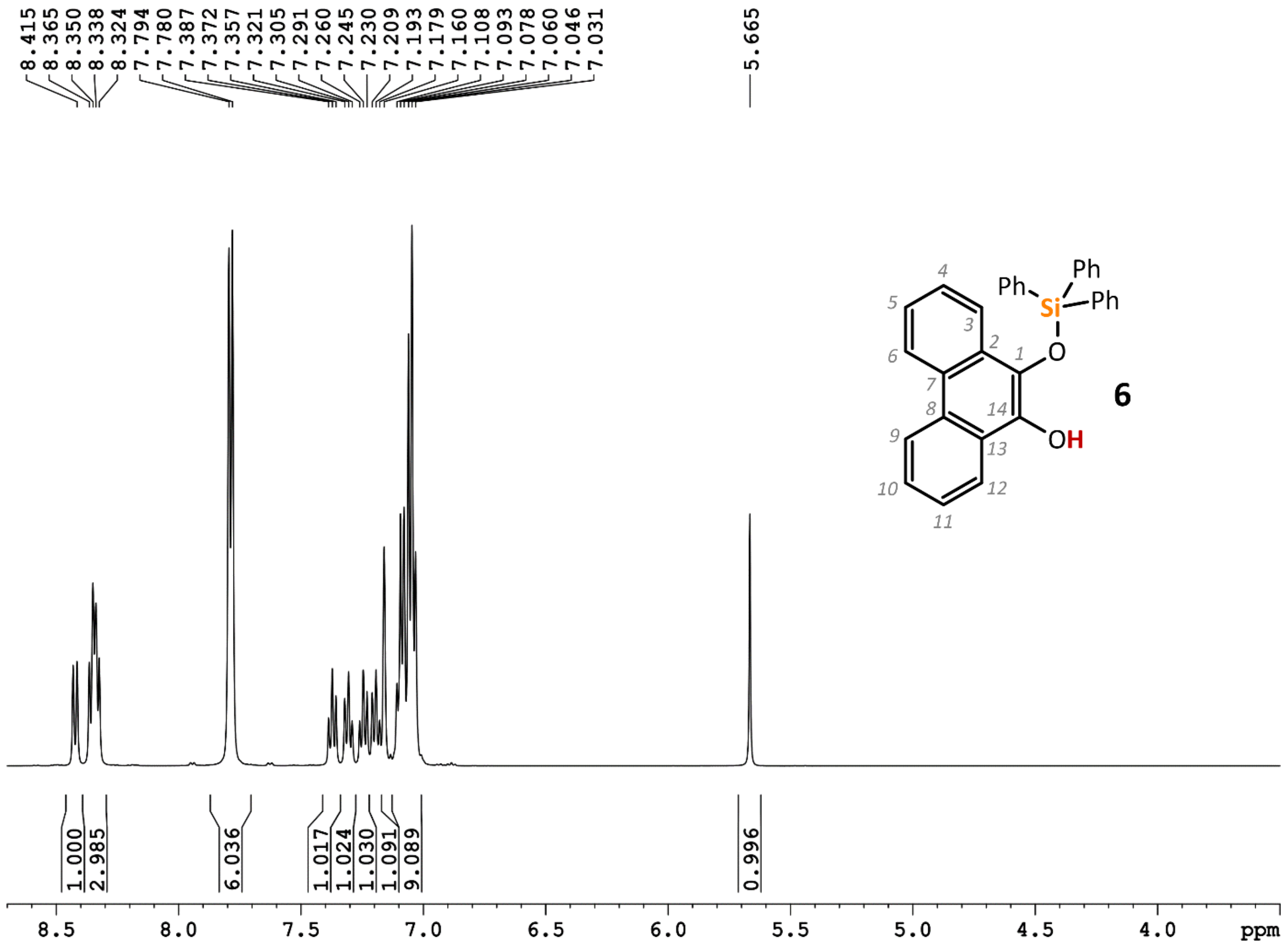

Figure S70: **^{1}H NMR** spectrum of isolated compound **6** in C_6D_6 (500.20 MHz, 295 K).

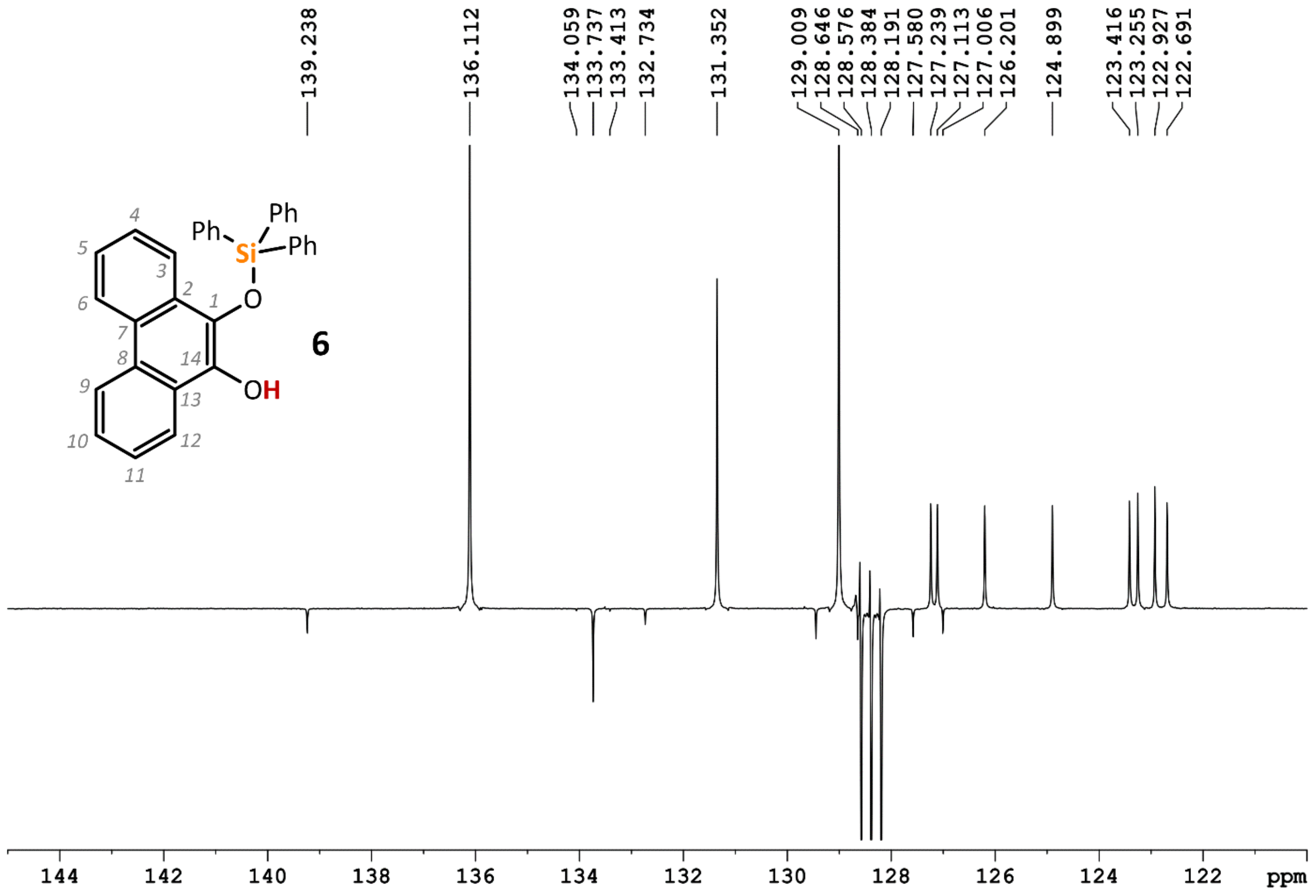

Figure S71: $^{13}C\{^{1}H\}$ APT NMR spectrum of isolated compound **6** in C_6D_6 (125.78 MHz, 295 K).

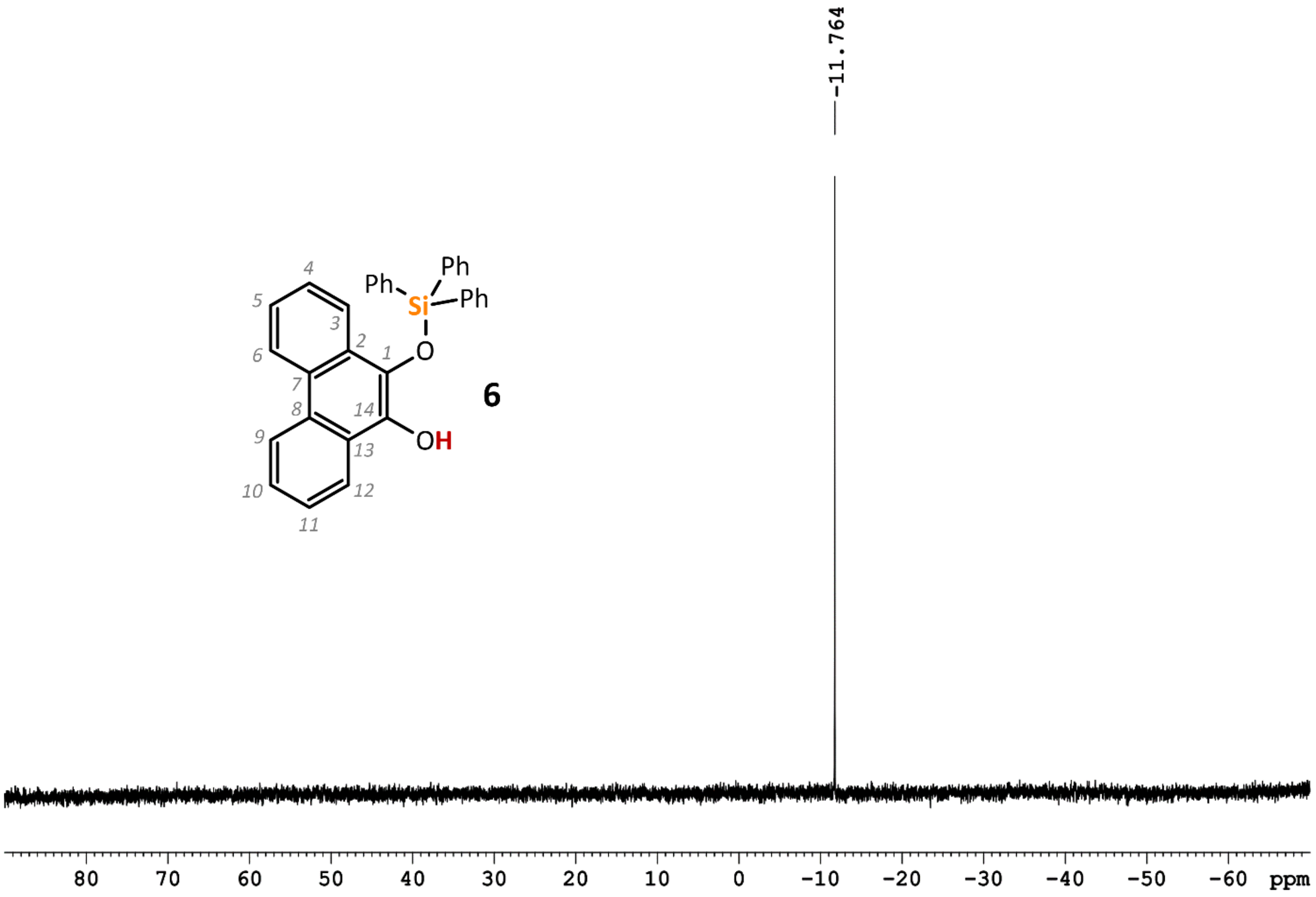

Figure S72: $^{29}Si\{^{1}H\}$ spectrum of isolated compound **6** in C_6D_6 (99.37 MHz, 295 K).

NMR spectra for reaction of o-q^{Cl} with Et_3SiH leading to formation of 7

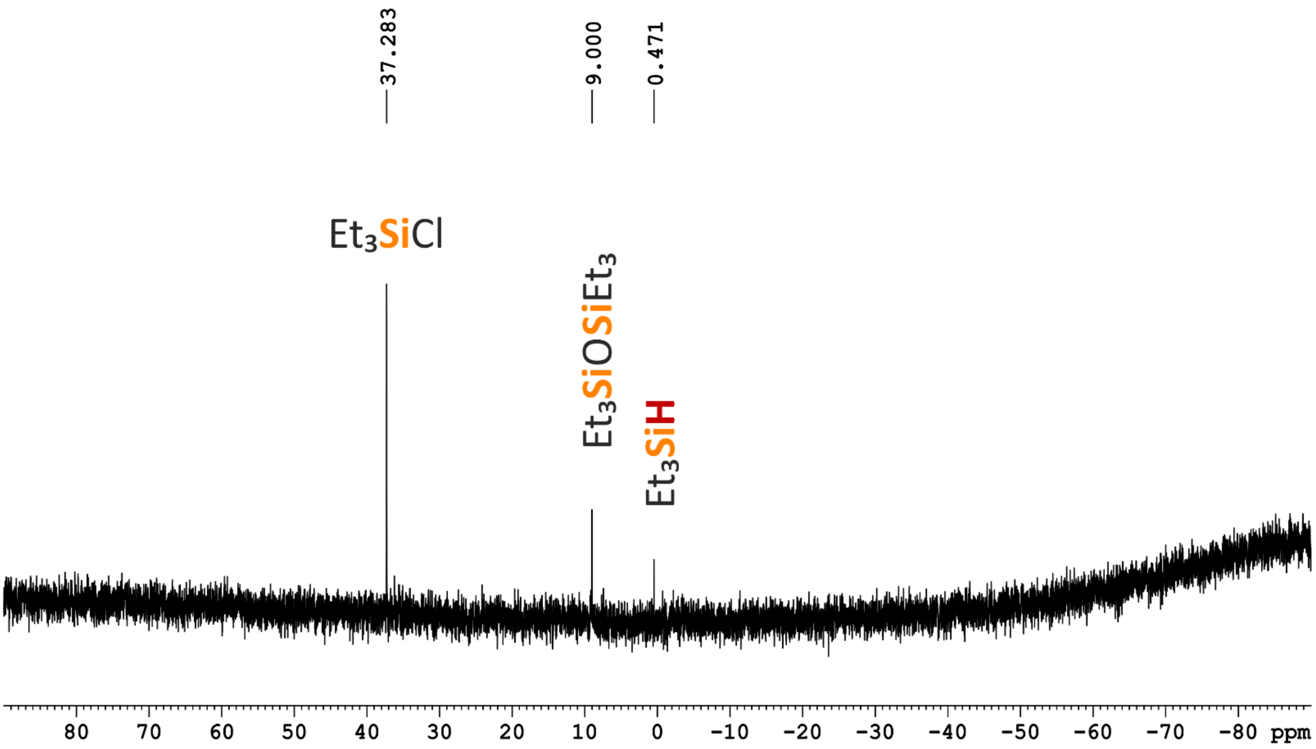

Figure S73: ^{29}Si{^{1}H} NMR spectrum of mother liquor of non-catalyzed reaction of ***o*-q^{Cl}** with Et_3SiH (99.37 MHz, 295 K). Minor presence of $Et_3SiOSiEt_3$ is a result of hydrolysis of Et_3SiCl/Et_3SiH.

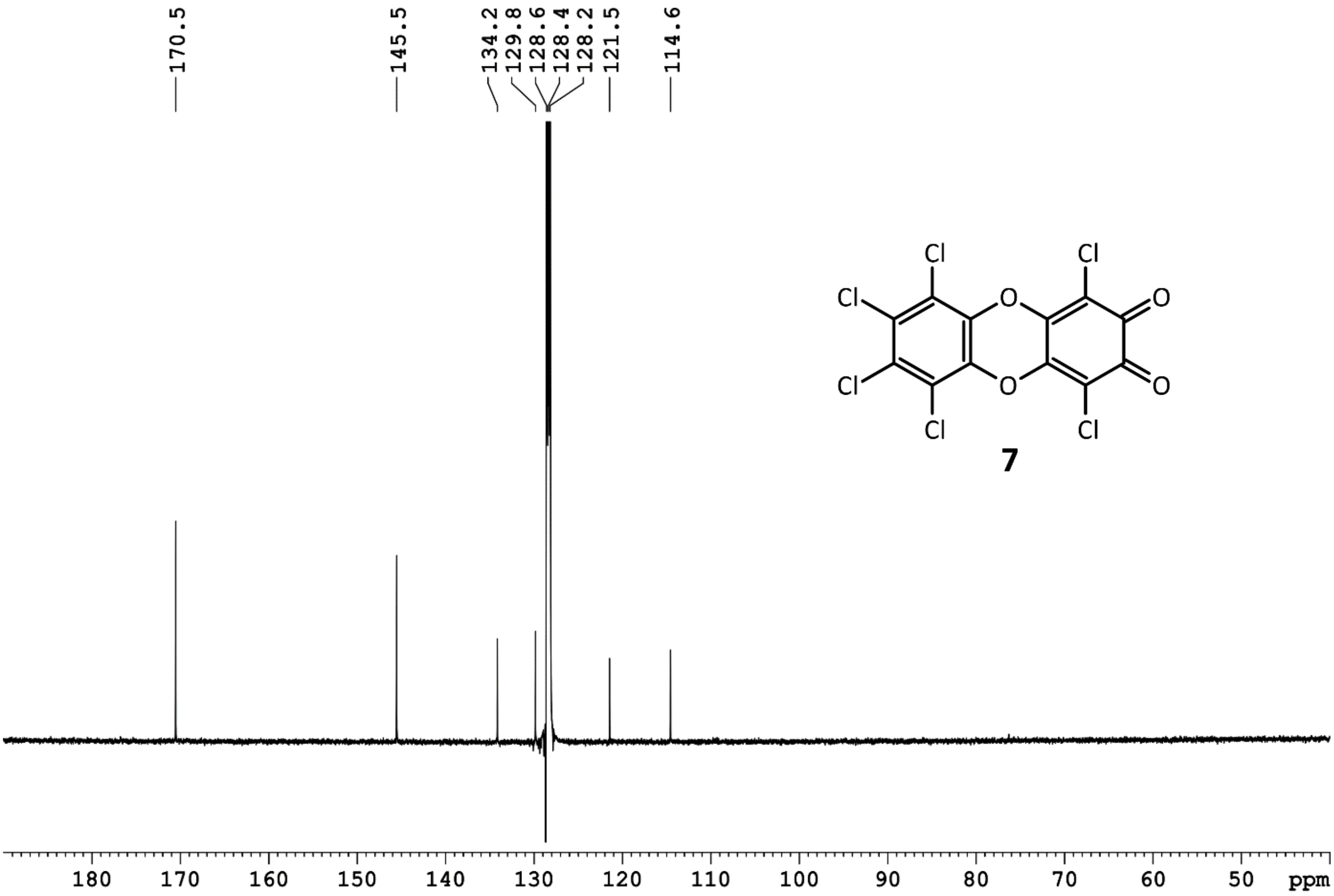

Figure S74: ^{13}C{^{1}H} APT NMR spectrum of isolated **7** in C_6D_6 (125.78 MHz, 295 K). **7** is poorly soluble in C_6D_6, thus C_6D_6 has the most intense signal in the spectra.

Non-reactivity of o-q^tBu with Et_3SiH

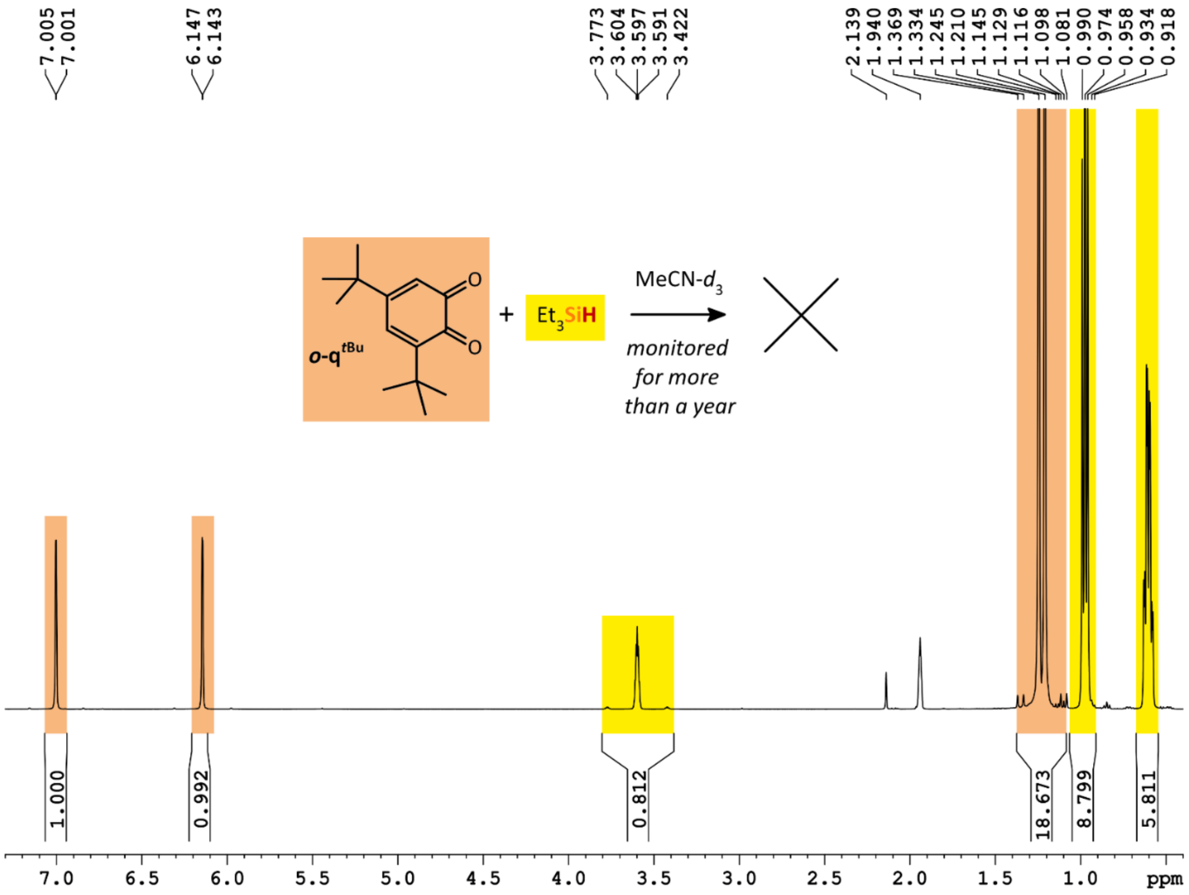

Figure S75: ^1H NMR spectrum of 1:1 mixture of ***o*-q^tBu** and Et_3SiH in MeCN-d_3 forming clear solution. This particular spectrum was acquired after 12 months of staying in the flame-sealed NMR tube at RT and then heated overnight for 95 °C and shows no signs of reaction (500 MHz, 295 K).

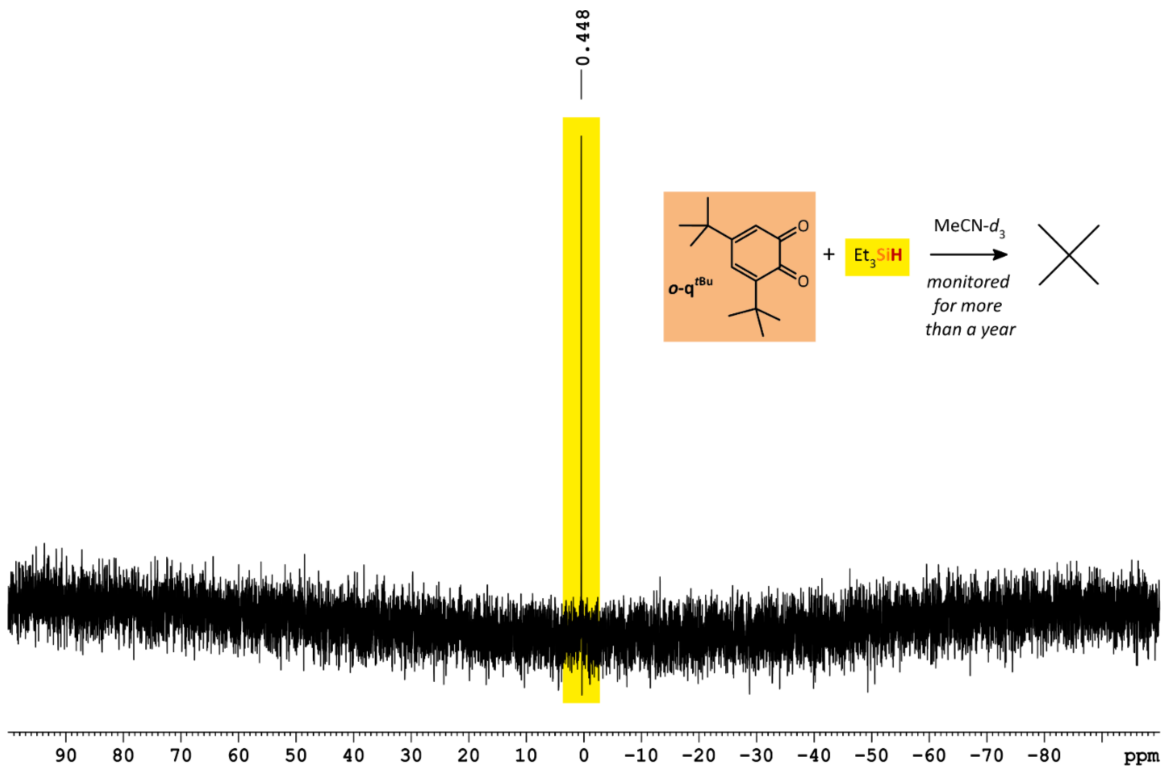

Figure S76: ^{29}Si{^{1}H} NMR spectrum of 1:1 mixture of ***o*-q^tBu** and Et_3SiH in MeCN-d_3 forming clear solution. This particular spectrum was acquired after 12 months of staying in the flame-sealed NMR tube at RT and then heated overnight for 95 °C and shows no signs of reaction (99.37 MHz, 295 K).

Non-catalyzed reaction of o-q^phen with Et_3SiH

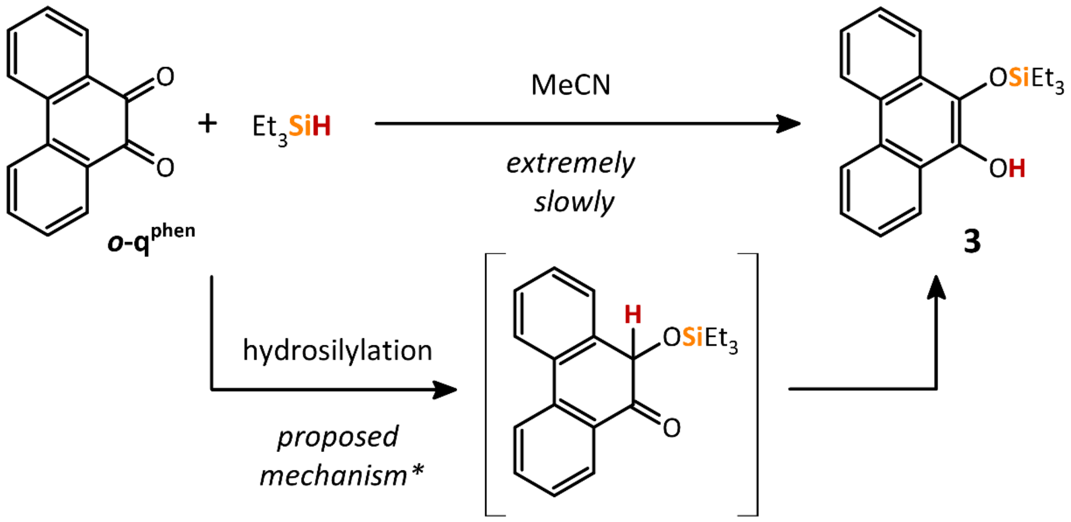

* based on a similar reaction described for the reaction of *ortho*-quinones with BH_3.[1]

45 mg (0.22 mmol) of ***o*-q^phen** was loaded into NMR tube and 0.6 mL of dry and degassed MeCN-d_3 was added under an argon atmosphere to form an orange suspension. Subsequently, 35 μL (0.22 mmol) of neat Et_3SiH was added and the NMR tube was flame-sealed. The NMR tube was placed on nutating mixer for a week and then analyzed by NMR (see spectra below).

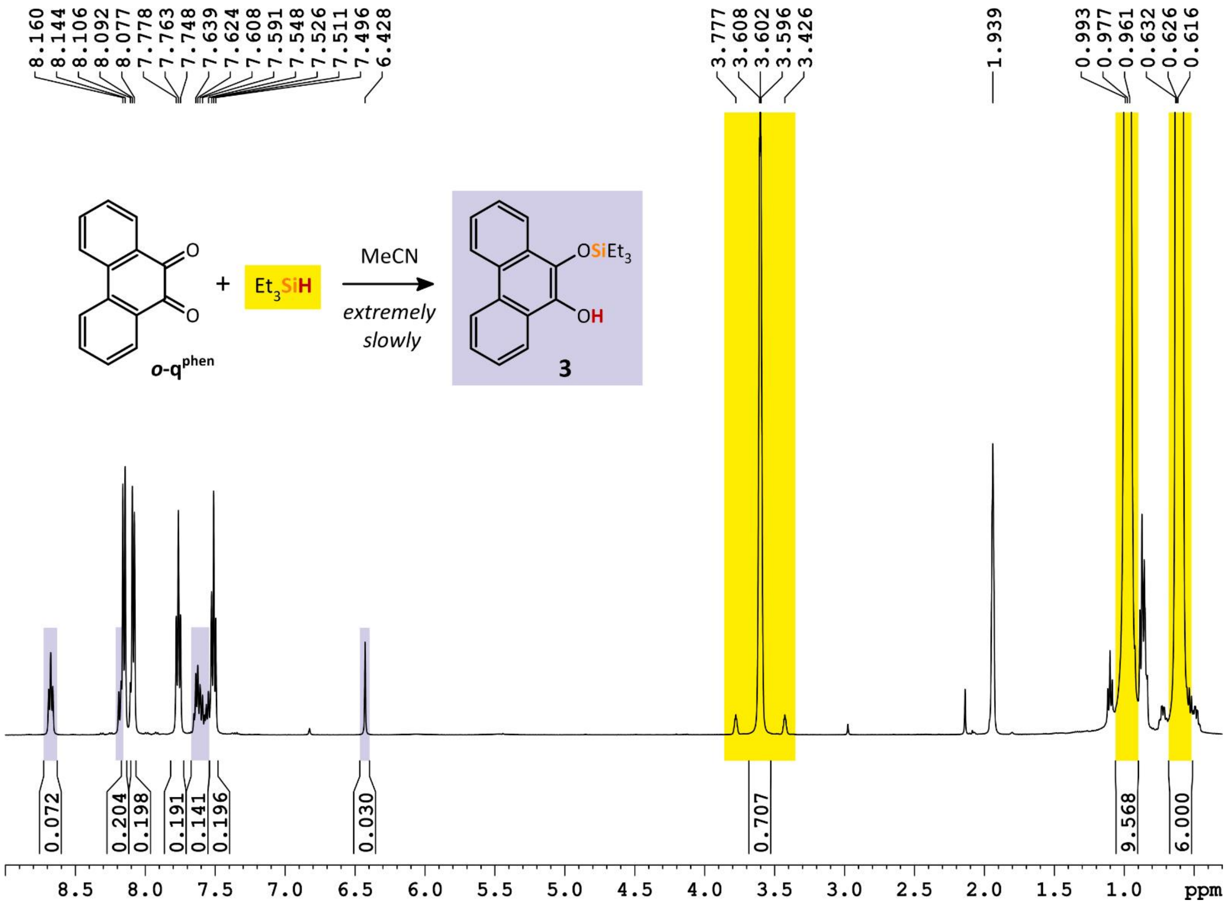

Figure S77: **^{1}H NMR** spectrum of reaction mixture (a suspension) of non-catalyzed reaction of ***o*-q^{phen}** with Et_3SiH (1:1 molar ratio) in MeCN-d_3 after a week of stirring at RT (500.20 MHz, 295 K). Signal of the product **3** is integrated against signal for ethyl group of Et_3SiH as ***o*-q^{phen}** is not fully dissolved.

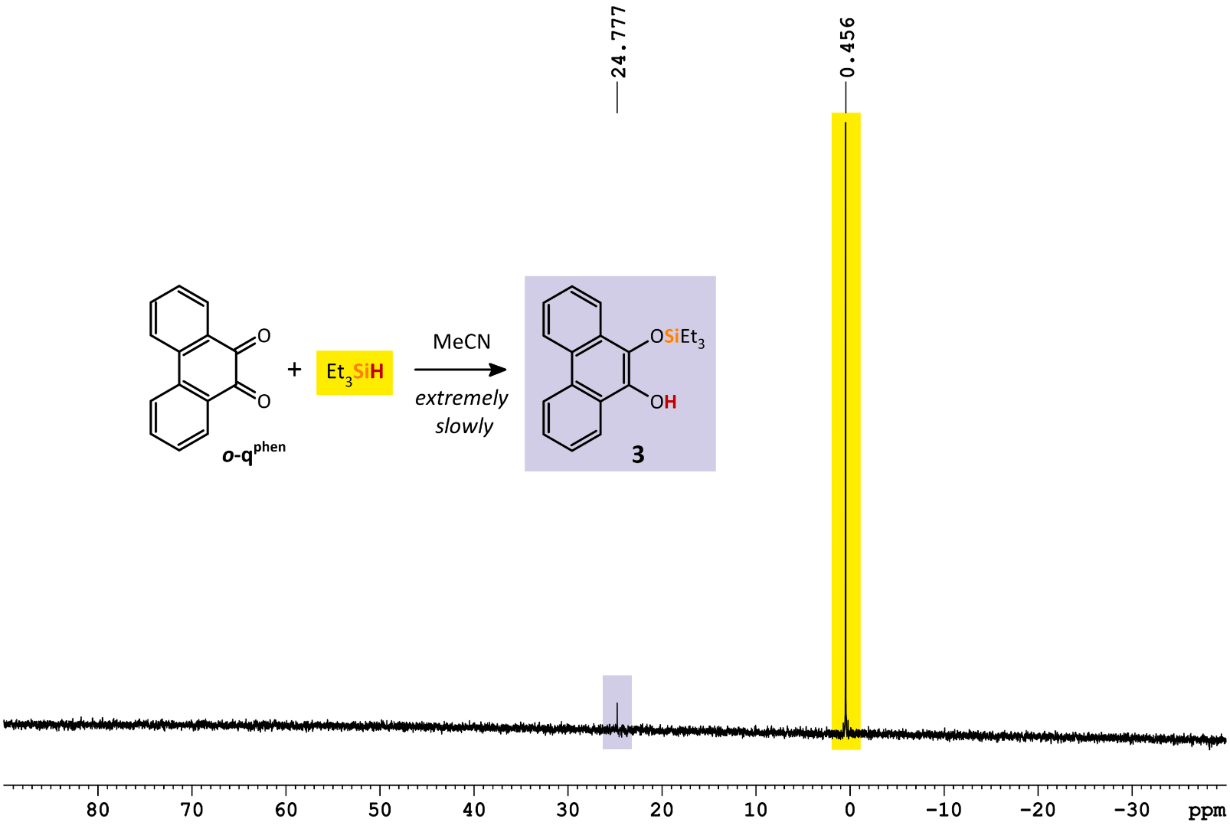

Figure S78: **^{29}Si{^{1}H} NMR** spectrum of reaction mixture (a suspension) of non-catalyzed reaction of ***o*-q^{phen}** with Et_3SiH (1:1 molar ratio) in MeCN-d_3 after a week of stirring at RT (99.37 MHz, 295 K).

NMR spectra of compound 8

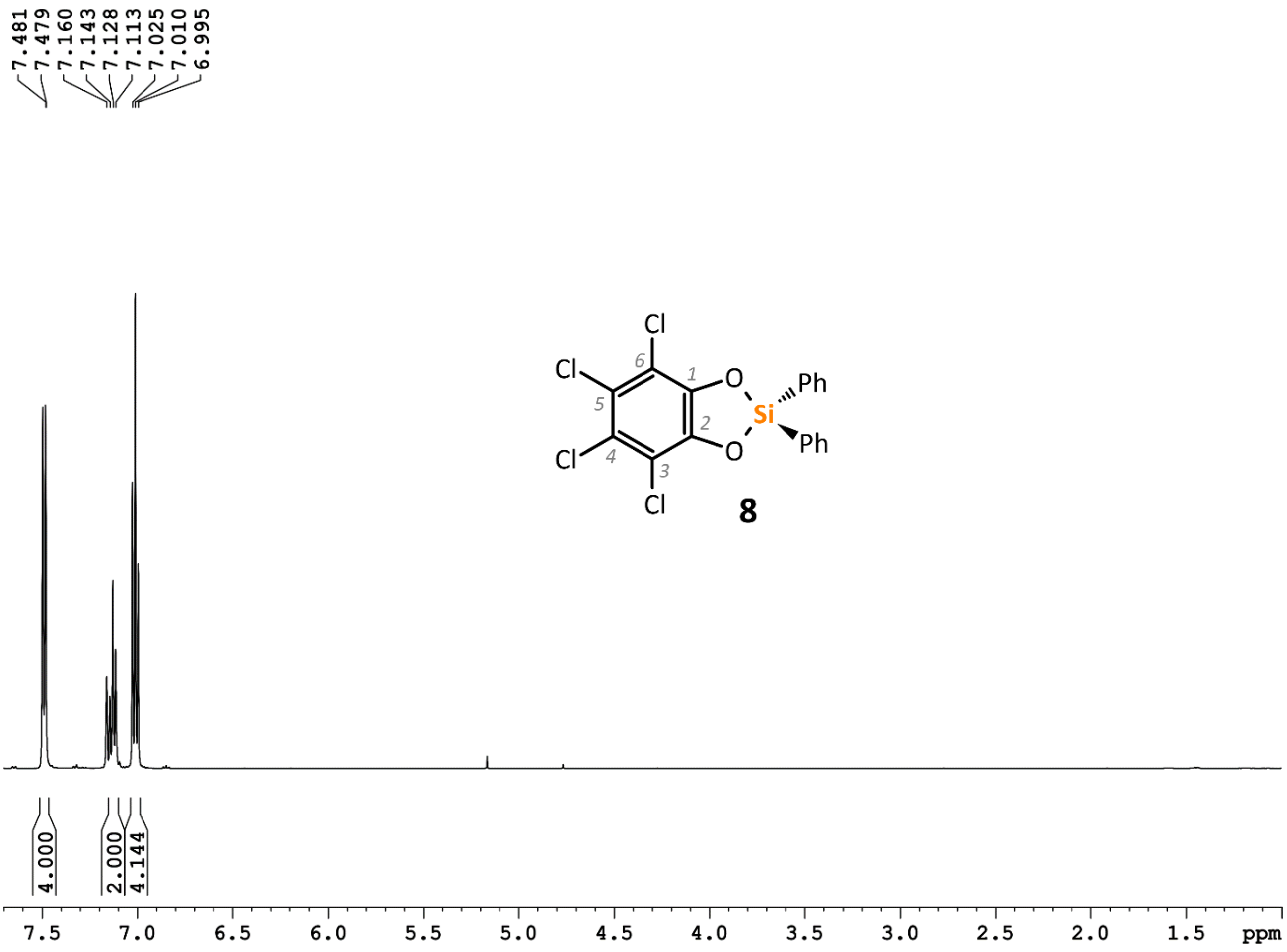

Figure S79: **^{1}H NMR** spectrum of isolated compound **8** in C_6D_6 (500.20 MHz, 295 K).

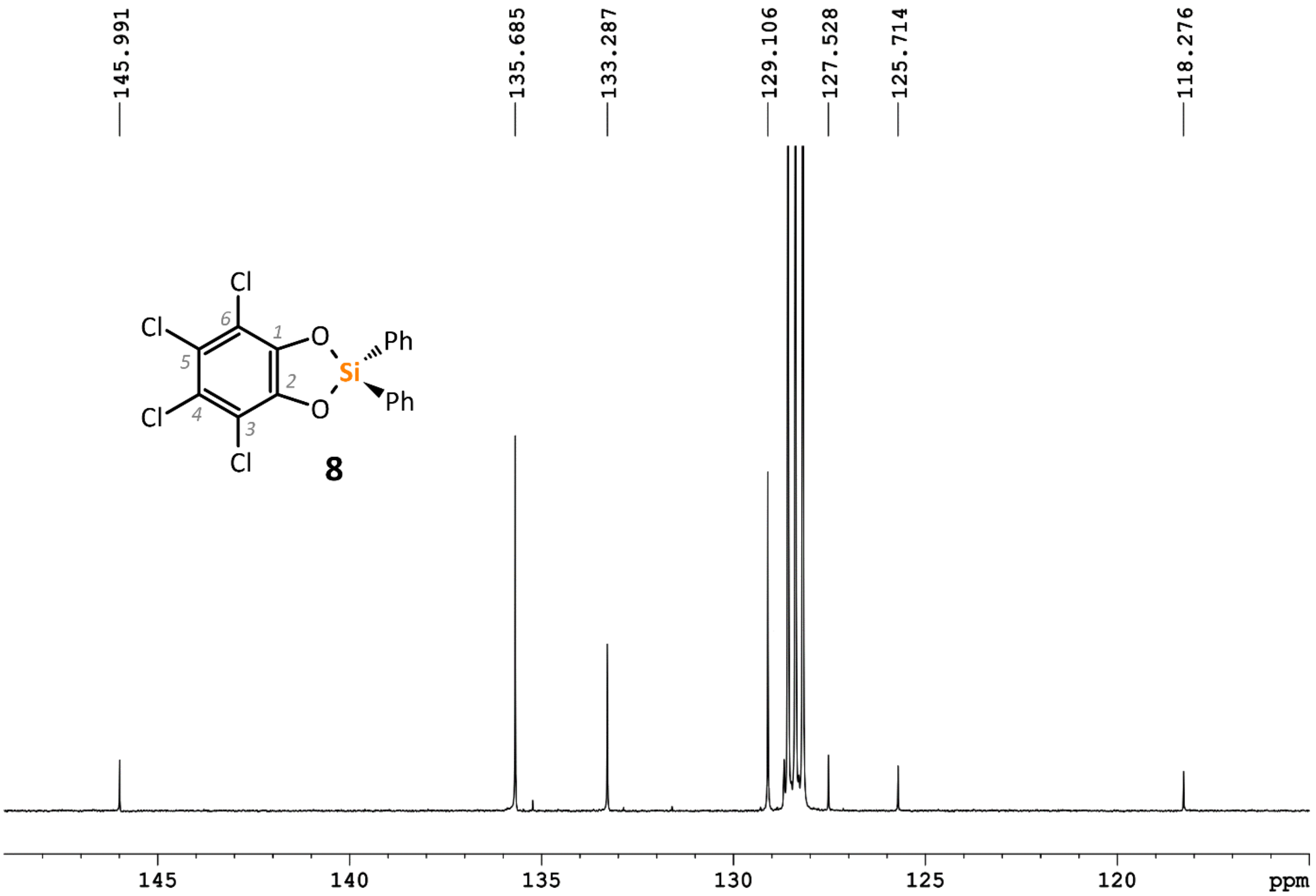

Figure S80: $^{13}C\{^1H\}$ NMR spectrum of isolated compound **8** in C_6D_6 (125.78 MHz, 295 K).

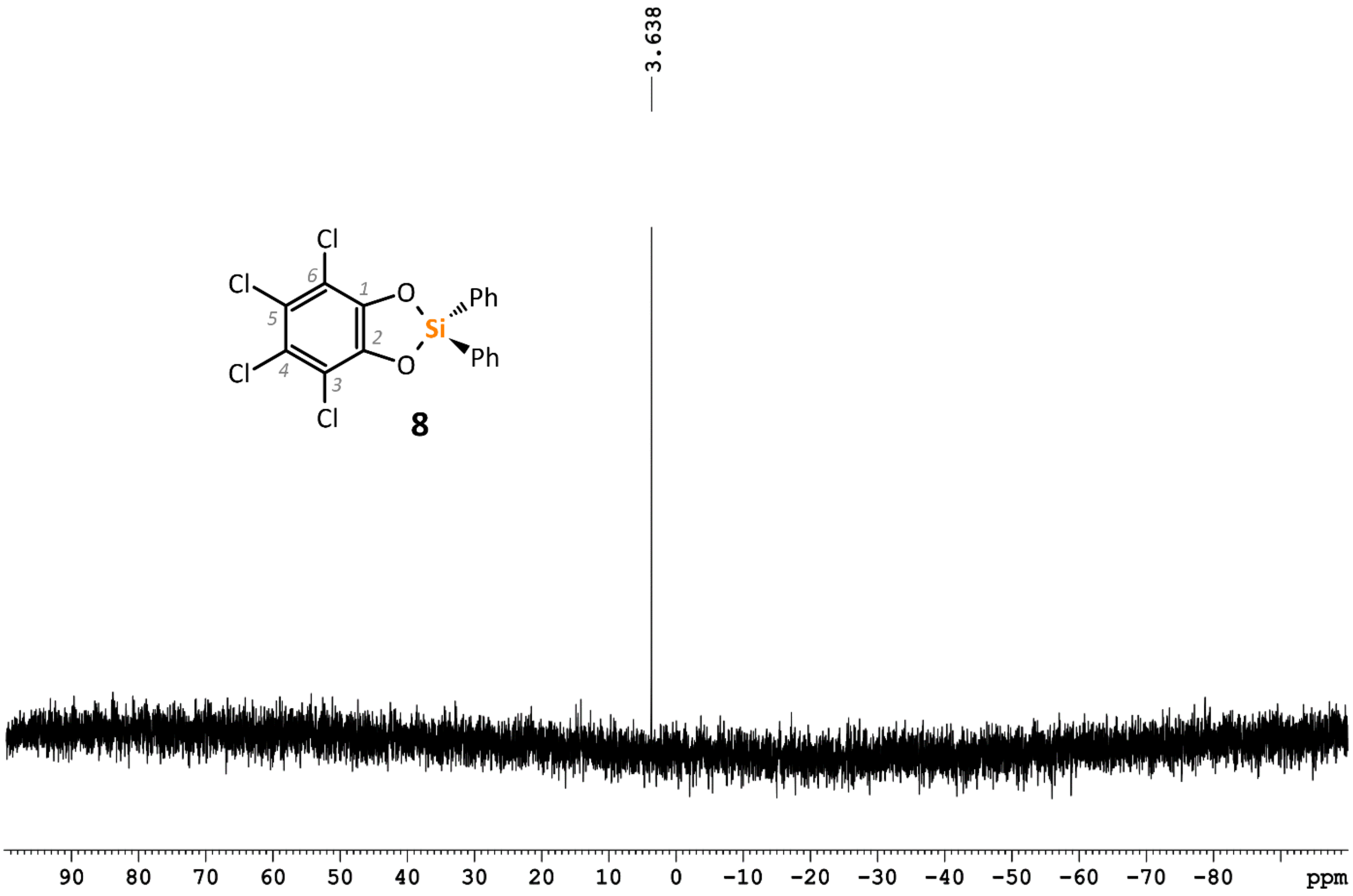

Figure S81: $^{29}Si\{^1H\}$ NMR spectrum of isolated compound **8** in C_6D_6 (99.37 MHz, 295 K).

NMR spectra of compound 9

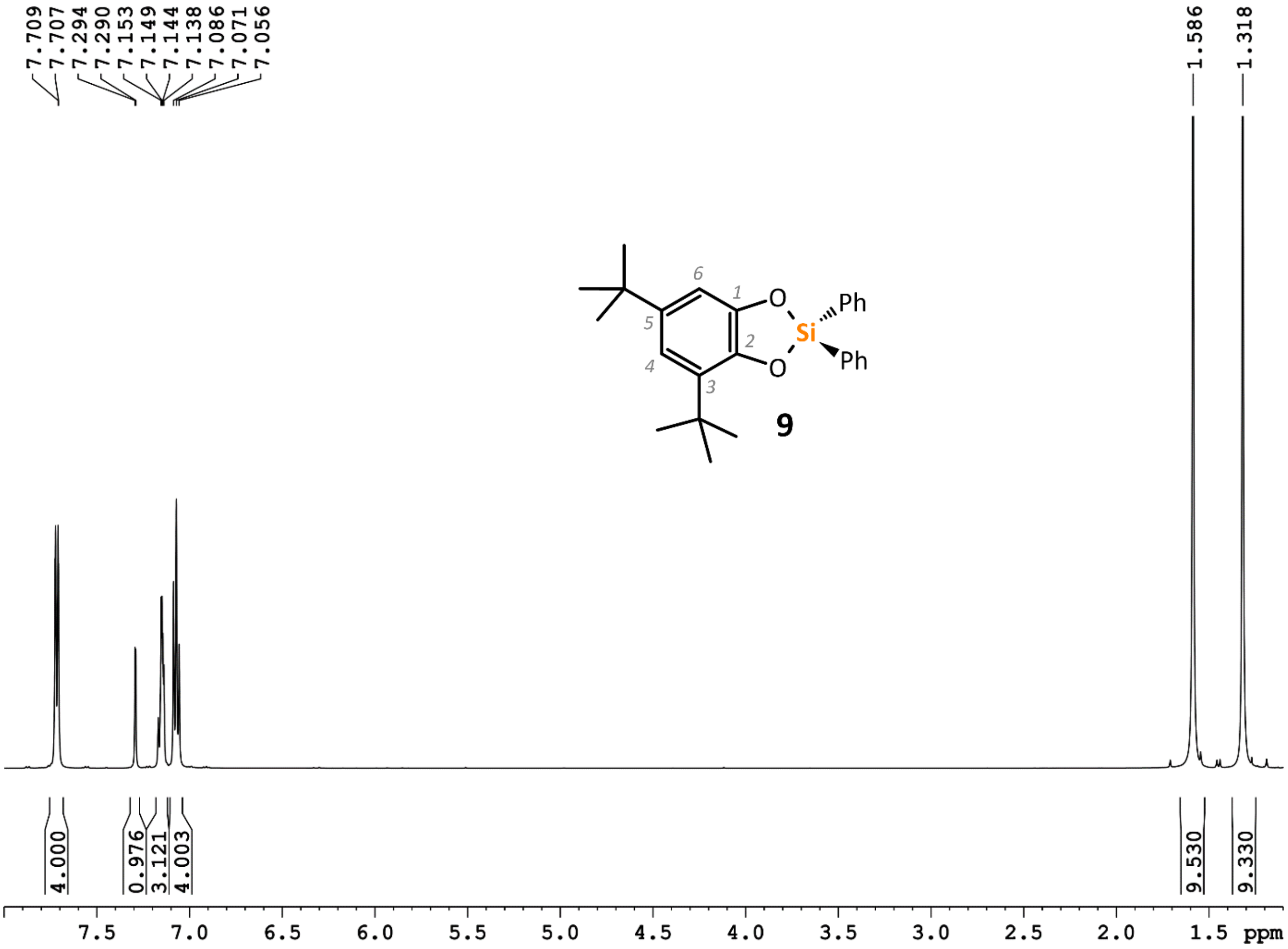

Figure S82: ^{1}**H NMR** spectrum of isolated compound **9** in C_6D_6 (500.20 MHz, 295 K).

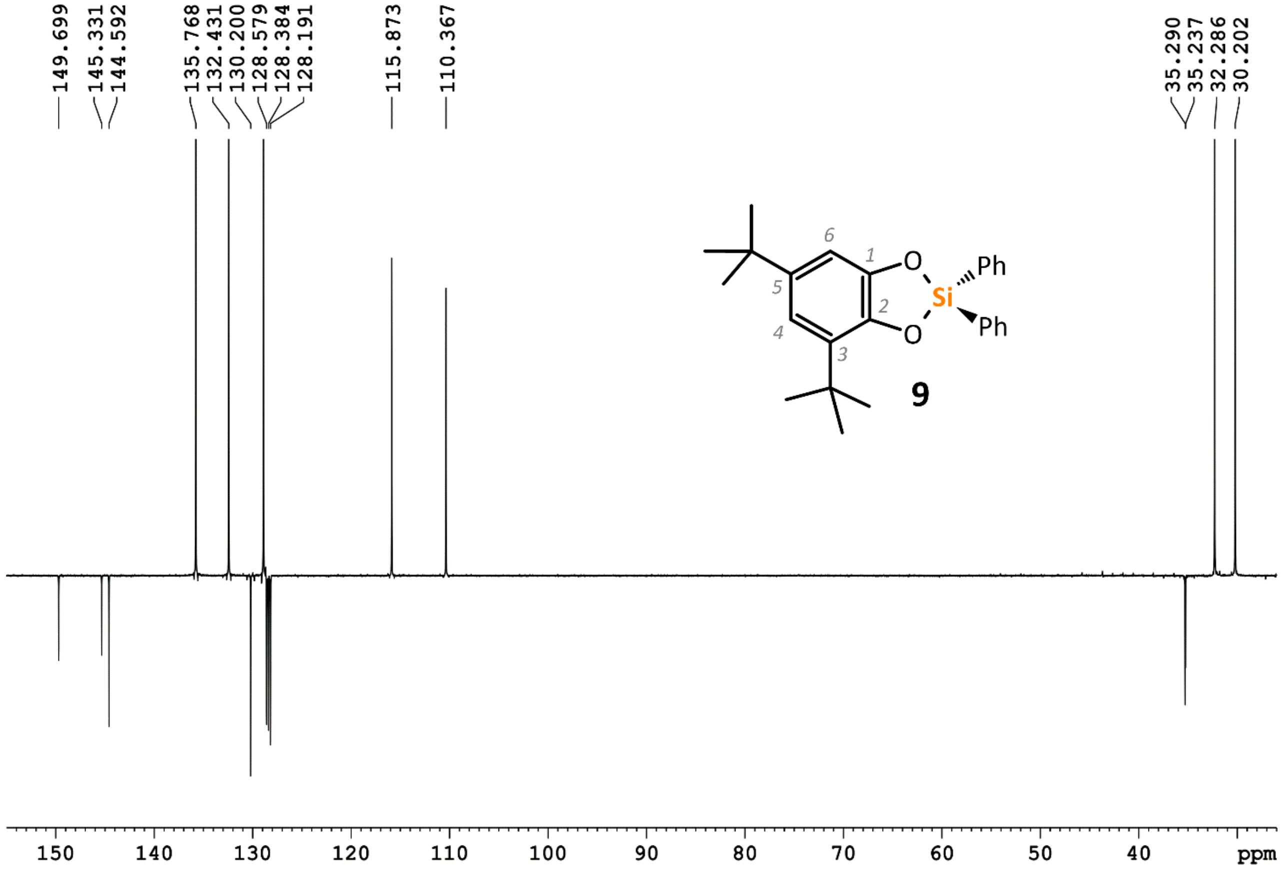

Figure S83: $^{13}C\{^{1}H\}$ **APT NMR** spectrum of isolated compound **9** in C_6D_6 (125.78 MHz, 295 K).

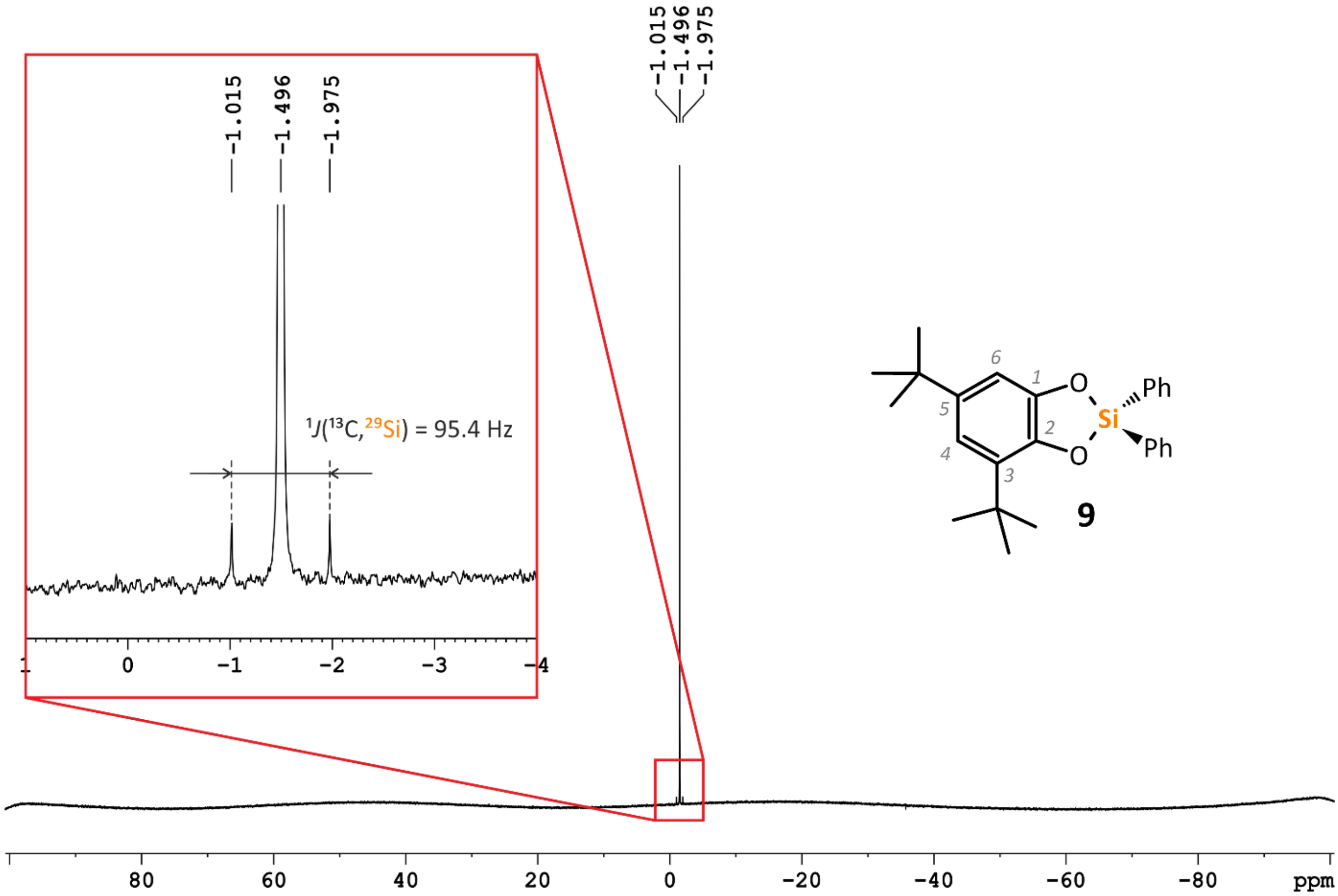

Figure S84: $^{29}Si\{^{1}H\}$ **NMR** spectrum of isolated compound **9** in C_6D_6 (99.37 MHz, 295 K, NS = 512).

Experiments proving stepwise Si-H bond activation during formation of compound 9 via formation of compound 9a and corresponding NMR data

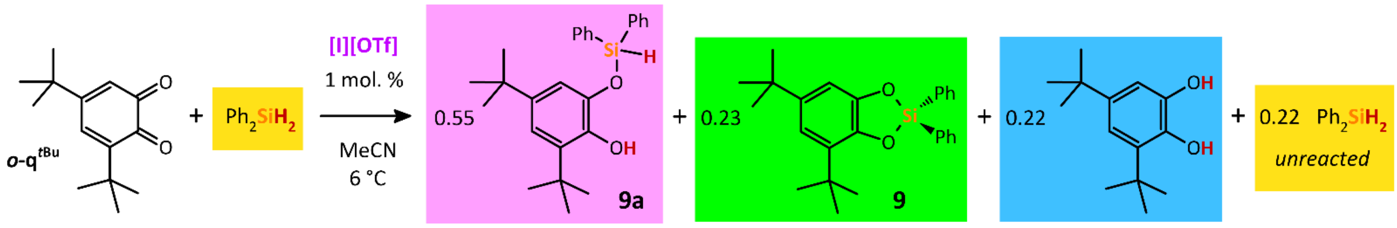

18.0 mg (41.2 µmol, 1 mol. % *vs* Si-H) of **[I][OTf]** and 908 mg (4.12 mmol) of ***o*-q**^{tBu} were loaded into a Schlenk tube and dissolved in 25 mL of dry and degassed acetonitrile under an argon atmosphere. This solution was cooled down to 6 °C. Subsequently, 0.76 mL (4.12 mmol) of neat Ph_2SiH_2 was added to this dark red solution under vigorous stirring. After 20 minutes, a white precipitate of **9** started to form within the dark red solution. This reaction mixture was stirred for 1 hour at 6 °C in total, during which the dark red solution completely transformed to a light-orange suspension. The suspension was evaporated at low pressure and dried in vacuo under formation of honey-like orange material which was characterized by NMR spectroscopy in C_6D_6 (see spectra below).

Despite all efforts, **9a** could not be separated from the reaction mixture and due to overlapping signals in the NMR spectra, only characteristic chemical shifts of **9a** are listed:

^{1}H NMR (500.20 MHz, C_6D_6) δ (ppm): 5.85 [1H, s, Si-*H*, $^{1}J(^{29}Si,^{1}H)$ = 222.0 Hz]; 5.95 [1H, s, O*H*].

^{29}Si NMR (99.38 MHz, C_6D_6) δ: −11.7 (dq) ppm ($^{1}J(^{29}Si,^{1}H)$ = 222.0 Hz).

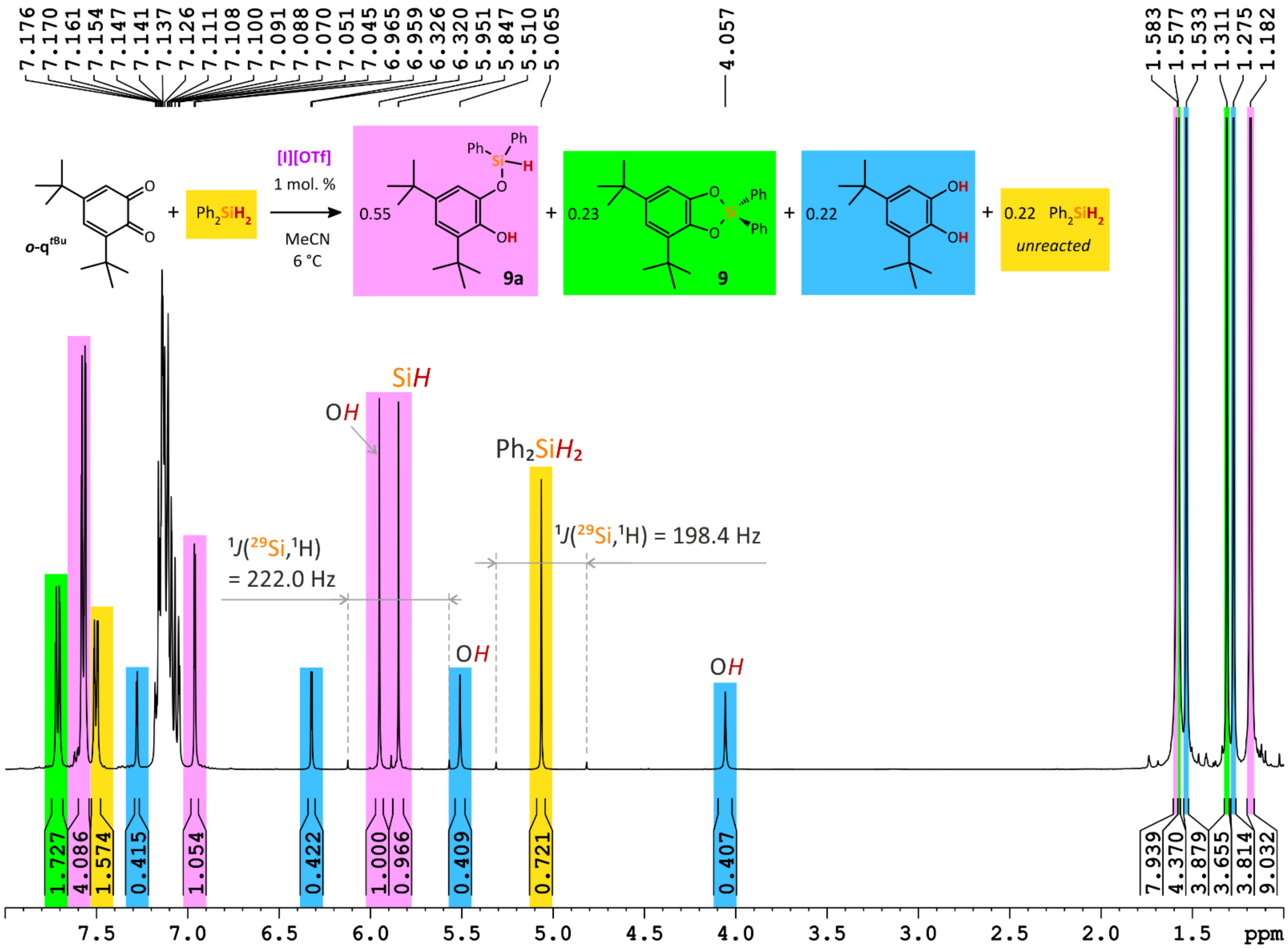

Figure S85: **^{1}H NMR** spectrum of evaporated reaction mixture obtained from reaction of ***o*-q**^{tBu} and Ph_2SiH_2 in 1:1 molar ratio catalyzed by 1 mol. % of **[I][OTf]** in MeCN at 6 °C (500.20 MHz, 295 K, C_6D_6).

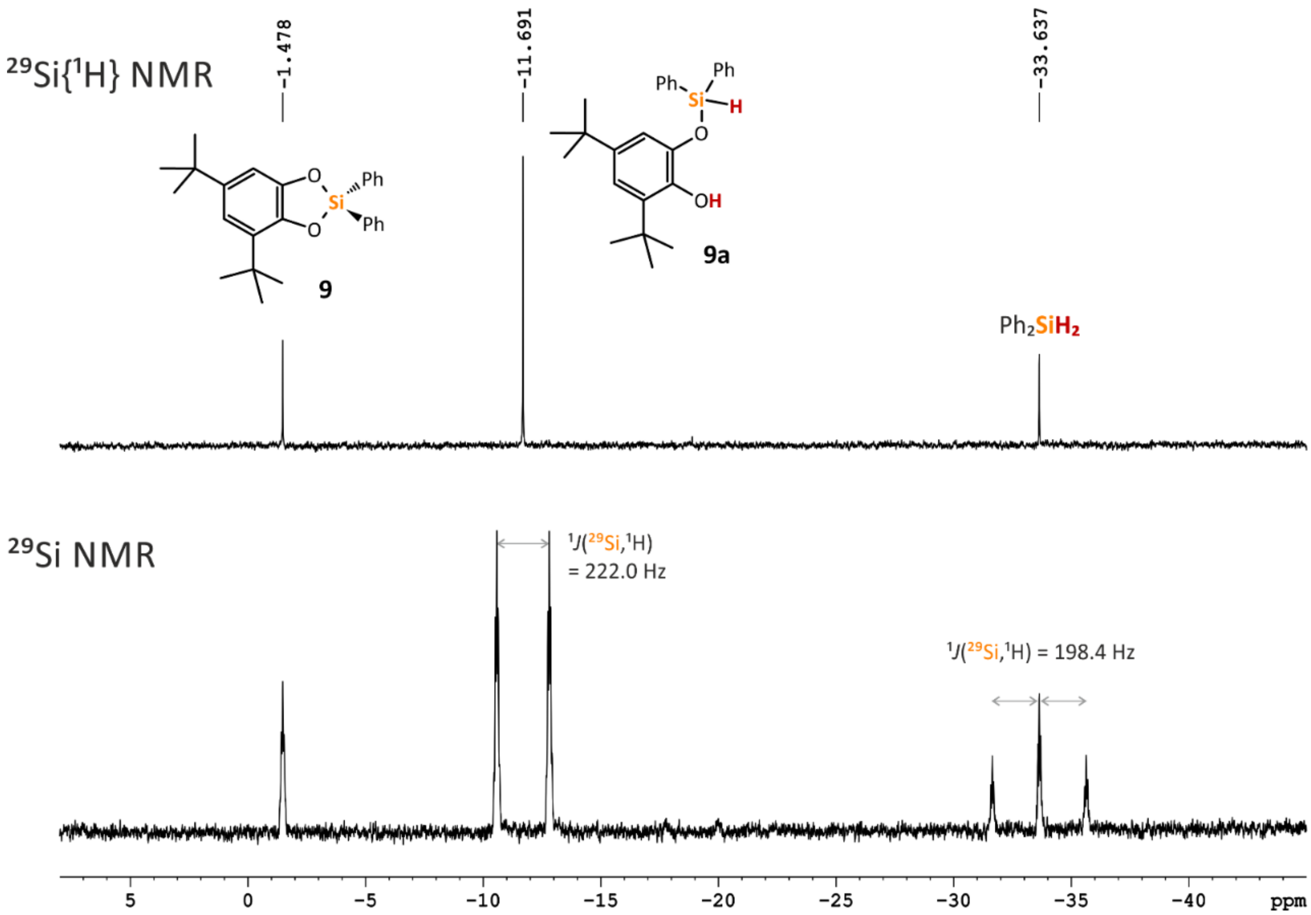

Figure S86: ^{29}Si{^{1}H} NMR spectrum of evaporated reaction mixture obtained from reaction of ***o*-q**^{tBu} and Ph_2SiH_2 in 1:1 molar ratio catalyzed by 1 mol. % of **[I][OTf]** in MeCN at 6 °C (99.37 MHz, 295 K, C_6D_6).

NMR spectra of compound 10

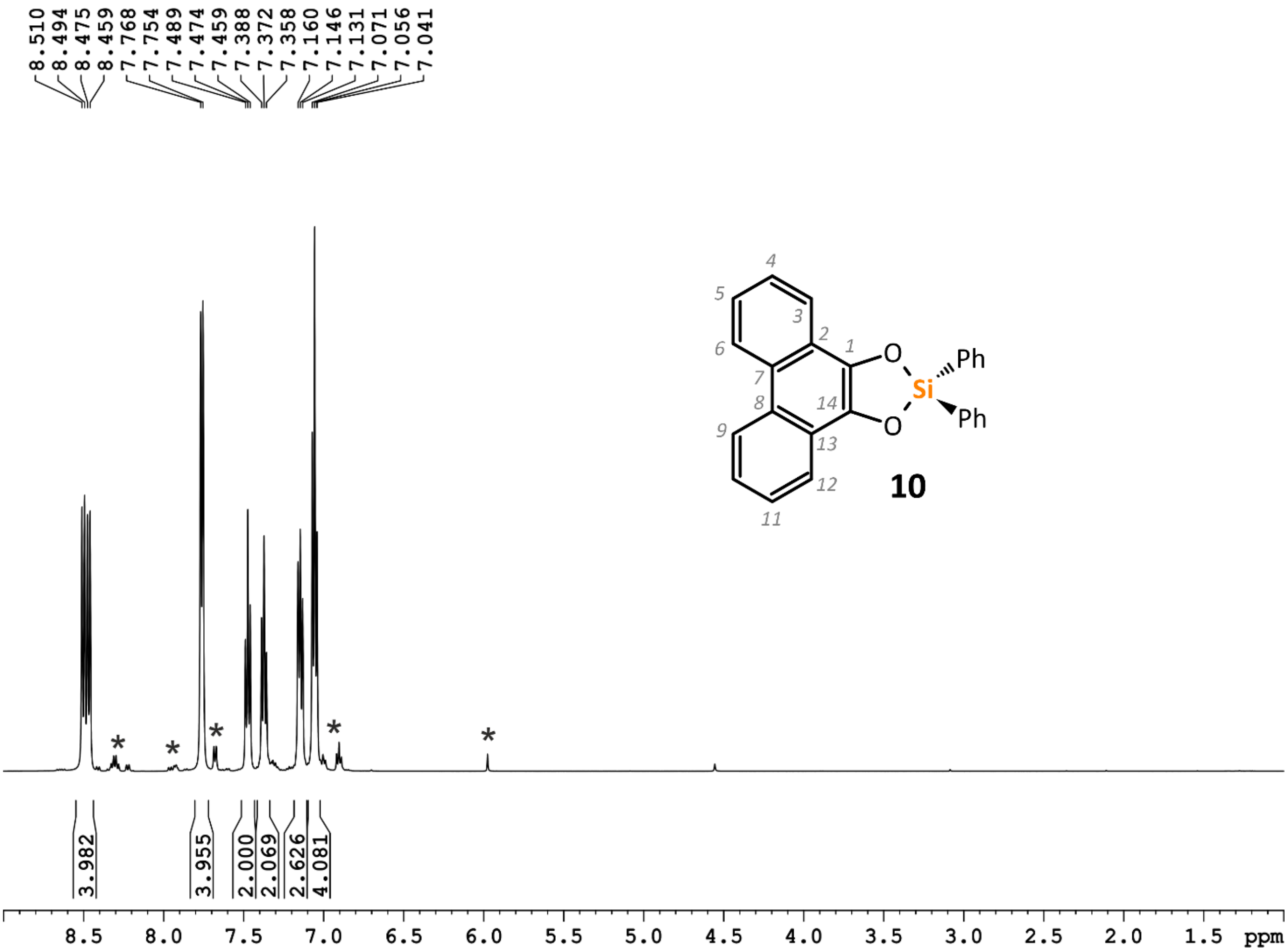

Figure S87: **^{1}H NMR** spectrum of isolated compound **10** in C_6D_6 (500.20 MHz, 295 K). * denotes signals of hydrolysed product **13**.

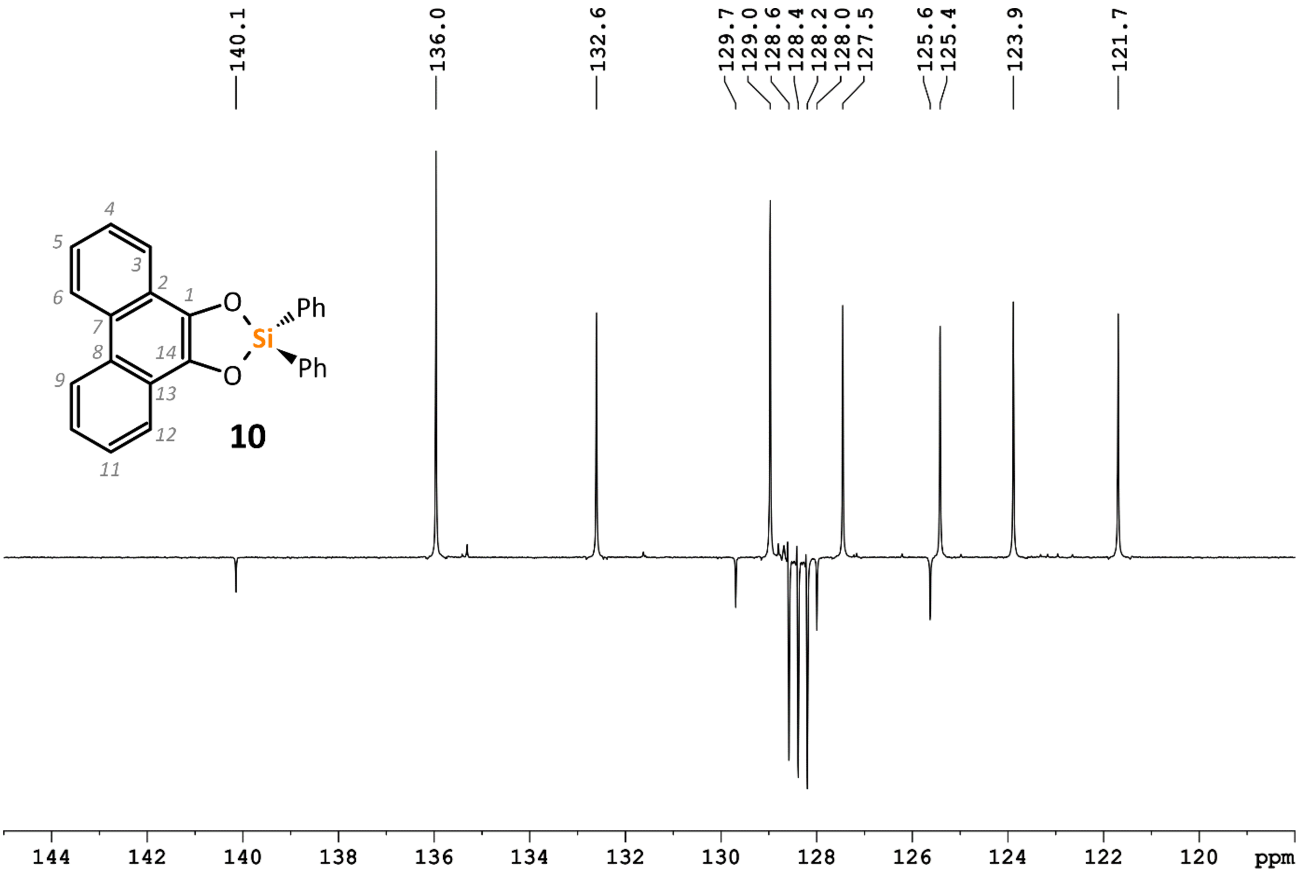

Figure S88: ^{13}**C{**^{1}**H} APT NMR** spectrum of isolated compound **10** in C_6D_6 (125.78 MHz, 295 K).

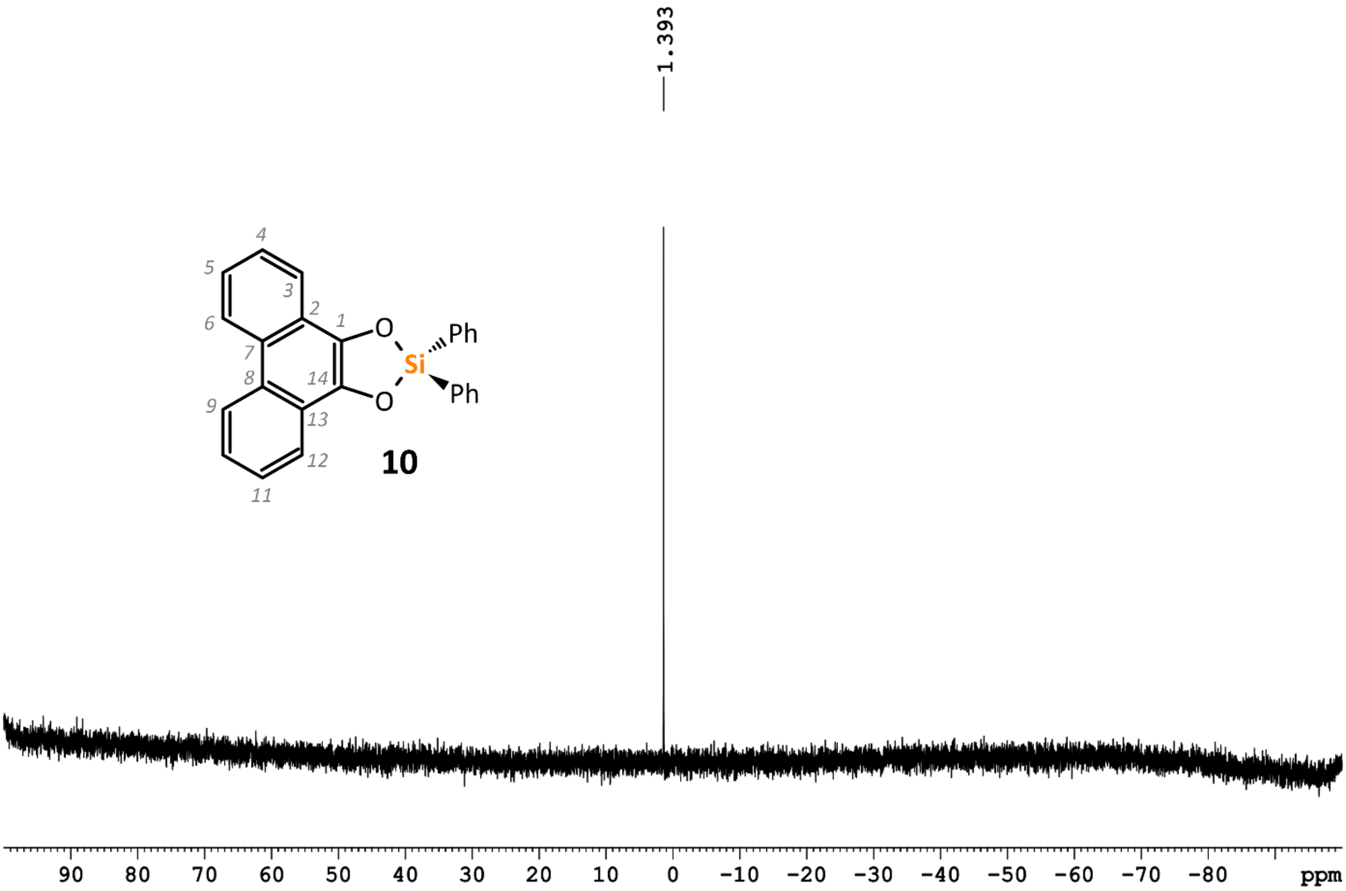

Figure S89: ^{29}**Si{**^{1}**H} NMR** spectrum of isolated compound **10** in C_6D_6 (99.37 MHz, 295 K).

Intentional hydrolysis of compounds 8-10 leading to compounds 11-13

NMR spectra of compound 11

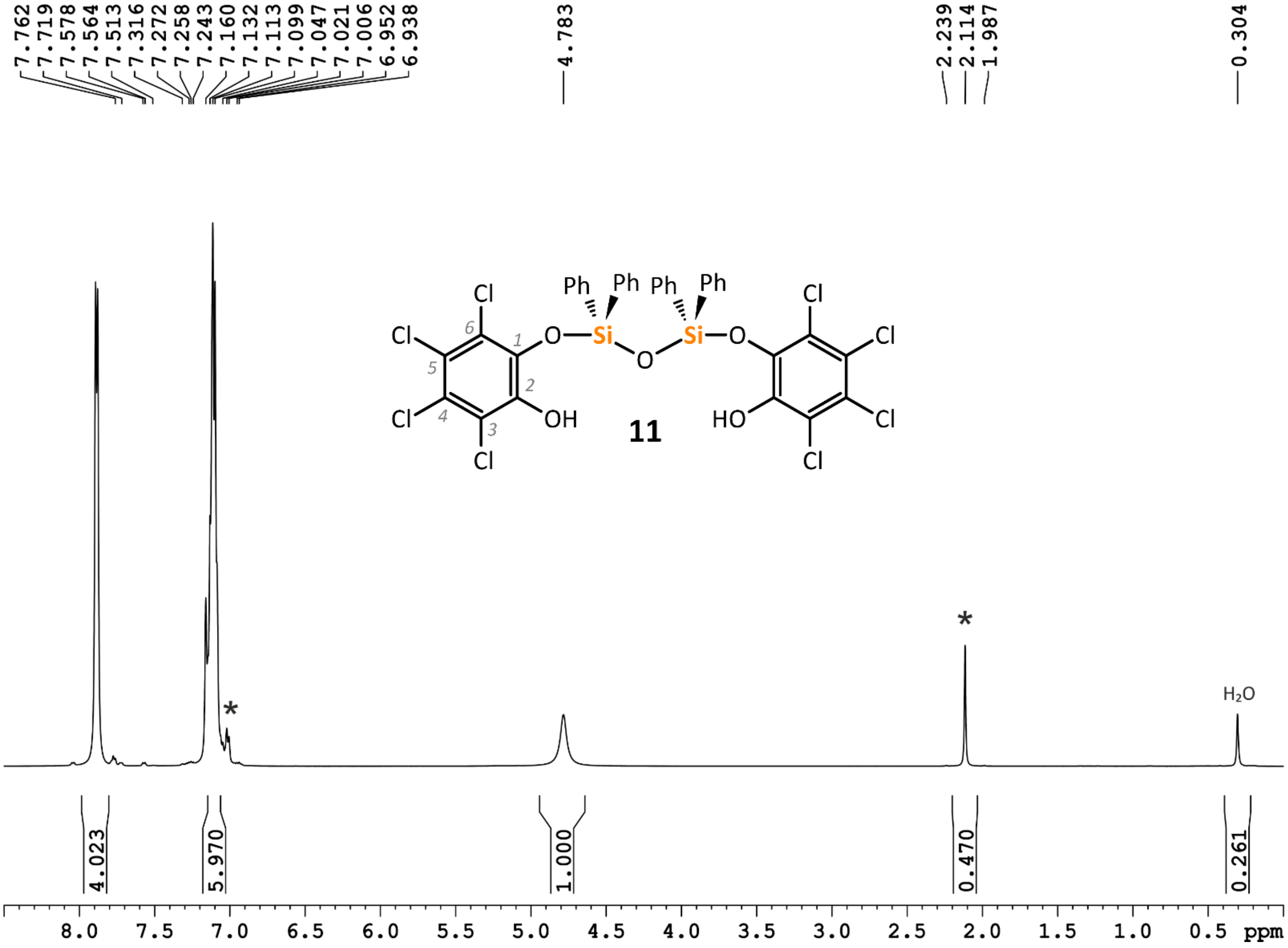

Figure S90: **^{1}H NMR** spectrum of isolated compound **11** in C_6D_6 (500.20 MHz, 295 K). * denotes signals of toluene.

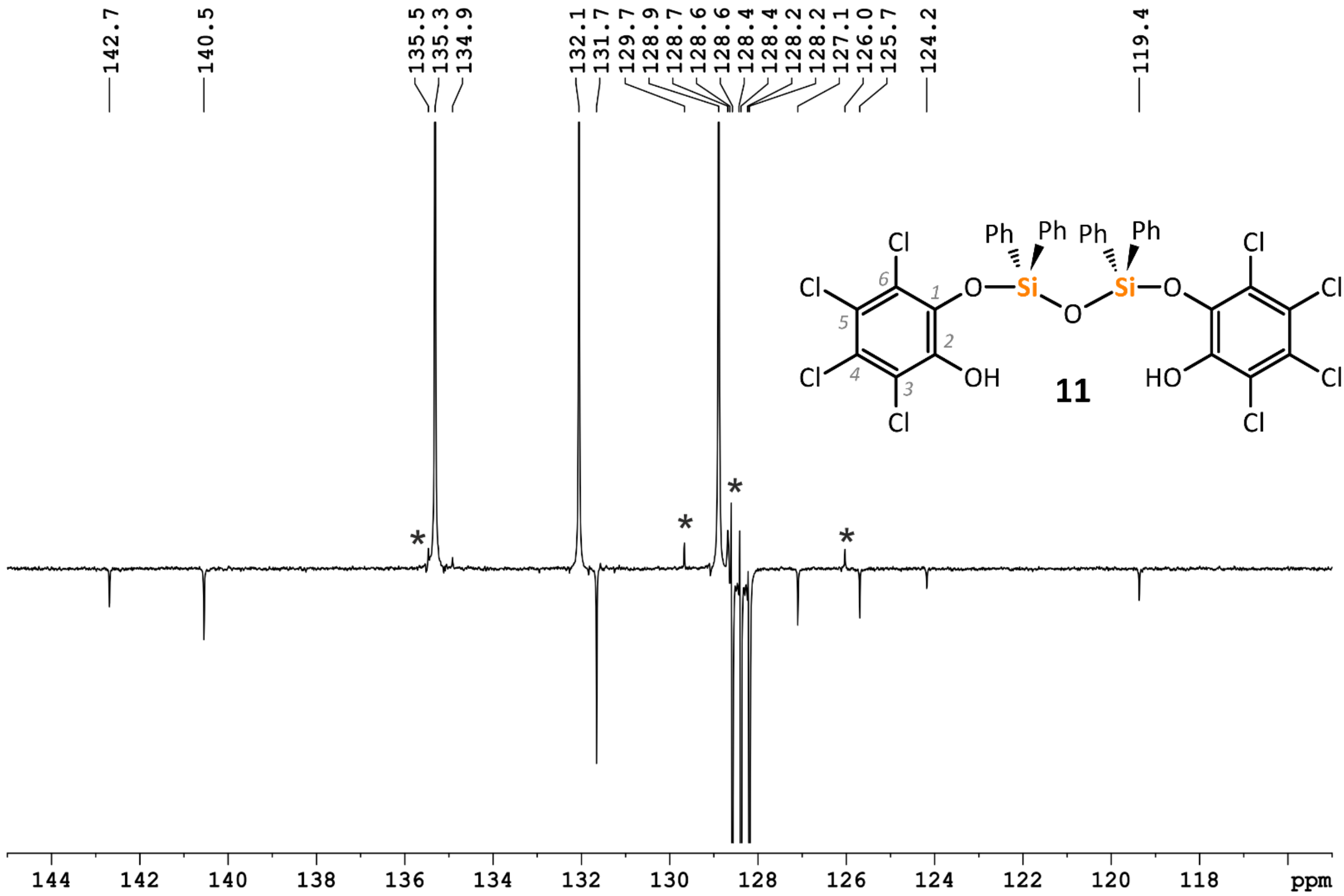

Figure S91: $^{13}C\{^{1}H\}$ **APT NMR** spectrum of isolated compound **11** in C_6D_6 (125.78 MHz, 295 K). * denotes signals of toluene.

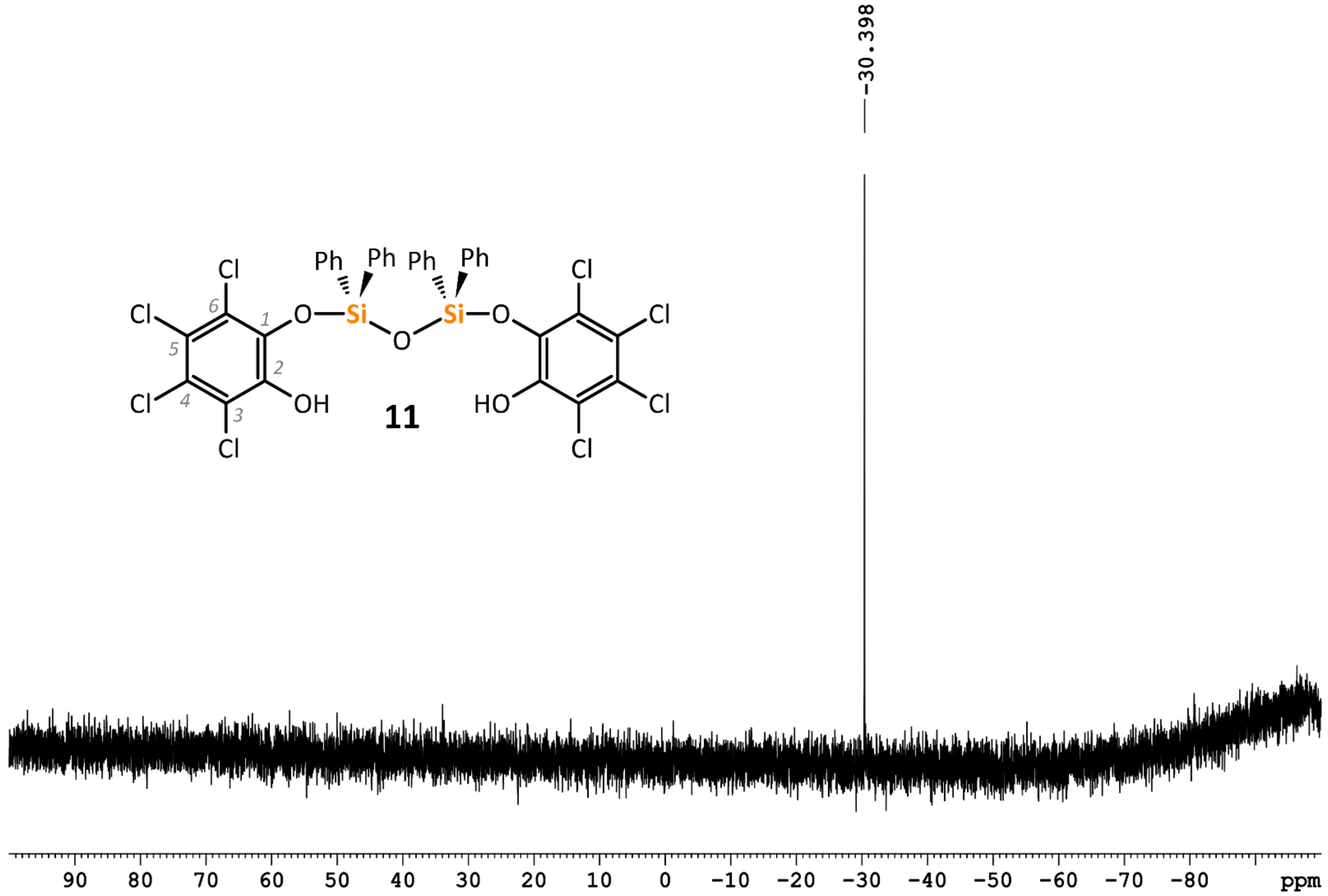

Figure S92: $^{29}Si\{^{1}H\}$ **NMR** spectrum of compound **11** in C_6D_6 (99.37 MHz, 295 K).

NMR spectra of compound 12

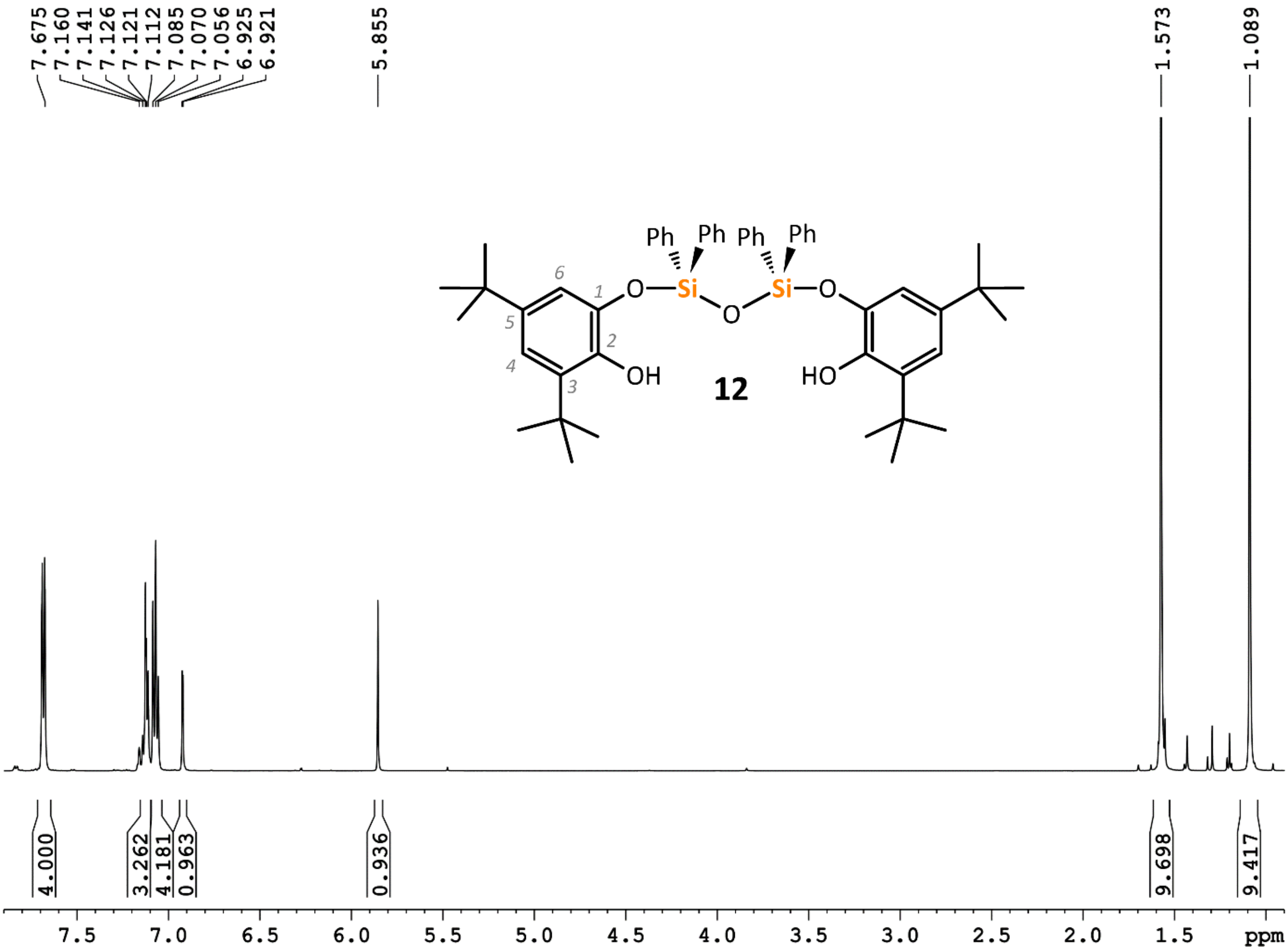

Figure S93: **^{1}H NMR** spectrum of isolated compound **12** in C_6D_6 (500.20 MHz, 295 K).

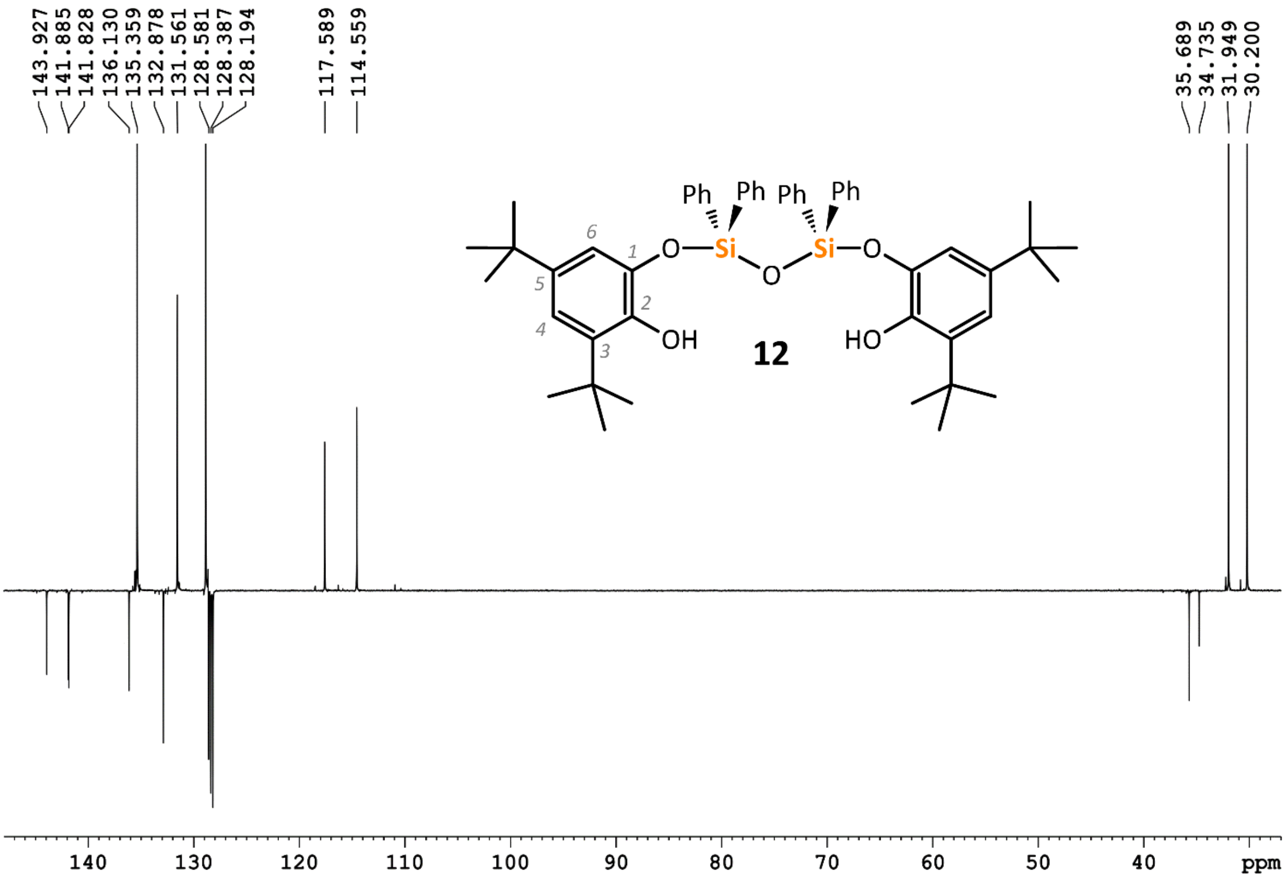

Figure S94: **$^{13}C\{^{1}H\}$ APT NMR** spectrum of isolated compound **12** in C_6D_6 (125.78 MHz, 295 K).

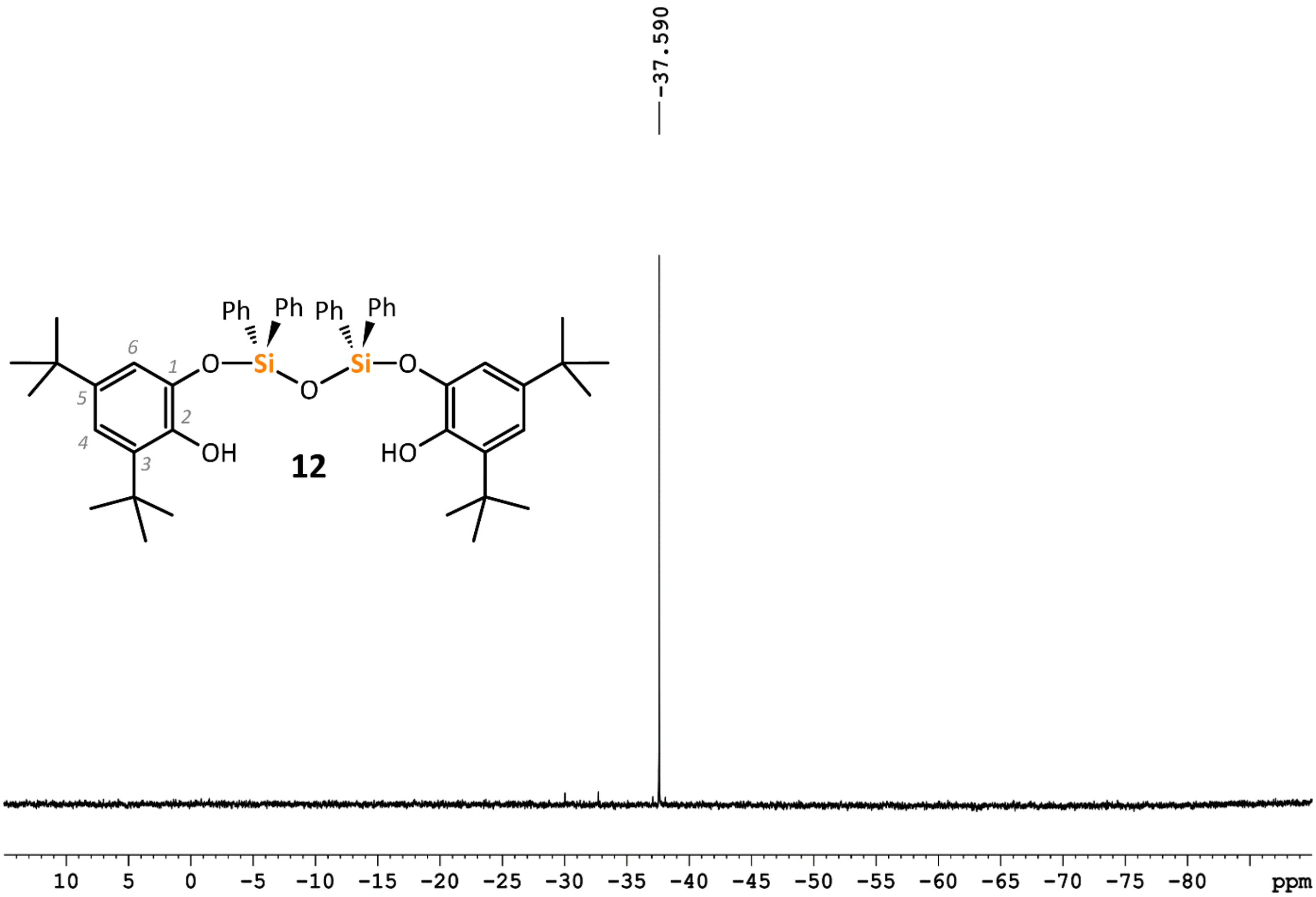

Figure S95: **$^{29}Si\{^{1}H\}$ NMR** spectrum of compound **12** in C_6D_6 (99.37 MHz, 295 K).

NMR spectra of compound 13

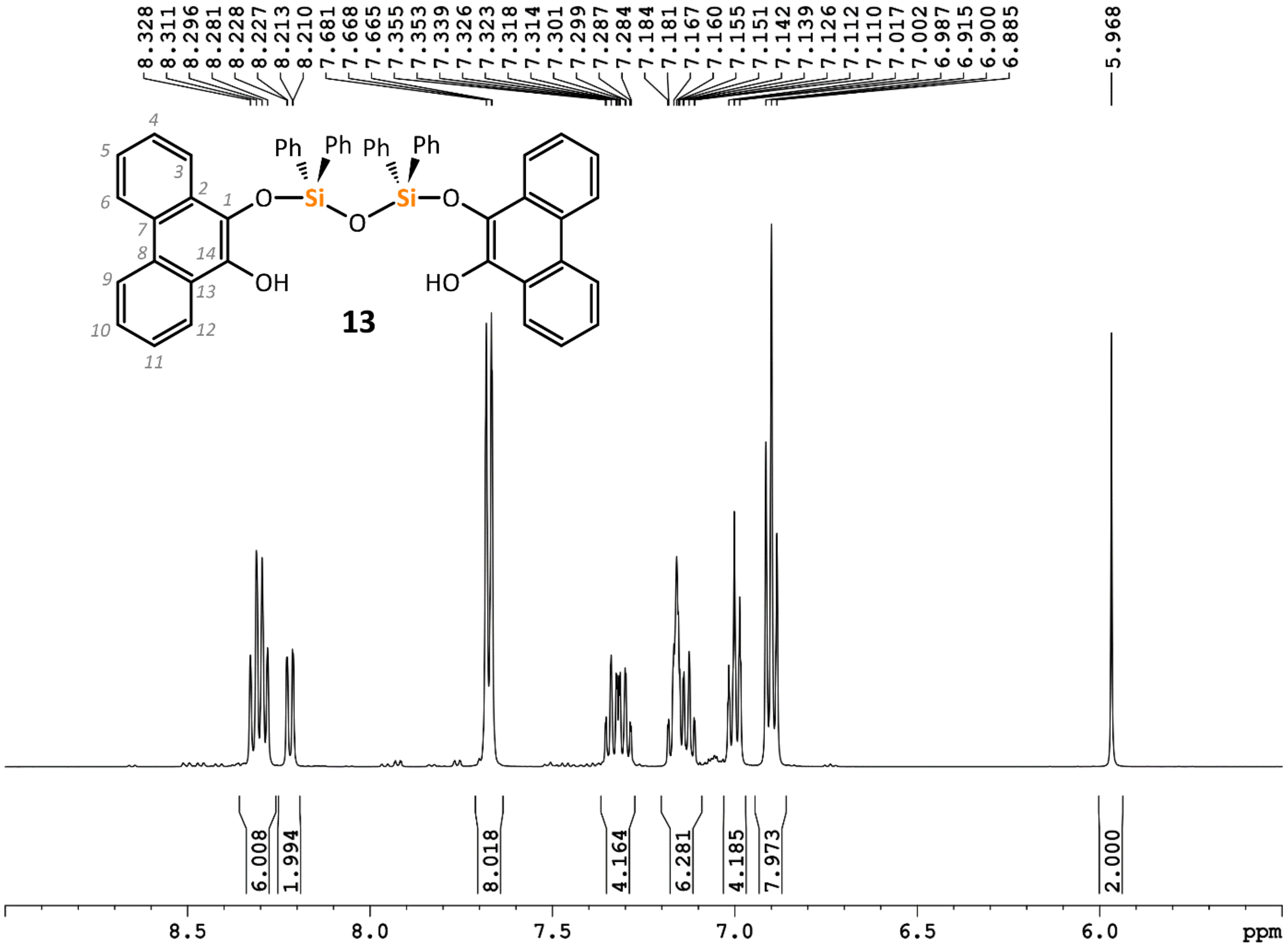

Figure S96: ^{1}H NMR spectrum of isolated compound **13** in C_6D_6 (500.20 MHz, 295 K).

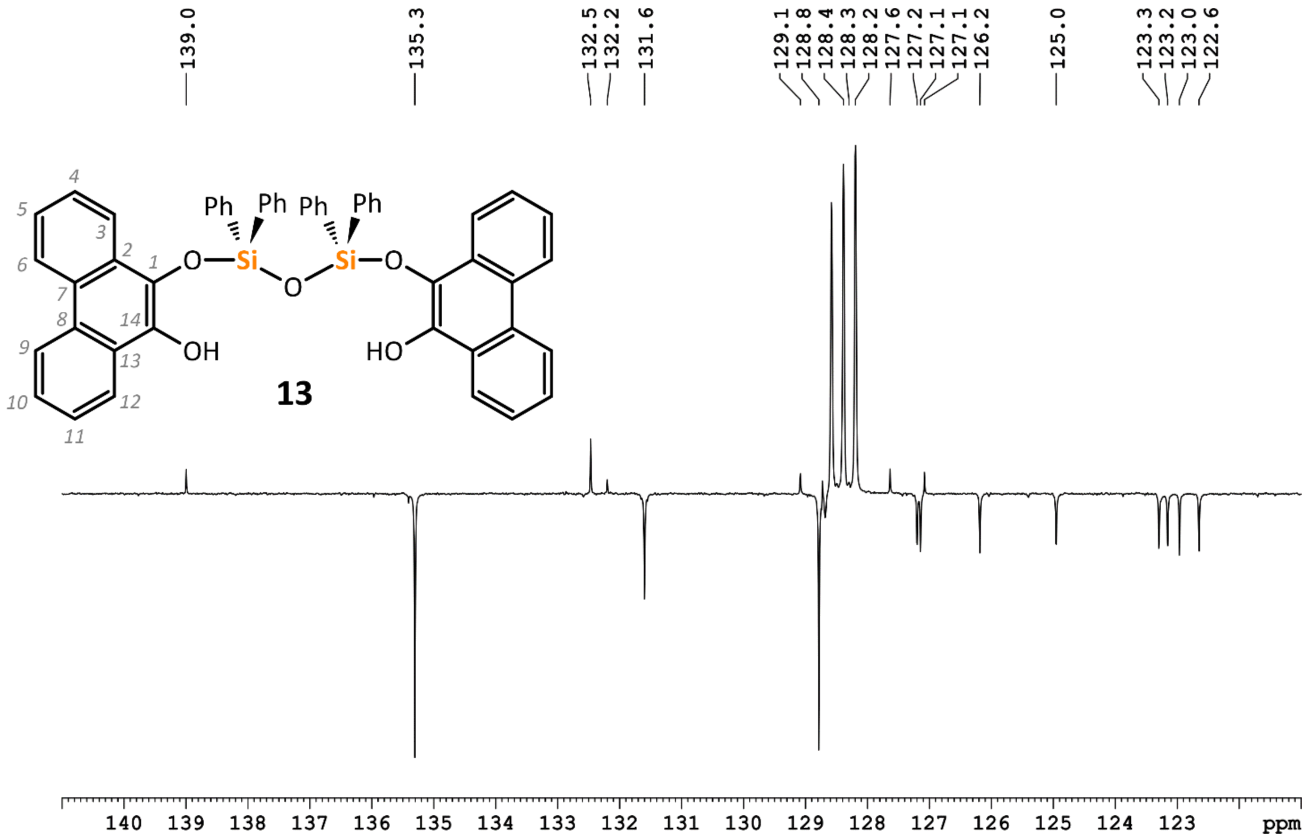

Figure S97: $^{13}C\{^{1}H\}$ APT NMR spectrum of isolated compound **13** in C_6D_6 (125.78 MHz, 295 K).

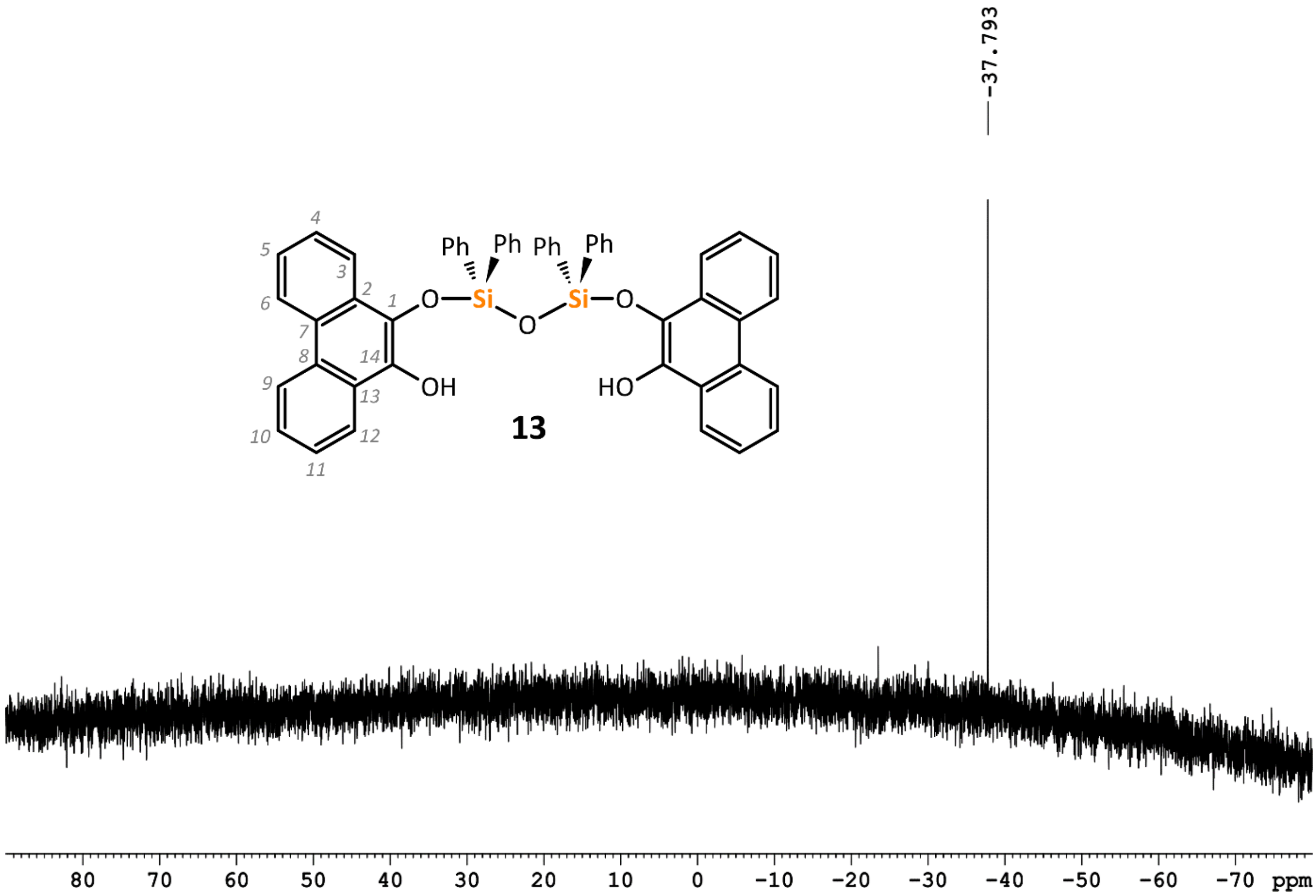

Figure S98: $^{29}Si\{^{1}H\}$ spectrum of isolated compound **13** in C_6D_6 (99.37 MHz, 295 K).

Non-catalyzed reactions of quinones with Ph_2SiH_2

Non-catalyzed reaction of o-q^{Cl} with Ph_2SiH_2

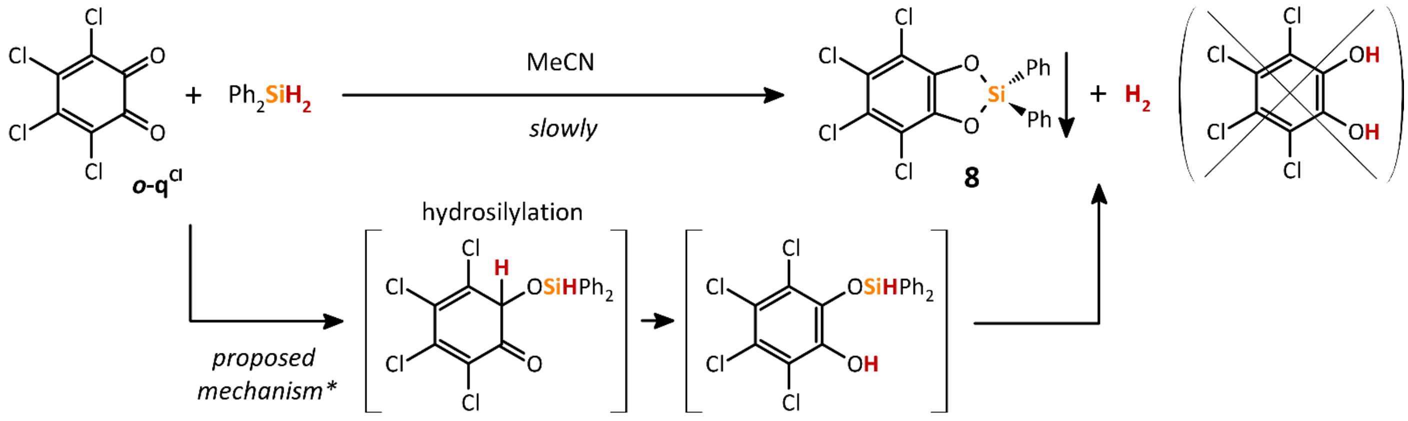

* proposed based on similar reaction described for reaction of *ortho*-quinones with BH_3.[1]

1.20 g (4.88 mmol) of ***o*-q^{Cl}** was loaded into a Schlenk tube and dissolved in 25 mL of dry and degassed acetonitrile under an argon atmosphere. Subsequently, 0.91 mL (4.88 mmol) of neat Ph_2SiH_2 was added to this dark red solution under vigorous stirring. During the next 5 days, a white precipitate was slowly formed. The red mother solution was filtrated, and the white precipitate was dried in vacuo and analyzed by NMR in C_6D_6 (see spectra below). Solvent from the red mother liquor was removed under reduced pressure and resulting red oil was dried in vacuo, which based on NMR spectroscopy did not contain the catechol, but only unreacted starting ***o*-q^{Cl}** and Ph_2SiH_2.

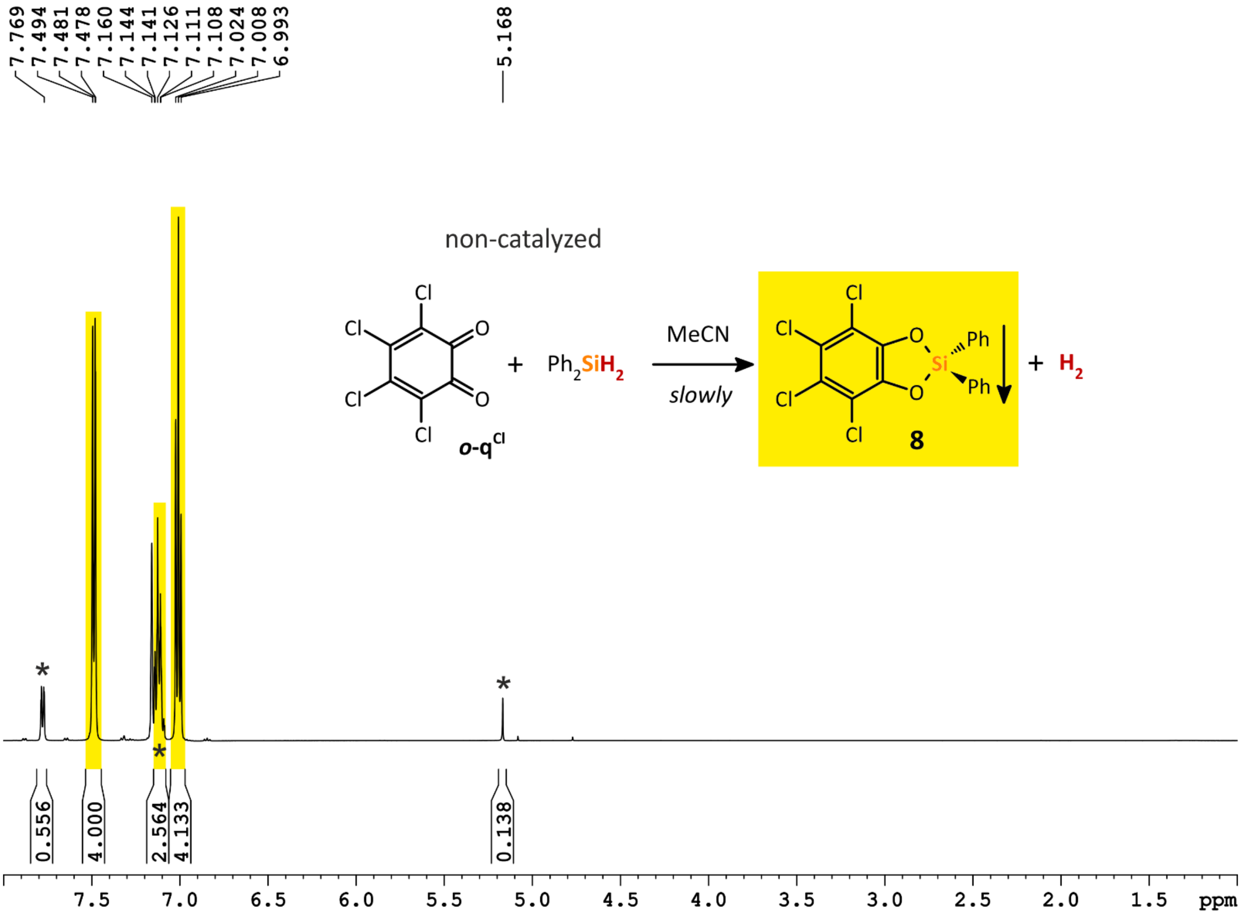

Figure S99: **^{1}H NMR** spectrum of white precipitate of **8** slowly formed during 5 days of mixing of ***o*-q^{Cl}** with Ph_2SiH_2 (1:1 molar ratio) with no addition of catalyst in MeCN at RT (500.20 MHz, 295 K, C_6D_6). * denotes signals of compound **11** formed by partial hydrolysis of **8.**

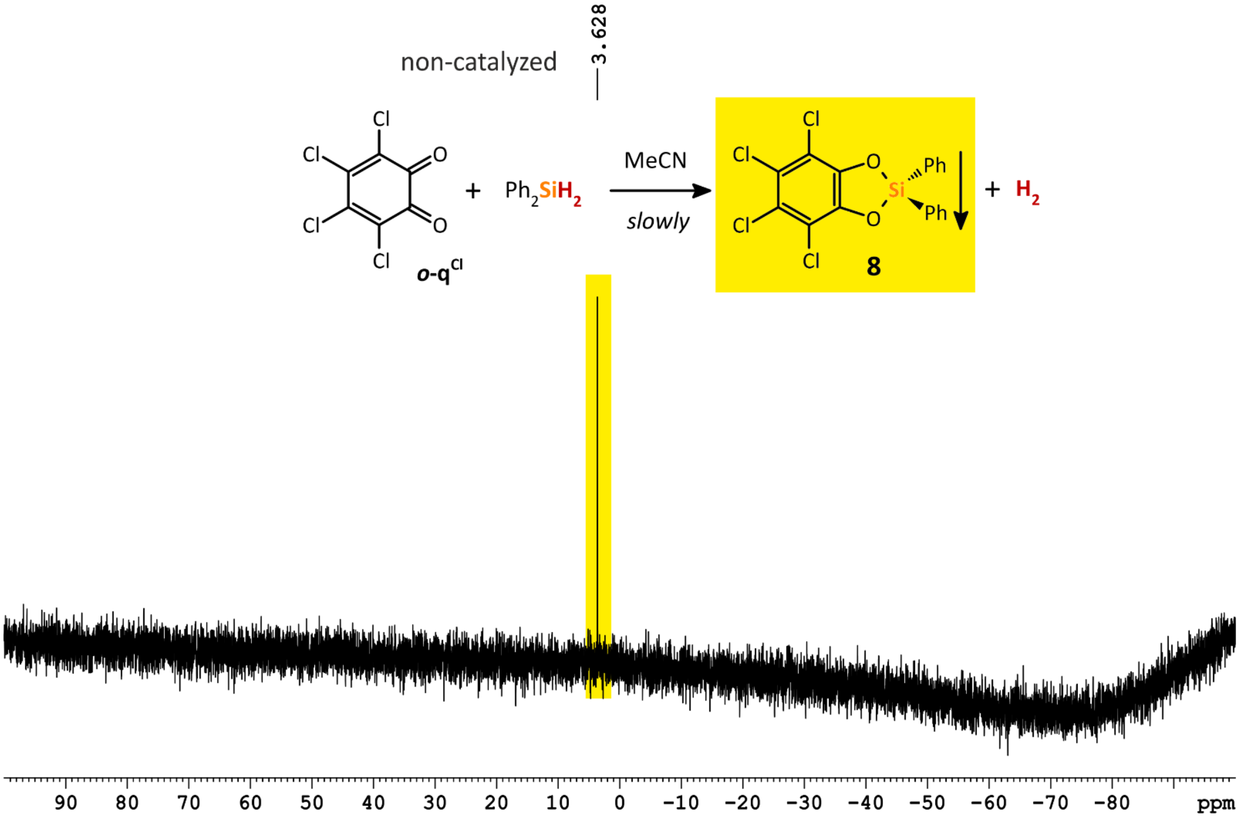

Figure S100: **^{29}Si{^{1}H} NMR** spectrum of white precipitate slowly formed during 5 days of mixing of ***o*-q^{Cl}** with Ph_2SiH_2 (1:1 molar ratio) with no addition of catalyst in MeCN at RT (99.37 MHz, 295 K, C_6D_6). Signal for product of partial hydrolysis (compound **11**) is not observed due to low SNR.

Non-reactivity of o-q^{tBu} with Ph_2SiH_2

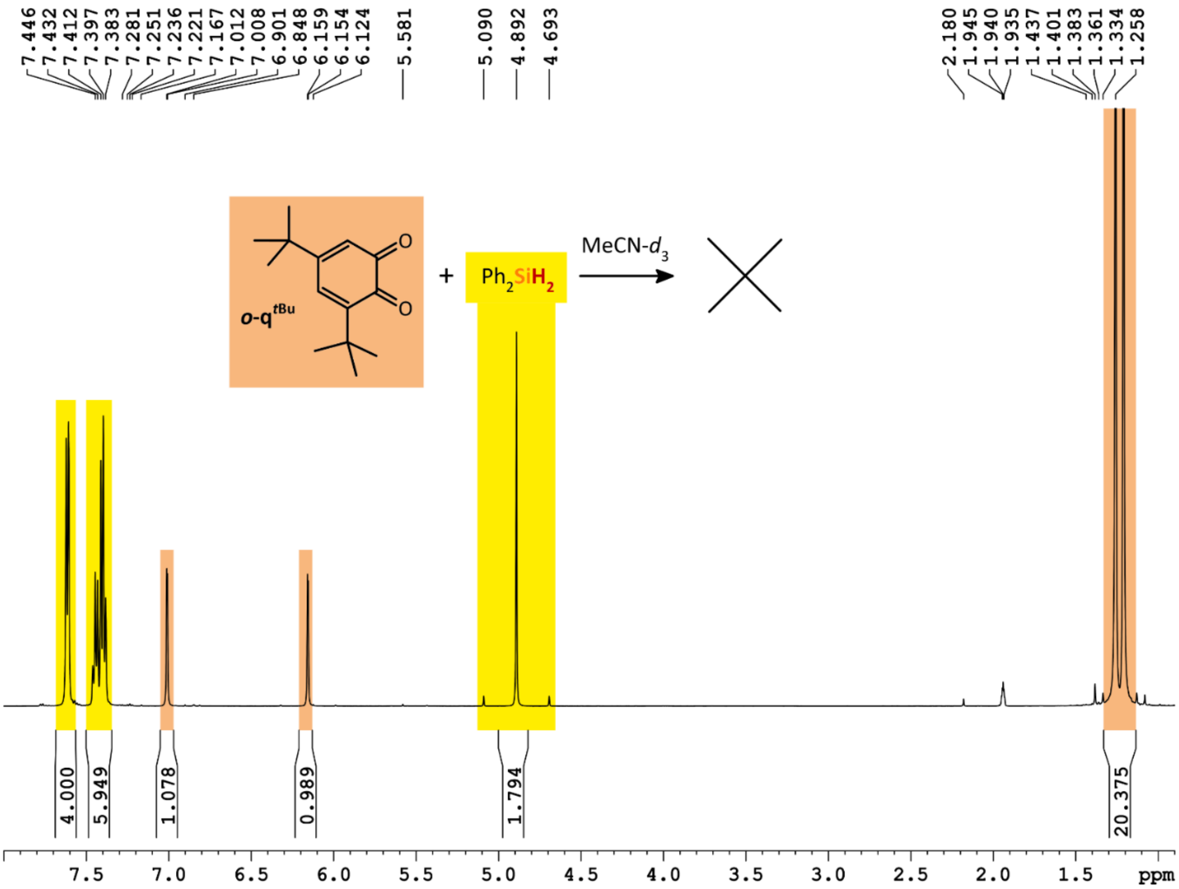

Figure S101: ^{1}**H NMR** spectrum of 1:1 mixture of ***o*-q^{tBu}** and Ph_2SiH_2 in MeCN-d_3 forming clear dark brown solution. This spectrum was acquired after a week of staying in the flame-sealed NMR tube at RT and then heated overnight for 70 °C and shows no signs of reaction (500 MHz, 295 K).

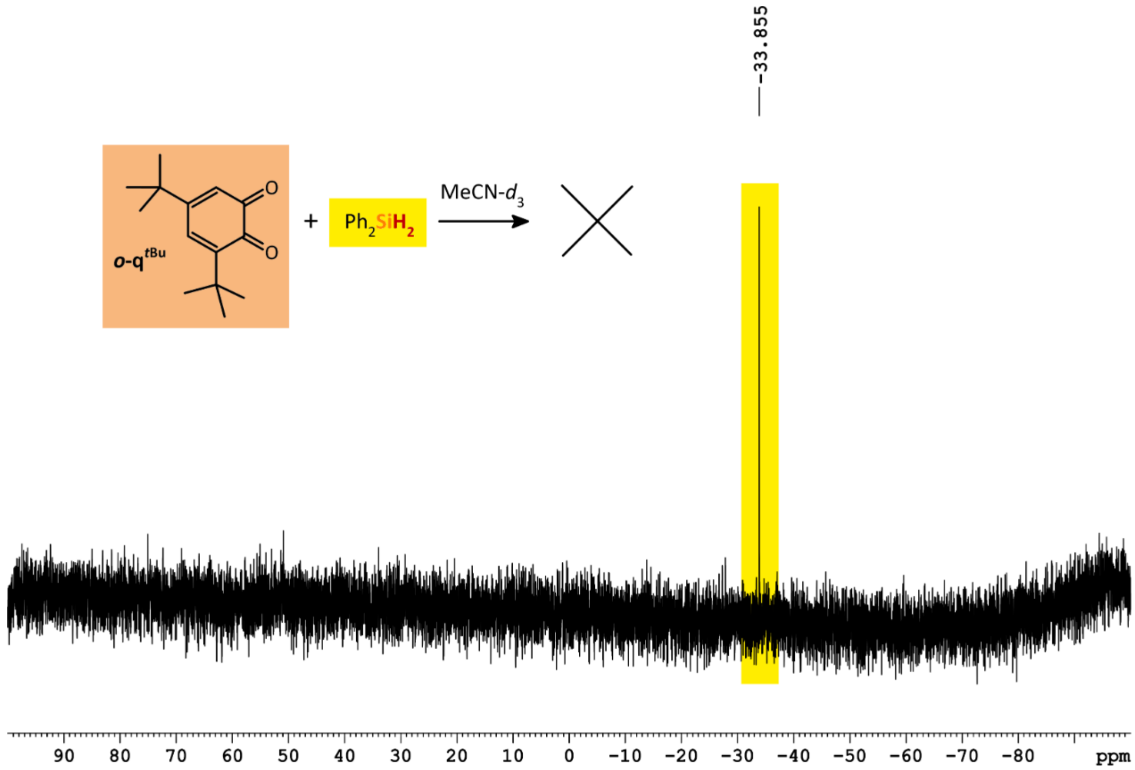

Figure S102: ^{29}**Si{^{1}H} NMR** spectrum of 1:1 mixture of ***o*-q^{tBu}** and Ph_2SiH_2 in MeCN-d_3 forming clear dark brown solution. This spectrum was acquired after a week of staying in the flame-sealed NMR tube at RT and then heated overnight for 70 °C and shows no signs of reaction (99.37 MHz, 295 K).

Non-catalyzed reaction of o-q^phen with Ph_2SiH_2

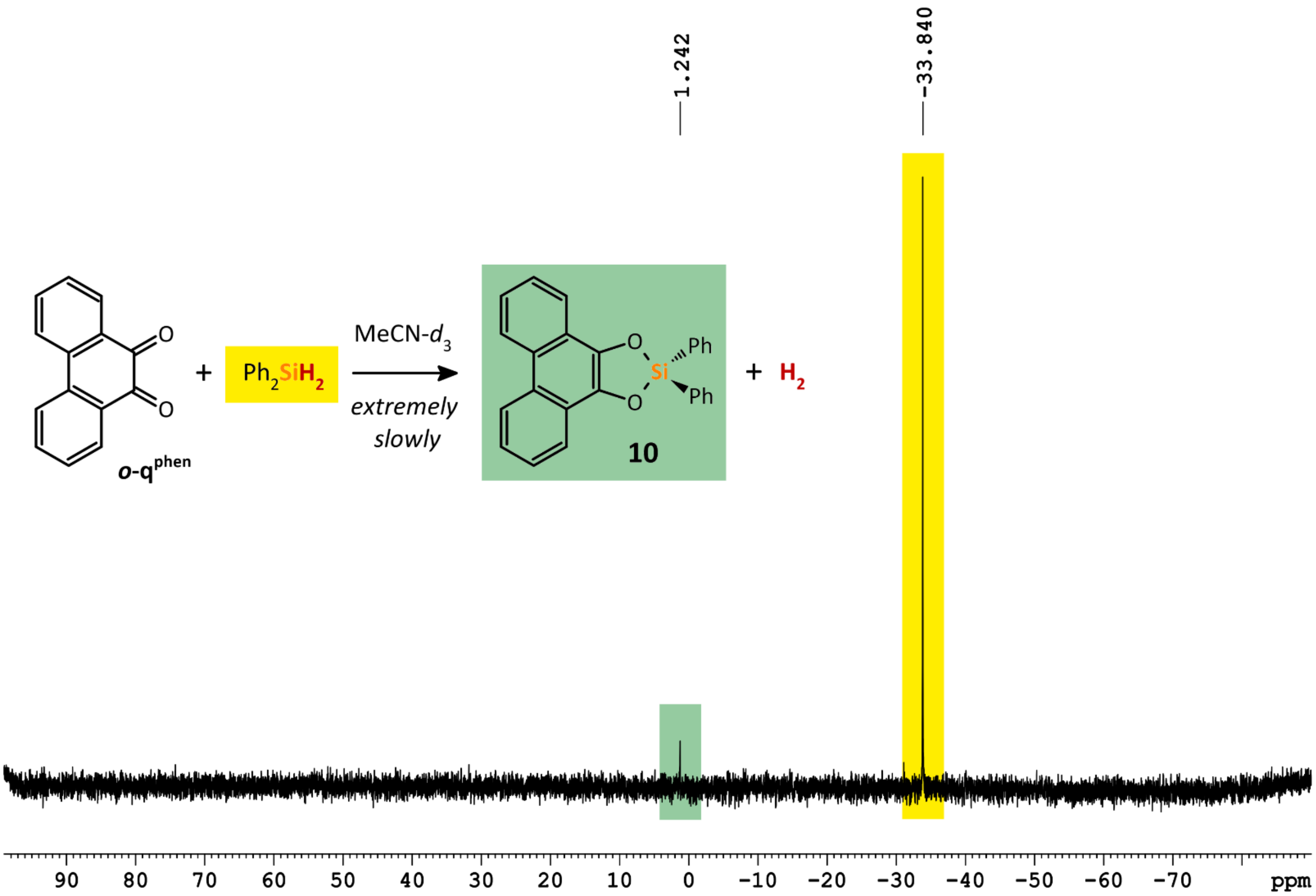

Figure S103: $^{29}Si\{^1H\}$ NMR spectrum of reaction mixture of non-catalyzed reaction of ***o*-q^phen** with Ph_2SiH_2 in MeCN-d_3 after a month at RT showing trace amount of formed compound **10** (99.37 MHz, 295 K).

NMR spectra of compound [14·(EtOH)]

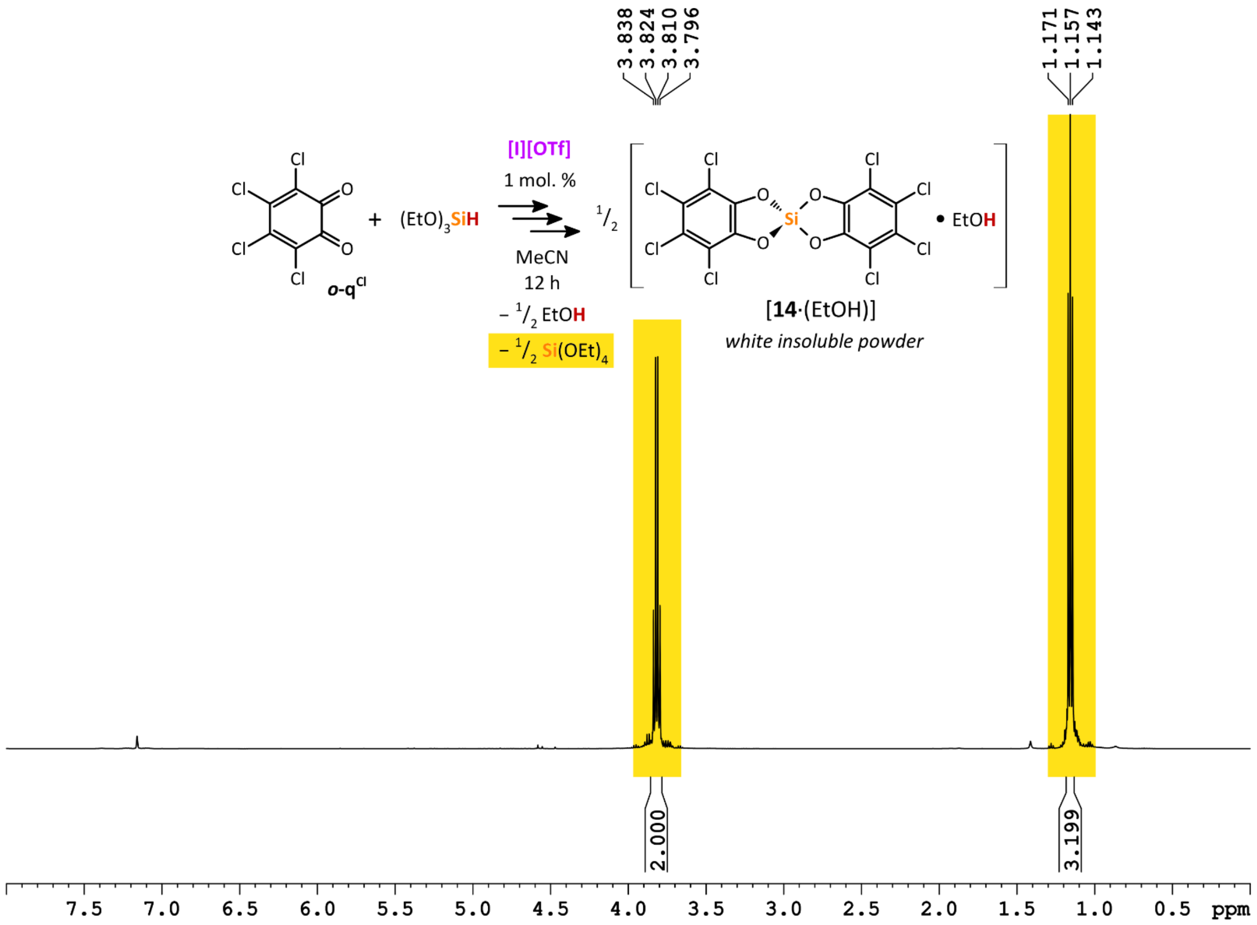

Figure S104: **^{1}H NMR** spectrum of evaporated solution (mother liquor) from white insoluble powder [**14**·(EtOH)] showing presence of $(EtO)_4Si$ (500.20 MHz, 295 K, C_6D_6). *Note:* Spectrum contains no signal for EtOH as it was completely evaporated with the solvent removal step.

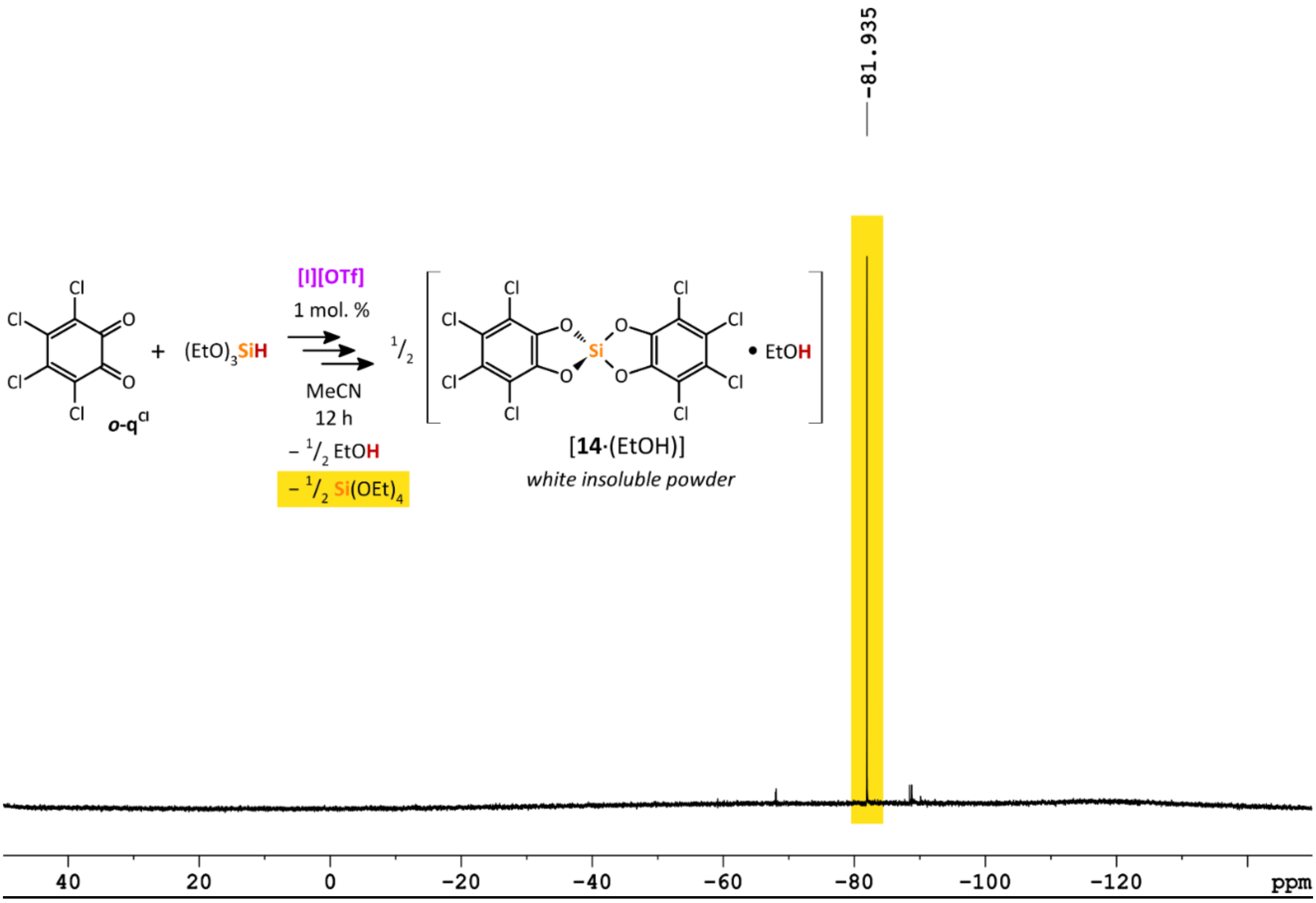

Figure S105: $^{29}Si\{^{1}H\}$ NMR spectrum of evaporated solution (mother liquor) from white insoluble powder [**14**·(EtOH)] showing presence of $(EtO)_4Si$ (99.37 MHz, 295 K, C_6D_6).

NMR spectra of compound [14·(dmso)$_2$]

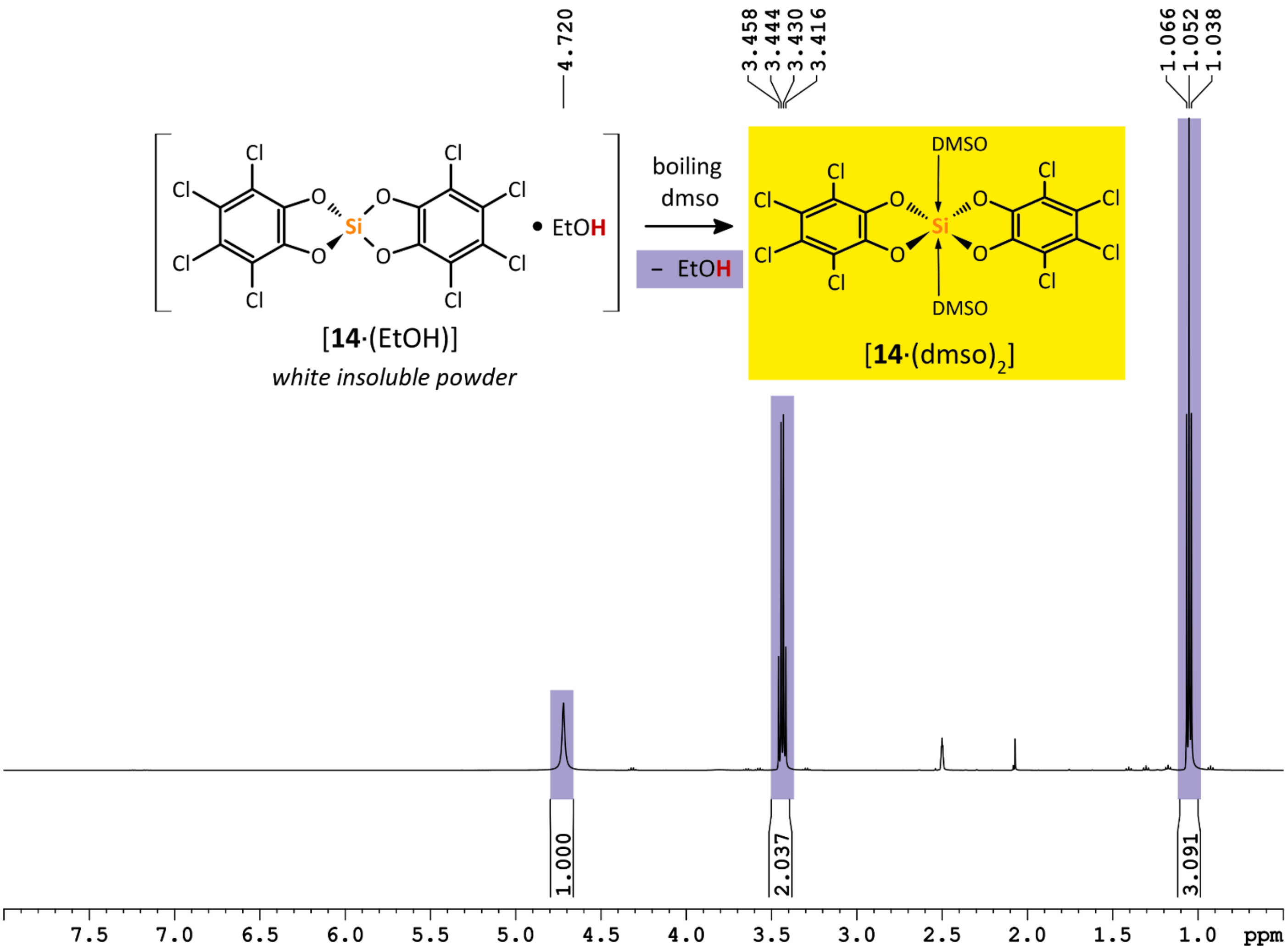

Figure S106: ^{1}**H NMR** spectrum of [**14**·(dmso-*d*$_6$)$_2$] in mixture with EtOH in DMSO-*d*$_6$ (500.20 MHz, 295 K).

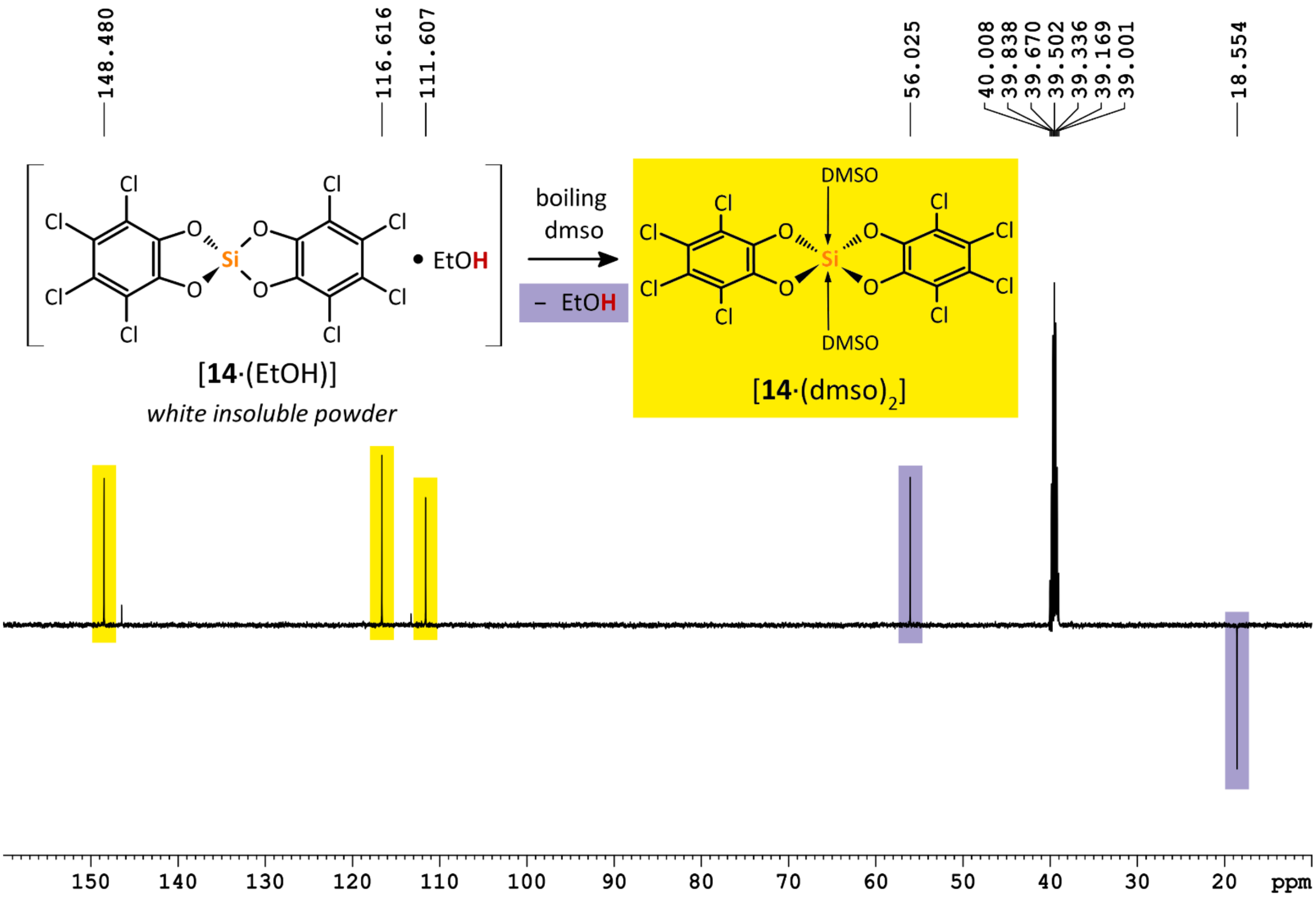

Figure S107: ^{13}**C{^{1}H} APT NMR** spectrum of [**14**·(dmso-*d*$_6$)$_2$] in mixture with EtOH in DMSO-*d*$_6$ (125.78 MHz, 295 K).

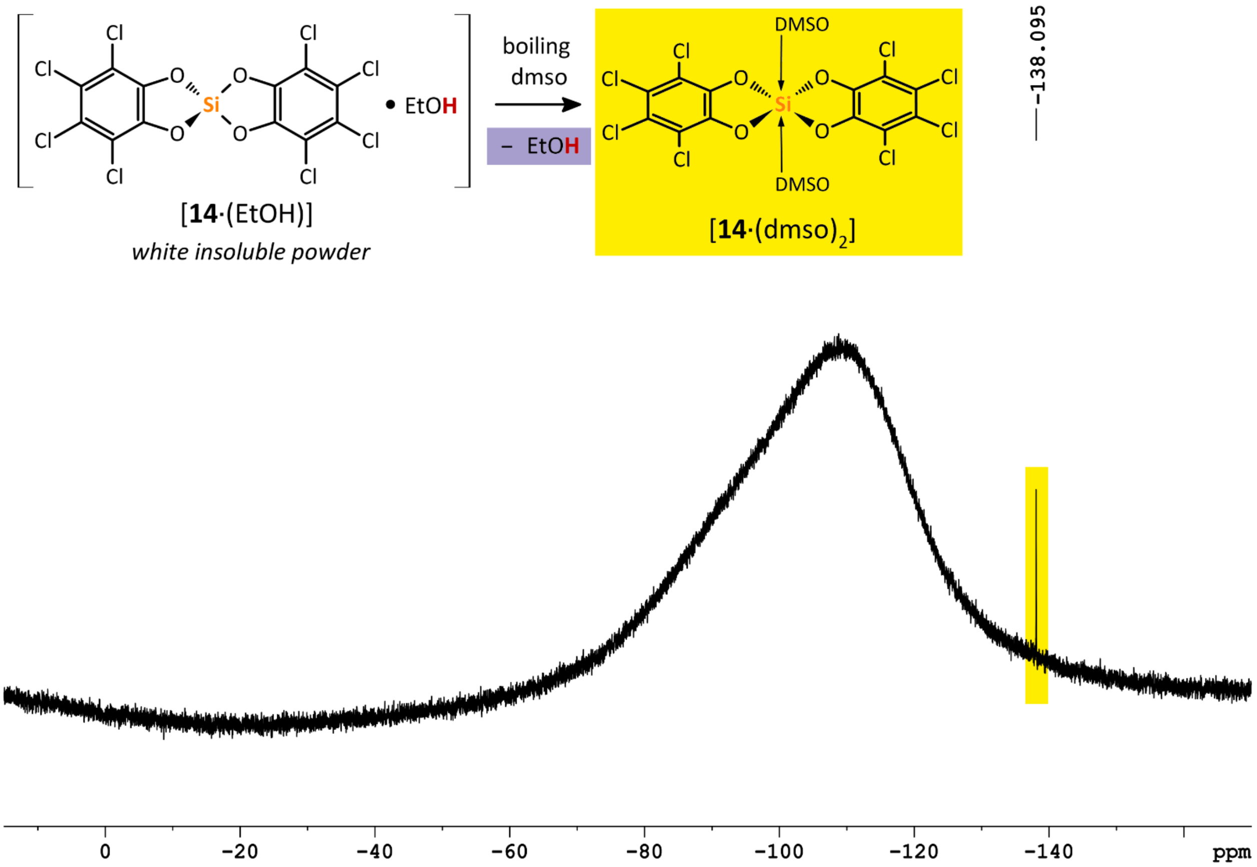

Figure S108: ^{29}**Si{^{1}H} NMR** spectrum of [**14**·(dmso-*d*$_6$)$_2$] in mixture with EtOH in DMSO-*d*$_6$ (99.37 MHz, 295 K, NS = 1024).

NMR spectra of compound 15

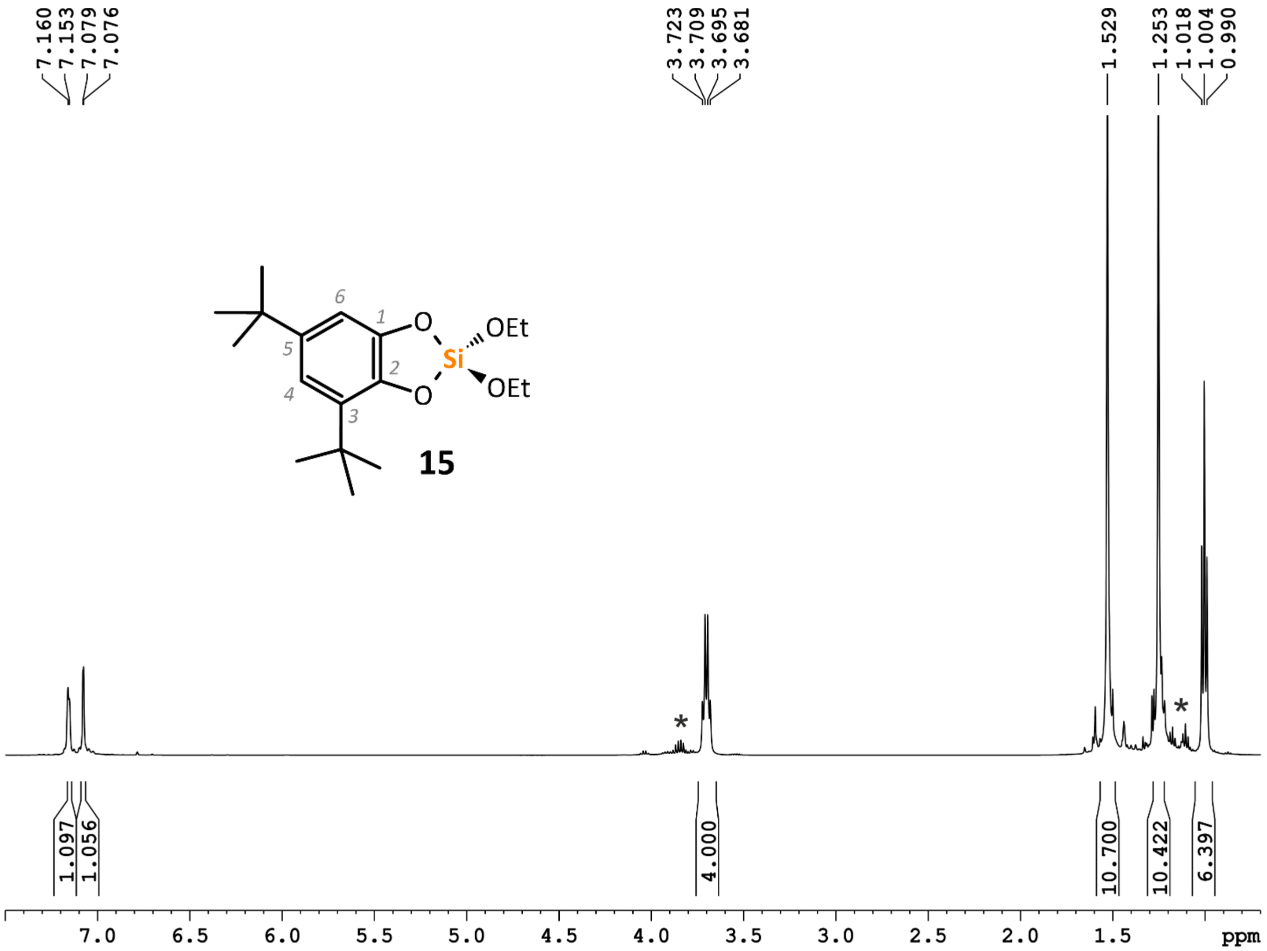

Figure S109: [1]**H NMR** spectrum of crude oily compound **15** with traces of $(EtO)_4Si$ denoted by * and other impurities due to ongoing subsequent reaction (500.20 MHz, 295 K, C_6D_6).

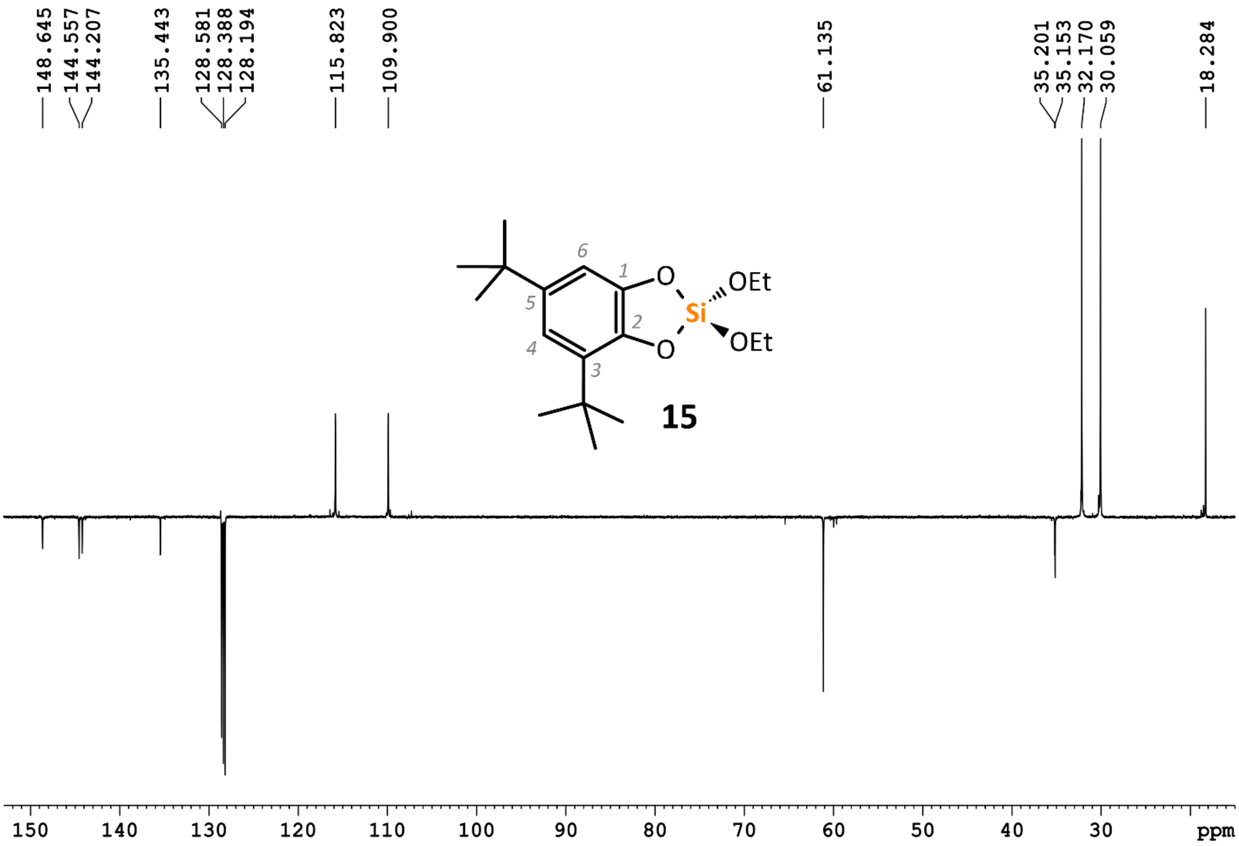

Figure S110: ^{13}C{^{1}H} APT NMR spectrum of crude oily compound **15** with traces of $(EtO)_4Si$ and other impurities due to ongoing subsequent reaction (125.78 MHz, 295 K, C_6D_6).

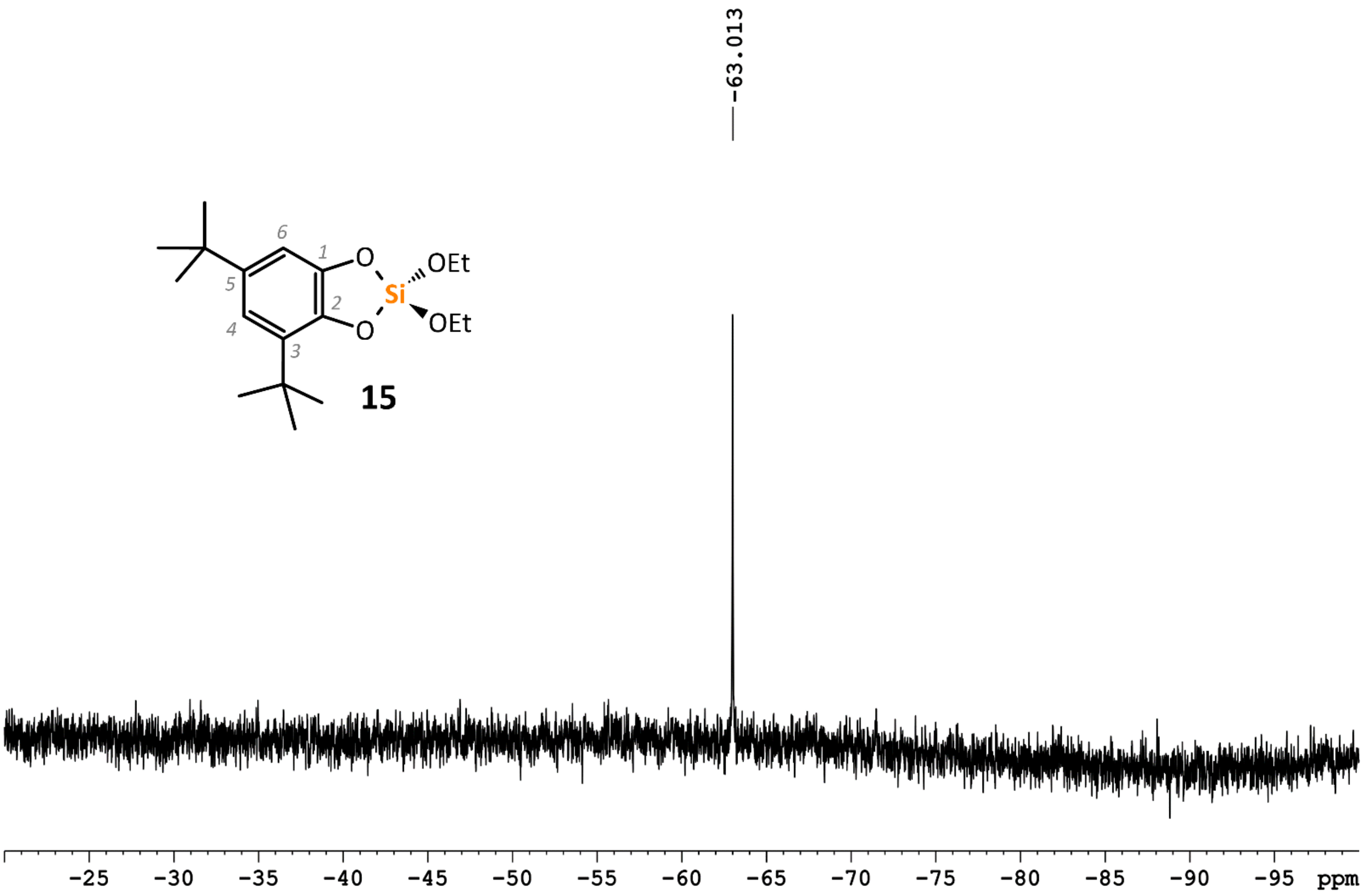

Figure S111: ^{29}Si{^{1}H} NMR spectrum of crude oily compound **15** with traces of $(EtO)_4Si$ and other impurities due to ongoing subsequent reaction (99.37 MHz, 295 K, C_6D_6).

NMR spectra of compound [16·(dmso-d₆)₂]

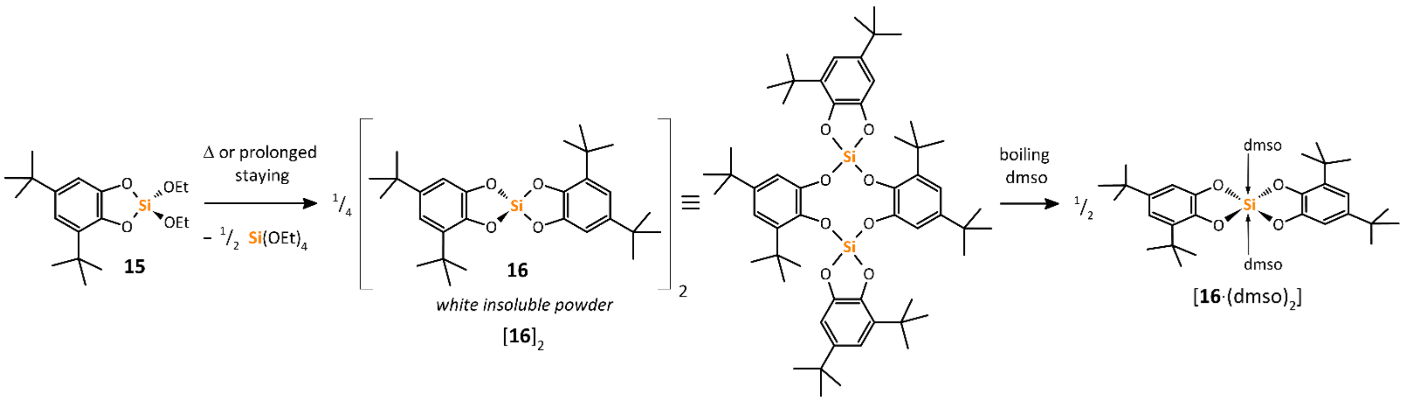

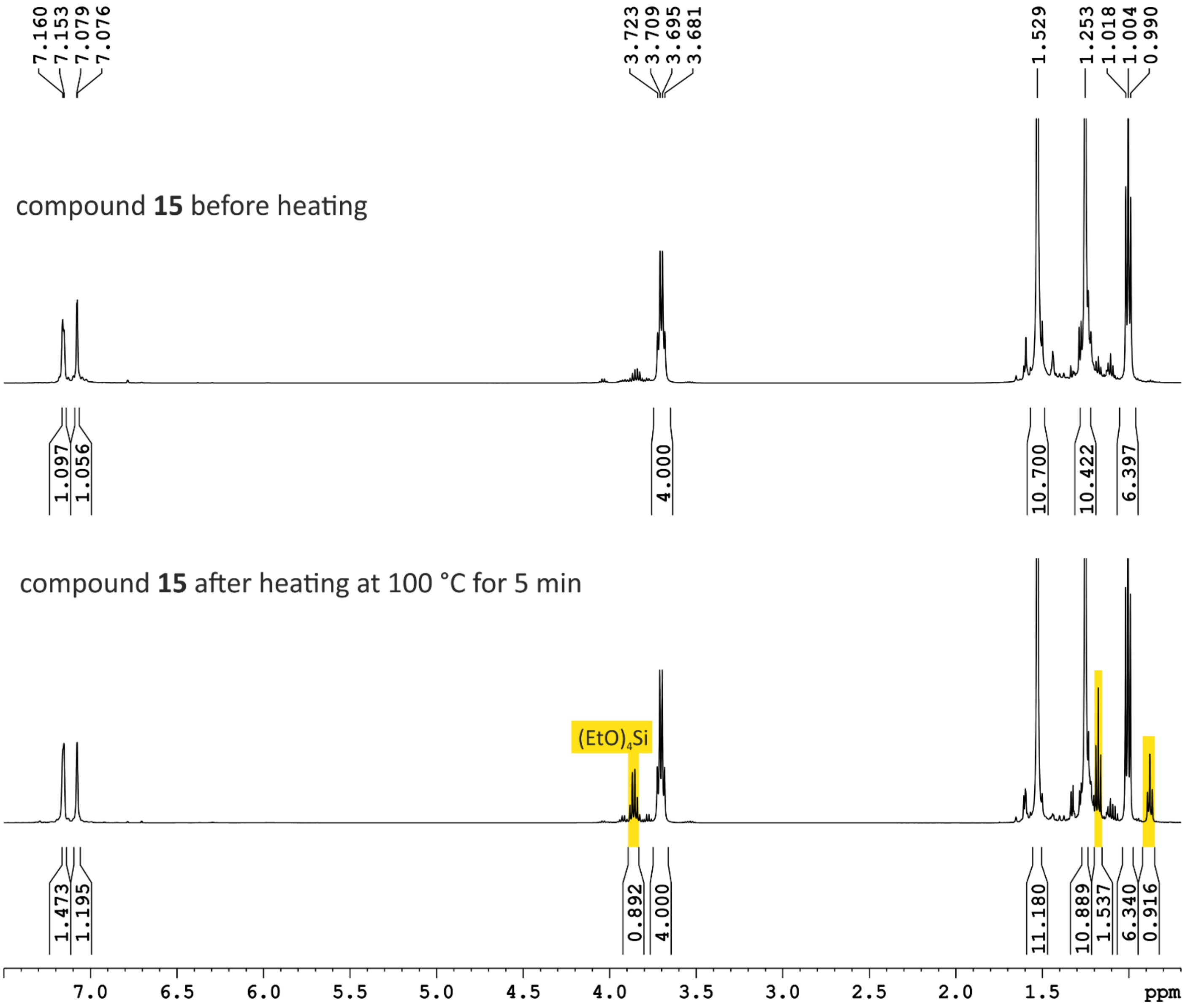

Figure S112: [1]**H NMR** spectrum of compound **15** (top) and after heating (bottom) showing elimination of $(EtO)_4Si$. Signals for [**16**]$_2$ as a co-product are not present as this white powder is insoluble in C_6D_6 (500.20 MHz, 295 K, C_6D_6).

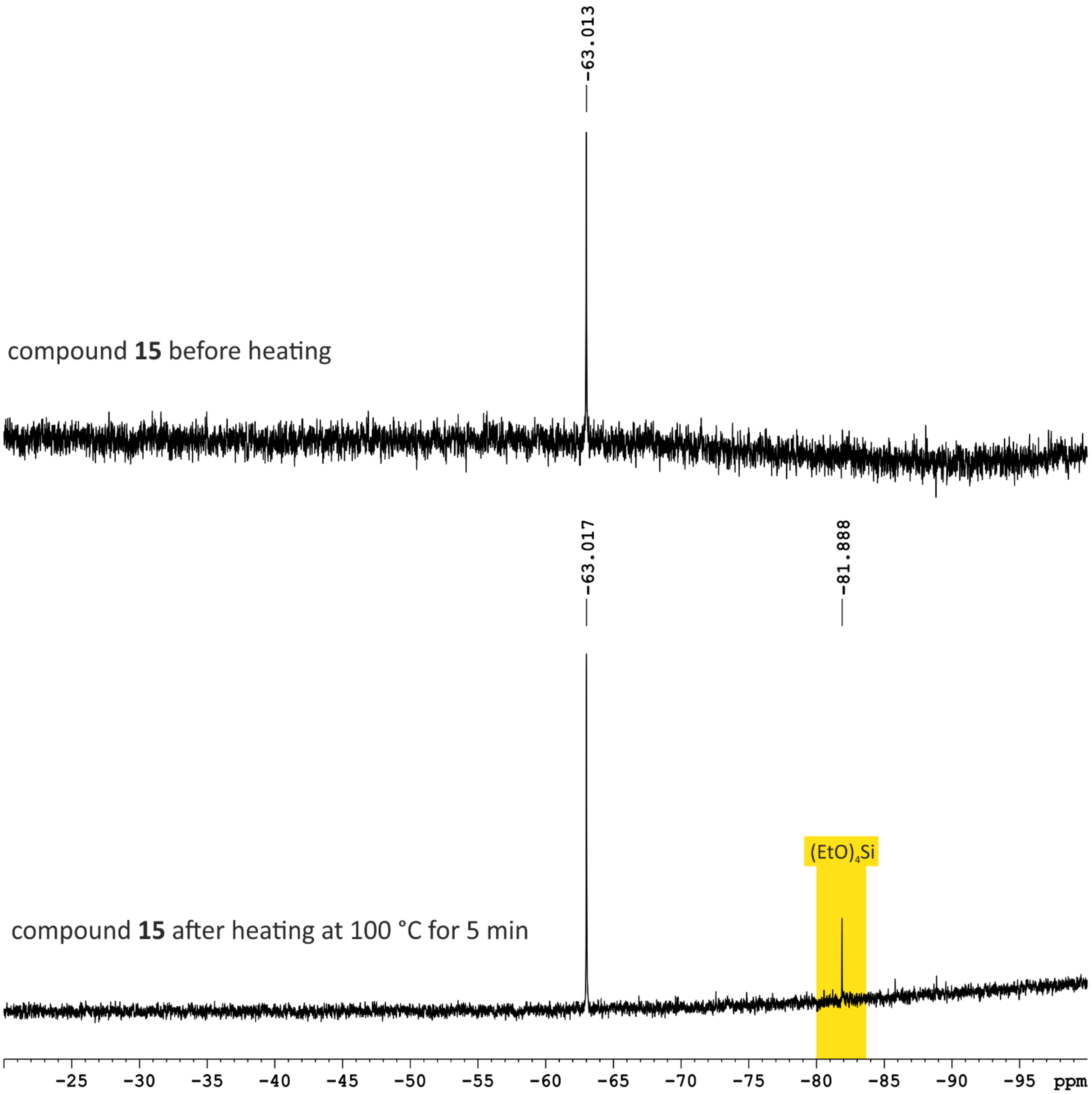

Figure S113: $^{29}Si\{^1H\}$ NMR spectrum of compound **15** (top) and after heating (bottom) showing elimination of $(EtO)_4Si$. Signals for [**16**]$_2$ as a co-product are not present as this white powder is insoluble in C_6D_6 (99.37 MHz, 295 K, C_6D_6).

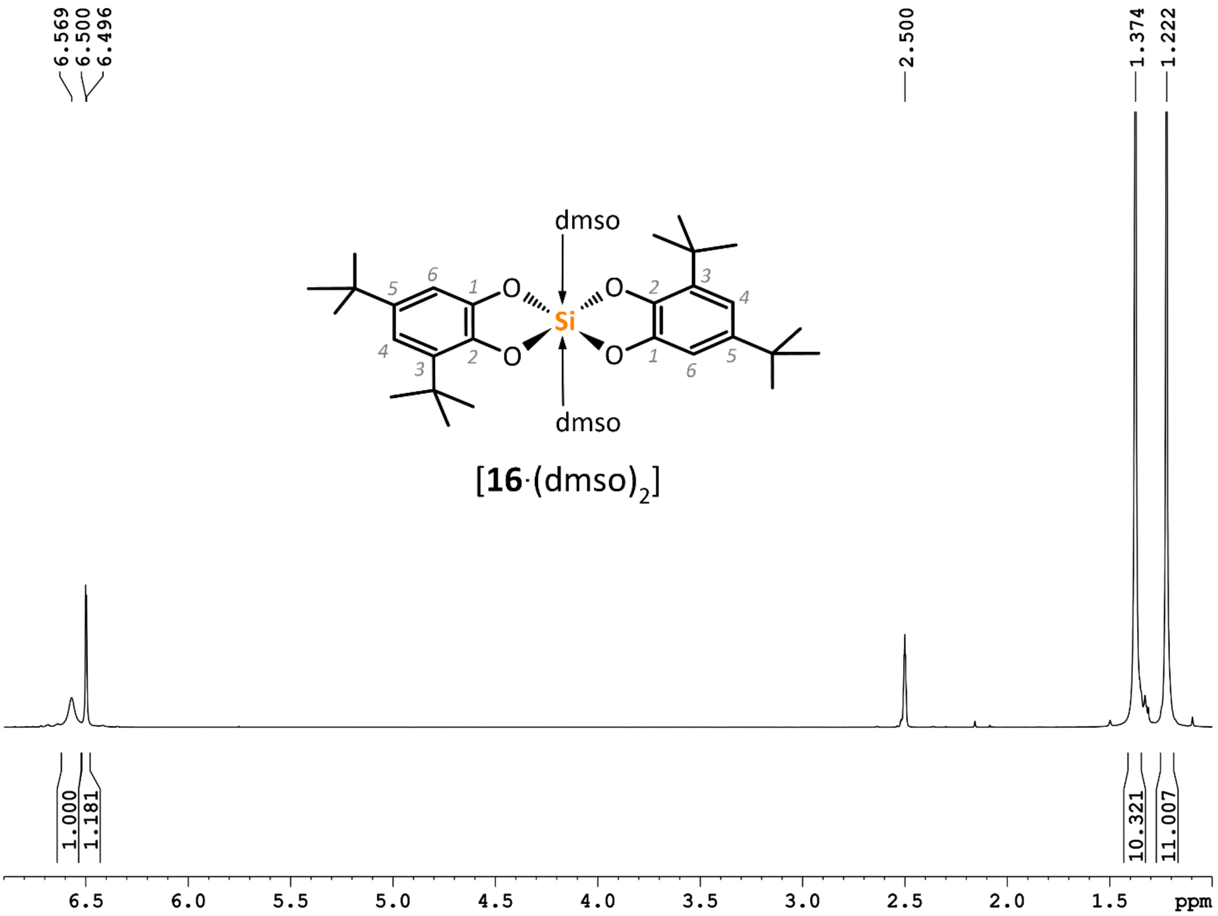

Figure S114: [1]**H NMR** spectrum of isolated [**16**·(dmso)$_2$] in DMSO-d_6 (500.20 MHz, 295 K).

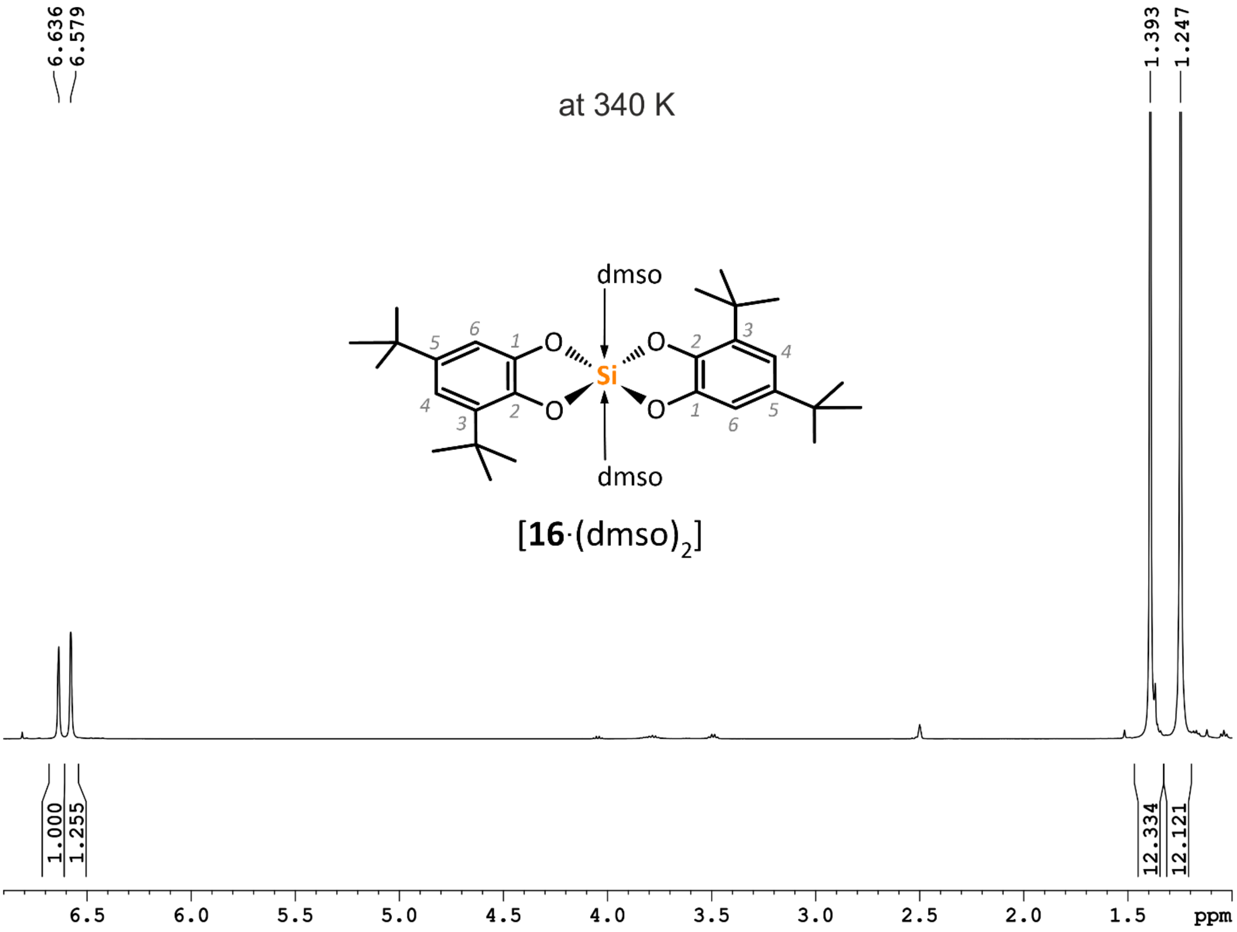

Figure S115: [1]**H NMR** spectrum of isolated [**16**·(dmso)$_2$] in DMSO-d_6 (500.20 MHz, 340 K).

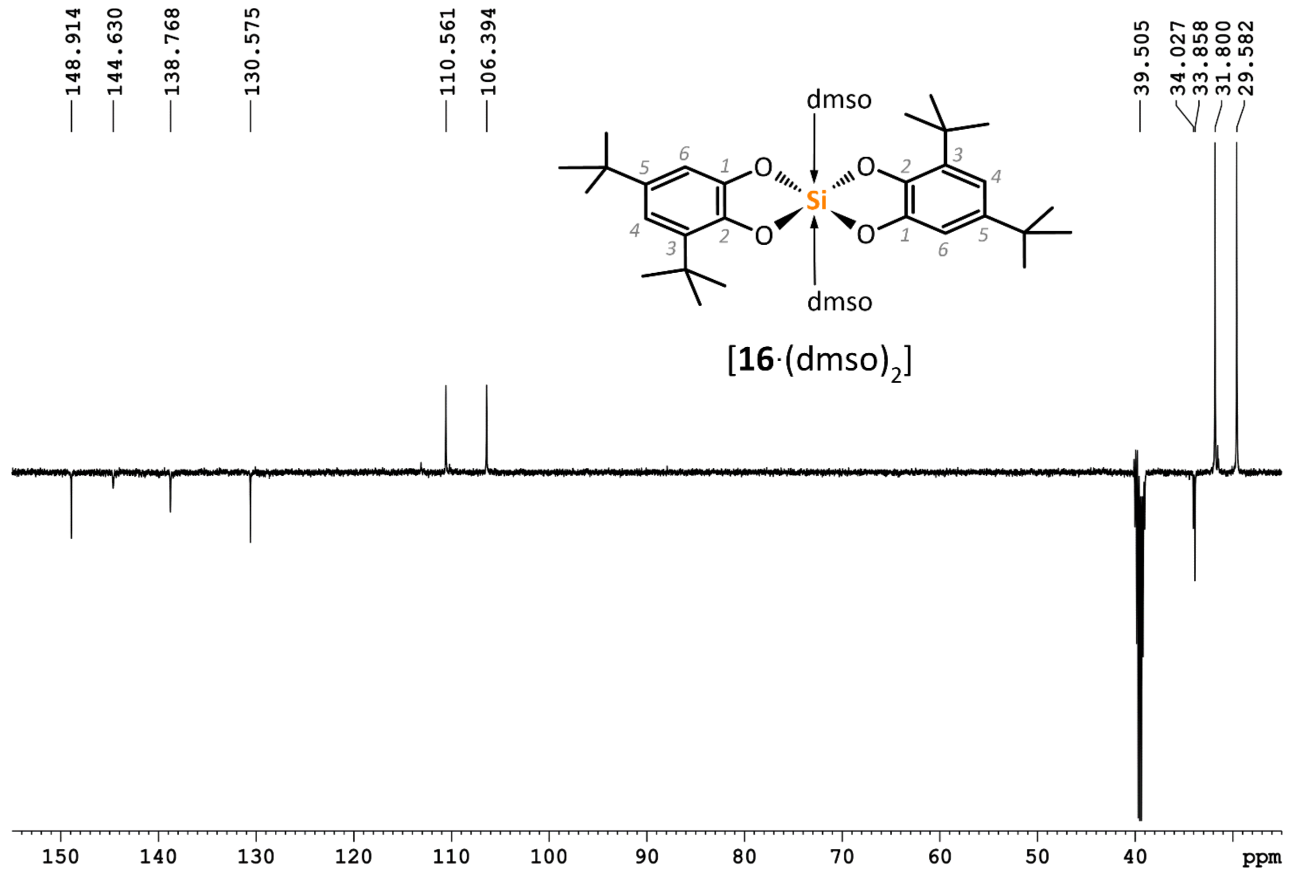

Figure S116: $^{13}C\{^1H\}$ APT NMR spectrum of isolated [**16**·(dmso)$_2$] in DMSO-d_6 (125.78 MHz, 295 K).

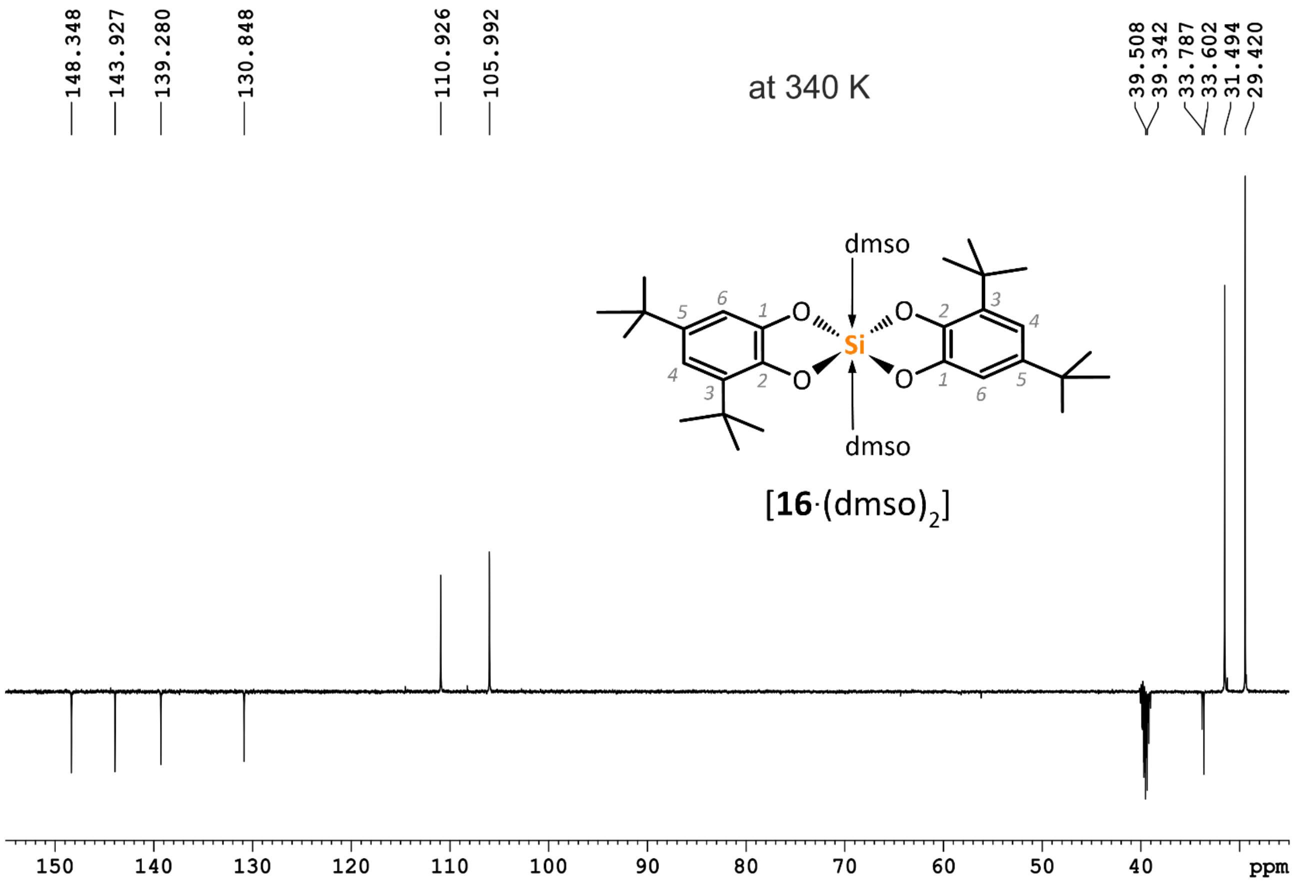

Figure S117: $^{13}C\{^1H\}$ APT NMR spectrum of isolated [**16**·(dmso)$_2$] in DMSO-d_6 (125.78 MHz, 340 K).

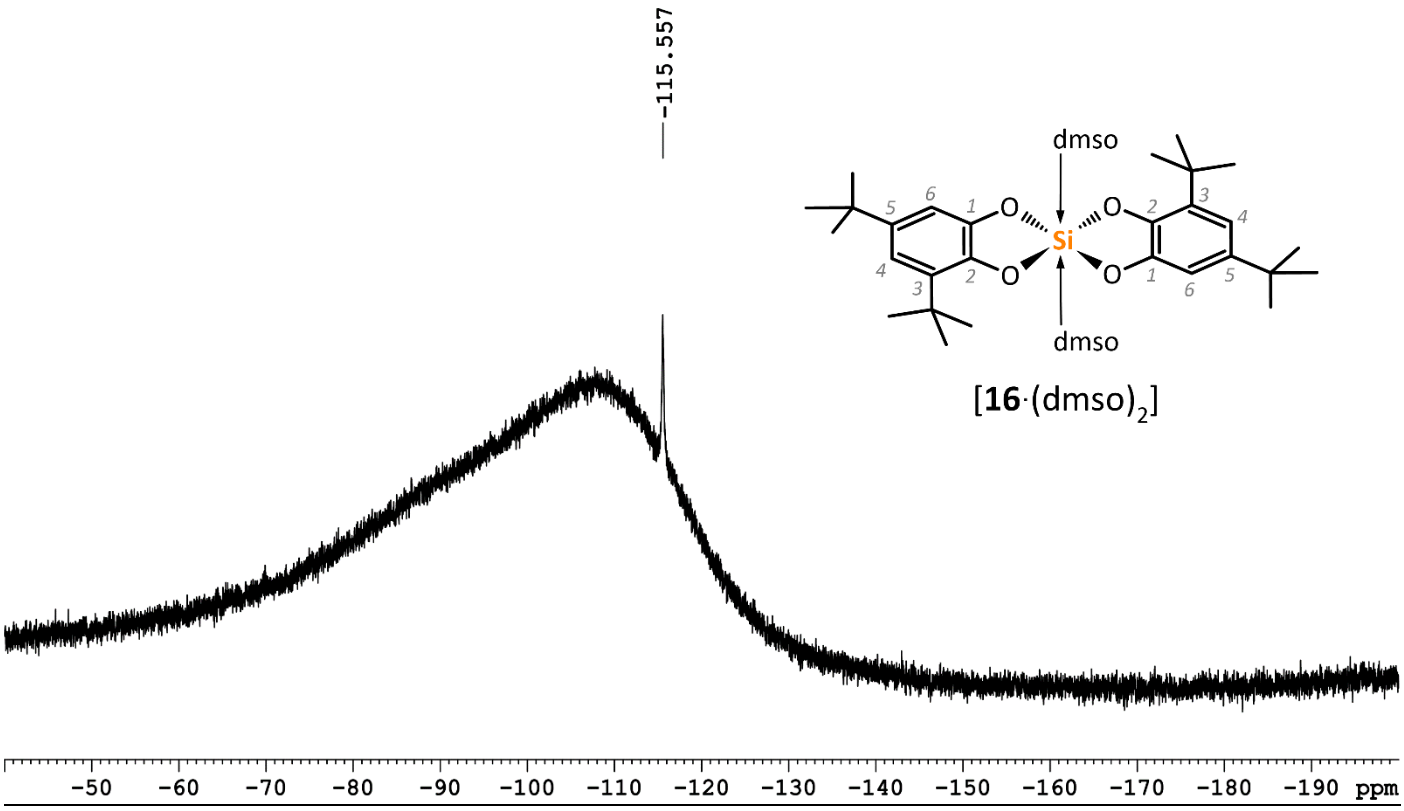

Figure S118: ^{29}Si{^{1}H} NMR spectrum of isolated [**16**·(dmso)$_2$] in DMSO-d_6 (99.37 MHz, 340 K, NS = 800). No signal was obtained when measured at 295 K.

Mechanistic study of the catalysis

To shed some light on the mechanism of the catalysis, we examined the reaction leading to silylated catechol **2** by performing control stoichiometric reactions on all three components of the reaction, i.e. Et_3SiH, **[I][OTf]** and ***o*-q^{tBu}**. When 1 eq. of Et_3SiH was added to ongoing dynamic equilibrium of **[I][OTf]** + ***o*-q^{tBu}** ⇌ 0.35 ***a*-[I(cat^{tBu})][OTf]** + 0.65 ***b*-[I(cat^{tBu})][OTf]**, a clean formation of silylated catechol **2** next to regenerated **[I][OTf]** was obtained within few seconds as judged by NMR spectroscopy (**Figure S119**). On the other hand, when 1 eq. of ***o*-q^{tBu}** was added to in-situ generated 1:1 mixture of mono-iminium ditelluride **II** and Et_3SiOTf (from the reaction of 2 eq. **[I][OTf]** and Et_3SiH; see **Scheme 1A** in the main text), a silylated catechol **2** was immediately formed again, next to regenerated 2 eq. of **[I][OTf]** (**Figure S120**). Performing stoichiometric reaction of isolated mono-iminium ditelluride **II** with ***o*-q^{tBu}**, a new oxonium species **III^{tBu}** is formed (**Figure S121** and following figures)**.** Importantly, ad addition of equivalent of Et_3SiOTf to oxonium species **III^{tBu}** leads to quantitative formation of **[I][OTf]** and silylated catechol **2** (**Scheme S5**).

These result points to analogous mechanism of the catalysis as determined for *para*-quinones being both oxidants and substrates.[2] This means the catalysis is initiated by redox reaction of 2 eq. of **[I][OTf]** with Et_3SiH yielding **II** and Et_3SiOTf. Then a redox reaction of in-situ generated **II** being the reducing species for ***o*-q^{tBu}**, resulting in formation of oxonium species **III^{tBu}**, which is attacked by the in-situ generated Et_3SiOTf. This results in formation of silylated catechol **2** and recovery of the catalyst **[I][OTf]** (see **Scheme S6** below for full mechanism). However, the whole process is complicated by side-reaction of ***o*-q^{tBu}** with **[I][OTf]** leading to dynamic equilibrium of **[I][OTf]** + ***o*-q^{tBu}** ⇌ 0.35 ***a*-[I(cat^{tBu})][OTf]** + 0.65 ***b*-[I(cat^{tBu})][OTf]**. This means that by formation of Te(IV) species ***a/b*-[I(cat^{tBu})][OTf]**, there is a temporary "storage buffer" for both the catalyst **[I][OTf]** and ***o*-q^{tBu}**.

It is to note that we also tested whether **[I]Cl** or **[I][SbF$_6$]** can serve as the catalyst in for the reaction of Et_3SiH and ***o*-q^{tBu}**. While **[I][SbF$_6$]** turned out to be comparably active to **[I][OTf]**, the neutral **[I]Cl** did not show any catalytic activity. These results correspond to the premise that the redox catalysis is based on tellurenyl cation which in-situ generates **II** (**Scheme 1A** in the main text).

Addition of 1 eq. of Et_3SiH into a dynamic equilibrium of [I][OTf] + o-q^{tBu} ⇌ a-[I(cat^{tBu})][OTf] + b-[I(cat^{tBu})][OTf] (a stoichiometric reaction)

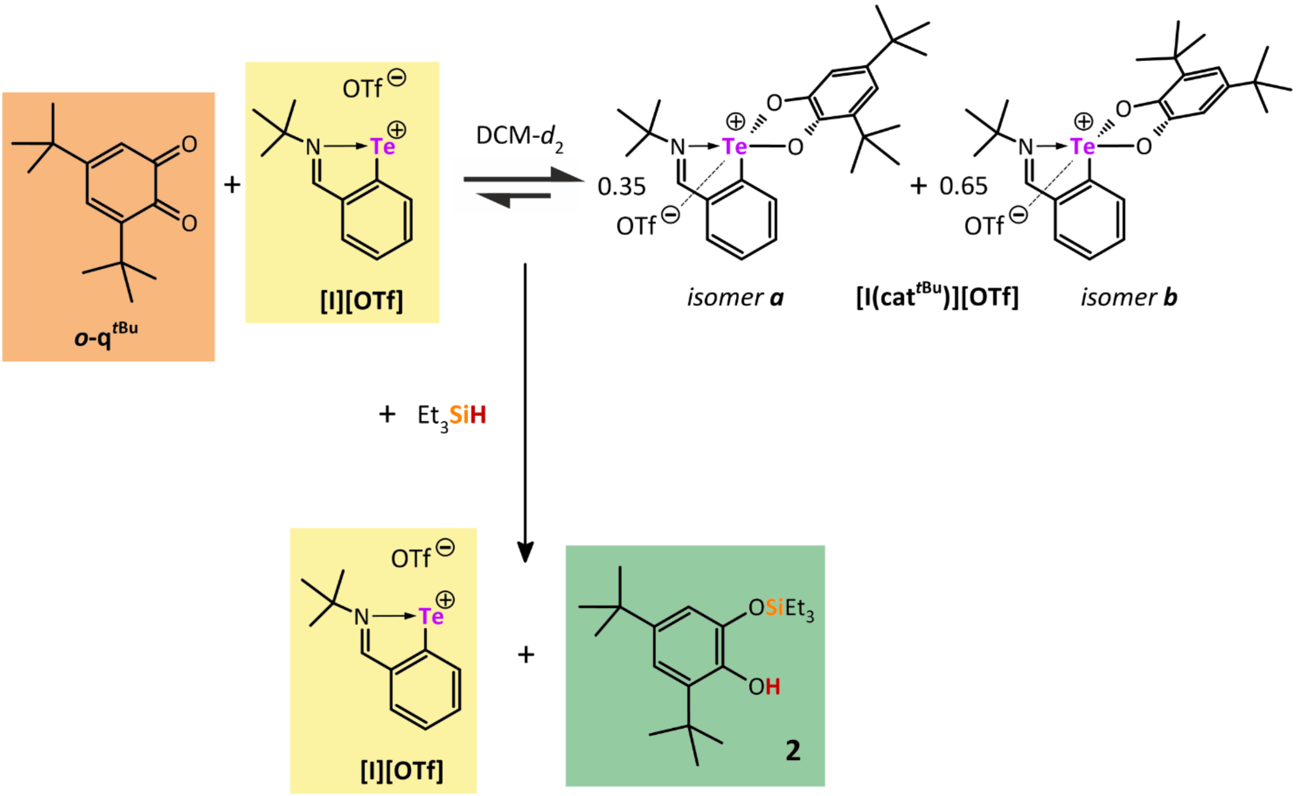

Scheme S2: An addition of 1 eq. of Et_3SiH into a dynamic equilibrium of **[I][OTf]** + ***o*-q^{tBu}** ⇌ 0.35 ***a*-[I(cat^{tBu})][OTf]** + 0.65 ***b*-[I(cat^{tBu})][OTf]** resulting in the formation of silylated catechol **2** and regeneration of **[I][OTf]**.

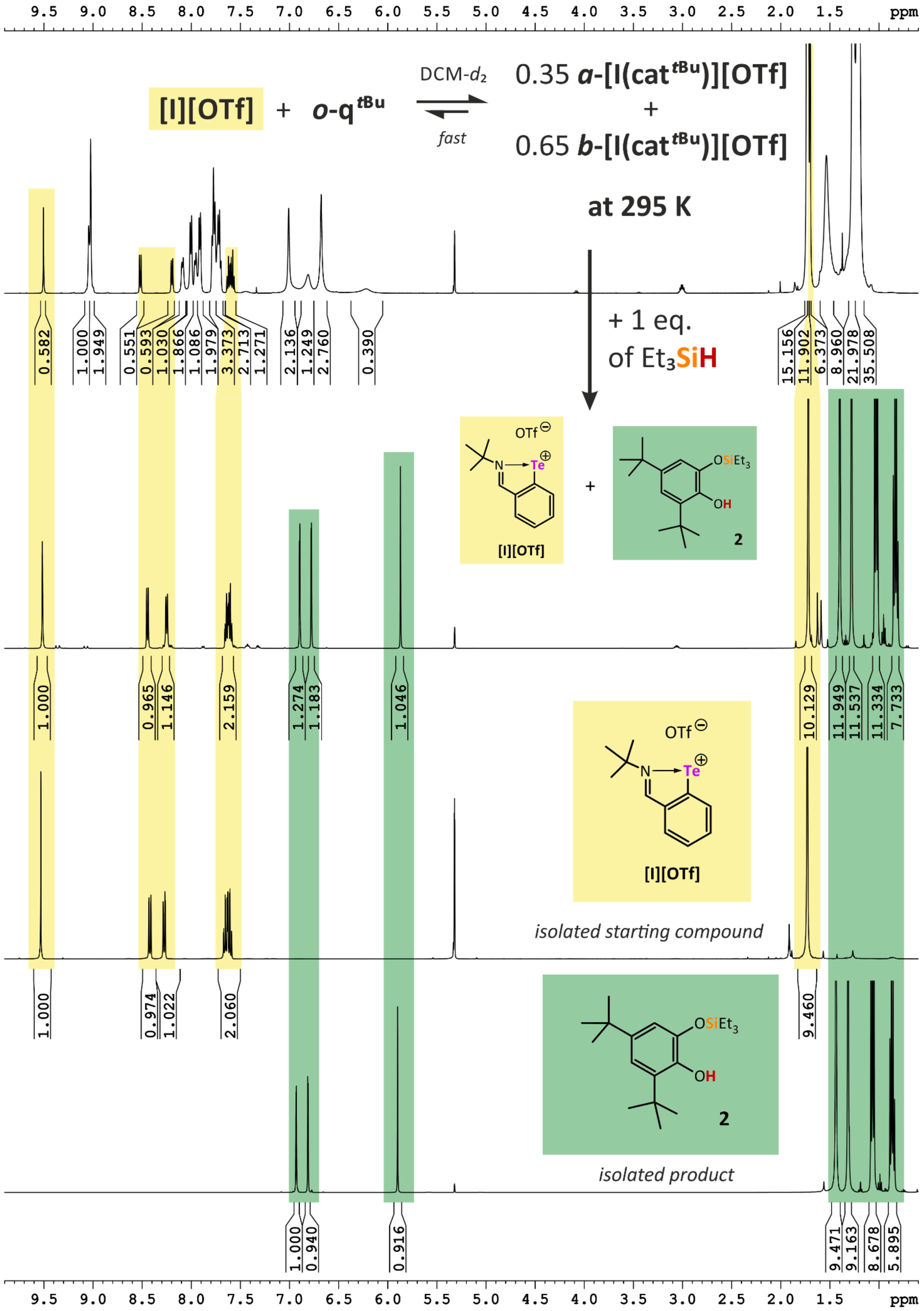

Figure S119: Stacked plot of ^{1}**H NMR** spectra of a dynamic equilibrium of **[I][OTf]** + ***o*-q^{tBu}** ⇌ 0.35 ***a*-[I(cat^{tBu})][OTf]** + 0.65 ***b*-[I(cat^{tBu})][OTf]** in DCM-d_2 (top) and after addition of 1 eq. of Et_3SiH (the 2nd spectrum from the top) showing regeneration of starting compound **[I][OTf]** along with formation of catecholatosilane **2** (500.20 MHz, 295 K).

Addition of 1 eq. of o-q^{tBu} into a mixture of II and Et_3SiOTf (a stoichiometric reaction)

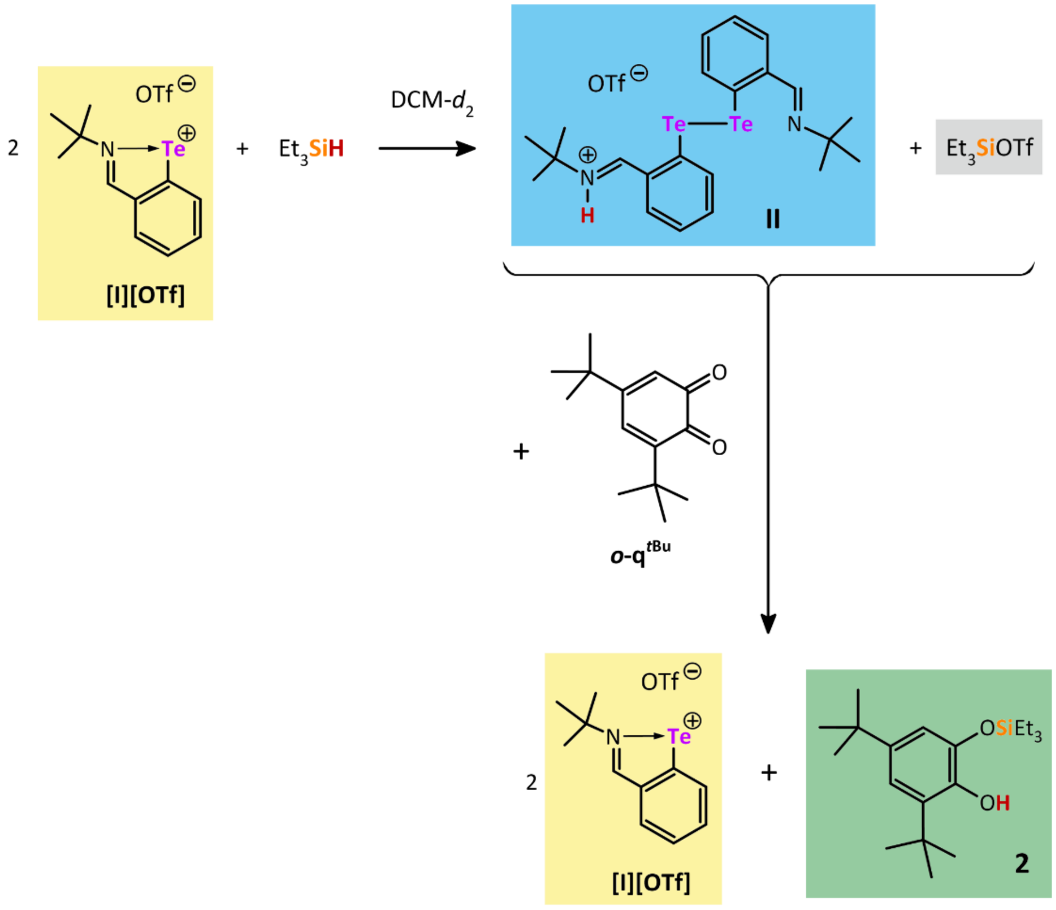

Scheme S3: An addition of 1 eq. of ***o*-q^{tBu}** into a 1:1 molar mixture of mono-iminium ditelluride **II** and Et_3SiOTf resulting in the formation of silylated catechol **2** and regeneration of **[I][OTf]**.

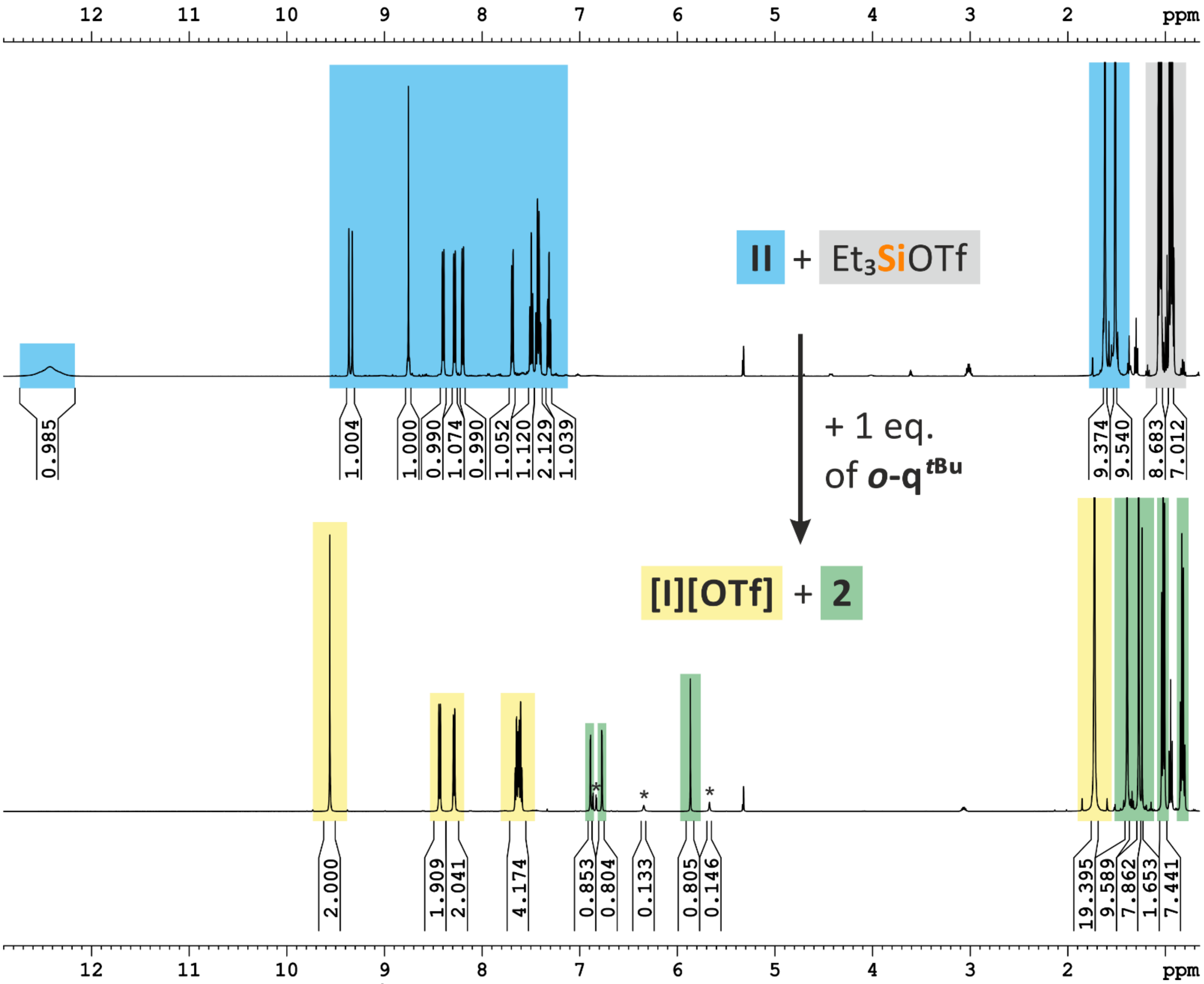

Figure S120: Stacked plot of [1]**H NMR** spectra of mixture of mono-iminium ditelluride **II** and Et_3SiOTf generated in-situ from 2 eq. of **[I][OTf]** and Et_3SiH in DCM-d_2 (top) and after addition of 1 eq. of ***o*-q**^{tBu} (bottom) showing regeneration of starting compound **[I][OTf]** along with formation of catecholatosilane **2** (500.20 MHz, 295 K).***** These signals corresponds to non-silylated catechol (3,5-di-*tert*-butylcatechol) formed by unintentional partial hydrolysis. See **Figure S119** above for comparison showing formation of the same final products regardless on order of added reagents.

Stoichiometric reaction of ditelluride II and o-q^{tBu} leading to formation of oxonium species III^{tBu}

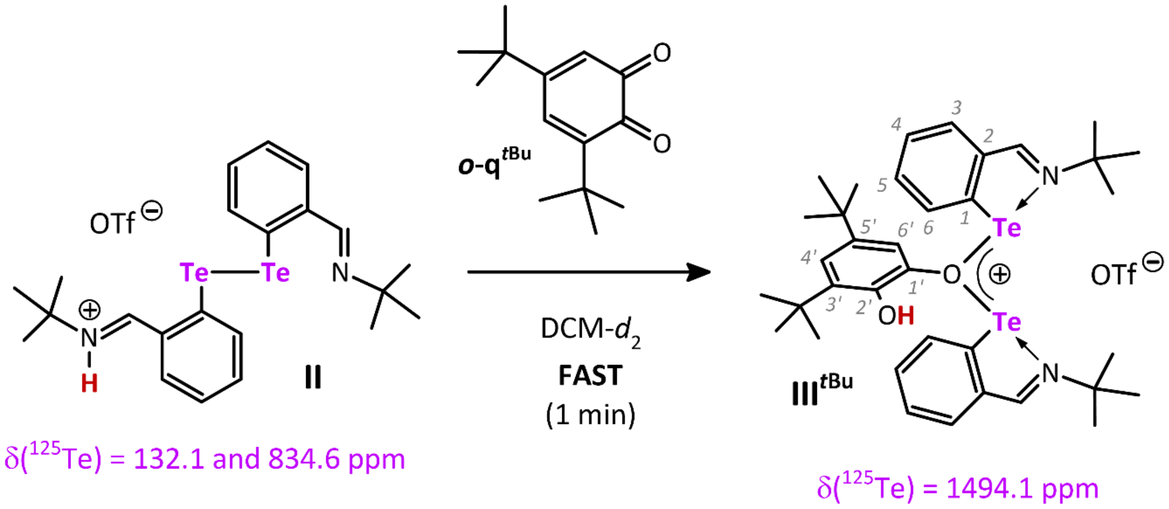

Scheme S4: A quantitative formation of light-yellow oxonium species **III^{tBu}** in the reaction of red mono-iminium ditelluride **II** with ***o*-q^{tBu}**.

NMR data for **III^{tBu}**:

^{1}H NMR (500.20 MHz, DCM-d_2) δ (ppm): 1.21 [9H, s, (C*H*$_3$)$_3$C-C5']; 1.43 [9H, s, (C*H*$_3$)$_3$C-C3']; 1.67 [18H, s, 2x (C*H*$_3$)$_3$C-N]; 6.58 [1H, br. s, O*H*]; 6.73 [1H, dd, Ar(C4')*H*]; 6.78 [1H, dd, Ar(C6')*H*]; 7.54 [2H, t, 2x Ar(C4)*H*]; 7.59 [2H, t, 2x Ar(C5)*H*]; 8.16 [2H, d, 2x Ar(C3)*H*]; 8.37 [2H, d, 2x Ar(C6)*H*]; 9.41 [2H, s, 2x C*H*=N]. **^{13}C{^{1}H} NMR** (125.78 MHz, DCM-d_2) δ (ppm): 29.9 [s, (*C*H$_3$)$_3$C-C3']; 31.9 [s, (*C*H$_3$)$_3$C-C5']; 32.4 [s, (*C*H$_3$)$_3$C-N]; 34.8 [s, qC, (CH$_3$)$_3$*C*-C5']; 35.1 [s, qC, (CH$_3$)$_3$*C*-C3']; 47.5 [s, qC, (CH$_3$)$_3$*C*-]; 63.9 [s, qC, (CH$_3$)$_3$*C*-N]; 112.5 [s, Ar-(*C6'*)H]; 114.0 [s, Ar-(*C4'*)H]; 120.5 [q, qC, F$_3$*C*-, $^{1}J(^{19}F, ^{13}C)$ = 320.1 Hz]; 127.5 [s, Ar(*C4*)H]; 132.3 [s, Ar(*C6*)H]; 132.4 [s, Ar(*C5*)H]; 133.3 [s, Ar(*C3*)H]; 136.9 [s, qC]; 141.4 [s, qC]; 143.5 [s, qC]; 162.4 [s, CH=*N*]. **^{15}N NMR** (40.54 MHz, DCM-d_2) δ: –107.9 ppm [CH=*N*→Te]. **^{125}Te{^{1}H} NMR** (157.79 MHz, DCM-d_2) δ: 1494.1 ppm [br. s].

The structure of oxonium species **III^{tBu}** is analogous to a oxonium species formed by reaction of ditelluride **II** and *para*-quinones.[2] The following NMR spectra for **III^{tBu}** in DCM-d_2 were obtained from crude reaction mixture resulting from the reaction between **II** and ***o*-q^{tBu}**. All attempts to crystallize **III^{tBu}** failed.

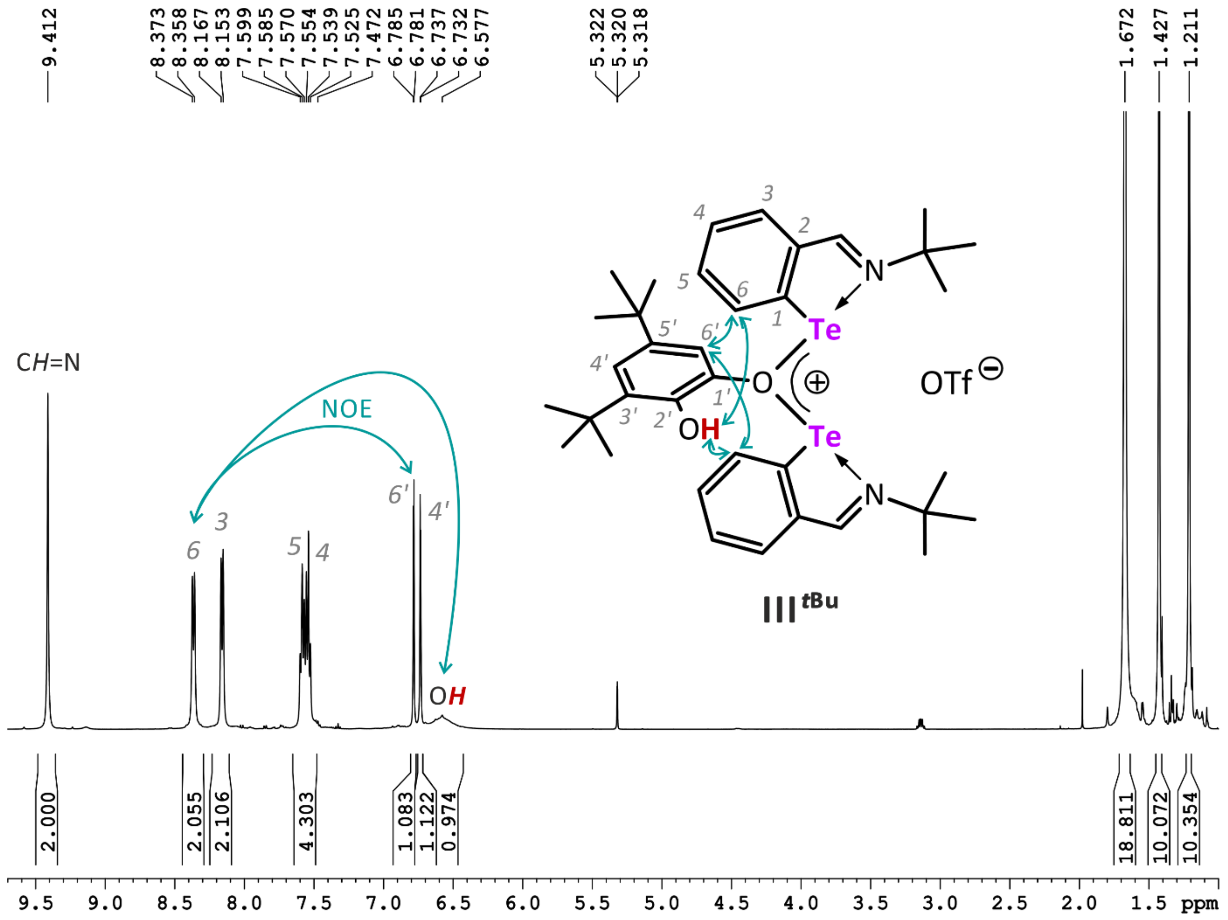

Figure S121: **^{1}H NMR** spectrum of oxonium species **III^{tBu}** in DCM-d_2 (500.20 MHz, 295 K).

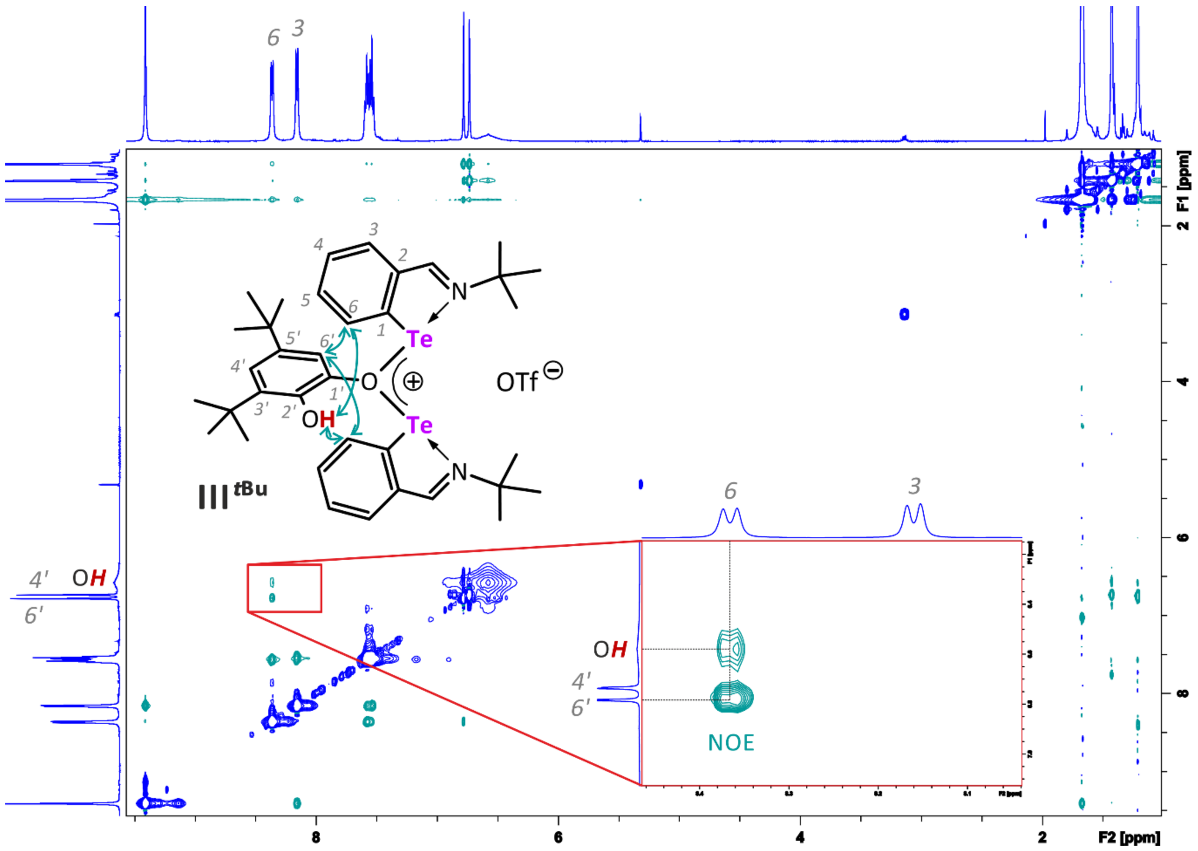

Figure S122: **^{1}H-^{1}H NOESY NMR** spectrum of **III^{tBu}** in DCM-d_2 (500.20 MHz, 295 K, d8 = 1 s).

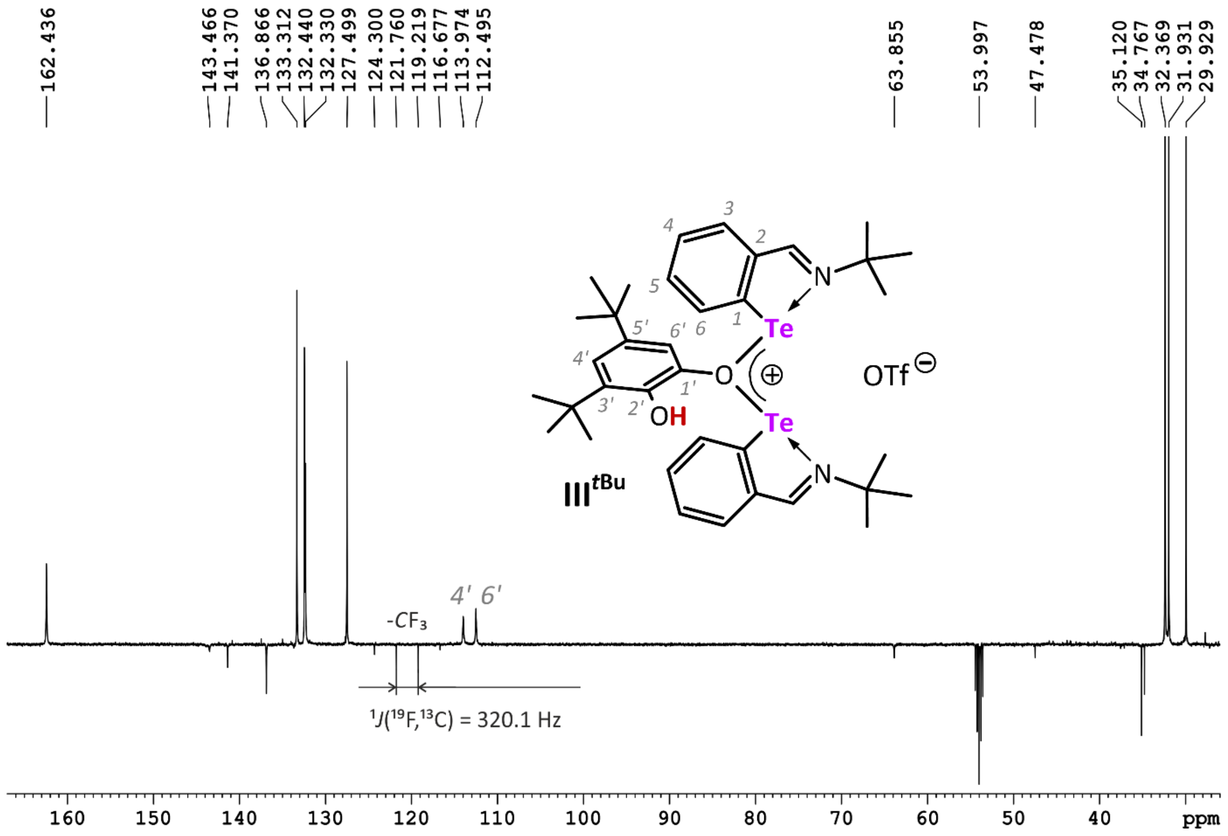

Figure S123: ^{13}**C{**^{1}**H} APT NMR** spectrum of oxonium species **III**^{tBu} in DCM-d_2 (125.78 MHz, 295 K).

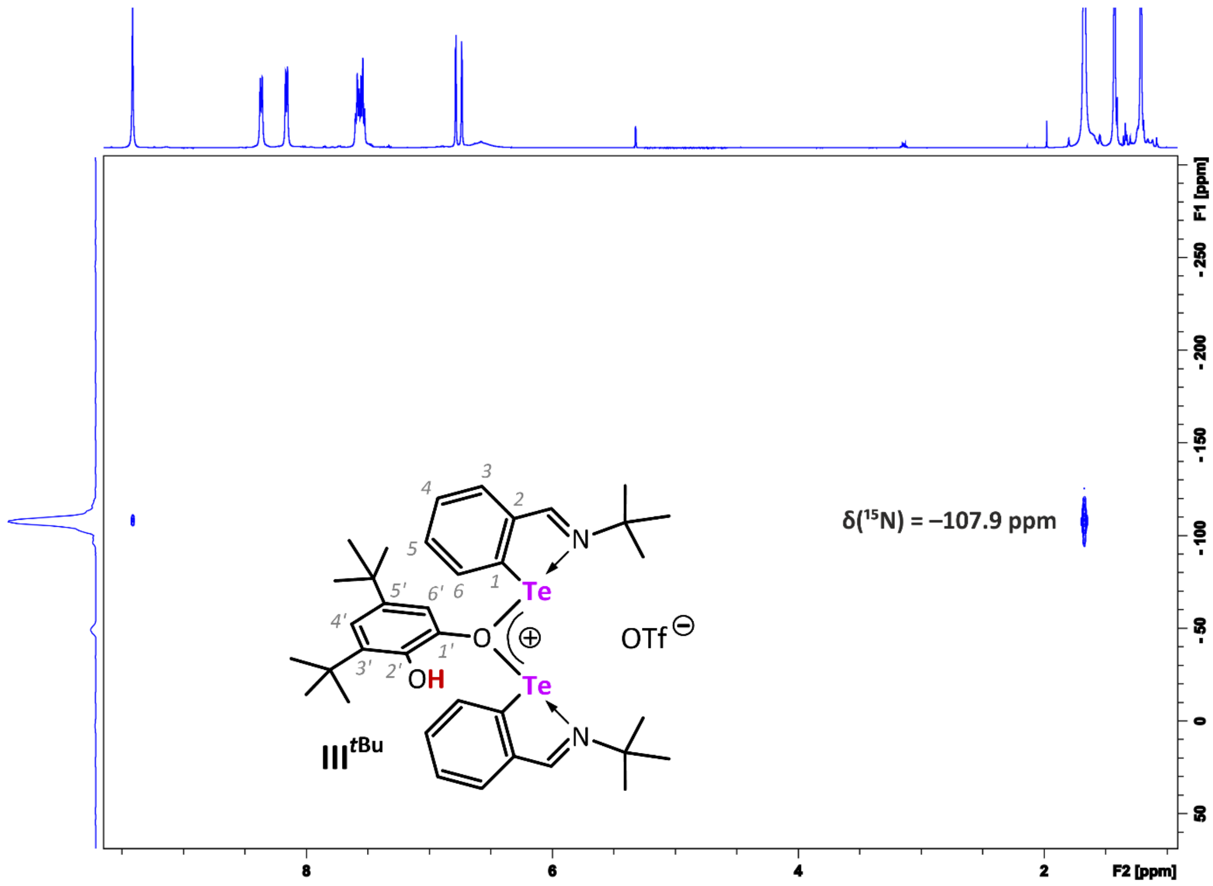

Figure S124: ^{1}**H-**^{15}**N HMBC NMR** spectrum of oxonium species **III**^{tBu} in DCM-d_2 (500.20 MHz, 295 K).

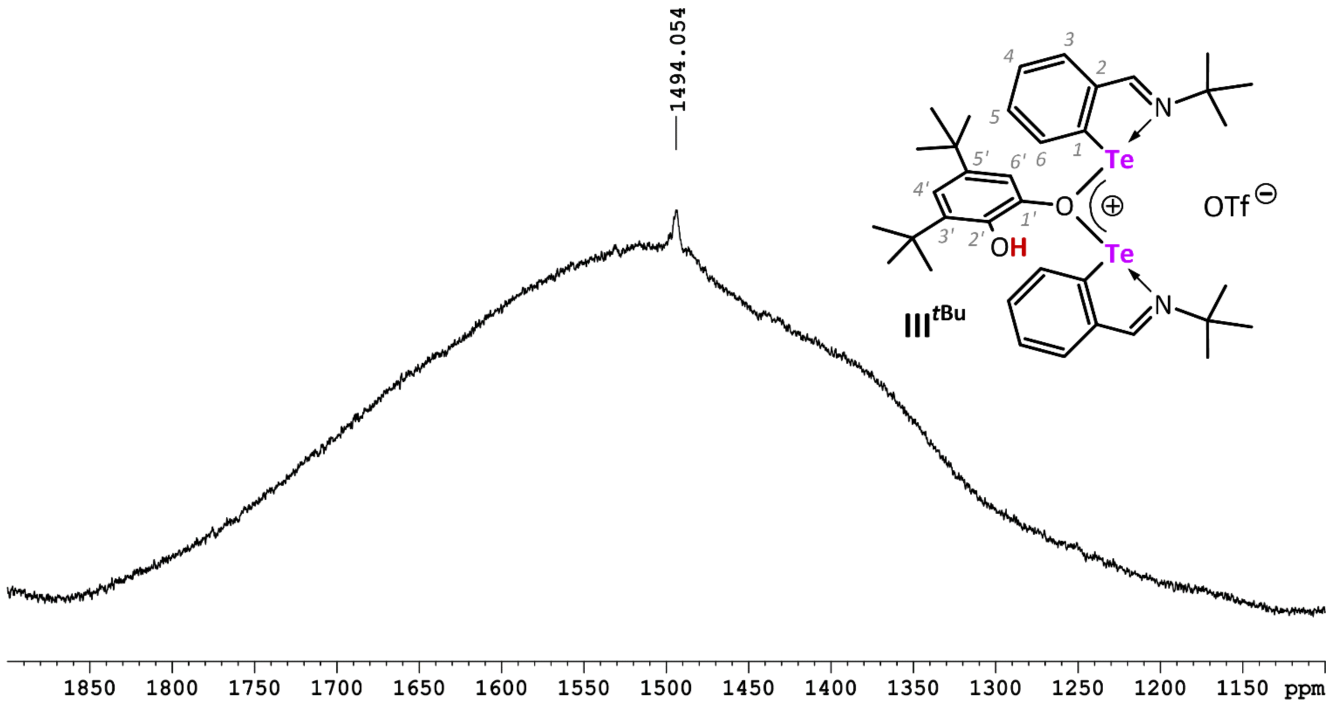

Figure S125: ^{125}**Te{**^{1}**H} NMR** spectrum of oxonium species **III**^{tBu} in DCM-d_2 (157.79 MHz, 295 K, NS = 102400).

Stoichiometric reaction of oxonium species III^{tBu} with Et_3SiOTf

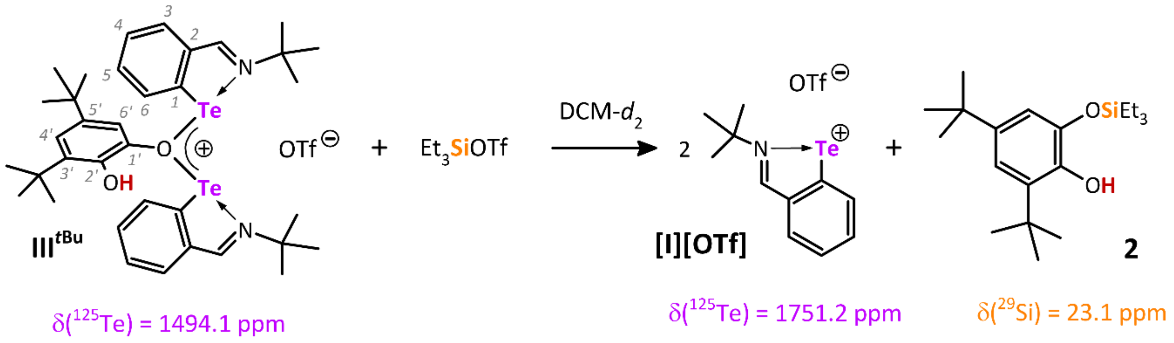

Scheme S5: A quantitative formation of silylated catechol **2** next to **[I][OTf]** in the reaction of oxonium species **III**^{tBu} with Et_3SiOTf. The reaction time is under 10 min.

Proposed catalytic cycle for the [I][OTf]-catalyzed reaction

Proposed catalytic cycle utilizing redox Si–H bond activation in Et_3SiH using tellurenyl triflate **[I][OTf]** as the catalyst with ***o*-q**^{tBu} as both oxidant and substrate is following:

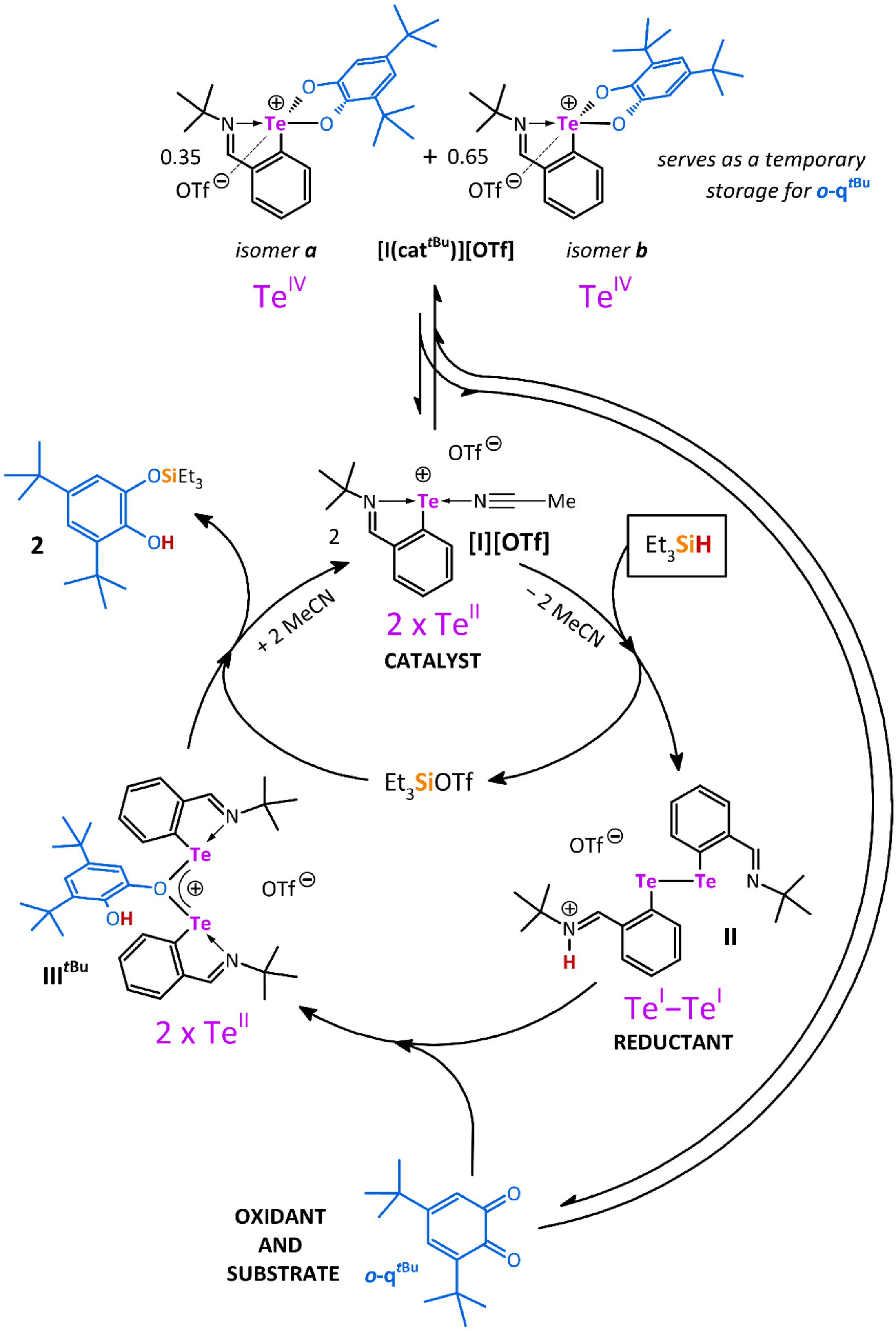

Scheme S6: A proposed catalytic cycle.

DFT computations

Table S1. Thermochemistry data [in hartree].

	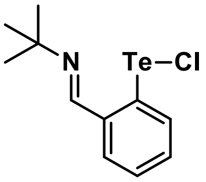	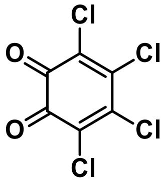	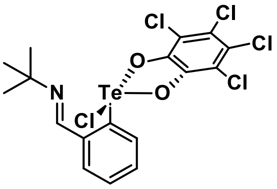
**E**	–1210.7641	–2219.7382	–3430.5490
**ZPE**	0.2256	0.0473	0.2750
**E+ZPE**	–1210.5384	–2219.6910	–3430.2740
**H**	–1210.5226	–2219.6790	–3430.2461
**G**	–1210.5814	–2219.7303	–3430.3333

Table S2. Thermochemistry data [in hartree].

	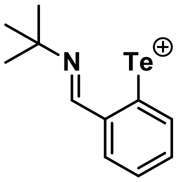	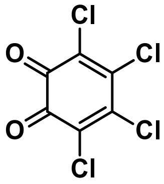	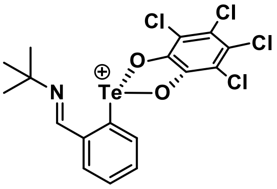
**E**	–750.3686	–2219.7382	–2970.1472
**ZPE**	0.2252	0.0473	0.2750
**E+ZPE**	–750.1434	–2219.6910	–2969.8721
**H**	–750.1296	–2219.6790	–2969.8467
**G**	–750.1837	–2219.7303	–2969.9275

Table S3. Thermochemistry data [in hartree].

	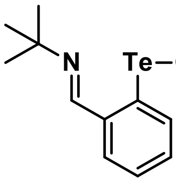	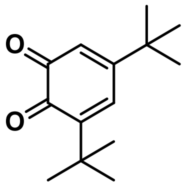	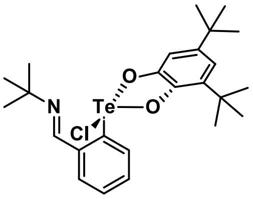	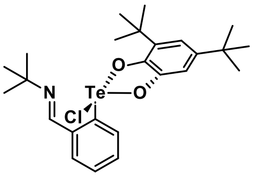
**E**	–1210.7641	–695.9904	–1906.7854	–1906.7876
**ZPE**	0.2256	0.3090	0.5364	0.5362
**E+ZPE**	–1210.5384	–695.6814	–1906.2490	–1906.2515
**H**	–1210.5226	–695.6631	–1906.2150	–1906.2174
**G**	–1210.5814	–695.7254	–1906.3131	–1906.3160

Table S4. Thermochemistry data [in hartree].

	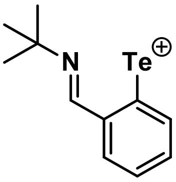	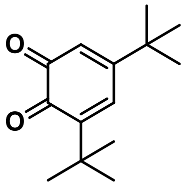	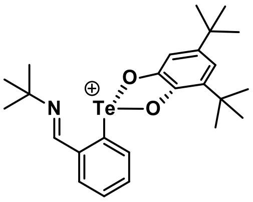	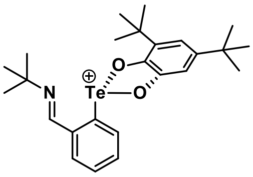
**E**	–750.3686	–695.9904	–1446.3923	–1446.3948
**ZPE**	0.2252	0.3090	0.5368	0.5366
**E+ZPE**	–750.1434	–695.6814	–1445.8555	–1445.8582
**H**	–750.1296	–695.6631	–1445.8239	–1445.8265
**G**	–750.1837	–695.7254	–1445.9154	–1445.9187

Table S5. Thermochemistry data [in hartree].

	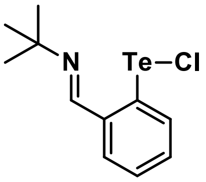	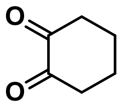	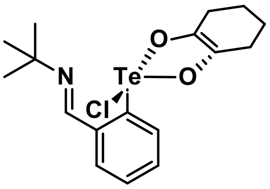
**E**	–1210.7641	–383.9050	–1594.6557
**ZPE**	0.2256	0.1313	0.3587
**E+ZPE**	–1210.5384	–383.7737	–1594.2970
**H**	–1210.5226	–383.7655	–1594.2730
**G**	–1210.5814	–383.8050	–1594.3503

Table S6. Thermochemistry data [in hartree].

	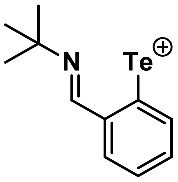	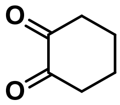	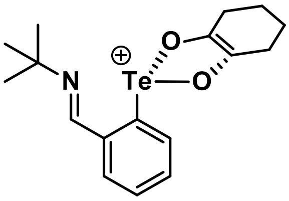
**E**	–750.3686	–383.9050	–1134.2660
**ZPE**	0.2252	0.1313	0.3592
**E+ZPE**	–750.1434	–383.7737	–1133.9068
**H**	–750.1296	–383.7655	–1133.8853
**G**	–750.1837	–383.8050	–1133.9560

Table S7. Thermochemistry data [in hartree].

	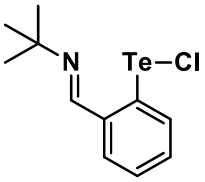	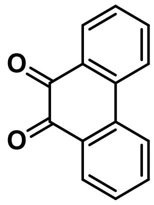	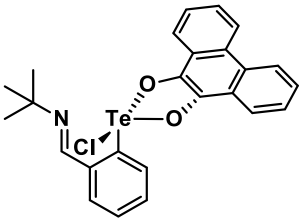
**E**	–1210.7641	–688.7637	–1899.5401
**ZPE**	0.2256	0.1801	0.4070
**E+ZPE**	–1210.5384	–688.5836	–1899.1332
**H**	–1210.5226	–688.5714	–1899.1050
**G**	–1210.5814	–688.6214	–1899.1916

Table S8. Thermochemistry data [in hartree].

	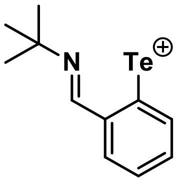	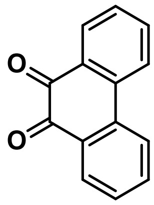	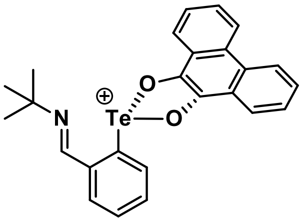
**E**	–750.3686	–688.7637	–1439.1451
**ZPE**	0.2252	0.1801	0.4074
**E+ZPE**	–750.1434	–688.5836	–1438.7377
**H**	–750.1296	–688.5714	–1438.7119
**G**	–750.1837	–688.6214	–1438.7931

Table S9. Thermochemistry data [in hartree].

	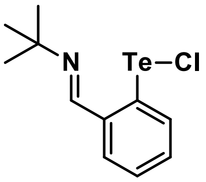	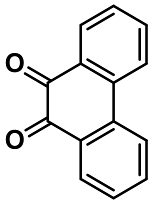	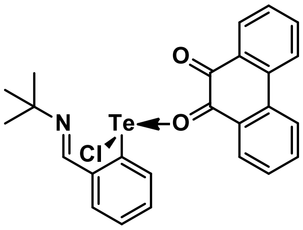
**E**	–1210.7641	–688.7637	–1899.5282
**ZPE**	0.2256	0.1801	0.4069
**E+ZPE**	–1210.5384	–688.5836	–1899.1213
**H**	–1210.5226	–688.5714	–1899.0924
**G**	–1210.5814	–688.6214	–1899.1820

Table S10. Thermochemistry data [in hartree].

	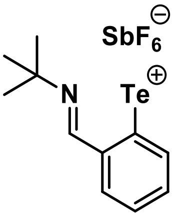	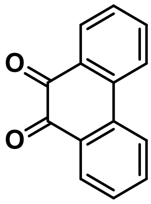	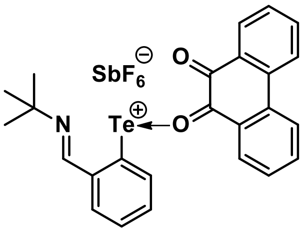
**E**	–1590.1522	–688.7637	–2278.9399
**ZPE**	0.2394	0.1801	0.4201
**E+ZPE**	–1589.9127	–688.5836	–2278.5199
**H**	–1589.8886	–688.5714	–2278.4822
**G**	–1589.9687	–688.6214	–2278.5939

Table S11. Thermochemistry data [in hartree].

	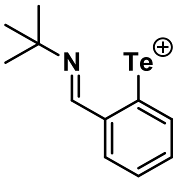	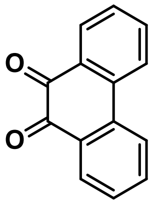	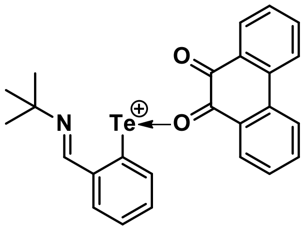
**E**	–750.3686	–688.7637	–1439.1586
**ZPE**	0.2252	0.1801	0.4067
**E+ZPE**	–750.1434	–688.5836	–1438.7519
**H**	–750.1296	–688.5714	–1438.7250
**G**	–750.1837	–688.6214	–1438.8106

Table S12. AIM derived topological bond properties of selected bonds of the quinone **o-q^phen^** as well as **[I·(*o*-q^phen^)][SbF6]** and **[I·(*o*-q^phen^)]^+^**.

Species	d [Å]	ρ(r) [eÅ^{-3}]	∇^2ρ(r) [eÅ^{-5}]	ε	G/ρ(r) [a.u.]	H/ρ(r) [a.u.]	δ
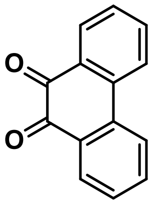							
**O23-C10**	1.212	2.83	–4.9	0.06	1.69	–1.82	1.37
**O24-C7**	1.212	2.83	–4.9	0.06	1.69	–1.82	1.37
**C7-C10**	1.542	1.69	–14.2	0.08	0.22	-0.80	0.85
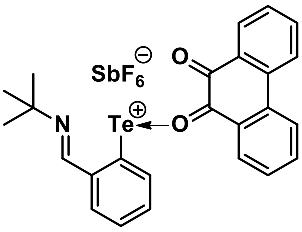							
**Te1-N2**	2.109	0.73	4.6	0.25	0.87	–0.42	0.82
**Te1-O3**	2.542	0.26	2.4	0.16	0.71	–0.06	0.28
**Te1-O4**	3.023	0.11	1.1	0.04	0.66	0.05	0.12
**Te1-C5**	2.072	0.90	–0.1	0.26	0.56	–0.57	1.08
**O3-C30**	1.227	2.72	–5.6	0.03	1.64	–1.78	1.26
**O4-C31**	1.215	2.81	–5.3	0.06	1.68	–1.81	1.34
**C30-C31**	1.529	1.74	–15.2	0.07	0.21	–0.83	0.85
**Te1-F55**	3.507	0.05	0.5	0.02	0.67	0.10	0.04
**Te1-F53**	3.553	0.04	0.5	0.15	0.71	0.12	0.04
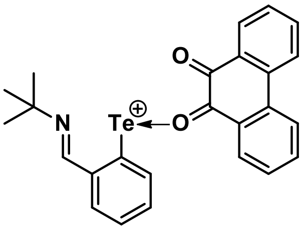							
**Te1-N2**	2.142	0.68	4.2	0.23	0.84	–0.40	0.78
**Te1-O3**	2.479	0.29	2.7	0.20	0.74	–0.08	0.33
**Te1-O4**	2.925	0.13	1.3	0.07	0.68	0.04	0.15
**Te1-C5**	2.087	0.88	–0.2	0.26	0.54	–0.56	1.07
**O3-C30**	1.229	2.69	–5.0	0.04	1.65	–1.78	1.26
**O4-C31**	1.216	2.81	–5.4	0.07	1.67	–1.81	1.34
**C30-C31**	1.529	1.74	–15.1	0.08	0.22	–0.83	0.86
**Te1-N2**	2.142	0.68	4.2	0.23	0.84	–0.40	0.78

Crystallographic data for studied compounds

Table S13. Crystal data and structure refinement.

	**[I(cat^{Cl})]Cl**	**[I·(*o*-q^{phen})][SbF$_6$]**
Formula	2($C_{17}H_{14}Cl_5NO_2Te$).3(CH_2Cl_2)	$C_{25}H_{22}NO_2TeF_6Sb$
Formula weight, g mol^{-1}	1393.06	731.78
Crystal system	Monoclinic	Monoclinic
Crystal size, mm	0.59 × 0.31 × 0.27	0.48 × 0.07 × 0.03
Space group	C2/c	$P2_1/n$
a, Å	8.6458(3)	9.1439(2)
b, Å	22.9275(9)	15.8227(5)
c, Å	25.3603(10)	16.9957(6)
α, °	90	90
β, °	93.0420(10)	98.1190(10)
γ, °	90	90
V, Å^3	5020.0(3)	2434.31(13)
Z	4	4
ρ_{calcd}, Mg m^{-3}	1.843	1.997
μ (Mo $K\alpha$), mm^{-1}	2.054	2.378
F(000)	2712	1408
θ range, deg	1 to 27.5	1 to 28
Index ranges	$-11 \le h \le 10$	$-12 \le h \le 12$
	$-29 \le k \le 29$	$-21 \le k \le 20$
	$-32 \le l \le 32$	$-22 \le l \le 22$
No. of reflns collected	66040	82412
No. indep. Reflns	5777	6018
No. obsd reflns with ($I>2\sigma(I)$)	5479	5345
No. refined params	280	328
GooF (F^2)	1.105	1.062
R_1 (F) ($I > 2\sigma(I)$)	0.028	0.021
wR_2 (F^2) (all data)	0.054	0.046
Largest diff peak/hole, e Å^{-3}	1.292/–1.240	1.055/–0.567
CCDC	2516256	2516259

$R_{int} = \sum|F_o^2 - F_{o,mean}^2|/\sum F_o^2$, $S = [\sum(w(F_o^2 - F_c^2)^2)/(N_{diffrs} - N_{params})]^{½}$ for all data, $R(F) = \sum||F_o| - |F_c||/\sum|F_o|$ for observed data, $wR(F^2) = [\sum(w(F_o^2 - F_c^2)^2)/(\sum w(F_o^2)^2)]^{½}$ for all data.

Table S13 (continuation). Crystal data and structure refinement.

	**5**	**6**	**7.(benzene)**
Formula	$C_{32}H_{36}O_2Si$	$C_{32}H_{24}O_2Si$ 0.5(C_6H_6)	$C_{12}Cl_6O_4$ 3(C_6H_6)
Formula weight, g mol^{-1}	480.70	507.65	655.14
Crystal system	Monoclinic	Triclinic	Monoclinic
Crystal size, mm	0.41 × 0.26 × 0.16	0.22 × 0.21 × 0.15	0.30 × 0.26 × 0.06
Space group	$P2_1/n$	P-1	$P2_1/c$
a, Å	12.8640(2)	9.6477(2)	17.2138(4)
b, Å	10.0366(2)	11.7684(3)	7.4322(2)
c, Å	21.4659(5)	12.6341(3)	23.5881(6)
α, °	90	109.7620(10)	90
β, °	101.7060(10)	94.1990(10)	110.3040(10)
γ, °	90	101.9680(10)	90
V, Å^3	2713.84(8)	1304.52(5)	2830.27(12)
Z	4	2	4
ρ_{calcd}, Mg m^{-3}	1.117	1.292	1.538
μ (Mo *Kα*), mm^{-1}	0.113	0.122	0.644
F(000)	1032	534	1328
θ range, deg	1 to 28	1 to 28	1 to 28
Index ranges	$-17 \le h \le 17$	$-12 \le h \le 12$	$-22 \le h \le 22$
	$-13 \le k \le 13$	$-15 \le k \le 15$	$-9 \le k \le 9$
	$-28 \le l \le 28$	$-16 \le l \le 16$	$-31 \le l \le 31$
No. of reflns collected	71696	66427	89248
No. indep. Reflns	6710	6437	7013
No. obsd reflns with ($I>2\sigma(I)$)	5834	5501	5767
No. refined params	325	346	361
GooF (F^2)	1.030	1.056	1.073
R_1 (*F*) ($I > 2\sigma(I)$)	0.036	0.038	0.034
wR_2 (F^2) (all data)	0.091	0.095	0.076
Largest diff peak/hole, e Å^{-3}	0.345/–0.229	0.348/–0.253	0.315/–0.284
CCDC	2516258	2516257	2516260

$R_{int} = \sum|F_o^2 - F_{o,mean}^2|/\sum F_o^2$, S = $[\sum(w(F_o^2 - F_c^2)^2)/(N_{diffrs} - N_{params})]^{½}$ for all data, $R(F) = \sum||F_o| - |F_c||/\sum|F_o|$ for observed data, $wR(F^2) = [\sum(w(F_o^2 - F_c^2)^2)/(\sum w(F_o^2)^2)]^{½}$ for all data.

Table S13 (continuation). Crystal data and structure refinement.

	**8**	**9**	**12**
Formula	$C_{18}H_{10}Cl_4O_2Si$	$C_{26}H_{30}O_2Si$	$C_{52}H_{62}O_5Si_2$
Formula weight, g mol^{-1}	428.15	402.59	823.19
Crystal system	Triclinic	Triclinic	Monoclinic
Crystal size, mm	0.59 × 0.23 × 0.20	0.22 × 0.20 × 0.10	0.36 × 0.11 × 0.11
Space group	P-1	P-1	$P2_1/c$
a, Å	8.0303(8)	9.9300(5)	9.6844(2)
b, Å	10.4059(8)	10.2104(4)	20.7840(4)
c, Å	11.9656(13)	12.2894(8)	23.0537(5)
α, °	68.166(6)	68.814(3)	90
β, °	71.400(6)	78.635(3)	95.6640(10)
γ, °	80.429(6)	79.491(2)	90
V, Å^3	878.38(55)	1130.56(11)	4617.61(16)
Z	2	2	4
ρ_{calcd}, Mg m^{-3}	1.619	1.183	1.184
μ (Mo *Kα*), mm^{-1}	0.751	0.123	0.123
F(000)	423	432	1768
θ range, deg	1 to 27.5	1 to 27.5	1 to 28
Index ranges	$-10 \le h \le 10$	$-12 \le h \le 12$	$-12 \le h \le 12$
	$-13 \le k \le 13$	$-13 \le k \le 13$	$-27 \le k \le 27$
	$-15 \le l \le 15$	$-15 \le l \le 15$	$-30 \le l \le 30$
No. of reflns collected	27718	39681	207413
No. indep. Reflns	4014	5139	11443
No. obsd reflns with ($I>2\sigma(I)$)	3728	4817	10004
No. refined params	226	268	550
GooF (F^2)	1.084	1.028	1.026
R_1 (*F*) ($I > 2\sigma(I)$)	0.026	0.035	0.035
wR_2 (F^2) (all data)	0.063	0.087	0.094
Largest diff peak/hole, e Å^{-3}	0.315/–0.285	0.347/–0.262	0.342 / –0.317
CCDC	2516252	2516253	2516262

$R_{int} = \sum|F_o^2 - F_{o,mean}^2|/\sum F_o^2$, $S = [\sum(w(F_o^2 - F_c^2)^2)/(N_{diffrs} - N_{params})]^{½}$ for all data, $R(F) = \sum||F_o| - |F_c||/\sum|F_o|$ for observed data, $wR(F^2) = [\sum(w(F_o^2 - F_c^2)^2)/(\sum w(F_o^2)^2)]^{½}$ for all data.

Table S13 (continuation). Crystal data and structure refinement.

	**13**	**[16.(dmso)$_2$]**
Formula	$C_{52}H_{38}O_5Si_2$	$C_{32}H_{52}O_6S_2Si$. .2(C_2H_6OS)
Formula weight, g mol^{-1}	799.00	781.20
Crystal system	Triclinic	Triclinic
Crystal size, mm	0.59 × 0.25 × 0.10	0.37 × 0.12 × 0.06
Space group	P-1	P-1
a, Å	9.5233(4)	10.0260(14)
b, Å	9.6490(3)	11.6179(17)
c, Å	11.8452(5)	21.138(3)
α, º	78.1230(10)	83.739(6)
β, º	67.022(2)	86.661(6)
γ, º	76.454(2)	64.535(5)
V, Å^3	966.23(7)	2209.8(6)
Z	1	2
ρ_{calcd}, Mg m^{-3}	1.373	1.174
μ (Mo $K\alpha$), mm^{-1}	0.145	0.285
F(000)	423	844
θ range, deg	1 to 26.5	1 to 28.5
Index ranges	$-11 \le h \le 11$	$-13 \le h \le 13$
	$-12 \le k \le 12$	$-15 \le k \le 15$
	$-14 \le l \le 14$	$-28 \le l \le 28$
No. of reflns collected	32753	112559
No. indep. Reflns	3818	11103
No. obsd reflns with ($I>2\sigma(I)$)	3477	8259
No. refined params	271	506
GooF (F^2)	1.029	0.996
R_1 (F) ($I > 2\sigma(I)$)	0.037	0.125
wR_2 (F^2) (all data)	0.095	0.334
Largest diff peak/hole, e Å^{-3}	0.385/–0.317	1.286/–0.879
CCDC	2516255	2516261

$R_{int} = \sum|F_o^2 - F_{o,mean}^2|/\sum F_o^2$, S = $[\sum(w(F_o^2 - F_c^2)^2)/(N_{diffrs} - N_{params})]^{½}$ for all data, $R(F) = \sum||F_o| - |F_c||/\sum|F_o|$ for observed data, $wR(F^2) = [\sum(w(F_o^2 - F_c^2)^2)/(\sum w(F_o^2)^2)]^{½}$ for all data.

References

1. Lang, A.; Nöth, H.; Thomann-Albach, M., Contributions of the Chemistry of Boron, 236. In Quest of New and Stable Bis(organyloxy)boranes (RO)2BH for Catalytic Hydroboration. *Chemische Berichte* **1997,** *130* (3), 363-370.
2. Hejda, M.; Doležal, L.; Erben, M.; Hupf, E.; Růžička, A.; Beckmann, J.; Dostál, L., Redox Cycling at Tellurium: Selective and Multiple Activation of Si–H Bonds in Common Organosilanes and SiH4. *Chemistry – A European Journal* **2025,** *31* (43), e202502141.
